# Global, regional, and national burden of neonatal diseases attributable to particulate matter pollution from 1990 to 2021

**DOI:** 10.3389/fpubh.2025.1556340

**Published:** 2025-06-09

**Authors:** Hui Li, Lifang Liang, Zhenyu Song, Yongfeng Li

**Affiliations:** ^1^GMU-GIBH Joint School of Life Sciences, The Guangdong-Hong Kong-Macao Joint Laboratory for Cell Fate Regulation and Diseases, Guangzhou Medical University, Guangzhou, China; ^2^Guangxi Key Laboratory of Reproductive Health and Birth Defect Prevention, Maternal and Child Health Hospital of Guangxi Zhuang Autonomous Region, Nanning, China; ^3^Laboratory Animal Center of Guangxi Medical University, Nanning, China; ^4^School of Public Health, Guangxi Medical University, Nanning, China

**Keywords:** neonatal disease, particulate matter pollution, epidemiology, global burden of disease (GBD), public health

## Abstract

**Background:**

The impact of particulate matter pollution (PMP) on neonatal health has garnered growing public attention. However, the global burden of PMP-related neonatal diseases remains insufficiently characterized. This study aimed to evaluate the current burden and temporal trends (1990–2021) of PMP-related neonatal diseases.

**Methods:**

We used data from the 2021 Global Burden of Disease Study (GBD) to estimate disability-adjusted life years (DALYs) of neonatal diseases attributed to PMP. Our analysis included DALY trends by age, gender, and sociodemographic index (SDI) from 1990 to 2021 at global, regional, and national levels. We employed health inequality analysis and frontier analysis to quantify the factors that contribute to the neonatal diseases burden attributed to PMP.

**Results:**

In 2021, the global age-standardized DALYs of neonatal diseases attributed to PMP, household air pollution (HAP), and ambient particulate matter pollution (APMP) were 723.06/100,000 (95% UI: 610.39, 845.18), 518.10/100,000 (95% UI: 410.06, 641.68), and 204.81/100,000 (95% UI: 121.31, 311.25), respectively. From 1990 to 2021, PMP- and HAP-related neonatal disease burdens declined significantly, whereas APMP-related DALYs increased in low-middle SDI regions. Age-specific DALYs showed a gradual downward trend, and male DALYs were higher than female DALYs in all age groups. DALYs of neonatal diseases attributed to PMP, HAP, and APMP were negatively correlated with SDI. Frontier analysis indicated that urgent action was required to alleviate the burden of neonatal diseases attributed to PMP in countries such as Mali, South Sudan, the Central African Republic, Sierra Leone, and Nigeria.

**Conclusion:**

The burden of neonatal disease attributed to PMP remains a major health problem worldwide, especially in low SDI regions. This suggests that future air pollution-induced neonatal disease responses should emphasize health equity. Low SDI regions should be prioritized when allocating resources to address climate change.

## 1 Introduction

Neonatal diseases constitute a critical global public health challenge due to their multifactorial etiology and heterogeneous manifestations. Globally, an estimated 2.7 million newborns die within the first 28 days of life annually (approximately 7,000 daily), representing 47% of all under-five childhood mortality (1). The World Health Organization (WHO) estimates that environmental factors contribute to over 28% of under-five mortality, including neonatal disease-related deaths. Particulate matter pollution (PMP) has emerged as a major public health concern due to its adverse effects on neonatal health. As a ubiquitous form of air pollution, PMP is divided into ambient particulate matter pollution (APMP) and household air pollution (HAP). PMP is significantly associated with a variety of human diseases, including health problems in newborns. Studies have shown that exposure to PMP during pregnancy, especially PM_2.5_, may increase the risk of low birth weight and affect the growth and development of newborns (2). In addition, toxic substances such as polycyclic aromatic hydrocarbons (PAHs) in PMP can reach organs throughout the body through blood circulation, affecting the placenta and fetus, possibly causing endocrine disorders and placental damage, thereby affecting newborn health (3, 4). Given that the relationship between PMP and neonatal diseases poses a major public health challenge, it is critical to understand the epidemiological characteristics of the neonatal diseases burden attributed to PMP.

It is worth noting that the current research on the relationship between neonatal diseases and PMP has not covered all aspects. Previous studies primarily focused on specific neonatal diseases related to PMP, lacking a comprehensive exploration of the varying roles of different PMP subtypes in neonatal diseases (2, 5–7). To fill this gap, we conducted the first comprehensive assessment of the total burden of neonatal disability-adjusted life years (DALYs) attributed to PMP. Furthermore, we specifically analyzed two specific subtypes of PMPs, APMP and HAP, and explored the potential impact of age, gender, sociodemographic index (SDI), and geographic location on the total burden of neonatal DALY attributed to PMP.

In this study, we elucidate the impact of PMP on neonatal health by analyzing the global, regional, and national burden of neonatal diseases attributed to PMP from 1990 to 2021 and by examining the independent effects of age, gender, and SDI. Utilizing health inequality analysis and frontier analysis, we estimate the theoretical minimum of DALYs for PMP-related neonatal diseases, accounting for varying SDI levels across countries. We present a comprehensive epidemiological perspective on the impact of PMP on the burden of neonatal diseases. Thus, we offer an epidemiological reference for government officials to formulate policies for neonatal disease management.

## 2 Materials and methods

### 2.1 Study data

To investigate the burden of neonatal disease attributed to PMP, we selected the 2021 Global Burden of Disease Study (GBD) data from the Global Health Data Exchange GBD Results Tool.^1^ To our knowledge, the GBD database is the most comprehensive and extensive public database of neonatal disease and PMP data. The GBD collaborators developed this tool to build the most comprehensive and extensive public epidemiological database designed to quantifying global health and disease trends (8, 9). It provides a comprehensive assessment of disability life expectancy and prevalence of 371 diseases and injuries, and age-and gender-specific mortality rates for 288 causes in 204 countries and regions. For a more detailed introduction to the GBD database, please refer to the references below (10). The open-source data used in this article are available at GBD 2021,^2^ including (1) global, regional, and national age-specific rates of DALYs from neonatal diseases attributed to PMP, HAP, and APMP in three gender groups (Both, Male and Female) from 1990 to 2021; (2) global, regional, and national age-standardized rates of DALYs from neonatal diseases attributed to PMP, HAP, and APMP in three gender groups from 1990 to 2021; (3) SDIs for 204 countries and regions from 1990 to 2021.

### 2.2 Study variables

#### 2.2.1 Outcome variables

The outcome variable was DALYs due to neonatal diseases, defined by GBD 2021 in alignment with the WHO as conditions occurring within the first 28 days of life. Neonatal diseases were classified under International Classification of Diseases (ICD) 10 codes P07, P91.6, P39, and P58. DALYs were selected to assess the burden of PMP-related neonatal diseases, as they capture both mortality and morbidity, offering a comprehensive measure of overall disease burden (11).

#### 2.2.2 Main independent variable

The main independent variable in our analysis comprised PMP, which is an overall measure of the sum of HAP and APMP. HAP is influenced by factors such as the number of people using solid fuels and particulate matter levels, while APMP represents the average mass concentration of PM2.5 per cubic meter of air, estimated through satellite observations and ground measurements (12, 13). In addition to this main independent variable, we conducted a series of subgroup analyses to examine the effects of the PMP and its composites on neonatal diseases based on SDI, gender, and age. The SDI was defined as a composite indicator that was directly proportional to per capita income and average years of education, and inversely proportional to the total fertility rate among women under 25 years of age (11). GBD2021 divides 204 countries and regions into five levels of economic development based on SDI values: high SDI, high-middle SDI, middle SDI, low-middle SDI, and low SDI (14, 15). This study used SDI as an indicator to measure the economic development status of countries and regions and further analyzed the impact of economic development level on neonatal diseases attributed to PMP. We included the variable gender in the subgroup analyses, which was defined as Male, Female, and Both. Finally, age was classified as early neonatal period and late neonatal period.

### 2.3 Statistical analysis

In this study, we used the latest version of Joinpoint software (version 5.1.0) to perform trend analysis and calculate the annual percentage change (APC) and average annual percentage change (AAPC) (16). This statistical tool has been widely cited in many top academic publications (17, 18). Joinpoint regression analysis is a powerful tool for evaluating disease epidemic dynamics. The Joinpoint model can identify trend change points and analyze the overall trend within a time period, which is of great significance for analyzing disease epidemic trends (16). The Joinpoint model determines its parameters through rigorous statistical tests, making the model results more credible (19). In addition, we used the Spearman correlation coefficient to assess the association between SDI and age-standardized neonatal DALYs. Absolute and relative health inequalities were captured by the slope index and concentration index, respectively, which were extracted from the health inequality package. Health inequalities are measurable differences in health between population subgroups defined by social, economic, geographic, or demographic characteristics (20, 21). The slope index is derived by weighted regression analysis of all age groups with a measure of relative position related to the SDI, expressed as the midpoint of the cumulative range of the population sorted by SDI (22). The concentration index is determined by numerically calculating the area under the Lorenz concentration curve, which is determined using the relative cumulative scores and the cumulative relative population distribution based on the SDI (23). Frontier analysis is a statistical method for evaluating efficiency and performance. It identifies the “frontier” or best-performing entities in the data to assess the performance of other entities in terms of efficiency, productivity, or other related indicators (24, 25). It is mainly used to analyze the efficiency differences between different countries or regions in terms of disease control, health resource utilization, etc. Frontier analysis was used to assess the ideal DALYs for neonatal diseases for 204 countries and regions at a specific SDI level. Through this analysis, we identified the countries and regions with the most significant differences in neonatal disease DALYs performance from the frontier analysis results, including (1) the top 15 countries with the largest differences in DALYs from the frontier globally; (2) the top 5 countries with the smallest differences from the frontier boundary value among low SDI (SDI < 0.5) countries; (3) the top 5 countries with the largest differences from the frontier DALYs level among high SDI (SDI ≥ 0.85) countries. To accurately determine the specific number of countries and regions included in each category, we referred to previous research literature in the relevant field (26). We used R 4.4.0 and Stata 16.0 to perform all statistical analyses and data visualization. In the statistical analyses, a *p* value of less than 0.05 indicated a statistically significant difference. We considered it statistically significant when the 95% uncertainty interval (UI) did not contain zero. In addition, all rate ratios are reported per 100,000 people.

## 3 Results

### 3.1 Spatiotemporal patterns of neonatal diseases attributable to PMP

**Figure 1:**
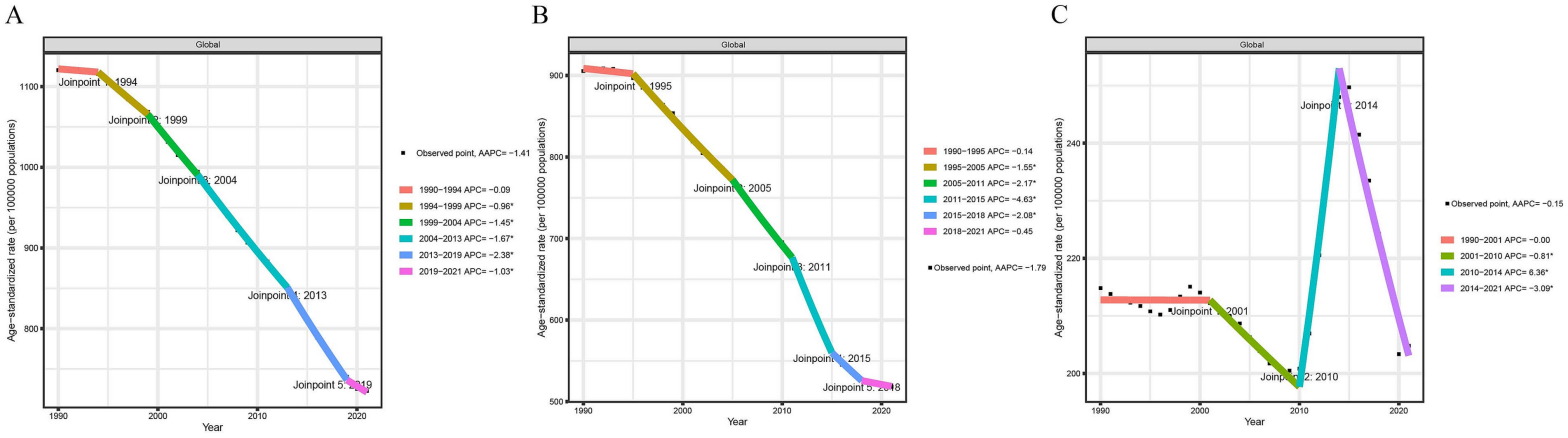
Trends for different regions from 1990 to 2021 based on joinpoint regression model, the sequence of each line represents global. **(A)** Neonatal diseases DALYs attributable to PMP, **(B)** neonatal diseases DALYs attributable to HAP, **(C)** neonatal diseases DALYs attributable to APMP. SDI, socio-demographic index; DALYs, disability-adjusted life years; PMP, particulate matter pollution; HAP, household air pollution; APMP, ambient particulate matter pollution.

**Figure 2:**
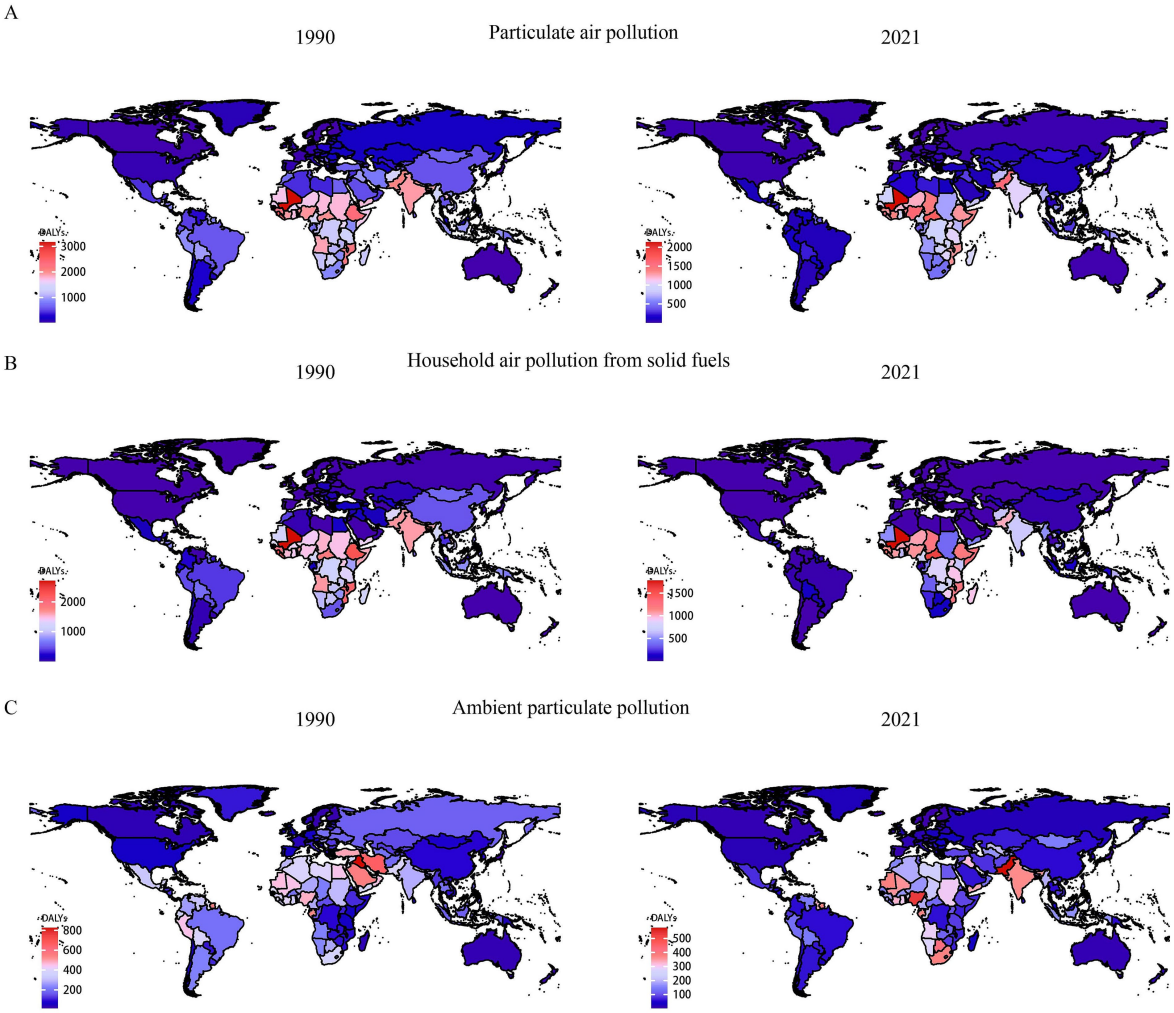
Comparison of neonatal diseases DALYs in 1990 and 2021 in 204 countries and territories worldwide. **(A)** Neonatal diseases DALYs attributable to PMP, **(B)** neonatal diseases DALYs attributable to HAP, **(C)** neonatal diseases DALYs attributable to APMP. DALYs, disability-adjusted life years; APMP, ambient particulate matter pollution; HAP, household air pollution; PMP, particulate matter pollution.

In 2021, the global age-standardized DALYs for neonatal diseases attributed to PMP, HAP, and APMP were 723.06 per 100,000 (95% UI: 610.39, 845.18), 518.10 per 100,000 (95% UI: 410.06, 641.68), and 204.81 per 100,000 (95% UI: 121.31, 311.25), respectively. Joinpoint regression analysis was used to evaluate the DALYs of neonatal diseases from 1990 to 2021 and to calculate the AAPCs and APCs (Supplementary Tables S1, S2). The DALYs of neonatal diseases attributed to PMP and HAP showed a downward trend globally and at the SDI level, with the most significant decrease in the high SDI region. Similarly, the neonatal diseases DALYs attributable to APMP showed a decreasing trend at both the global level and the five SDI levels (Figure 1 and Supplementary Table S1). The DALYs of neonatal diseases attributable to APMP in the low-middle SDI region are significantly higher than those in other regions, indicating that the impact of APMP is closely related to local socioeconomic conditions. Regionally, between 1990 and 2021, the most significant increase in DALYs of neonatal diseases attributable to APMP was observed in South Asia, Oceania, and Central Sub-Saharan Africa. In contrast, neonatal DALYs attributed to HAP decreased overall, with the most significant reduction in the high-income Asia Pacific region (Supplementary Figure S1). To further illustrate these results, we mapped data for 204 countries and regions, revealing a high disease burden in Africa (Figure 2). This suggests that although PMP pollution control is effective globally, regions such as Africa still need to pay more attention to APMP. Next, we found that Africa had the largest AAPC value during 1990–2021, whereas Europe had a lower AAPC value (Supplementary Table S1). This may be closely related to socioeconomic factors.

### 3.2 Association with the SDI, gender and age

**Figure 3:**
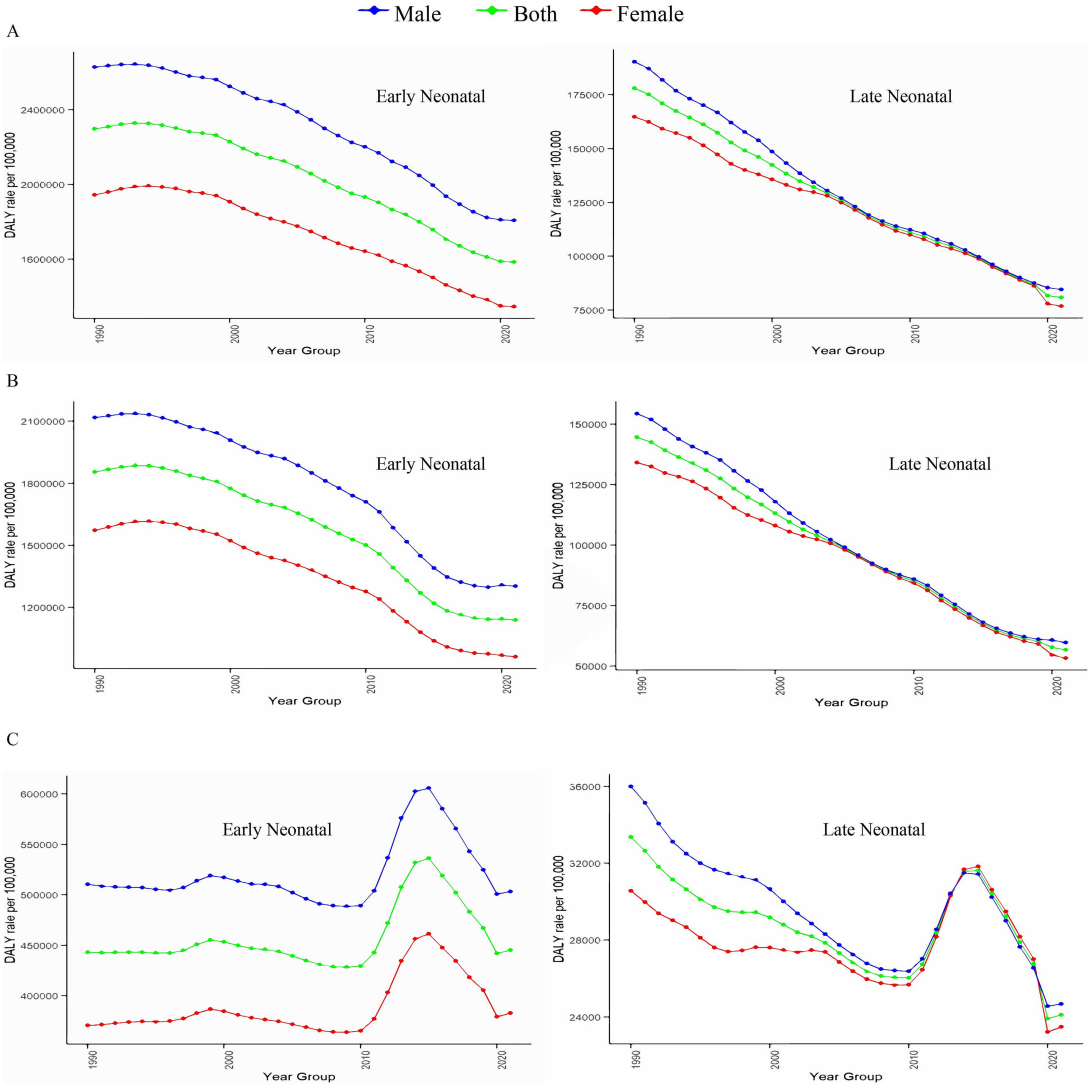
Associations between DALYs of neonatal diseases and gender, and age. **(A)** Neonatal diseases DALYs attributable to PMP, **(B)** neonatal diseases DALYs attributable to HAP, **(C)** neonatal diseases DALYs attributable to APMP. DALYs, disability-adjusted life years; PMP, particulate matter pollution; HAP, household air pollution; APMP, ambient particulate matter pollution.

**Figure 4:**
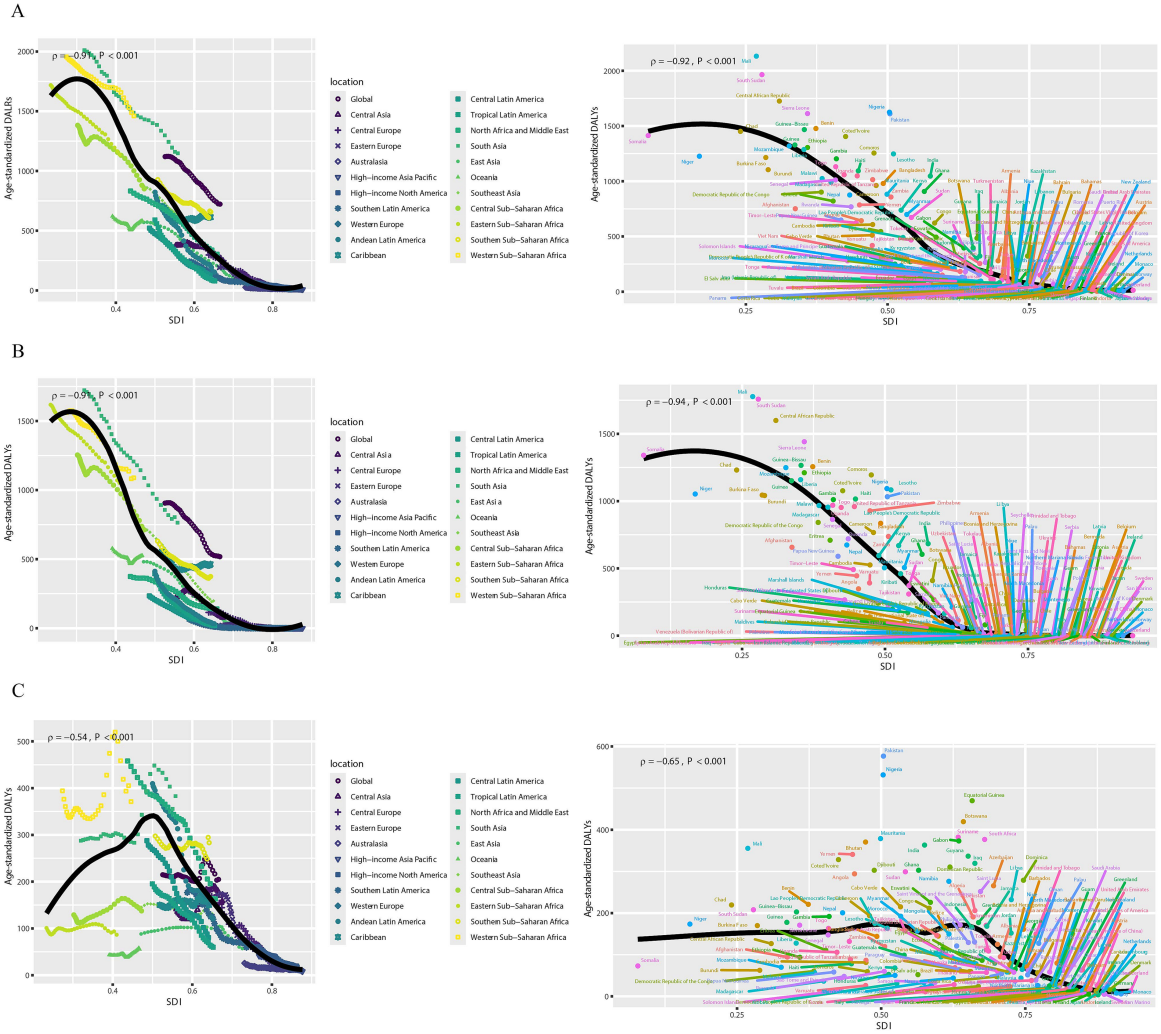
Associations between DALYs of neonatal diseases and SDI. **(A)** Neonatal diseases DALYs attributable to PMP, **(B)** neonatal diseases DALYs attributable to HAP, **(C)** neonatal diseases DALYs attributable to APMP. DALYs, disability-adjusted life years; SDI, socio-demographic index; APMP, ambient particulate matter pollution; HAP, household air pollution; PMP, particulate matter pollution.

Figure 3 and Supplementary Figure S2 show the age-specific DALYs of neonatal diseases attributable to PMP, HAP, and APMP globally in 2021 and their trends from 1990 to 2021. Globally, males had higher DALYs of neonatal diseases than females in the early neonatal period, whereas DALYs became similar for males and females in the late neonatal period (Figure 3). Additionally, we plotted subgroup joinpoint regression of AAPC values for three gender categories (Both, Female, and Male) globally and for the five SDI regions (Supplementary Figure S2). Notably, males had higher DALYs of neonatal diseases attributable to APMP, HAP, and PMP than females across all regions. To explore the relationship between SDI and DALYs of neonatal diseases, we conducted a Spearman correlation analysis (Figure 4). At both national and regional levels, DALYs of neonatal diseases attributed to PMP, HAP, and APMP were negatively correlated with SDI (*p* < 0.001), indicating that higher SDI is associated with lower DALYs of neonatal diseases from these causes.

### 3.3 Health inequality analysis

**Figure 5:**
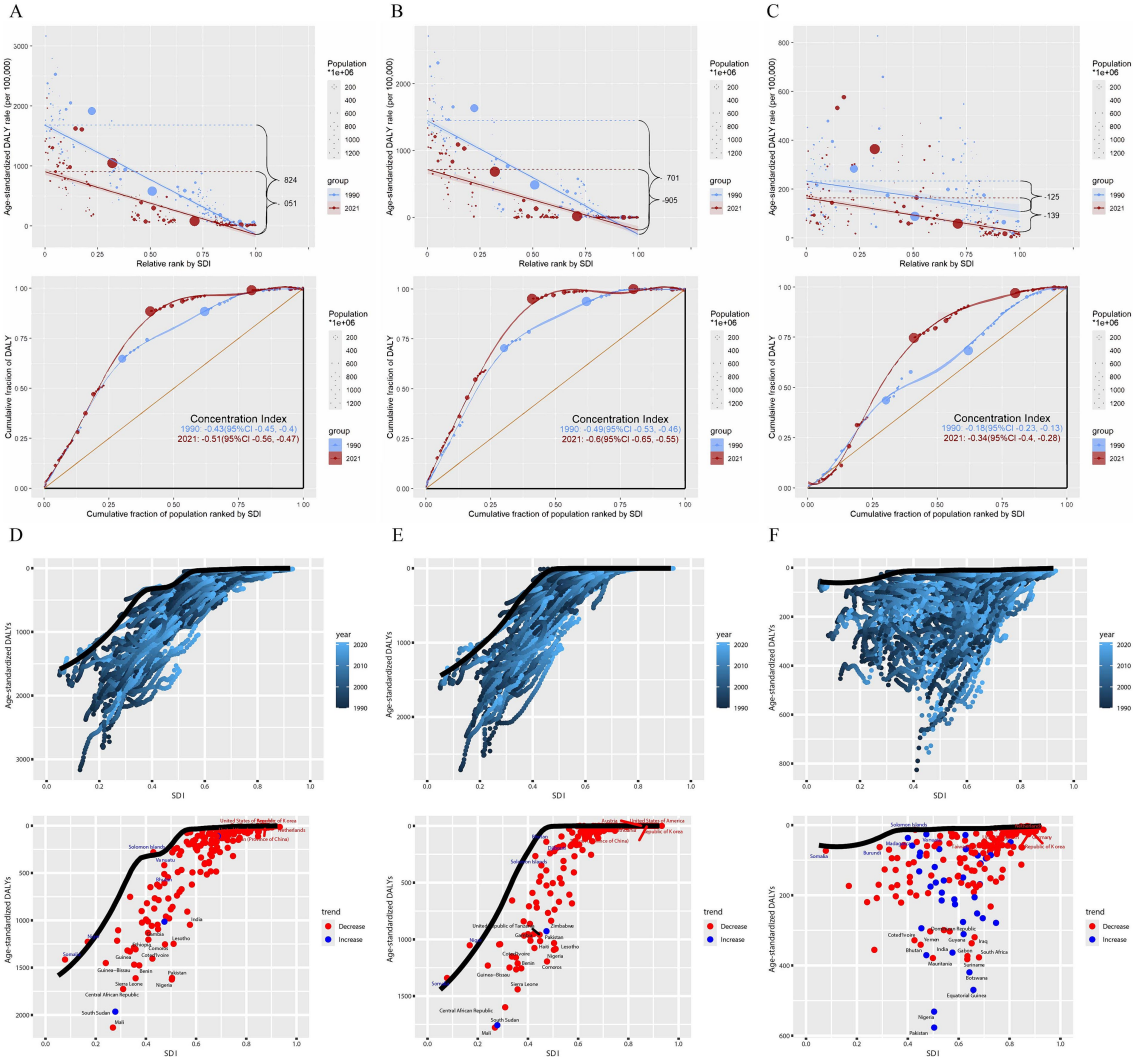
Health inequality analysis and frontier analysis for neonatal diseases DALYs. **(A)** Neonatal diseases DALYs attributable to PMP, **(B)** neonatal diseases DALYs attributable to HAP, **(C)** neonatal diseases DALYs attributable to APMP; **(D)** neonatal diseases DALYs attributable to PMP, **(E)** neonatal diseases DALYs attributable to HAP, **(F)** neonatal diseases DALYs attributable to APMP. SDI, socio-demographic index; DALYs, disability-adjusted life years; APMP, ambient particulate matter pollution; HAP, household air pollution; PMP, particulate matter pollution.

Continuing with the above conclusion that DALYs from neonatal diseases are highly correlated with SDI, we further analyzed health inequality (Figures 5A–C and Supplementary Table S3). The slope index of DALYs from neonatal diseases attributed to APMP decreased from −124.65 (95% CI: −177.22, −72.09) in 1990 to −139.45 (95% CI: −166.94, −111.96) in 2021. Additionally, its concentration index decreased from −0.18 (95% CI: −0.23, −0.13) to −0.34 (95% CI: −0.40, −0.28) in 2021. Interestingly, the slope index of DALYs from neonatal diseases attributed to PMP showed an inverse trend, increasing from −1824.34 (95% CI: −1927.32, −1721.37) in 1990 to −1051.39 (95% CI: −1146.15, −956.64) in 2021, while the concentration index decreased from −0.43 (95% CI: −0.45, −0.40) to −0.51 (95% CI: −0.56, −0.47) in 2021. Similarly, the slope index of DALYs from neonatal diseases attributed to HAP increasing from −1701.47 (95% CI: −1799.28, −1603.67) in 1990 to −904.67 (95% CI: −1005.40, −803.93) in 2021, while the concentration index decreased from −0.49 (95% CI: −0.53, −0.46) to −0.60 (95% CI: −0.65, −0.55) in 2021.

### 3.4 Frontier analysis

To identify the optimal control of disease burden under the corresponding SDI conditions each year, we conducted a frontier analysis (Figures 5D–F and Supplementary Table S4). In the frontier analysis, among high SDI countries, the five farthest from the border fitting line are marked in red; among low SDI countries, the five closest to the line are marked in blue; and among all countries, the 15 farthest from the line are marked in black. For DALYs of neonatal diseases attributable to PMP, the countries farthest from the borderline are Mali, South Sudan, the Central African Republic, Sierra Leone, Nigeria. For DALYs of neonatal diseases attributable to HAP, the countries farthest from the borderline are Mali, South Sudan, the Central African Republic, Sierra Leone. For DALYs of neonatal diseases attributable to APMP, the countries farthest from the borderline are Pakistan, Nigeria, Equatorial Guinea, Botswana, and Suriname. Notably, the frontier fitted line for DALYs of neonatal diseases attributed to APMP is flatter than those for PMP and HAP, suggesting that its results are less significant compared to those for HAP and PMP.

## 4 Discussion

This study comprehensively evaluated the epidemiological trends of neonatal disease burden attributed to PMP (including HAP and APMP) from 1990 to 2021, taking into account multiple factors such as age, gender, region, and SDI. Joinpoint regression analysis revealed a significant downward trend in DALYs of neonatal diseases attributed to HAP from 1990 to 2021. Over the past three decades, as the global economy developed, many countries transitioned from using solid fuels (e.g., firewood and coal) to cleaner gas or liquid fuels, thereby reducing HAP pollution (27, 28). However, DALYs of neonatal diseases attributed to APMP exhibited a slight downward trend. Following the implementation of the United Nations Framework Convention on Climate Change and the Kyoto Protocol to limit greenhouse gas emissions in 2005, DALYs of neonatal diseases attributed to APMP decreased significantly from 2001 to 2010. However, further efforts are needed to continue mitigating global climate change. DALYs of neonatal diseases attributed to APMP increased significantly from 2010 to 2014. However, the implementation of the Paris Agreement in 2016 led to a reduction in global greenhouse gas emissions, and DALYs of neonatal diseases attributed to APMP decreased significantly from 2015 to 2021. Therefore, there is an urgent need for global cooperation to formula environmental conventions to reduce outdoor pollutant emissions, improve air quality, and protect human health.

From 1990 to 2021, the DALYs of neonatal diseases attributable to PMP, HAP, and APMP decreased across all age groups worldwide, with the most significant decline observed in the late neonatal period. The immune system of early neonates are immature and lack sufficient pathogen exposure and activation, increasing their sensitivity to environmental PMP (29). Moreover, the lung structure and function of early neonates differ from those of adults, and the immaturity of the lung immune system increases their sensitivity to pollutants (30). Fine particles such as PM_2.5_ can penetrate the lungs, causing oxidative stress and inflammatory responses that activate inflammatory cells to produce tumor necrosis factor *α* (TNF-α) and interleukin 1 (IL-1) (30). These inflammatory factors may increase the health burden of newborns. Environmental particulate matter may also affect the expression of liver metabolic enzymes and transporter genes, as well as the responses of immune cells such as T lymphocytes and macrophages (31, 32), thereby increasing the burden of neonatal disease. Additionally, we observed gender differences in DALYs of neonatal diseases attributed to PMP, particularly in the early neonatal period. DALYs of neonatal diseases attributed to PMP were consistently higher in males than in females, possibly due to male physiological characteristics and increased sensitivity to pollutants. Specifically, fine particles such as PM_2.5_ can penetrate deeper into the respiratory tract and enter the bloodstream, posing a serious threat to the respiratory, circulatory, central nervous, and reproductive systems (33). Male fetuses grow faster and have a higher oxygen demand, making them more susceptible to pollutants such as carbon monoxide (34). Therefore, future preventive interventions should consider gender and age difference.

The level of PMP pollution is closely related to the economic environment of a city. The spatiotemporal distribution of different SDIs has varying impacts on the burden of neonatal diseases. Among the five SDI subdivisions, the burden of neonatal diseases attributed to APMP and HAP increases as the SDI index decreases. In low and low-middle SDI countries, progress in reducing the burden of neonatal diseases attributed to PMP has been slow. Between 1990 and 2021, DALYs of neonatal diseases attributed to APMP increased in South Asia, a low-middle SDI region. As a low-middle SDI region, South Asia may have limited infrastructure and medical resources, making newborns more vulnerable to particulate pollution exposure. In low SDI regions, due to scarce medical resources and weak air pollution monitoring and control capabilities, pregnant women and newborns are more likely to be exposed to high concentrations of PM_2.5_ (34). Moreover, the poor quality of housing and medical facilities in low SDI regions may not provide adequate support to address health problems attributed to air pollution, making newborns more vulnerable to diseases (34, 35). Furthermore, public health systems in low SDI regions may not effectively report or record the burden of diseases associated with air pollution, making it difficult for policymakers to accurately assess and respond to the problem (34). From 1990 to 2021, Central and Eastern Europe had the largest decline in DALYs due to neonatal diseases attributed to PMP. The EU is an active promoter of global energy reform, and an important advocate for environmental protection and climate change. Since 1979, the EU has adopted several protocols to reduce air pollution, including the Convention on Long-range Transboundary Air Pollution and the Gothenburg Protocol. Therefore, low SDI regions still have a long way to go in reducing the burden of neonatal diseases. More attention and policy support are needed to improve air quality and increase awareness and response to air pollution-related health issues.

Additionally, the trend of health inequality in DALYs of neonatal diseases attributed to PMP has been increasing over the past three decades, highlighting the persistent gap between the rich and the poor. One of the key factors contributing to health inequality is the socioeconomic status (34). Therefore, this study offers a reference for PMP governance in 204 major countries and regions worldwide through frontier analysis. For countries with higher SDI, those with a large gap in DALYs from frontier neonatal diseases should increasing health economic investment in PMP pollution. The burden of neonatal diseases attributed to PMP remains significant in these economically affluent countries, necessitating additional policy and economic support. For countries with lower SDI, those with a small gap in DALYs from frontier neonatal diseases should reduce health economic investment in PMP pollution. These neonatal diseases are well controlled at their current SDI levels, allowing for the reallocation of health economic resources to other areas. For all countries with the largest gap in DALYs from frontier neonatal diseases, regardless of their SDI level, priority should be given to strengthening PMP management. Each country and region can use the frontier analysis results to develop PMP health policies tailored to their SDI level.

This study has several limitations. First, the GBD database depends on health data reported by countries and regions. In areas with insufficient data, the results are primarily based statistical modeling methods used by the GBD 2021 team. Although optimizing data processing and models can gradually improve prediction accuracy, this relies on the collection of higher-quality data. Secondly, inadequate medical system in some resource-poor areas may result in inaccurate diagnoses and underreporting of diseases, potentially underestimating the disease burden. Third, although this study covers the burden of early and late neonatal diseases, it lacks a detailed stratified analysis of this age group. Finally, while current studies suggest that PMP may impact newborn health via mechanisms like inflammatory response and oxidative stress (36), more large-scale population studies are needed to further elucidate the causal relationship between PMP and newborn health.

In summary, this study used the latest GBD 2021 database to update the global trends and burden of neonatal diseases attributed to PMP from 1990 to 2021. Over the past 30 years, the global burden of neonatal diseases attributed to PMP has declined, with males experiencing a higher burden than females. The early neonatal period is more susceptible to PMP than the late neonatal period. The burden of neonatal diseases attributed to PMP is highest in low SDI areas. Frontier analysis helps identify countries that most urgently need to address local PMP pollution problems. The current research results indicate that PMP, especially APMP, remains a significant factor in the burden of neonatal diseases. This study provides decision-makers and practitioners with a precise and targeted scientific basis for more effectively preventing and alleviating neonatal diseases attributed to PMP.

## Data Availability

The datasets presented in this study can be found in online repositories. The names of the repository/repositories and accession number(s) can be found in the article/Supplementary material.

## Footnotes

^1^ http://ghdx.healthdata.org/gbd-results-tool

^2^https://www.health data.org/research-analysis/about-gbd

## Supplementary material

SUPPLEMENTARY FIGURE S1 ASDRs of neonatal diseases attributable to PMP, HAP, and APMP in 1990 and 2021, and the corresponding AAPCs for 21 regions from 1990 to 2021. ASDRs of neonatal diseases attributable to **(A)** PMP, **(B)** HAP and **(C)** APMP. ASDR, age-standardized DALYs (disability-adjusted life years) rate; PMP, particulate matter pollution; HAP, household air pollution; APMP, ambient particulate matter pollution; AAPC, average annual percentage change.

SUPPLEMENTARY FIGURE S2 Gender trends in different regions from 1990 to 2021 based on the joinpoint regression model, with each line series representing global, high SDI, high-middle SDI, middle SDI, low-middle SDI, and low SDI. **(A)** Neonatal diseases DALYs attributable to PMP, **(B)** neonatal diseases DALYs attributable to HAP, **(C)** neonatal diseases DALYs attributable to APMP. SDI, socio-demographic index; DALYs, disability-adjusted life years; PMP, particulate matter pollution; HAP, household air pollution; APMP, ambient particulate matter pollution.
