# Supplementary figures and images for: Global, regional, and national burden of neonatal diseases attributable to particulate matter pollution from 1990 to 2021
Source: Front Public Health. 2025 Jun 9;13:1556340. doi: 10.3389/fpubh.2025.1556340 (PMC12183240; doi:10.3389/fpubh.2025.1556340)

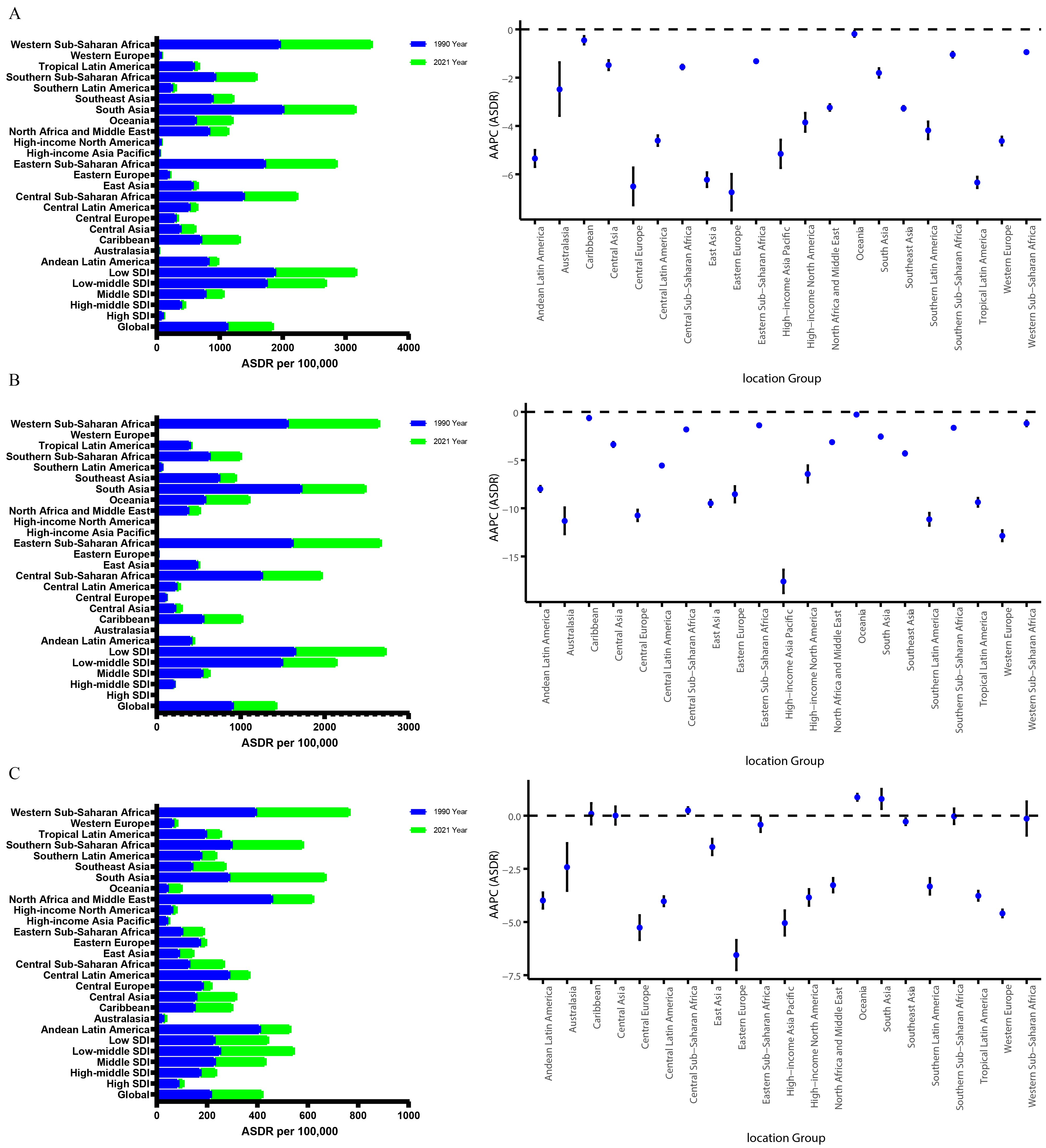

Supplement: SUPPLEMENTARY FIGURE S1 — ASDRs of neonatal diseases attributable to PMP, HAP, and APMP in 1990 and 2021, and the corresponding AAPCs for 21 regions from 1990 to 2021. ASDRs of neonatal diseases attributable to (A) PMP, (B) HAP and (C) APMP. ASDR, age-standardized DALYs (disability-adjusted life years) rate; PMP, particulate matter pollution; HAP, household air pollution; APMP, ambient particulate matter pollution; AAPC, average annual percentage change. [file Image_1.JPEG]

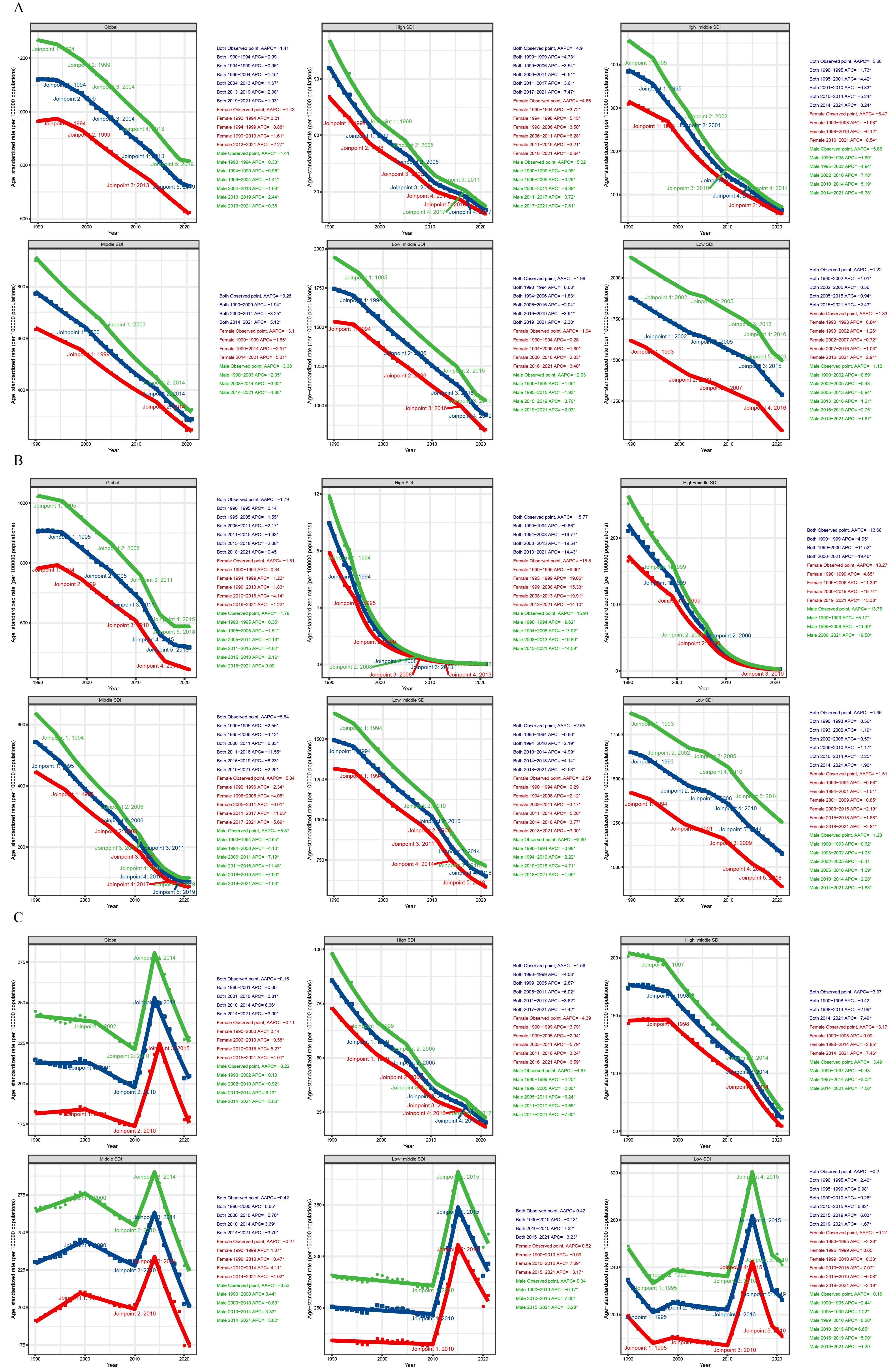

Supplement: SUPPLEMENTARY FIGURE S2 — Gender trends in different regions from 1990 to 2021 based on the joinpoint regression model, with each line series representing global, high SDI, high-middle SDI, middle SDI, low-middle SDI, and low SDI. (A) Neonatal diseases DALYs attributable to PMP, (B) neonatal diseases DALYs attributable to HAP, (C) neonatal diseases DALYs attributable to APMP. SDI, socio-demographic index; DALYs, disability-adjusted life years; PMP, particulate matter pollution; HAP, household air pollution; APMP, ambient particulate matter pollution. [file Image_2.JPEG]
