# Supplementary material for: Global, regional, and national burden of neonatal diseases attributable to particulate matter pollution from 1990 to 2021
Source: Front Public Health. 2025 Jun 9;13:1556340. doi: 10.3389/fpubh.2025.1556340 (PMC12183240; doi:10.3389/fpubh.2025.1556340)
Supplement: Supplementary file 3 [file Table_1.DOCX]

| Table S1 Global and regional age-standardized DALYs of neonatal diseases attributable to particulate matter pollution, household air pollution and ambient particulate matter pollution in 1990 and 2021, and AAPC of DALYs from 1990 to 2021. |
| --- |

| Location | Particulate matter pollution | | |  | Ambient particulate matter pollution | | | | Household air pollution from solid fuels | | | |
| --- | --- | --- | --- | --- | --- | --- | --- | --- | --- | --- | --- | --- |
|  | DALYs (per 100000 population), 1990 | DALYs (per 100000 population), 2021 | AAPC,1990-2021 | *P* value | DALYs (per 100000 population), 1990 | DALYs (per 100000 population), 2021 | AAPC,1990-2021 | *P* value | DALYs (per 100000 population), 1990 | DALYs (per 100000 population), 2021 | AAPC,1990-2021 | *P* value |
| Global | 1120.4(1025.3,1213.2) | 723.1(610.4,845.2) | -1.4(-1.5,-1.3) | 0.0 | 214.8(142.8,296.9) | 204.8(121.3,311.2) | -0.2(-0.4,0.1) | 0.1 | 905.4(801.2,1007.5) | 518.1(410.1,641.7) | -1.8(-1.9,-1.7) | 0.0 |
| SDI |  |  |  |  |  |  |  |  |  |  |  |  |
| High SDI | 95.7(80.2,111.0) | 20.3(16.6,23.9) | -4.9(-5.2,-4.6) | 0.0 | 85.7(69.9,102.0) | 20.2(16.4,23.9) | -4.6(-4.8,-4.3) | 0.0 | 9.9(3.8,21.7) | 0.1(0.0,0.4) | -15.8(-16.5,-15.0) | 0.0 |
| High-middle SDI | 384.3(332.3,444.6) | 64.8(52.9,76.6) | -5.7(-6.0,-5.3) | 0.0 | 173.7(108.7,232.5) | 62.1(49.2,75.2) | -3.4(-3.6,-3.2) | 0.0 | 210.6(151.3,282.7) | 2.7(0.2,14.5) | -13.7(-14.2,-13.2) | 0.0 |
| Middle SDI | 773.5(703.7,852.3) | 287.6(241.3,339.9) | -3.3(-3.4,-3.1) | 0.0 | 230.4(147.7,310.9) | 201.3(127.7,272.8) | -0.4(-0.7,-0.2) | 0.0 | 542.9(451.8,643.1) | 86.2(39.5,162.8) | -5.8(-5.9,-5.7) | 0.0 |
| Low-middle SDI | 1744.9(1554.7,1941.7) | 941.8(775.1,1140.5) | -2.0(-2.1,-1.8) | 0.0 | 251.3(160.1,380.6) | 293.0(153.1,467.5) | 0.4(0.2,0.7) | 0.0 | 1493.4(1299.7,1677.8) | 648.7(471.8,838.4) | -2.6(-2.8,-2.5) | 0.0 |
| Low SDI | 1879.1(1719.0,2046.9) | 1290.0(1064.3,1529.2) | -1.2(-1.3,-1.2) | 0.0 | 229.5(157.0,327.4) | 212.5(129.0,341.4) | -0.2(-0.4,0.0) | 0.0 | 1649.5(1493.4,1807.8) | 1077.3(874.2,1307.6) | -1.4(-1.4,-1.3) | 0.0 |
| **Region** |  |  |  |  |  |  |  |  |  |  |  |  |
| Andean Latin America | 822.0(638.1,1022.7) | 153.5(97.7,230.8) | -5.3(-5.7,-5.0) | 0.0 | 409.8(190.8,689.6) | 120.4(60.9,189.0) | -4.0(-4.4,-3.6) | 0.0 | 411.8(200.6,647.0) | 33.0(7.0,90.0) | -8.0(-8.3,-7.7) | 0.0 |
| Australasia | 26.3(3.6,61.7) | 11.9(1.7,28.3) | -2.5(-3.6,-1.4) | 0.0 | 25.8(3.6,60.5) | 11.9(1.7,28.3) | -2.4(-3.5,-1.3) | 0.0 | 0.5(0.0,4.4) | 0.0(0.0,0.1) | -11.3(-12.7,-9.9) | 0.0 |
| Caribbean | 705.1(545.6,898.4) | 613.4(423.2,816.5) | -0.5(-0.6,-0.3) | 0.0 | 150.0(64.6,276.7) | 150.2(80.3,235.1) | 0.1(-0.4,0.6) | 0.7 | 554.9(380.4,747.3) | 463.2(301.7,630.8) | -0.6(-0.8,-0.4) | 0.0 |
| Central Asia | 376.7(298.7,463.3) | 236.8(172.8,320.2) | -1.5(-1.7,-1.3) | 0.0 | 156.8(67.5,264.5) | 158.4(98.9,230.7) | 0.0(-0.4,0.4) | 1.0 | 219.8(122.9,321.8) | 78.3(38.5,142.5) | -3.4(-3.6,-3.1) | 0.0 |
| Central Europe | 297.3(255.9,339.9) | 38.2(29.1,47.8) | -6.5(-7.3,-5.7) | 0.0 | 182.7(78.8,258.4) | 34.7(20.4,46.0) | -5.3(-5.8,-4.7) | 0.0 | 114.4(48.3,213.5) | 3.5(0.2,16.2) | -10.7(-11.3,-10.2) | 0.0 |
| Central Latin America | 522.0(443.7,609.8) | 121.7(88.8,159.9) | -4.6(-4.8,-4.4) | 0.0 | 287.6(153.5,411.2) | 80.5(48.0,114.5) | -4.0(-4.2,-3.8) | 0.0 | 234.2(127.5,379.6) | 41.1(19.9,72.3) | -5.6(-5.7,-5.4) | 0.0 |
| Central Sub-Saharan Africa | 1382.5(1077.7,1779.5) | 850.4(600.5,1143.0) | -1.6(-1.6,-1.5) | 0.0 | 128.9(71.2,212.9) | 137.6(66.1,224.8) | 0.2(0.1,0.4) | 0.0 | 1253.6(969.9,1613.4) | 712.7(468.0,984.3) | -1.8(-1.9,-1.7) | 0.0 |
| East Asia | 570.1(477.8,665.5) | 80.4(64.5,99.2) | -6.2(-6.5,-5.9) | 0.0 | 88.2(43.6,152.6) | 57.1(31.9,79.8) | -1.5(-1.8,-1.1) | 0.0 | 481.8(385.4,579.8) | 23.2(9.2,49.8) | -9.5(-9.8,-9.1) | 0.0 |
| Eastern Europe | 191.1(156.5,226.0) | 25.4(18.7,33.0) | -6.7(-7.5,-6.0) | 0.0 | 171.3(128.3,209.3) | 24.0(17.0,32.0) | -6.6(-7.3,-5.9) | 0.0 | 19.6(6.1,56.4) | 1.4(0.2,4.9) | -8.6(-9.4,-7.7) | 0.0 |
| Eastern Sub-Saharan Africa | 1718.8(1521.0,1910.0) | 1138.4(893.7,1405.4) | -1.3(-1.4,-1.3) | 0.0 | 100.9(69.1,143.6) | 86.6(53.1,137.9) | -0.4(-0.8,-0.1) | 0.0 | 1617.9(1426.1,1804.1) | 1051.8(818.7,1298.5) | -1.4(-1.4,-1.4) | 0.0 |
| High-income Asia Pacific | 42.6(24.7,65.5) | 8.2(5.6,11.5) | -5.2(-5.7,-4.6) | 0.0 | 41.1(23.2,64.0) | 8.2(5.6,11.5) | -5.0(-5.6,-4.5) | 0.0 | 1.5(0.1,7.0) | 0.0(0.0,0.0) | -17.6(-18.8,-16.4) | 0.0 |
| High-income North America | 61.4(48.4,74.1) | 17.2(11.9,22.9) | -3.8(-4.2,-3.5) | 0.0 | 61.3(48.1,74.1) | 17.2(11.9,22.9) | -3.8(-4.2,-3.5) | 0.0 | 0.1(0.0,0.5) | 0.0(0.0,0.0) | -6.4(-7.3,-5.6) | 0.0 |
| North Africa and Middle East | 832.8(703.8,983.4) | 303.1(239.4,377.9) | -3.2(-3.4,-3.1) | 0.0 | 458.1(320.5,585.6) | 163.1(125.8,212.2) | -3.3(-3.6,-2.9) | 0.0 | 374.5(261.9,523.9) | 139.9(99.8,194.5) | -3.1(-3.3,-3.0) | 0.0 |
| Oceania | 621.0(457.4,817.5) | 581.7(382.4,792.6) | -0.2(-0.3,-0.1) | 0.0 | 43.4(12.9,107.8) | 55.5(16.3,130.7) | 0.9(0.7,1.0) | 0.0 | 577.5(408.2,760.4) | 526.1(344.6,743.5) | -0.3(-0.4,-0.2) | 0.0 |
| South Asia | 2010.4(1783.9,2237.8) | 1148.1(937.4,1398.7) | -1.8(-2.0,-1.6) | 0.0 | 287.8(164.6,470.5) | 382.7(212.6,591.0) | 0.8(0.3,1.3) | 0.0 | 1722.5(1474.7,1961.5) | 765.2(559.9,1002.9) | -2.6(-2.8,-2.4) | 0.0 |
| Southeast Asia | 886.4(784.1,999.8) | 329.6(264.3,400.8) | -3.3(-3.4,-3.2) | 0.0 | 141.2(69.5,245.9) | 131.7(58.7,210.4) | -0.3(-0.4,-0.1) | 0.0 | 745.0(610.1,885.9) | 197.7(126.9,282.5) | -4.3(-4.5,-4.1) | 0.0 |
| Southern Latin America | 242.1(134.6,365.0) | 61.0(25.7,98.6) | -4.2(-4.5,-3.8) | 0.0 | 176.6(68.6,304.5) | 59.3(25.4,96.6) | -3.3(-3.7,-2.9) | 0.0 | 65.2(17.6,160.0) | 1.6(0.0,13.8) | -11.1(-11.8,-10.5) | 0.0 |
| Southern Sub-Saharan Africa | 928.0(759.8,1106.7) | 657.5(501.4,860.0) | -1.0(-1.2,-0.9) | 0.0 | 297.4(156.2,480.5) | 283.2(172.4,406.5) | 0.0(-0.4,0.3) | 0.9 | 630.3(438.6,832.3) | 374.1(245.7,534.1) | -1.7(-1.7,-1.6) | 0.0 |
| Tropical Latin America | 590.4(496.3,681.0) | 79.4(56.6,104.2) | -6.3(-6.6,-6.1) | 0.0 | 195.3(85.6,316.1) | 59.8(33.5,86.1) | -3.8(-4.0,-3.5) | 0.0 | 394.9(266.5,514.0) | 19.5(6.8,39.2) | -9.4(-9.8,-8.9) | 0.0 |
| Western Europe | 66.3(55.2,79.2) | 15.6(10.9,21.4) | -4.6(-4.8,-4.4) | 0.0 | 65.7(54.6,78.7) | 15.6(10.9,21.2) | -4.6(-4.8,-4.4) | 0.0 | 0.6(0.0,4.1) | 0.0(0.0,0.1) | -12.9(-13.4,-12.3) | 0.0 |
| Western Sub-Saharan Africa | 1955.0(1771.7,2149.4) | 1460.5(1246.2,1694.1) | -0.9(-1.0,-0.9) | 0.0 | 394.1(233.3,586.5) | 371.2(190.5,621.4) | -0.1(-0.9,0.7) | 0.7 | 1560.5(1317.8,1789.5) | 1089.0(825.8,1359.8) | -1.2(-1.5,-0.9) | 0.0 |
| **Country** |  |  |  |  |  |  |  |  |  |  |  |  |
| Afghanistan | 1465.8(951.5,2120.5) | 751.5(461.9,1076.9) | -2.2(-2.4,-2.0) | 0.0 | 186.1(80.1,382.1) | 94.4(50.2,155.3) | -2.4(-3.3,-1.6) | 0.0 | 1279.6(811.3,1906.9) | 657.0(396.7,945.8) | -2.1(-2.3,-2.0) | 0.0 |
| Albania | 478.5(291.4,693.6) | 130.4(46.5,231.8) | -4.1(-4.3,-3.9) | 0.0 | 84.6(30.0,179.9) | 95.2(24.2,190.0) | 0.4(0.1,0.6) | 0.0 | 393.7(225.7,582.7) | 35.1(3.0,112.1) | -7.5(-7.7,-7.4) | 0.0 |
| Algeria | 430.9(197.8,707.5) | 191.3(81.9,324.5) | -2.5(-2.7,-2.3) | 0.0 | 394.3(175.1,659.7) | 190.9(81.9,323.9) | -2.2(-2.5,-2.0) | 0.0 | 36.5(2.9,160.0) | 0.4(0.0,2.7) | -13.3(-13.6,-13.1) | 0.0 |
| American Samoa | 46.6(3.5,105.7) | 28.6(3.8,65.2) | -1.4(-1.7,-1.0) | 0.0 | 39.8(2.7,100.5) | 23.1(2.4,55.9) | -1.5(-1.9,-1.2) | 0.0 | 6.6(0.0,41.5) | 5.5(0.1,25.2) | -0.5(-1.1,0.1) | 0.1 |
| Andorra | 37.9(6.4,75.3) | 2.6(0.0,6.9) | -8.1(-9.7,-6.5) | 0.0 | 37.9(6.4,74.8) | 2.6(0.0,6.9) | -8.1(-9.7,-6.5) | 0.0 | 0.0(0.0,0.0) | 0.0(0.0,0.0) | -12.0(-17.1,-6.5) | 0.0 |
| Angola | 1875.4(1245.8,2565.6) | 642.7(355.0,976.9) | -3.5(-3.6,-3.3) | 0.0 | 225.6(100.0,412.6) | 294.2(103.0,573.1) | 0.9(0.8,1.1) | 0.0 | 1649.8(1094.1,2302.3) | 348.0(126.7,650.2) | -5.0(-5.3,-4.7) | 0.0 |
| Antigua and Barbuda | 134.6(44.4,252.8) | 82.9(28.8,141.5) | -1.4(-1.8,-1.1) | 0.0 | 127.5(43.7,246.5) | 82.5(29.0,141.1) | -1.3(-1.6,-0.9) | 0.0 | 7.0(0.2,40.0) | 0.4(0.0,3.8) | -8.8(-9.5,-8.0) | 0.0 |
| Argentina | 269.4(106.4,458.1) | 64.0(18.2,117.7) | -4.5(-4.9,-4.0) | 0.0 | 225.4(77.5,402.6) | 62.6(17.5,116.4) | -4.0(-4.4,-3.5) | 0.0 | 43.8(3.5,165.5) | 1.4(0.0,11.0) | -10.5(-11.2,-9.7) | 0.0 |
| Armenia | 456.1(226.1,738.2) | 131.6(68.2,205.9) | -3.9(-4.5,-3.3) | 0.0 | 254.1(95.4,473.2) | 124.9(66.0,195.7) | -2.2(-3.0,-1.3) | 0.0 | 201.9(71.7,402.3) | 6.7(0.6,24.9) | -10.5(-11.2,-9.8) | 0.0 |
| Australia | 26.7(0.5,68.1) | 11.4(0.5,30.3) | -2.7(-3.5,-1.8) | 0.0 | 26.2(0.5,66.3) | 11.4(0.5,29.8) | -2.6(-3.5,-1.7) | 0.0 | 0.5(0.0,4.5) | 0.0(0.0,0.0) | -11.9(-13.7,-10.0) | 0.0 |
| Austria | 71.5(26.3,121.8) | 14.9(1.1,33.6) | -5.0(-5.7,-4.3) | 0.0 | 71.4(26.2,121.7) | 14.9(1.1,33.6) | -5.0(-5.7,-4.3) | 0.0 | 0.1(0.0,1.3) | 0.0(0.0,0.0) | -9.9(-11.6,-8.1) | 0.0 |
| Azerbaijan | 483.8(244.3,825.9) | 279.4(106.5,494.8) | -1.8(-2.0,-1.5) | 0.0 | 233.2(59.8,573.2) | 265.5(100.8,477.8) | 0.4(0.1,0.7) | 0.0 | 250.2(72.7,550.0) | 13.7(0.2,83.4) | -9.1(-9.6,-8.5) | 0.0 |
| Bahamas | 208.5(77.9,388.3) | 80.3(29.3,148.2) | -3.0(-3.2,-2.7) | 0.0 | 206.2(76.4,384.6) | 80.0(28.9,147.5) | -2.9(-3.2,-2.7) | 0.0 | 2.3(0.0,23.1) | 0.3(0.0,2.6) | -6.7(-7.1,-6.2) | 0.0 |
| Bahrain | 410.6(271.2,588.3) | 70.2(44.1,103.3) | -5.5(-6.2,-4.9) | 0.0 | 408.4(258.1,586.3) | 70.2(44.1,103.3) | -5.5(-6.1,-4.9) | 0.0 | 2.1(0.0,19.0) | 0.0(0.0,0.1) | -14.5(-15.3,-13.8) | 0.0 |
| Bangladesh | 2528.9(1800.0,3312.4) | 979.7(619.2,1421.6) | -3.1(-3.2,-2.9) | 0.0 | 216.4(102.5,412.6) | 143.7(66.6,276.3) | -1.4(-2.0,-0.8) | 0.0 | 2312.5(1625.0,3060.3) | 835.8(522.2,1258.2) | -3.3(-3.6,-3.0) | 0.0 |
| Barbados | 250.8(96.6,433.5) | 161.2(59.9,301.7) | -1.0(-1.3,-0.7) | 0.0 | 249.9(96.5,433.2) | 161.0(59.8,301.3) | -1.0(-1.3,-0.7) | 0.0 | 0.8(0.0,4.9) | 0.1(0.0,1.0) | -5.3(-6.0,-4.6) | 0.0 |
| Belarus | 181.9(83.7,309.6) | 18.5(3.7,35.9) | -7.4(-8.6,-6.2) | 0.0 | 173.5(79.9,298.8) | 18.3(3.7,35.8) | -7.3(-8.5,-6.0) | 0.0 | 8.3(0.5,40.1) | 0.1(0.0,1.1) | -13.1(-13.8,-12.3) | 0.0 |
| Belgium | 64.1(16.5,117.6) | 15.3(1.7,34.9) | -4.5(-5.5,-3.4) | 0.0 | 63.9(16.5,117.8) | 15.3(1.7,34.9) | -4.4(-5.5,-3.4) | 0.0 | 0.1(0.0,1.1) | 0.0(0.0,0.0) | -11.0(-11.6,-10.4) | 0.0 |
| Belize | 586.6(277.2,934.4) | 216.6(101.9,351.8) | -2.9(-3.5,-2.3) | 0.0 | 258.0(81.3,497.2) | 164.4(67.2,291.3) | -1.2(-1.8,-0.7) | 0.0 | 328.5(102.8,646.1) | 52.1(7.6,138.6) | -5.7(-6.1,-5.3) | 0.0 |
| Benin | 2014.0(1367.3,2756.7) | 1476.7(922.6,2071.3) | -1.0(-1.0,-1.0) | 0.0 | 303.6(159.2,504.5) | 220.8(109.6,379.5) | -1.1(-2.0,-0.1) | 0.0 | 1710.3(1148.8,2310.4) | 1255.9(765.7,1803.9) | -1.0(-1.2,-0.8) | 0.0 |
| Bermuda | 43.5(3.6,93.7) | 8.4(0.0,24.1) | -5.1(-5.6,-4.6) | 0.0 | 42.7(3.3,92.0) | 8.3(0.0,24.1) | -5.0(-5.5,-4.6) | 0.0 | 0.7(0.0,8.2) | 0.0(0.0,0.1) | -12.1(-13.3,-11.0) | 0.0 |
| Bhutan | 2238.3(1525.9,2998.4) | 511.2(269.4,770.7) | -5.0(-5.6,-4.4) | 0.0 | 251.8(116.5,469.9) | 370.6(153.8,596.4) | 1.0(0.1,1.9) | 0.0 | 1986.2(1353.3,2655.4) | 140.1(27.4,361.9) | -8.2(-8.4,-8.0) | 0.0 |
| Bolivia (Plurinational State of) | 980.7(655.5,1353.3) | 239.1(125.3,395.5) | -4.5(-4.7,-4.4) | 0.0 | 393.5(171.3,694.0) | 144.7(61.6,261.1) | -3.2(-3.5,-3.0) | 0.0 | 586.9(314.1,876.4) | 94.2(25.5,198.9) | -5.8(-5.9,-5.6) | 0.0 |
| Bosnia and Herzegovina | 587.9(369.9,832.9) | 103.9(45.5,165.1) | -5.4(-6.1,-4.6) | 0.0 | 77.1(37.1,139.0) | 75.8(20.7,134.5) | 0.0(-1.4,1.3) | 1.0 | 510.8(312.9,729.4) | 28.1(3.5,80.7) | -8.9(-9.7,-8.1) | 0.0 |
| Botswana | 1262.4(847.8,1745.2) | 515.7(256.0,870.3) | -2.9(-3.0,-2.7) | 0.0 | 277.1(88.3,580.2) | 419.6(117.8,794.0) | 1.3(1.0,1.6) | 0.0 | 985.0(606.0,1417.4) | 95.5(2.7,410.0) | -7.3(-7.4,-7.2) | 0.0 |
| Brazil | 591.1(494.3,684.1) | 77.5(55.3,103.6) | -6.4(-6.6,-6.2) | 0.0 | 199.1(88.2,320.5) | 59.6(34.1,85.0) | -3.8(-4.1,-3.6) | 0.0 | 391.7(264.3,515.9) | 17.8(6.5,35.8) | -9.6(-10.0,-9.2) | 0.0 |
| Brunei Darussalam | 27.3(2.1,67.1) | 27.3(2.6,63.2) | -0.3(-0.9,0.4) | 0.5 | 27.2(2.0,67.1) | 27.3(2.6,63.1) | -0.2(-0.9,0.4) | 0.5 | 0.1(0.0,0.3) | 0.0(0.0,0.1) | -3.0(-6.3,0.4) | 0.1 |
| Bulgaria | 144.2(70.5,225.0) | 42.2(14.3,77.6) | -4.4(-4.9,-3.8) | 0.0 | 114.6(38.5,195.3) | 39.2(10.0,74.9) | -3.7(-4.7,-2.7) | 0.0 | 29.5(2.9,97.0) | 3.0(0.0,20.0) | -7.5(-8.5,-6.5) | 0.0 |
| Burkina Faso | 1583.7(1100.8,2092.7) | 1215.1(826.6,1666.9) | -0.9(-0.9,-0.8) | 0.0 | 200.2(107.0,324.6) | 169.9(91.3,288.6) | -0.4(-1.5,0.8) | 0.5 | 1383.4(933.2,1863.5) | 1045.0(695.9,1446.2) | -0.9(-1.0,-0.8) | 0.0 |
| Burundi | 1644.7(1158.2,2233.8) | 1104.6(703.8,1615.4) | -1.3(-1.4,-1.1) | 0.0 | 112.5(49.9,214.2) | 62.6(30.0,112.9) | -1.9(-2.1,-1.6) | 0.0 | 1532.2(1076.6,2068.1) | 1041.9(649.5,1531.1) | -1.3(-1.4,-1.1) | 0.0 |
| Cabo Verde | 758.4(459.3,1080.0) | 309.0(169.9,495.3) | -2.9(-3.2,-2.5) | 0.0 | 124.1(59.8,202.4) | 215.1(93.2,365.7) | 2.0(1.4,2.6) | 0.0 | 634.3(374.2,903.6) | 93.8(25.9,209.5) | -6.1(-6.4,-5.8) | 0.0 |
| Cambodia | 1281.8(867.1,1763.7) | 607.4(374.4,907.1) | -2.4(-2.5,-2.3) | 0.0 | 96.7(36.1,204.7) | 81.4(31.6,162.5) | -0.3(-0.7,0.1) | 0.2 | 1185.0(796.5,1632.8) | 525.8(324.5,778.7) | -2.6(-2.7,-2.4) | 0.0 |
| Cameroon | 1469.9(992.6,2000.2) | 961.0(589.9,1375.9) | -1.4(-1.4,-1.3) | 0.0 | 279.2(122.6,490.9) | 194.0(88.0,365.9) | -1.2(-1.5,-0.9) | 0.0 | 1190.4(803.2,1674.1) | 766.9(436.0,1123.0) | -1.4(-1.5,-1.4) | 0.0 |
| Canada | 31.2(2.4,70.8) | 12.9(0.0,35.9) | -2.5(-3.3,-1.7) | 0.0 | 31.0(2.4,70.8) | 12.9(0.0,35.9) | -2.5(-3.2,-1.7) | 0.0 | 0.1(0.0,1.3) | 0.0(0.0,0.0) | -9.8(-11.9,-7.7) | 0.0 |
| Central African Republic | 1981.1(1363.5,2667.3) | 1725.6(1104.7,2470.9) | -0.5(-0.6,-0.4) | 0.0 | 150.8(76.7,280.3) | 126.5(66.5,206.3) | -0.6(-0.9,-0.3) | 0.0 | 1830.1(1252.1,2476.9) | 1599.1(1014.5,2296.1) | -0.4(-0.5,-0.4) | 0.0 |
| Chad | 1763.2(1265.2,2313.7) | 1451.2(963.8,2064.4) | -0.6(-0.7,-0.6) | 0.0 | 221.4(118.3,356.9) | 219.6(111.1,399.5) | -0.2(-0.6,0.2) | 0.3 | 1541.6(1103.1,2034.6) | 1231.3(798.3,1783.1) | -0.7(-0.8,-0.6) | 0.0 |
| Chile | 196.1(100.0,300.6) | 59.3(22.7,100.1) | -3.8(-4.8,-2.7) | 0.0 | 76.8(21.2,170.6) | 56.8(19.9,97.2) | -1.0(-2.1,0.2) | 0.1 | 119.1(44.7,212.3) | 2.5(0.0,25.1) | -11.6(-12.6,-10.7) | 0.0 |
| China | 579.7(485.1,675.7) | 78.0(62.3,97.5) | -6.4(-6.7,-6.1) | 0.0 | 89.4(44.1,155.4) | 58.5(32.3,81.9) | -1.4(-1.8,-1.1) | 0.0 | 490.2(391.0,592.1) | 19.4(5.1,47.2) | -10.3(-10.7,-9.9) | 0.0 |
| Colombia | 491.6(251.0,764.9) | 67.1(23.5,128.6) | -6.3(-6.6,-5.9) | 0.0 | 264.1(81.5,526.3) | 59.6(18.5,115.2) | -4.7(-5.3,-4.2) | 0.0 | 227.2(68.1,446.6) | 7.5(0.3,31.9) | -10.5(-10.8,-10.1) | 0.0 |
| Comoros | 2168.1(1507.2,2986.7) | 1254.6(796.5,1828.7) | -1.7(-1.8,-1.7) | 0.0 | 93.7(42.5,170.8) | 59.6(26.1,116.5) | -1.1(-1.4,-0.9) | 0.0 | 2074.2(1454.4,2850.5) | 1194.9(755.0,1726.3) | -1.8(-1.8,-1.7) | 0.0 |
| Congo | 1067.8(681.8,1513.1) | 622.0(368.0,933.8) | -1.8(-1.9,-1.6) | 0.0 | 154.7(54.0,331.9) | 211.8(68.4,429.2) | 1.1(0.9,1.2) | 0.0 | 912.9(562.3,1324.1) | 409.9(201.2,698.0) | -2.6(-2.7,-2.4) | 0.0 |
| Cook Islands | 74.1(11.6,159.2) | 8.5(0.7,20.6) | -6.5(-7.0,-6.0) | 0.0 | 54.2(4.8,137.3) | 8.3(0.7,20.4) | -5.8(-6.1,-5.4) | 0.0 | 19.8(0.4,84.1) | 0.2(0.0,2.3) | -13.5(-14.5,-12.6) | 0.0 |
| Costa Rica | 222.6(108.8,363.0) | 51.3(7.1,102.8) | -4.4(-4.5,-4.3) | 0.0 | 121.6(37.4,240.3) | 47.8(6.1,100.1) | -2.7(-3.0,-2.3) | 0.0 | 100.9(28.1,211.8) | 3.5(0.1,20.0) | -10.3(-10.8,-9.7) | 0.0 |
| Coted'Ivoire | 1928.0(1343.6,2587.3) | 1405.0(900.6,1992.4) | -1.0(-1.1,-0.9) | 0.0 | 409.1(195.1,684.3) | 328.2(152.9,630.3) | -0.6(-1.2,0.0) | 0.0 | 1518.8(1041.3,2118.7) | 1076.5(680.5,1599.6) | -1.1(-1.3,-1.0) | 0.0 |
| Croatia | 143.0(50.2,231.3) | 27.0(5.3,55.1) | -5.2(-5.9,-4.6) | 0.0 | 127.2(38.9,216.5) | 26.5(4.9,54.8) | -5.1(-5.6,-4.6) | 0.0 | 15.7(0.3,85.2) | 0.5(0.0,4.9) | -10.6(-11.9,-9.3) | 0.0 |
| Cuba | 114.8(34.0,199.2) | 25.6(7.0,46.2) | -4.2(-4.8,-3.7) | 0.0 | 94.0(24.2,170.9) | 24.3(6.4,42.9) | -4.0(-4.6,-3.4) | 0.0 | 20.7(2.4,61.6) | 1.3(0.1,5.1) | -8.5(-9.9,-7.0) | 0.0 |
| Cyprus | 122.0(41.8,212.9) | 12.8(3.9,24.0) | -7.3(-7.5,-7.0) | 0.0 | 121.1(41.5,212.0) | 12.8(3.9,24.0) | -7.2(-7.5,-6.9) | 0.0 | 1.0(0.0,7.8) | 0.0(0.0,0.0) | -15.7(-16.0,-15.5) | 0.0 |
| Czechia | 136.4(56.1,216.7) | 12.5(2.2,26.1) | -7.6(-8.4,-6.8) | 0.0 | 134.8(55.7,213.5) | 12.4(2.2,25.6) | -7.5(-8.3,-6.7) | 0.0 | 1.6(0.0,15.1) | 0.0(0.0,0.3) | -11.2(-12.2,-10.3) | 0.0 |
| Democratic People's Republic of Korea | 484.5(311.7,694.3) | 196.8(116.4,291.9) | -2.9(-3.3,-2.4) | 0.0 | 71.9(27.2,153.0) | 19.7(10.3,33.1) | -4.1(-4.8,-3.5) | 0.0 | 412.5(264.3,602.7) | 177.1(105.3,261.3) | -2.7(-3.0,-2.4) | 0.0 |
| Democratic Republic of the Congo | 1232.1(853.4,1726.1) | 902.2(553.4,1311.4) | -1.0(-1.1,-0.8) | 0.0 | 90.7(45.9,169.5) | 60.7(31.4,102.0) | -1.2(-1.5,-1.0) | 0.0 | 1141.3(784.5,1607.8) | 841.5(515.6,1211.1) | -1.0(-1.1,-0.8) | 0.0 |
| Denmark | 47.5(11.0,88.4) | 15.6(0.3,38.8) | -3.7(-5.4,-2.0) | 0.0 | 47.4(10.9,89.1) | 15.6(0.3,38.8) | -3.7(-5.4,-2.0) | 0.0 | 0.0(0.0,0.3) | 0.0(0.0,0.0) | -8.1(-9.2,-7.1) | 0.0 |
| Djibouti | 1026.2(651.7,1490.1) | 545.4(308.7,899.3) | -2.0(-2.3,-1.8) | 0.0 | 314.0(131.2,563.9) | 302.9(136.9,550.0) | -0.2(-0.6,0.2) | 0.4 | 711.9(387.4,1102.8) | 242.2(95.3,457.4) | -3.4(-3.8,-3.1) | 0.0 |
| Dominica | 353.1(188.9,540.4) | 321.5(137.1,585.7) | -0.3(-0.7,0.1) | 0.2 | 124.8(25.9,294.7) | 278.6(101.4,527.9) | 2.6(2.0,3.1) | 0.0 | 228.0(86.4,408.2) | 42.5(2.9,154.3) | -5.4(-6.1,-4.7) | 0.0 |
| Dominican Republic | 949.8(572.1,1391.6) | 335.7(127.8,572.1) | -3.3(-3.5,-3.0) | 0.0 | 234.2(62.5,550.1) | 310.7(116.9,550.2) | 1.0(0.4,1.5) | 0.0 | 715.2(368.1,1134.6) | 24.8(0.6,150.1) | -10.5(-11.3,-9.7) | 0.0 |
| Ecuador | 528.2(300.9,793.2) | 90.0(28.8,166.8) | -5.5(-6.2,-4.7) | 0.0 | 335.3(144.0,578.2) | 80.6(25.3,150.0) | -4.6(-5.2,-3.9) | 0.0 | 192.6(54.2,407.4) | 9.3(0.6,38.8) | -9.4(-9.9,-8.8) | 0.0 |
| Egypt | 643.8(384.9,937.4) | 108.0(63.9,164.7) | -5.7(-6.7,-4.6) | 0.0 | 447.6(229.4,702.3) | 107.7(63.8,164.2) | -4.7(-5.9,-3.4) | 0.0 | 196.0(76.5,378.1) | 0.2(0.0,0.9) | -20.0(-20.7,-19.2) | 0.0 |
| El Salvador | 667.7(407.5,953.2) | 78.1(28.2,146.3) | -6.7(-7.1,-6.2) | 0.0 | 141.0(52.0,292.6) | 52.2(15.3,106.8) | -3.2(-4.1,-2.2) | 0.0 | 526.5(311.4,794.4) | 25.9(5.6,68.2) | -9.4(-9.8,-9.0) | 0.0 |
| Equatorial Guinea | 1461.2(969.1,2058.7) | 483.8(237.2,830.1) | -3.5(-4.0,-3.0) | 0.0 | 155.1(69.4,289.8) | 469.7(206.5,800.0) | 3.7(3.1,4.3) | 0.0 | 1305.9(848.8,1851.3) | 13.8(0.0,136.1) | -13.5(-14.9,-12.0) | 0.0 |
| Eritrea | 1250.4(812.7,1698.6) | 820.1(520.4,1182.9) | -1.4(-1.5,-1.3) | 0.0 | 129.7(67.3,228.6) | 110.9(51.3,209.7) | -0.7(-1.2,-0.2) | 0.0 | 1120.6(731.8,1553.1) | 709.1(439.5,1022.3) | -1.4(-1.6,-1.3) | 0.0 |
| Estonia | 105.4(31.3,184.2) | 4.2(0.2,10.6) | -10.2(-10.8,-9.6) | 0.0 | 76.6(15.3,145.9) | 4.0(0.2,10.3) | -9.2(-9.9,-8.5) | 0.0 | 28.8(2.9,92.5) | 0.1(0.0,1.2) | -15.9(-16.9,-14.8) | 0.0 |
| Eswatini | 980.5(598.7,1416.8) | 493.9(268.5,766.0) | -2.2(-2.5,-2.0) | 0.0 | 186.4(67.6,402.6) | 225.8(57.7,482.2) | 0.5(0.0,1.0) | 0.1 | 793.8(450.9,1194.0) | 267.8(77.2,530.5) | -3.4(-3.6,-3.3) | 0.0 |
| Ethiopia | 2376.8(1961.8,2846.2) | 1305.5(988.9,1739.0) | -1.9(-2.0,-1.8) | 0.0 | 118.6(81.1,173.7) | 94.0(55.2,146.9) | -0.8(-1.0,-0.5) | 0.0 | 2258.2(1859.3,2675.7) | 1211.5(915.6,1614.5) | -2.0(-2.1,-1.9) | 0.0 |
| Fiji | 331.1(188.0,490.5) | 167.2(60.6,301.3) | -2.2(-2.5,-1.9) | 0.0 | 44.2(9.1,122.4) | 86.7(18.2,193.0) | 2.3(1.4,3.1) | 0.0 | 286.7(148.2,448.1) | 80.4(14.1,195.2) | -4.0(-4.5,-3.5) | 0.0 |
| Finland | 23.8(0.8,58.8) | 5.1(0.0,15.5) | -4.9(-6.1,-3.6) | 0.0 | 23.7(0.8,58.7) | 5.1(0.0,15.6) | -4.9(-6.1,-3.6) | 0.0 | 0.1(0.0,0.7) | 0.0(0.0,0.0) | -10.2(-11.9,-8.5) | 0.0 |
| France | 37.6(8.8,71.7) | 15.0(1.1,38.0) | -2.8(-3.0,-2.6) | 0.0 | 37.5(8.5,71.7) | 15.0(1.1,38.0) | -2.8(-2.9,-2.6) | 0.0 | 0.1(0.0,0.9) | 0.0(0.0,0.0) | -8.9(-9.7,-8.1) | 0.0 |
| Gabon | 673.5(292.4,1128.2) | 396.1(181.8,659.3) | -1.6(-1.9,-1.2) | 0.0 | 531.1(113.1,982.3) | 373.1(166.2,642.2) | -1.0(-1.5,-0.4) | 0.0 | 141.8(6.2,527.1) | 22.7(0.5,130.1) | -5.8(-6.0,-5.6) | 0.0 |
| Gambia | 1987.9(1363.2,2667.2) | 1203.5(803.3,1666.5) | -1.6(-1.7,-1.5) | 0.0 | 343.0(168.4,606.4) | 191.7(100.7,342.7) | -1.8(-2.1,-1.5) | 0.0 | 1644.8(1097.3,2221.5) | 1011.5(665.5,1414.6) | -1.6(-1.6,-1.5) | 0.0 |
| Georgia | 388.1(141.4,672.4) | 91.9(34.5,157.3) | -4.7(-5.5,-4.0) | 0.0 | 210.6(41.6,482.7) | 65.5(19.1,131.7) | -3.8(-4.6,-3.0) | 0.0 | 177.1(26.9,440.8) | 26.4(3.7,74.1) | -6.1(-7.1,-5.0) | 0.0 |
| Germany | 67.9(30.9,110.5) | 17.3(2.3,36.8) | -4.4(-5.0,-3.8) | 0.0 | 67.9(30.8,110.5) | 17.3(2.3,36.8) | -4.4(-4.9,-3.8) | 0.0 | 0.0(0.0,0.3) | 0.0(0.0,0.0) | -8.7(-9.7,-7.8) | 0.0 |
| Ghana | 1750.8(1139.0,2366.8) | 908.2(560.2,1374.2) | -2.1(-2.2,-2.0) | 0.0 | 328.1(178.7,546.7) | 302.8(115.7,584.2) | -0.1(-0.8,0.5) | 0.7 | 1422.4(872.5,1962.2) | 605.3(319.8,987.9) | -2.8(-3.4,-2.2) | 0.0 |
| Greece | 92.9(28.9,165.8) | 25.2(6.5,46.1) | -4.0(-5.8,-2.2) | 0.0 | 91.0(27.1,159.3) | 25.2(6.5,46.1) | -4.0(-5.7,-2.2) | 0.0 | 1.8(0.0,13.4) | 0.1(0.0,0.6) | -10.6(-12.3,-8.9) | 0.0 |
| Greenland | 101.7(5.0,253.8) | 24.5(0.0,66.2) | -4.4(-4.6,-4.1) | 0.0 | 101.4(5.0,253.0) | 24.5(0.0,66.2) | -4.4(-4.6,-4.1) | 0.0 | 0.2(0.0,0.8) | 0.0(0.0,0.0) | -10.8(-11.6,-10.0) | 0.0 |
| Grenada | 383.1(190.5,604.8) | 181.5(61.1,328.5) | -2.3(-2.6,-2.0) | 0.0 | 177.8(49.8,361.0) | 177.4(59.1,324.2) | 0.0(-0.3,0.4) | 0.9 | 205.1(64.1,398.5) | 4.1(0.1,24.9) | -11.9(-12.4,-11.5) | 0.0 |
| Guam | 46.1(4.9,98.8) | 48.8(5.3,105.3) | 0.2(-0.3,0.7) | 0.4 | 45.7(4.8,98.7) | 48.5(5.0,105.4) | 0.2(-0.3,0.7) | 0.4 | 0.4(0.0,5.3) | 0.3(0.0,2.2) | -1.4(-2.2,-0.6) | 0.0 |
| Guatemala | 1359.2(952.3,1824.8) | 271.4(158.5,393.3) | -5.1(-5.6,-4.5) | 0.0 | 372.5(150.5,711.1) | 98.9(27.8,215.3) | -4.1(-4.6,-3.6) | 0.0 | 986.3(576.4,1458.4) | 172.4(76.9,296.3) | -5.5(-5.9,-5.1) | 0.0 |
| Guinea | 2293.2(1633.8,3092.5) | 1328.9(848.9,1886.3) | -1.7(-1.9,-1.5) | 0.0 | 324.6(165.1,563.7) | 177.6(85.0,305.0) | -2.0(-2.5,-1.5) | 0.0 | 1968.4(1348.4,2669.3) | 1151.3(737.7,1658.8) | -1.7(-1.9,-1.5) | 0.0 |
| Guinea-Bissau | 2460.6(1725.2,3369.1) | 1468.4(923.0,2057.3) | -1.7(-1.8,-1.6) | 0.0 | 365.3(190.5,637.4) | 202.7(103.9,357.9) | -1.9(-2.2,-1.5) | 0.0 | 2095.0(1467.3,2893.4) | 1265.4(778.7,1799.5) | -1.6(-1.7,-1.6) | 0.0 |
| Guyana | 1007.0(512.9,1687.5) | 397.0(173.7,715.4) | -3.1(-3.5,-2.6) | 0.0 | 354.6(122.6,799.7) | 336.6(130.6,635.5) | -0.2(-0.8,0.3) | 0.4 | 651.8(263.3,1197.5) | 60.1(9.1,170.1) | -7.7(-8.1,-7.2) | 0.0 |
| Haiti | 1132.8(728.6,1651.7) | 1093.7(723.8,1502.4) | -0.1(-0.3,0.0) | 0.1 | 74.6(21.5,168.2) | 78.1(31.9,159.5) | 0.2(0.0,0.4) | 0.1 | 1058.1(679.9,1518.8) | 1015.5(667.9,1396.3) | -0.2(-0.3,0.0) | 0.1 |
| Honduras | 675.8(439.1,947.7) | 249.5(145.9,383.6) | -3.2(-3.3,-3.1) | 0.0 | 111.2(47.4,214.2) | 52.7(18.3,112.1) | -2.3(-2.7,-1.9) | 0.0 | 564.4(358.8,797.7) | 196.7(111.8,303.5) | -3.4(-3.5,-3.2) | 0.0 |
| Hungary | 282.8(127.0,461.5) | 21.1(4.6,42.0) | -8.3(-9.0,-7.5) | 0.0 | 234.7(64.9,420.3) | 20.2(4.1,40.8) | -8.0(-8.5,-7.6) | 0.0 | 47.9(1.7,209.7) | 0.9(0.0,10.3) | -12.4(-13.4,-11.5) | 0.0 |
| Iceland | 19.3(0.7,48.7) | 3.6(0.0,10.2) | -5.2(-5.9,-4.6) | 0.0 | 19.3(0.8,48.5) | 3.6(0.0,10.2) | -5.2(-5.9,-4.6) | 0.0 | 0.0(0.0,0.3) | 0.0(0.0,0.0) | -9.2(-11.6,-6.8) | 0.0 |
| India | 1915.0(1684.4,2166.5) | 1046.7(843.9,1309.5) | -2.0(-2.2,-1.8) | 0.0 | 282.7(163.4,455.5) | 363.4(209.8,554.9) | 0.7(0.3,1.1) | 0.0 | 1632.1(1393.4,1885.2) | 683.1(482.0,908.7) | -2.7(-3.0,-2.5) | 0.0 |
| Indonesia | 939.0(788.1,1107.2) | 301.4(227.2,386.9) | -3.9(-4.1,-3.8) | 0.0 | 174.0(76.8,314.9) | 166.4(74.1,261.4) | -0.3(-0.7,0.2) | 0.2 | 764.7(607.7,943.8) | 134.8(66.5,235.1) | -5.7(-6.0,-5.3) | 0.0 |
| Iran (Islamic Republic of) | 780.0(632.6,948.4) | 80.4(62.6,101.3) | -7.1(-7.5,-6.7) | 0.0 | 659.3(494.7,822.2) | 80.0(62.2,101.0) | -6.6(-7.1,-6.1) | 0.0 | 120.4(48.7,250.5) | 0.3(0.0,1.7) | -17.1(-17.6,-16.6) | 0.0 |
| Iraq | 958.9(563.3,1420.4) | 321.6(171.8,503.9) | -3.5(-3.8,-3.2) | 0.0 | 826.1(438.9,1280.8) | 319.5(171.0,503.4) | -3.0(-3.5,-2.6) | 0.0 | 132.5(17.1,449.3) | 2.0(0.0,13.9) | -13.0(-14.0,-12.0) | 0.0 |
| Ireland | 44.8(5.6,89.7) | 10.2(0.0,29.0) | -4.8(-6.1,-3.4) | 0.0 | 44.4(5.6,89.2) | 10.2(0.0,29.0) | -4.7(-6.1,-3.3) | 0.0 | 0.5(0.0,3.9) | 0.0(0.0,0.0) | -16.1(-17.1,-15.1) | 0.0 |
| Israel | 112.9(48.3,183.4) | 12.5(4.3,23.2) | -6.9(-8.0,-5.6) | 0.0 | 112.6(48.2,182.7) | 12.5(4.3,23.2) | -6.8(-8.0,-5.6) | 0.0 | 0.3(0.0,3.1) | 0.0(0.0,0.0) | -13.0(-14.0,-12.0) | 0.0 |
| Italy | 108.3(87.4,129.9) | 16.2(11.0,21.8) | -6.2(-6.6,-5.8) | 0.0 | 107.2(85.3,128.8) | 16.2(11.0,21.8) | -6.2(-6.6,-5.7) | 0.0 | 1.1(0.0,7.7) | 0.0(0.0,0.2) | -12.0(-12.4,-11.6) | 0.0 |
| Jamaica | 640.3(354.9,961.9) | 227.4(92.5,416.6) | -3.3(-3.7,-3.0) | 0.0 | 161.0(40.1,374.7) | 168.3(56.7,329.4) | 0.2(-0.2,0.5) | 0.3 | 479.1(235.3,770.9) | 59.0(8.7,153.3) | -6.6(-6.9,-6.3) | 0.0 |
| Japan | 19.0(15.1,23.7) | 4.8(3.7,6.0) | -4.4(-4.9,-3.9) | 0.0 | 18.8(14.6,23.4) | 4.8(3.7,5.9) | -4.3(-4.8,-3.9) | 0.0 | 0.2(0.0,1.6) | 0.0(0.0,0.0) | -12.0(-12.7,-11.3) | 0.0 |
| Jordan | 351.5(174.5,559.9) | 152.1(75.2,251.6) | -2.7(-3.3,-2.2) | 0.0 | 343.9(169.3,539.4) | 152.0(75.1,251.5) | -2.7(-3.2,-2.1) | 0.0 | 7.5(1.5,23.1) | 0.1(0.0,0.5) | -12.4(-12.7,-12.1) | 0.0 |
| Kazakhstan | 209.9(88.4,341.6) | 68.2(26.5,119.0) | -3.7(-4.4,-2.9) | 0.0 | 149.7(43.6,282.5) | 66.8(26.3,113.9) | -2.5(-3.4,-1.7) | 0.0 | 60.1(5.4,177.8) | 1.3(0.0,12.8) | -11.6(-12.7,-10.6) | 0.0 |
| Kenya | 900.2(779.8,1039.6) | 737.5(585.5,913.6) | -0.6(-0.8,-0.5) | 0.0 | 57.9(35.1,92.6) | 68.5(37.1,118.7) | 0.9(-0.1,1.9) | 0.1 | 842.2(717.8,969.5) | 668.9(529.3,837.8) | -0.7(-0.9,-0.6) | 0.0 |
| Kiribati | 1045.8(694.6,1463.6) | 491.2(294.6,733.1) | -2.4(-2.6,-2.3) | 0.0 | 34.3(9.6,91.6) | 30.9(11.1,69.0) | -0.3(-0.5,-0.2) | 0.0 | 1011.3(682.7,1425.0) | 460.3(266.1,694.5) | -2.5(-2.7,-2.4) | 0.0 |
| Kuwait | 224.3(112.4,338.3) | 112.5(59.4,171.3) | -1.1(-2.2,-0.1) | 0.0 | 224.1(112.4,338.3) | 112.5(59.4,171.3) | -1.1(-2.2,-0.1) | 0.0 | 0.2(0.0,1.6) | 0.0(0.0,0.0) | -10.4(-11.5,-9.2) | 0.0 |
| Kyrgyzstan | 490.6(259.7,735.4) | 260.1(128.9,408.0) | -2.1(-2.6,-1.6) | 0.0 | 129.4(37.7,288.4) | 94.8(35.6,192.6) | -1.1(-2.1,-0.2) | 0.0 | 361.0(172.9,582.4) | 165.2(76.6,278.2) | -2.6(-3.0,-2.2) | 0.0 |
| Lao People's Democratic Republic | 1766.2(1170.9,2534.8) | 769.6(471.1,1154.2) | -2.6(-2.7,-2.6) | 0.0 | 139.2(53.1,286.8) | 175.0(50.4,369.4) | 0.8(0.7,0.9) | 0.0 | 1626.8(1105.9,2340.0) | 594.4(307.7,932.6) | -3.2(-3.3,-3.1) | 0.0 |
| Latvia | 148.2(74.0,230.1) | 17.3(1.6,35.8) | -6.9(-8.7,-5.1) | 0.0 | 117.9(41.4,209.2) | 16.5(1.4,35.3) | -6.3(-8.0,-4.7) | 0.0 | 30.2(2.1,103.3) | 0.8(0.0,6.7) | -11.6(-12.9,-10.3) | 0.0 |
| Lebanon | 233.0(85.7,411.6) | 64.9(25.5,116.8) | -4.2(-5.1,-3.3) | 0.0 | 201.5(70.2,367.7) | 64.6(25.5,116.7) | -3.7(-4.6,-2.8) | 0.0 | 31.4(3.6,107.7) | 0.3(0.0,2.3) | -13.8(-14.2,-13.5) | 0.0 |
| Lesotho | 1628.5(917.8,2418.8) | 1247.5(789.5,1829.0) | -0.9(-1.3,-0.5) | 0.0 | 150.7(79.1,268.5) | 163.6(74.8,304.5) | 0.4(-0.2,0.9) | 0.2 | 1477.8(828.3,2212.1) | 1083.9(666.1,1569.3) | -1.0(-1.5,-0.6) | 0.0 |
| Liberia | 2645.4(1842.8,3465.3) | 1287.2(830.0,1851.6) | -2.4(-3.2,-1.6) | 0.0 | 283.3(156.7,439.7) | 127.7(70.4,205.9) | -2.6(-3.6,-1.6) | 0.0 | 2361.9(1636.9,3115.5) | 1159.4(751.5,1652.4) | -2.3(-3.1,-1.5) | 0.0 |
| Libya | 403.8(205.8,635.3) | 233.2(113.3,382.8) | -2.1(-3.2,-0.9) | 0.0 | 399.7(203.9,635.5) | 232.3(113.2,381.4) | -2.1(-3.2,-0.9) | 0.0 | 3.9(0.0,39.0) | 0.9(0.0,6.4) | -4.3(-5.4,-3.2) | 0.0 |
| Lithuania | 114.2(46.7,186.9) | 10.1(0.7,23.2) | -7.7(-8.6,-6.8) | 0.0 | 101.3(38.3,168.3) | 10.0(0.8,23.1) | -7.4(-8.7,-6.1) | 0.0 | 12.9(0.8,58.1) | 0.1(0.0,0.9) | -14.5(-15.3,-13.8) | 0.0 |
| Luxembourg | 50.2(11.0,89.9) | 9.3(0.7,22.1) | -5.1(-6.0,-4.3) | 0.0 | 50.1(11.0,89.8) | 9.3(0.7,22.1) | -5.1(-6.0,-4.3) | 0.0 | 0.0(0.0,0.3) | 0.0(0.0,0.0) | -10.9(-14.0,-7.6) | 0.0 |
| Madagascar | 1200.0(849.6,1606.1) | 990.9(673.9,1423.7) | -0.6(-0.7,-0.5) | 0.0 | 32.0(17.8,52.8) | 36.6(20.2,63.5) | 0.5(0.2,0.7) | 0.0 | 1167.9(829.2,1568.3) | 954.3(647.4,1380.6) | -0.7(-0.8,-0.5) | 0.0 |
| Malawi | 1827.1(1333.0,2403.7) | 1024.1(658.6,1374.9) | -1.9(-2.0,-1.7) | 0.0 | 94.5(52.1,154.1) | 57.0(31.4,97.3) | -1.6(-2.2,-0.9) | 0.0 | 1732.6(1251.4,2306.5) | 967.1(609.7,1305.3) | -1.9(-2.0,-1.8) | 0.0 |
| Malaysia | 202.7(104.7,328.0) | 43.9(15.2,79.6) | -5.0(-6.2,-3.8) | 0.0 | 184.5(88.0,301.6) | 43.8(15.2,79.5) | -4.8(-6.0,-3.5) | 0.0 | 18.1(1.9,72.0) | 0.1(0.0,1.1) | -14.6(-15.1,-14.2) | 0.0 |
| Maldives | 1198.5(716.3,1737.9) | 86.4(20.4,170.0) | -8.2(-8.5,-8.0) | 0.0 | 220.7(61.9,502.9) | 77.8(17.0,160.8) | -3.5(-4.2,-2.7) | 0.0 | 977.5(539.3,1491.4) | 8.6(0.1,44.3) | -14.3(-14.6,-14.1) | 0.0 |
| Mali | 3166.8(2233.9,4182.1) | 2132.9(1457.2,2849.7) | -1.3(-1.4,-1.2) | 0.0 | 453.2(245.1,727.5) | 355.3(184.3,636.1) | -0.7(-1.0,-0.5) | 0.0 | 2713.2(1882.6,3626.9) | 1777.3(1167.6,2390.4) | -1.3(-1.4,-1.3) | 0.0 |
| Malta | 100.0(29.8,181.9) | 27.9(2.7,61.0) | -4.1(-5.1,-3.2) | 0.0 | 96.9(28.9,176.2) | 27.8(2.7,61.0) | -4.0(-5.0,-3.1) | 0.0 | 3.1(0.1,18.3) | 0.0(0.0,0.2) | -15.1(-16.1,-14.2) | 0.0 |
| Marshall Islands | 316.7(174.5,485.6) | 196.8(96.8,317.4) | -1.6(-1.8,-1.4) | 0.0 | 36.5(10.1,91.6) | 32.7(10.8,73.1) | -0.3(-0.6,0.0) | 0.0 | 280.2(146.3,440.1) | 164.2(79.6,269.7) | -1.8(-2.1,-1.5) | 0.0 |
| Mauritania | 1701.8(1213.3,2311.2) | 882.9(593.2,1205.1) | -2.1(-2.2,-2.0) | 0.0 | 458.1(233.6,767.9) | 378.8(196.8,622.2) | -0.6(-0.7,-0.5) | 0.0 | 1243.4(818.1,1743.0) | 504.1(289.8,740.3) | -2.9(-3.0,-2.8) | 0.0 |
| Mauritius | 134.9(24.3,253.1) | 59.3(12.3,121.0) | -2.3(-4.2,-0.3) | 0.0 | 85.8(13.5,183.5) | 58.4(11.9,119.2) | -1.0(-2.9,1.0) | 0.3 | 49.0(6.1,128.6) | 0.8(0.0,7.0) | -12.5(-14.3,-10.7) | 0.0 |
| Mexico | 439.9(363.0,513.1) | 95.3(71.3,120.8) | -4.8(-5.0,-4.7) | 0.0 | 320.8(166.6,444.0) | 78.3(45.3,107.1) | -4.4(-4.6,-4.3) | 0.0 | 118.8(29.1,268.6) | 17.0(3.0,47.7) | -6.2(-6.5,-5.9) | 0.0 |
| Micronesia (Federated States of) | 566.2(340.5,854.7) | 162.1(73.2,259.6) | -4.0(-4.2,-3.7) | 0.0 | 40.0(9.3,99.3) | 27.7(8.6,65.7) | -1.2(-1.5,-0.8) | 0.0 | 526.2(308.8,804.5) | 134.4(56.6,223.7) | -4.3(-4.6,-4.1) | 0.0 |
| Monaco | 23.2(1.2,55.6) | 8.0(0.8,18.1) | -3.3(-3.9,-2.8) | 0.0 | 23.2(1.2,55.6) | 8.0(0.8,18.1) | -3.3(-3.9,-2.8) | 0.0 | 0.0(0.0,0.0) | 0.0(0.0,0.0) | -7.4(-20.9,8.4) | 0.3 |
| Mongolia | 652.9(388.7,958.5) | 211.0(101.3,332.0) | -3.7(-4.0,-3.3) | 0.0 | 78.5(27.6,194.0) | 149.5(39.8,278.2) | 2.2(1.7,2.7) | 0.0 | 574.4(333.3,854.6) | 61.3(5.6,182.6) | -7.1(-7.7,-6.6) | 0.0 |
| Montenegro | 352.4(197.4,547.5) | 34.9(12.2,62.5) | -7.2(-8.0,-6.3) | 0.0 | 252.9(68.9,441.7) | 29.2(5.7,57.1) | -6.7(-7.8,-5.5) | 0.0 | 99.3(7.8,291.6) | 5.6(0.1,26.2) | -9.0(-9.7,-8.2) | 0.0 |
| Morocco | 708.0(375.8,1126.1) | 170.3(70.9,276.7) | -4.6(-4.8,-4.3) | 0.0 | 295.3(124.3,530.5) | 156.9(63.7,257.9) | -2.0(-2.3,-1.8) | 0.0 | 412.4(195.7,672.2) | 13.2(2.1,42.4) | -11.0(-11.7,-10.3) | 0.0 |
| Mozambique | 2205.3(1526.2,2930.7) | 1318.7(844.2,1882.5) | -1.6(-1.8,-1.5) | 0.0 | 93.3(56.7,137.9) | 70.0(39.4,120.0) | -0.9(-1.1,-0.6) | 0.0 | 2112.1(1453.1,2804.7) | 1248.6(800.5,1775.9) | -1.7(-1.9,-1.5) | 0.0 |
| Myanmar | 1314.0(884.5,1856.1) | 699.2(434.9,1051.1) | -2.1(-2.2,-2.0) | 0.0 | 125.7(51.6,245.5) | 192.5(62.1,393.3) | 1.4(1.3,1.5) | 0.0 | 1188.2(788.2,1695.8) | 506.6(283.1,771.2) | -2.9(-3.1,-2.6) | 0.0 |
| Namibia | 1172.8(753.1,1661.4) | 488.7(260.2,805.8) | -2.8(-2.9,-2.7) | 0.0 | 236.5(71.0,521.7) | 276.1(69.0,562.7) | 0.5(0.3,0.7) | 0.0 | 935.8(508.9,1371.0) | 212.4(38.7,491.1) | -4.8(-5.0,-4.5) | 0.0 |
| Nauru | 70.8(5.4,179.5) | 55.0(1.4,135.3) | -0.9(-1.3,-0.4) | 0.0 | 58.7(3.4,161.2) | 43.8(0.8,112.8) | -0.9(-1.2,-0.6) | 0.0 | 12.0(0.1,68.0) | 11.1(0.1,49.0) | -0.5(-1.0,-0.1) | 0.0 |
| Nepal | 2036.3(1367.5,2787.3) | 875.4(480.4,1312.3) | -2.7(-2.8,-2.6) | 0.0 | 263.3(123.7,513.8) | 200.9(101.3,365.3) | -1.1(-1.7,-0.4) | 0.0 | 1772.9(1172.7,2415.5) | 674.4(361.5,1011.2) | -3.0(-3.2,-2.8) | 0.0 |
| Netherlands | 68.1(19.1,121.2) | 18.6(1.3,42.4) | -4.0(-4.8,-3.2) | 0.0 | 68.0(19.1,120.1) | 18.6(1.3,42.4) | -4.0(-4.8,-3.2) | 0.0 | 0.1(0.0,0.7) | 0.0(0.0,0.0) | -11.4(-12.9,-9.9) | 0.0 |
| New Zealand | 24.6(2.8,53.5) | 14.3(1.6,33.0) | -1.5(-2.9,-0.1) | 0.0 | 24.0(2.7,53.0) | 14.3(1.6,32.5) | -1.4(-2.8,0.0) | 0.0 | 0.6(0.0,4.1) | 0.0(0.0,0.2) | -9.9(-10.7,-9.0) | 0.0 |
| Nicaragua | 836.0(564.2,1147.4) | 236.1(123.2,366.9) | -4.0(-4.1,-3.9) | 0.0 | 116.5(42.3,239.7) | 50.5(17.2,110.5) | -2.5(-3.5,-1.6) | 0.0 | 719.4(469.9,991.9) | 185.6(93.1,294.1) | -4.3(-4.4,-4.2) | 0.0 |
| Niger | 1688.1(1205.0,2296.2) | 1226.2(768.0,1724.2) | -1.1(-1.7,-0.5) | 0.0 | 276.2(142.4,483.5) | 173.5(82.5,301.6) | -1.5(-2.4,-0.6) | 0.0 | 1411.8(981.5,1926.8) | 1052.7(657.8,1483.9) | -1.0(-1.3,-0.8) | 0.0 |
| Nigeria | 1942.0(1706.8,2176.3) | 1625.1(1326.1,1958.3) | -0.6(-0.6,-0.5) | 0.0 | 478.9(252.0,762.2) | 531.7(246.5,935.3) | 0.4(-0.5,1.3) | 0.4 | 1462.7(1153.4,1768.6) | 1092.8(729.3,1502.9) | -1.0(-1.3,-0.7) | 0.0 |
| Niue | 166.2(59.1,290.8) | 103.4(9.2,222.1) | -1.3(-2.4,-0.2) | 0.0 | 44.3(7.7,106.4) | 85.7(6.6,189.4) | 2.3(1.4,3.2) | 0.0 | 121.8(44.3,220.9) | 17.7(0.8,80.8) | -5.7(-5.9,-5.5) | 0.0 |
| North Macedonia | 579.9(335.0,874.1) | 91.0(38.2,147.0) | -5.6(-7.9,-3.3) | 0.0 | 355.9(107.9,624.3) | 79.0(27.1,142.3) | -4.6(-6.9,-2.2) | 0.0 | 223.8(45.3,495.0) | 11.9(0.4,50.9) | -9.2(-11.3,-7.0) | 0.0 |
| Northern Mariana Islands | 38.0(4.4,81.5) | 26.8(2.9,62.3) | -1.0(-1.4,-0.7) | 0.0 | 37.4(4.1,81.4) | 26.2(2.9,62.1) | -1.1(-1.4,-0.8) | 0.0 | 0.6(0.0,7.4) | 0.6(0.0,5.8) | 0.0(-1.3,1.3) | 1.0 |
| Norway | 34.3(22.4,48.0) | 5.8(3.2,9.4) | -5.8(-6.5,-5.1) | 0.0 | 34.3(22.4,47.9) | 5.7(3.2,9.4) | -5.8(-6.5,-5.1) | 0.0 | 0.0(0.0,0.2) | 0.0(0.0,0.0) | -12.4(-13.0,-11.8) | 0.0 |
| Oman | 470.6(248.5,746.4) | 127.5(75.7,192.3) | -4.1(-4.5,-3.8) | 0.0 | 458.0(239.8,732.0) | 127.4(75.7,192.3) | -4.0(-4.3,-3.7) | 0.0 | 12.4(0.0,108.4) | 0.0(0.0,0.3) | -16.4(-17.1,-15.7) | 0.0 |
| Pakistan | 2051.5(1506.3,2623.5) | 1610.2(1128.8,2093.6) | -0.8(-0.9,-0.6) | 0.0 | 395.0(192.0,719.0) | 576.9(275.4,1015.0) | 0.9(0.4,1.4) | 0.0 | 1656.1(1145.9,2210.1) | 1032.9(592.1,1515.4) | -1.4(-1.6,-1.3) | 0.0 |
| Palau | 88.0(4.0,203.7) | 51.8(2.1,128.2) | -1.9(-2.6,-1.2) | 0.0 | 87.3(3.8,201.9) | 51.7(2.1,127.6) | -1.9(-2.6,-1.2) | 0.0 | 0.7(0.0,4.0) | 0.1(0.0,0.8) | -5.9(-6.5,-5.3) | 0.0 |
| Palestine | 393.1(207.0,622.6) | 130.3(65.7,217.7) | -3.6(-4.0,-3.3) | 0.0 | 230.4(111.3,382.2) | 121.0(61.3,207.4) | -2.1(-2.7,-1.5) | 0.0 | 162.6(79.2,282.5) | 9.3(3.0,21.7) | -9.1(-9.5,-8.6) | 0.0 |
| Panama | 286.7(147.7,449.8) | 57.2(13.5,114.6) | -5.1(-5.6,-4.7) | 0.0 | 131.8(35.4,258.6) | 54.5(13.5,112.3) | -2.8(-3.4,-2.3) | 0.0 | 154.7(52.2,303.7) | 2.6(0.0,23.5) | -12.9(-13.6,-12.3) | 0.0 |
| Papua New Guinea | 710.4(479.0,988.6) | 648.9(415.7,893.0) | -0.3(-0.3,-0.2) | 0.0 | 47.3(11.5,128.5) | 58.4(15.4,142.7) | 0.8(0.7,0.9) | 0.0 | 663.0(438.0,915.0) | 590.5(375.9,844.4) | -0.4(-0.4,-0.3) | 0.0 |
| Paraguay | 574.0(336.3,879.7) | 127.1(38.8,241.2) | -4.9(-5.2,-4.7) | 0.0 | 100.6(20.5,259.3) | 64.7(10.5,147.4) | -1.4(-1.7,-1.1) | 0.0 | 473.1(263.2,733.9) | 62.3(9.5,159.4) | -6.6(-6.9,-6.2) | 0.0 |
| Peru | 903.5(619.0,1268.4) | 152.9(74.2,260.8) | -5.8(-6.4,-5.1) | 0.0 | 450.5(197.5,806.0) | 130.6(51.4,230.5) | -4.0(-4.7,-3.4) | 0.0 | 452.5(194.4,769.0) | 22.1(1.4,92.9) | -9.4(-9.9,-8.9) | 0.0 |
| Philippines | 723.8(607.2,849.1) | 383.5(296.3,478.4) | -2.1(-2.4,-1.8) | 0.0 | 147.1(68.4,250.7) | 129.4(60.9,209.2) | -0.5(-0.8,-0.2) | 0.0 | 576.5(452.5,713.0) | 254.0(172.2,359.2) | -2.7(-3.1,-2.4) | 0.0 |
| Poland | 296.9(253.4,349.6) | 31.1(22.1,41.7) | -7.0(-7.8,-6.2) | 0.0 | 197.0(87.4,279.5) | 30.1(19.9,41.3) | -5.8(-6.3,-5.3) | 0.0 | 99.7(24.1,212.6) | 1.0(0.0,8.6) | -13.9(-14.9,-13.0) | 0.0 |
| Portugal | 75.6(10.9,151.7) | 8.9(0.7,21.5) | -6.7(-8.3,-5.2) | 0.0 | 70.5(9.8,146.9) | 8.9(0.7,21.4) | -6.5(-8.1,-5.0) | 0.0 | 5.1(0.1,25.4) | 0.0(0.0,0.3) | -15.6(-16.4,-14.7) | 0.0 |
| Puerto Rico | 69.2(7.3,171.1) | 19.8(0.0,49.9) | -3.8(-4.2,-3.4) | 0.0 | 68.9(7.3,170.3) | 19.8(0.0,49.9) | -3.8(-4.2,-3.4) | 0.0 | 0.3(0.0,2.3) | 0.0(0.0,0.1) | -10.7(-11.6,-9.8) | 0.0 |
| Qatar | 321.5(212.1,464.1) | 62.9(37.8,92.5) | -5.1(-5.3,-4.9) | 0.0 | 321.4(212.1,464.0) | 62.9(37.8,92.5) | -5.1(-5.3,-4.9) | 0.0 | 0.0(0.0,0.1) | 0.0(0.0,0.0) | -13.0(-14.1,-12.0) | 0.0 |
| Republic of Korea | 84.4(30.8,147.4) | 18.7(6.9,33.2) | -4.8(-5.5,-4.0) | 0.0 | 80.3(28.5,143.9) | 18.7(6.9,33.2) | -4.7(-5.6,-3.8) | 0.0 | 4.1(0.2,17.0) | 0.0(0.0,0.0) | -20.0(-20.8,-19.1) | 0.0 |
| Republic of Moldova | 342.7(192.5,508.3) | 64.4(21.1,122.7) | -5.4(-6.4,-4.3) | 0.0 | 86.7(25.6,185.2) | 36.4(9.8,77.2) | -2.8(-3.8,-1.8) | 0.0 | 256.0(135.8,398.1) | 28.0(7.1,65.0) | -7.0(-7.8,-6.1) | 0.0 |
| Romania | 216.0(114.8,331.0) | 41.0(14.1,70.7) | -5.3(-6.6,-4.0) | 0.0 | 131.9(33.4,238.6) | 39.0(12.4,69.3) | -4.0(-5.2,-2.8) | 0.0 | 83.9(14.7,197.8) | 2.0(0.0,15.0) | -12.0(-13.0,-10.9) | 0.0 |
| Russian Federation | 186.9(160.3,212.2) | 22.2(17.8,27.0) | -7.2(-8.5,-5.9) | 0.0 | 176.0(143.4,204.9) | 21.8(16.9,27.0) | -7.0(-8.3,-5.7) | 0.0 | 10.7(1.7,43.1) | 0.4(0.0,2.8) | -10.5(-11.7,-9.4) | 0.0 |
| Rwanda | 1789.6(1259.0,2460.5) | 768.0(491.7,1100.3) | -2.6(-3.2,-2.0) | 0.0 | 128.3(63.0,246.6) | 47.9(19.6,93.5) | -3.1(-3.5,-2.8) | 0.0 | 1661.2(1176.4,2270.1) | 720.0(461.2,1039.5) | -2.5(-3.2,-1.9) | 0.0 |
| Saint Kitts and Nevis | 175.6(42.2,343.3) | 62.8(3.7,148.4) | -3.2(-3.5,-2.8) | 0.0 | 136.3(24.0,290.6) | 62.0(3.6,148.0) | -2.4(-2.8,-2.0) | 0.0 | 39.2(2.7,154.6) | 0.8(0.0,7.7) | -12.0(-12.6,-11.5) | 0.0 |
| Saint Lucia | 404.7(224.5,663.4) | 264.5(96.2,493.6) | -1.4(-1.9,-0.9) | 0.0 | 213.9(64.7,421.0) | 251.8(92.2,465.8) | 0.5(0.2,0.8) | 0.0 | 190.6(49.0,408.6) | 12.6(0.4,62.2) | -8.5(-8.9,-8.1) | 0.0 |
| Saint Vincent and the Grenadines | 448.8(227.2,705.2) | 180.9(65.2,325.6) | -2.9(-3.2,-2.7) | 0.0 | 210.2(65.1,431.8) | 171.6(62.0,310.9) | -0.7(-0.9,-0.6) | 0.0 | 238.4(72.0,460.1) | 9.3(0.5,39.6) | -10.0(-10.4,-9.7) | 0.0 |
| Samoa | 470.5(302.9,728.0) | 202.8(115.4,307.5) | -2.7(-2.9,-2.6) | 0.0 | 37.8(7.8,109.8) | 33.7(7.5,83.0) | -0.3(-0.6,-0.1) | 0.0 | 432.5(272.2,659.7) | 169.1(85.1,261.5) | -3.0(-3.2,-2.8) | 0.0 |
| San Marino | 52.2(5.9,108.1) | 5.6(0.2,14.8) | -6.8(-7.0,-6.6) | 0.0 | 52.2(5.7,108.1) | 5.6(0.2,14.8) | -6.8(-7.0,-6.6) | 0.0 | 0.0(0.0,0.1) | 0.0(0.0,0.0) | -11.4(-14.4,-8.4) | 0.0 |
| Sao Tome and Principe | 892.1(605.9,1196.4) | 282.4(156.5,430.4) | -3.5(-4.1,-3.0) | 0.0 | 74.1(38.8,130.2) | 60.2(23.9,115.6) | -0.1(-1.4,1.2) | 0.8 | 818.0(543.3,1099.3) | 222.2(121.5,343.3) | -4.1(-4.3,-3.9) | 0.0 |
| Saudi Arabia | 554.4(276.5,888.7) | 62.3(35.7,98.2) | -6.9(-7.4,-6.5) | 0.0 | 549.0(275.6,878.4) | 62.3(35.8,98.1) | -6.9(-7.3,-6.5) | 0.0 | 5.1(0.0,47.0) | 0.0(0.0,0.0) | -19.6(-20.0,-19.2) | 0.0 |
| Senegal | 1668.3(1138.8,2298.1) | 1011.7(660.1,1443.5) | -1.6(-1.7,-1.4) | 0.0 | 394.5(196.6,661.9) | 147.7(63.4,281.3) | -2.9(-3.6,-2.2) | 0.0 | 1273.6(859.8,1828.2) | 863.8(548.5,1238.1) | -1.2(-1.3,-1.2) | 0.0 |
| Serbia | 597.9(324.2,938.3) | 67.7(29.9,115.1) | -6.6(-8.7,-4.6) | 0.0 | 392.8(101.0,742.3) | 58.7(19.6,104.1) | -5.8(-7.2,-4.3) | 0.0 | 204.8(25.5,523.6) | 8.9(0.3,41.3) | -9.8(-11.5,-8.1) | 0.0 |
| Seychelles | 71.5(9.6,154.6) | 58.1(6.1,122.0) | -0.5(-1.0,0.0) | 0.0 | 62.3(8.1,134.5) | 57.9(6.2,121.4) | -0.2(-0.7,0.2) | 0.3 | 9.2(0.3,47.1) | 0.2(0.0,2.2) | -12.1(-13.4,-10.9) | 0.0 |
| Sierra Leone | 2790.5(1932.9,3728.6) | 1612.4(1099.1,2197.4) | -1.7(-1.9,-1.6) | 0.0 | 320.7(171.7,546.6) | 171.1(90.0,294.3) | -1.8(-2.7,-0.9) | 0.0 | 2469.7(1688.3,3348.9) | 1441.2(983.9,1964.6) | -1.7(-2.0,-1.5) | 0.0 |
| Singapore | 71.7(32.1,119.6) | 8.7(2.6,15.9) | -6.7(-8.2,-5.2) | 0.0 | 70.2(31.5,119.3) | 8.7(2.6,15.9) | -6.6(-8.1,-5.1) | 0.0 | 1.5(0.0,13.4) | 0.0(0.0,0.0) | -18.8(-20.9,-16.6) | 0.0 |
| Slovakia | 171.5(79.7,268.0) | 29.7(8.1,55.8) | -5.8(-7.2,-4.3) | 0.0 | 167.9(76.9,262.9) | 29.6(8.0,55.7) | -5.7(-7.1,-4.3) | 0.0 | 3.5(0.1,24.1) | 0.1(0.0,0.5) | -12.2(-13.4,-11.0) | 0.0 |
| Slovenia | 88.5(30.5,149.6) | 9.2(1.8,19.6) | -7.4(-8.7,-6.0) | 0.0 | 82.8(27.6,147.8) | 9.0(1.7,19.5) | -7.1(-7.9,-6.3) | 0.0 | 5.7(0.0,41.6) | 0.1(0.0,1.6) | -11.6(-12.3,-10.8) | 0.0 |
| Solomon Islands | 490.5(318.2,695.1) | 280.2(164.0,412.9) | -1.8(-1.9,-1.7) | 0.0 | 18.4(4.8,52.6) | 13.9(4.1,32.5) | -0.9(-1.1,-0.8) | 0.0 | 472.0(308.5,656.6) | 266.3(156.2,391.2) | -1.8(-1.9,-1.8) | 0.0 |
| Somalia | 1675.8(1130.0,2230.0) | 1413.6(932.3,1957.8) | -0.6(-0.7,-0.4) | 0.0 | 80.8(45.0,125.0) | 73.0(42.9,118.3) | -0.3(-0.9,0.4) | 0.4 | 1595.0(1082.0,2127.6) | 1340.6(885.3,1859.7) | -0.6(-0.8,-0.4) | 0.0 |
| South Africa | 875.7(699.3,1076.1) | 484.6(357.9,642.1) | -1.7(-2.0,-1.4) | 0.0 | 385.5(199.9,621.8) | 376.9(231.4,538.5) | 0.2(-0.3,0.6) | 0.5 | 489.8(293.8,742.3) | 107.5(27.0,245.4) | -4.7(-5.2,-4.2) | 0.0 |
| South Sudan | 1748.1(1174.4,2375.3) | 1965.2(1290.1,2746.5) | 0.3(0.2,0.5) | 0.0 | 382.9(124.6,792.4) | 208.1(98.3,397.5) | -2.1(-2.6,-1.5) | 0.0 | 1365.0(824.2,1925.9) | 1756.9(1149.1,2457.9) | 0.8(0.7,1.0) | 0.0 |
| Spain | 66.8(25.5,109.3) | 10.5(1.3,23.0) | -5.8(-6.8,-4.8) | 0.0 | 64.3(23.8,107.0) | 10.5(1.3,23.1) | -5.7(-6.6,-4.7) | 0.0 | 2.4(0.0,16.7) | 0.0(0.0,0.3) | -13.8(-14.4,-13.2) | 0.0 |
| Sri Lanka | 610.2(401.4,850.1) | 105.1(52.7,175.1) | -5.4(-6.8,-4.0) | 0.0 | 72.2(25.4,157.6) | 61.0(11.5,127.1) | 0.0(-1.4,1.3) | 1.0 | 537.8(339.6,748.5) | 44.1(7.0,105.3) | -7.8(-9.3,-6.3) | 0.0 |
| Sudan | 1772.5(1032.3,2574.0) | 671.5(372.9,1059.3) | -3.1(-3.2,-3.1) | 0.0 | 300.2(151.6,538.0) | 299.1(138.3,565.5) | 0.0(-0.3,0.4) | 1.0 | 1472.2(844.0,2170.3) | 372.1(169.5,654.5) | -4.4(-4.4,-4.3) | 0.0 |
| Suriname | 853.2(450.5,1348.5) | 405.3(204.3,664.0) | -2.4(-2.6,-2.3) | 0.0 | 556.3(156.2,1013.6) | 382.3(181.0,632.6) | -1.2(-1.7,-0.8) | 0.0 | 296.3(50.4,746.1) | 22.8(0.6,132.8) | -7.9(-8.3,-7.5) | 0.0 |
| Sweden | 25.5(6.1,50.0) | 5.1(0.6,11.5) | -5.0(-6.3,-3.8) | 0.0 | 25.5(6.1,49.9) | 5.1(0.6,11.6) | -5.0(-6.3,-3.7) | 0.0 | 0.0(0.0,0.4) | 0.0(0.0,0.0) | -10.3(-10.9,-9.6) | 0.0 |
| Switzerland | 46.5(12.0,88.1) | 13.3(0.3,32.2) | -4.2(-5.1,-3.4) | 0.0 | 46.5(12.0,88.1) | 13.3(0.3,32.2) | -4.2(-5.1,-3.4) | 0.0 | 0.0(0.0,0.1) | 0.0(0.0,0.0) | -7.4(-9.9,-4.8) | 0.0 |
| Syrian Arab Republic | 438.9(203.9,723.7) | 95.2(46.6,152.3) | -4.9(-5.8,-4.0) | 0.0 | 350.5(164.7,596.1) | 94.6(46.6,151.5) | -4.3(-5.1,-3.4) | 0.0 | 88.2(29.6,184.6) | 0.6(0.2,1.5) | -15.5(-16.4,-14.5) | 0.0 |
| Taiwan (Province of China) | 33.9(15.2,55.0) | 26.2(7.0,50.0) | -1.0(-2.8,0.8) | 0.3 | 26.6(7.4,48.1) | 26.0(6.8,50.0) | -0.2(-2.2,1.8) | 0.8 | 7.3(0.2,27.8) | 0.2(0.0,1.3) | -11.9(-14.2,-9.5) | 0.0 |
| Tajikistan | 614.7(382.5,868.3) | 467.7(247.8,716.0) | -0.9(-1.1,-0.7) | 0.0 | 133.5(39.3,309.0) | 157.9(67.3,289.7) | 0.6(0.2,1.0) | 0.0 | 480.9(272.3,740.1) | 309.7(142.4,496.9) | -1.4(-1.7,-1.2) | 0.0 |
| Thailand | 560.9(351.6,855.0) | 61.7(32.6,96.5) | -6.9(-7.5,-6.3) | 0.0 | 200.3(73.7,399.1) | 57.4(26.7,93.0) | -3.9(-4.4,-3.5) | 0.0 | 360.5(168.7,607.1) | 4.3(0.1,26.8) | -13.6(-14.2,-13.0) | 0.0 |
| Timor-Leste | 1325.3(877.5,1790.3) | 623.3(374.4,886.1) | -2.4(-2.5,-2.3) | 0.0 | 99.0(31.3,233.9) | 131.8(31.5,312.8) | 0.7(0.3,1.1) | 0.0 | 1226.2(822.3,1664.3) | 491.3(260.3,751.6) | -2.9(-3.0,-2.7) | 0.0 |
| Togo | 1903.3(1332.5,2556.8) | 1132.2(728.1,1641.8) | -1.7(-1.7,-1.6) | 0.0 | 291.7(151.2,519.8) | 163.0(83.7,286.2) | -1.8(-2.8,-0.8) | 0.0 | 1611.4(1085.9,2167.5) | 969.1(618.5,1399.1) | -1.6(-1.9,-1.4) | 0.0 |
| Tokelau | 55.4(3.2,143.4) | 109.9(0.0,272.5) | 2.6(0.6,4.6) | 0.0 | 50.2(2.8,128.1) | 109.1(0.0,271.1) | 2.8(0.8,4.8) | 0.0 | 5.2(0.2,17.6) | 0.8(0.0,3.5) | -4.8(-5.7,-3.9) | 0.0 |
| Tonga | 302.0(164.8,453.1) | 116.2(51.8,186.2) | -3.1(-3.3,-2.9) | 0.0 | 25.9(6.4,72.5) | 28.4(6.9,65.0) | 0.3(0.1,0.5) | 0.0 | 276.1(152.6,423.3) | 87.8(36.1,151.3) | -3.7(-3.9,-3.5) | 0.0 |
| Trinidad and Tobago | 332.0(107.1,630.2) | 149.8(50.8,297.0) | -2.5(-2.9,-2.1) | 0.0 | 327.4(104.9,622.7) | 149.8(50.8,297.0) | -2.4(-2.8,-2.0) | 0.0 | 4.6(0.1,29.3) | 0.0(0.0,0.4) | -14.5(-15.6,-13.4) | 0.0 |
| Tunisia | 503.3(269.5,801.7) | 112.0(49.9,177.7) | -4.6(-5.1,-4.1) | 0.0 | 335.1(164.9,589.3) | 111.5(49.4,177.0) | -3.3(-3.9,-2.8) | 0.0 | 168.0(55.5,348.4) | 0.5(0.0,2.2) | -17.5(-17.9,-17.1) | 0.0 |
| Turkey | 652.6(344.6,1010.3) | 93.7(42.9,153.4) | -6.1(-6.4,-5.8) | 0.0 | 491.3(178.3,845.4) | 93.1(42.8,153.5) | -5.4(-5.9,-5.0) | 0.0 | 160.9(24.3,456.4) | 0.5(0.0,4.7) | -17.1(-17.7,-16.4) | 0.0 |
| Turkmenistan | 208.0(64.8,393.4) | 171.0(56.6,296.6) | -0.9(-1.8,0.1) | 0.1 | 203.0(63.4,373.5) | 170.8(56.6,296.5) | -0.8(-1.7,0.1) | 0.1 | 5.0(0.6,20.6) | 0.2(0.0,1.5) | -10.5(-11.5,-9.5) | 0.0 |
| Tuvalu | 641.1(372.7,991.5) | 70.0(9.0,157.4) | -7.0(-7.2,-6.8) | 0.0 | 14.3(3.9,34.1) | 11.3(1.3,29.4) | -0.7(-0.9,-0.5) | 0.0 | 626.8(366.6,958.3) | 58.6(7.6,131.5) | -7.5(-7.7,-7.3) | 0.0 |
| Uganda | 1581.0(1114.4,2119.9) | 1058.9(704.2,1508.9) | -1.3(-1.7,-0.9) | 0.0 | 106.7(65.1,170.2) | 106.1(55.9,180.6) | 0.1(-0.4,0.5) | 0.8 | 1474.3(1035.3,1971.3) | 952.8(635.1,1362.3) | -1.4(-1.6,-1.2) | 0.0 |
| Ukraine | 199.0(93.6,331.1) | 42.1(6.9,82.1) | -4.9(-5.7,-4.2) | 0.0 | 178.8(75.8,292.9) | 38.1(5.5,76.4) | -5.0(-5.7,-4.3) | 0.0 | 20.1(1.5,83.5) | 4.1(0.3,16.4) | -5.2(-6.3,-4.1) | 0.0 |
| United Arab Emirates | 342.5(195.8,522.6) | 63.6(39.0,98.2) | -5.4(-6.3,-4.5) | 0.0 | 342.4(196.5,522.6) | 63.6(39.0,98.2) | -5.4(-6.3,-4.5) | 0.0 | 0.0(0.0,0.0) | 0.0(0.0,0.0) | -12.6(-15.9,-9.2) | 0.0 |
| United Kingdom | 67.1(59.8,75.2) | 20.2(15.9,24.9) | -3.9(-4.5,-3.3) | 0.0 | 67.0(59.8,75.2) | 20.2(15.9,24.9) | -3.9(-4.5,-3.2) | 0.0 | 0.1(0.0,0.5) | 0.0(0.0,0.0) | -9.0(-9.6,-8.4) | 0.0 |
| United Republic of Tanzania | 1365.2(879.3,1867.8) | 1048.0(651.6,1561.1) | -0.8(-1.0,-0.7) | 0.0 | 64.5(36.4,103.7) | 88.4(40.0,172.1) | 1.3(0.8,1.8) | 0.0 | 1300.8(841.7,1797.4) | 959.6(597.4,1437.5) | -1.0(-1.1,-0.9) | 0.0 |
| United States of America | 64.3(50.3,78.0) | 17.6(12.5,23.7) | -3.9(-4.3,-3.6) | 0.0 | 64.2(50.2,78.0) | 17.6(12.5,23.6) | -3.9(-4.3,-3.5) | 0.0 | 0.0(0.0,0.4) | 0.0(0.0,0.1) | -5.9(-6.7,-5.1) | 0.0 |
| United States Virgin Islands | 84.2(11.8,190.4) | 17.4(1.3,44.8) | -4.6(-4.9,-4.3) | 0.0 | 82.5(11.7,188.4) | 17.3(1.3,44.8) | -4.6(-4.8,-4.3) | 0.0 | 1.6(0.0,14.8) | 0.0(0.0,0.1) | -12.1(-14.9,-9.2) | 0.0 |
| Uruguay | 153.8(42.1,266.2) | 24.7(4.6,54.6) | -5.5(-7.1,-4.0) | 0.0 | 116.5(31.4,227.6) | 24.1(4.2,53.6) | -4.7(-6.3,-3.1) | 0.0 | 37.3(4.8,114.3) | 0.6(0.0,4.7) | -12.6(-14.0,-11.2) | 0.0 |
| Uzbekistan | 336.4(174.4,540.1) | 253.8(133.8,405.0) | -1.0(-2.1,0.1) | 0.1 | 135.2(51.4,278.4) | 205.8(94.9,351.0) | 1.3(0.6,2.0) | 0.0 | 201.0(90.5,360.7) | 48.0(8.7,137.1) | -4.9(-5.5,-4.2) | 0.0 |
| Vanuatu | 614.8(387.2,855.0) | 418.2(271.6,606.2) | -1.2(-1.3,-1.1) | 0.0 | 23.1(5.6,62.5) | 26.1(8.4,64.8) | 0.4(0.3,0.6) | 0.0 | 591.6(370.8,836.1) | 392.1(253.0,566.7) | -1.3(-1.4,-1.2) | 0.0 |
| Venezuela (Bolivarian Republic of) | 318.3(135.9,523.5) | 149.5(50.1,276.3) | -2.4(-4.1,-0.5) | 0.0 | 309.8(130.4,517.8) | 146.6(49.5,272.8) | -2.3(-4.1,-0.5) | 0.0 | 8.5(0.3,48.2) | 2.9(0.1,18.2) | -3.1(-5.0,-1.2) | 0.0 |
| Viet Nam | 919.4(625.8,1251.3) | 182.4(89.7,293.5) | -5.1(-5.2,-4.9) | 0.0 | 45.9(18.9,95.0) | 68.7(22.3,141.3) | 1.4(1.2,1.6) | 0.0 | 873.5(596.6,1196.4) | 113.6(47.1,205.1) | -6.4(-6.5,-6.3) | 0.0 |
| Yemen | 1772.6(1280.9,2471.1) | 786.0(522.5,1169.0) | -2.7(-2.9,-2.4) | 0.0 | 381.7(186.9,621.0) | 340.9(173.8,555.1) | -0.5(-1.4,0.3) | 0.2 | 1390.7(944.2,1977.2) | 444.9(264.0,688.9) | -3.7(-3.7,-3.6) | 0.0 |
| Zambia | 1087.6(720.6,1449.8) | 857.4(515.2,1263.8) | -0.7(-0.9,-0.6) | 0.0 | 96.0(48.3,162.3) | 119.7(49.6,247.5) | 1.0(0.1,1.8) | 0.0 | 991.5(662.6,1340.6) | 737.6(432.7,1117.7) | -0.9(-1.0,-0.8) | 0.0 |
| Zimbabwe | 896.0(580.4,1213.0) | 1014.7(646.2,1454.5) | 0.4(0.2,0.6) | 0.0 | 99.1(44.2,197.7) | 86.6(43.9,156.1) | -0.4(-0.8,0.1) | 0.1 | 796.9(515.6,1081.4) | 928.0(591.4,1338.0) | 0.5(0.3,0.6) | 0.0 |
