# Supplementary material for: Global, regional, and national burden of neonatal diseases attributable to particulate matter pollution from 1990 to 2021
Source: Front Public Health. 2025 Jun 9;13:1556340. doi: 10.3389/fpubh.2025.1556340 (PMC12183240; doi:10.3389/fpubh.2025.1556340)
Supplement: Supplementary file 4 [file Table_2.DOCX]

Table S2 APCs from joinpoint regression for 204 countries and territories from 1990 to 2021.

| location | Risk | joinpoint | Segment | Segment.Start | Segment.End | *P*.Value | APC_95CI |
| --- | --- | --- | --- | --- | --- | --- | --- |
| Afghanistan | APMP | 3 | 0 | 1990 | 1994 | 0.000 | -8.40(-12.00,-4.80) |
| Afghanistan | APMP | 3 | 1 | 1994 | 2010 | 0.000 | -2.80(-3.30,-2.30) |
| Afghanistan | APMP | 3 | 2 | 2010 | 2015 | 0.001 | 8.00(3.90,12.40) |
| Afghanistan | APMP | 3 | 3 | 2015 | 2021 | 0.000 | -5.50(-7.40,-3.40) |
| Afghanistan | HAP | 3 | 0 | 1990 | 1993 | 0.000 | -3.90(-4.70,-3.10) |
| Afghanistan | HAP | 3 | 1 | 1993 | 2001 | 0.000 | -0.60(-0.80,-0.40) |
| Afghanistan | HAP | 3 | 2 | 2001 | 2009 | 0.000 | -2.00(-2.20,-1.80) |
| Afghanistan | HAP | 3 | 3 | 2009 | 2021 | 0.000 | -2.80(-2.90,-2.70) |
| Afghanistan | PMP | 3 | 0 | 1990 | 1993 | 0.000 | -4.60(-5.80,-3.30) |
| Afghanistan | PMP | 3 | 1 | 1993 | 2001 | 0.000 | -0.90(-1.20,-0.50) |
| Afghanistan | PMP | 3 | 2 | 2001 | 2017 | 0.000 | -2.10(-2.20,-2.00) |
| Afghanistan | PMP | 3 | 3 | 2017 | 2021 | 0.000 | -3.60(-4.30,-2.80) |
| Albania | APMP | 4 | 0 | 1990 | 1992 | 0.000 | -4.80(-7.10,-2.50) |
| Albania | APMP | 4 | 1 | 1992 | 1996 | 0.743 | -0.20(-1.40,1.00) |
| Albania | APMP | 4 | 2 | 1996 | 2008 | 0.000 | 4.70(4.50,4.90) |
| Albania | APMP | 4 | 3 | 2008 | 2014 | 0.043 | 0.60(0.00,1.10) |
| Albania | APMP | 4 | 4 | 2014 | 2021 | 0.000 | -5.00(-5.30,-4.70) |
| Albania | HAP | 5 | 0 | 1990 | 1996 | 0.000 | 0.70(0.40,1.00) |
| Albania | HAP | 5 | 1 | 1996 | 2001 | 0.000 | -2.00(-2.60,-1.50) |
| Albania | HAP | 5 | 2 | 2001 | 2006 | 0.000 | -7.80(-8.30,-7.30) |
| Albania | HAP | 5 | 3 | 2006 | 2010 | 0.000 | -15.30(-16.00,-14.50) |
| Albania | HAP | 5 | 4 | 2010 | 2018 | 0.000 | -12.80(-13.00,-12.60) |
| Albania | HAP | 5 | 5 | 2018 | 2021 | 0.000 | -6.60(-7.50,-5.80) |
| Albania | PMP | 5 | 0 | 1990 | 2000 | 0.617 | 0.00(-0.20,0.10) |
| Albania | PMP | 5 | 1 | 2000 | 2006 | 0.000 | -4.30(-4.70,-3.90) |
| Albania | PMP | 5 | 2 | 2006 | 2010 | 0.000 | -8.30(-9.10,-7.50) |
| Albania | PMP | 5 | 3 | 2010 | 2015 | 0.000 | -5.30(-5.80,-4.80) |
| Albania | PMP | 5 | 4 | 2015 | 2018 | 0.000 | -8.40(-10.00,-6.70) |
| Albania | PMP | 5 | 5 | 2018 | 2021 | 0.000 | -4.90(-5.70,-4.00) |
| Algeria | APMP | 4 | 0 | 1990 | 1995 | 0.124 | -0.50(-1.10,0.10) |
| Algeria | APMP | 4 | 1 | 1995 | 2000 | 0.000 | 2.20(1.30,3.10) |
| Algeria | APMP | 4 | 2 | 2000 | 2011 | 0.000 | -4.00(-4.20,-3.80) |
| Algeria | APMP | 4 | 3 | 2011 | 2015 | 0.212 | 0.90(-0.50,2.30) |
| Algeria | APMP | 4 | 4 | 2015 | 2021 | 0.000 | -5.90(-6.30,-5.50) |
| Algeria | HAP | 5 | 0 | 1990 | 1996 | 0.000 | -4.00(-4.40,-3.60) |
| Algeria | HAP | 5 | 1 | 1996 | 2001 | 0.000 | -9.00(-9.70,-8.20) |
| Algeria | HAP | 5 | 2 | 2001 | 2006 | 0.000 | -18.70(-19.40,-18.10) |
| Algeria | HAP | 5 | 3 | 2006 | 2010 | 0.000 | -21.90(-22.90,-20.80) |
| Algeria | HAP | 5 | 4 | 2010 | 2019 | 0.000 | -15.80(-16.00,-15.50) |
| Algeria | HAP | 5 | 5 | 2019 | 2021 | 0.000 | -7.30(-9.70,-4.90) |
| Algeria | PMP | 4 | 0 | 1990 | 1995 | 0.011 | -0.80(-1.40,-0.20) |
| Algeria | PMP | 4 | 1 | 1995 | 2000 | 0.001 | 1.70(0.80,2.60) |
| Algeria | PMP | 4 | 2 | 2000 | 2010 | 0.000 | -4.50(-4.70,-4.30) |
| Algeria | PMP | 4 | 3 | 2010 | 2015 | 0.935 | 0.00(-0.90,0.80) |
| Algeria | PMP | 4 | 4 | 2015 | 2021 | 0.000 | -5.80(-6.20,-5.40) |
| American Samoa | APMP | 3 | 0 | 1990 | 2003 | 0.000 | -2.00(-2.20,-1.70) |
| American Samoa | APMP | 3 | 1 | 2003 | 2011 | 0.000 | -4.10(-4.80,-3.40) |
| American Samoa | APMP | 3 | 2 | 2011 | 2018 | 0.000 | 2.30(1.30,3.20) |
| American Samoa | APMP | 3 | 3 | 2018 | 2021 | 0.216 | -1.60(-4.20,1.00) |
| American Samoa | HAP | 4 | 0 | 1990 | 2001 | 0.339 | -0.20(-0.60,0.20) |
| American Samoa | HAP | 4 | 1 | 2001 | 2007 | 0.000 | -3.30(-4.60,-2.00) |
| American Samoa | HAP | 4 | 2 | 2007 | 2012 | 0.923 | 0.10(-1.80,2.00) |
| American Samoa | HAP | 4 | 3 | 2012 | 2016 | 0.023 | 3.60(0.60,6.60) |
| American Samoa | HAP | 4 | 4 | 2016 | 2021 | 0.024 | -1.50(-2.80,-0.20) |
| American Samoa | PMP | 3 | 0 | 1990 | 2002 | 0.000 | -1.70(-2.00,-1.30) |
| American Samoa | PMP | 3 | 1 | 2002 | 2011 | 0.000 | -3.60(-4.20,-3.00) |
| American Samoa | PMP | 3 | 2 | 2011 | 2017 | 0.000 | 2.50(1.30,3.80) |
| American Samoa | PMP | 3 | 3 | 2017 | 2021 | 0.249 | -1.00(-2.70,0.80) |
| Andorra | APMP | 4 | 0 | 1990 | 2002 | 0.000 | -7.50(-8.30,-6.70) |
| Andorra | APMP | 4 | 1 | 2002 | 2005 | 0.251 | 8.20(-5.90,24.60) |
| Andorra | APMP | 4 | 2 | 2005 | 2011 | 0.000 | -11.40(-14.20,-8.60) |
| Andorra | APMP | 4 | 3 | 2011 | 2019 | 0.000 | -6.30(-8.00,-4.50) |
| Andorra | APMP | 4 | 4 | 2019 | 2021 | 0.000 | -28.40(-37.80,-17.60) |
| Andorra | HAP | 4 | 0 | 1990 | 2002 | 0.000 | -13.60(-16.10,-10.90) |
| Andorra | HAP | 4 | 1 | 2002 | 2005 | 0.427 | 21.80(-26.90,102.90) |
| Andorra | HAP | 4 | 2 | 2005 | 2010 | 0.004 | -22.30(-33.80,-8.70) |
| Andorra | HAP | 4 | 3 | 2010 | 2018 | 0.534 | -2.00(-8.50,4.90) |
| Andorra | HAP | 4 | 4 | 2018 | 2021 | 0.001 | -36.60(-50.90,-18.20) |
| Andorra | PMP | 4 | 0 | 1990 | 2002 | 0.000 | -7.50(-8.30,-6.70) |
| Andorra | PMP | 4 | 1 | 2002 | 2005 | 0.251 | 8.30(-5.90,24.60) |
| Andorra | PMP | 4 | 2 | 2005 | 2011 | 0.000 | -11.40(-14.20,-8.60) |
| Andorra | PMP | 4 | 3 | 2011 | 2019 | 0.000 | -6.30(-8.00,-4.50) |
| Andorra | PMP | 4 | 4 | 2019 | 2021 | 0.000 | -28.40(-37.80,-17.60) |
| Angola | APMP | 4 | 0 | 1990 | 1994 | 0.000 | -2.90(-3.40,-2.40) |
| Angola | APMP | 4 | 1 | 1994 | 2000 | 0.000 | 0.80(0.40,1.20) |
| Angola | APMP | 4 | 2 | 2000 | 2005 | 0.994 | 0.00(-0.50,0.50) |
| Angola | APMP | 4 | 3 | 2005 | 2014 | 0.000 | 6.10(5.90,6.30) |
| Angola | APMP | 4 | 4 | 2014 | 2021 | 0.000 | -2.50(-2.70,-2.30) |
| Angola | HAP | 4 | 0 | 1990 | 1998 | 0.000 | -1.30(-1.70,-1.00) |
| Angola | HAP | 4 | 1 | 1998 | 2005 | 0.000 | -4.40(-5.00,-3.90) |
| Angola | HAP | 4 | 2 | 2005 | 2010 | 0.000 | -7.00(-7.90,-6.00) |
| Angola | HAP | 4 | 3 | 2010 | 2015 | 0.000 | -9.30(-10.20,-8.30) |
| Angola | HAP | 4 | 4 | 2015 | 2021 | 0.000 | -5.20(-5.70,-4.70) |
| Angola | PMP | 2 | 0 | 1990 | 1997 | 0.000 | -1.20(-1.50,-1.00) |
| Angola | PMP | 2 | 1 | 1997 | 2001 | 0.000 | -2.80(-3.60,-2.00) |
| Angola | PMP | 2 | 2 | 2001 | 2021 | 0.000 | -4.30(-4.40,-4.30) |
| Antigua and Barbuda | APMP | 4 | 0 | 1990 | 1995 | 0.000 | 3.60(2.60,4.60) |
| Antigua and Barbuda | APMP | 4 | 1 | 1995 | 2001 | 0.074 | -0.90(-1.80,0.10) |
| Antigua and Barbuda | APMP | 4 | 2 | 2001 | 2010 | 0.000 | -4.10(-4.60,-3.70) |
| Antigua and Barbuda | APMP | 4 | 3 | 2010 | 2018 | 0.181 | 0.40(-0.20,1.00) |
| Antigua and Barbuda | APMP | 4 | 4 | 2018 | 2021 | 0.000 | -5.70(-7.70,-3.70) |
| Antigua and Barbuda | HAP | 3 | 0 | 1990 | 2005 | 0.000 | -8.00(-8.40,-7.50) |
| Antigua and Barbuda | HAP | 3 | 1 | 2005 | 2010 | 0.000 | -14.40(-17.40,-11.30) |
| Antigua and Barbuda | HAP | 3 | 2 | 2010 | 2016 | 0.002 | -4.20(-6.60,-1.80) |
| Antigua and Barbuda | HAP | 3 | 3 | 2016 | 2021 | 0.000 | -10.60(-12.80,-8.30) |
| Antigua and Barbuda | PMP | 4 | 0 | 1990 | 1995 | 0.000 | 3.00(2.00,4.10) |
| Antigua and Barbuda | PMP | 4 | 1 | 1995 | 2001 | 0.051 | -1.00(-1.90,0.00) |
| Antigua and Barbuda | PMP | 4 | 2 | 2001 | 2010 | 0.000 | -4.20(-4.70,-3.80) |
| Antigua and Barbuda | PMP | 4 | 3 | 2010 | 2018 | 0.250 | 0.30(-0.30,0.90) |
| Antigua and Barbuda | PMP | 4 | 4 | 2018 | 2021 | 0.000 | -5.80(-7.80,-3.70) |
| Argentina | APMP | 2 | 0 | 1990 | 1994 | 0.762 | -0.50(-3.50,2.70) |
| Argentina | APMP | 2 | 1 | 1994 | 2010 | 0.000 | -6.20(-6.50,-5.80) |
| Argentina | APMP | 2 | 2 | 2010 | 2021 | 0.000 | -2.00(-2.70,-1.30) |
| Argentina | HAP | 4 | 0 | 1990 | 1995 | 0.000 | -10.50(-12.30,-8.80) |
| Argentina | HAP | 4 | 1 | 1995 | 2000 | 0.000 | -16.20(-18.50,-13.90) |
| Argentina | HAP | 4 | 2 | 2000 | 2004 | 0.017 | -5.30(-9.40,-1.10) |
| Argentina | HAP | 4 | 3 | 2004 | 2015 | 0.000 | -14.10(-14.70,-13.50) |
| Argentina | HAP | 4 | 4 | 2015 | 2021 | 0.047 | -1.50(-2.90,0.00) |
| Argentina | PMP | 2 | 0 | 1990 | 1994 | 0.176 | -2.10(-5.20,1.00) |
| Argentina | PMP | 2 | 1 | 1994 | 2010 | 0.000 | -6.60(-7.00,-6.20) |
| Argentina | PMP | 2 | 2 | 2010 | 2021 | 0.000 | -2.20(-2.80,-1.50) |
| Armenia | APMP | 5 | 0 | 1990 | 1997 | 0.000 | -3.80(-4.70,-3.00) |
| Armenia | APMP | 5 | 1 | 1997 | 2001 | 0.000 | -6.60(-9.70,-3.50) |
| Armenia | APMP | 5 | 2 | 2001 | 2009 | 0.000 | 4.00(3.10,5.00) |
| Armenia | APMP | 5 | 3 | 2009 | 2014 | 0.004 | -3.30(-5.30,-1.20) |
| Armenia | APMP | 5 | 4 | 2014 | 2017 | 0.708 | 1.20(-5.30,8.10) |
| Armenia | APMP | 5 | 5 | 2017 | 2021 | 0.000 | -7.60(-9.50,-5.60) |
| Armenia | HAP | 5 | 0 | 1990 | 1997 | 0.000 | 4.30(3.40,5.20) |
| Armenia | HAP | 5 | 1 | 1997 | 2001 | 0.001 | -6.00(-9.00,-2.90) |
| Armenia | HAP | 5 | 2 | 2001 | 2006 | 0.000 | -13.80(-15.60,-12.10) |
| Armenia | HAP | 5 | 3 | 2006 | 2010 | 0.000 | -24.10(-26.50,-21.50) |
| Armenia | HAP | 5 | 4 | 2010 | 2014 | 0.000 | -20.80(-23.30,-18.20) |
| Armenia | HAP | 5 | 5 | 2014 | 2021 | 0.000 | -9.60(-10.40,-8.80) |
| Armenia | PMP | 4 | 0 | 1990 | 1997 | 0.495 | 0.30(-0.50,1.00) |
| Armenia | PMP | 4 | 1 | 1997 | 2002 | 0.000 | -6.20(-7.90,-4.40) |
| Armenia | PMP | 4 | 2 | 2002 | 2014 | 0.000 | -5.10(-5.40,-4.70) |
| Armenia | PMP | 4 | 3 | 2014 | 2017 | 0.887 | 0.40(-5.30,6.50) |
| Armenia | PMP | 4 | 4 | 2017 | 2021 | 0.000 | -7.80(-9.40,-6.00) |
| Australia | APMP | 4 | 0 | 1990 | 1998 | 0.000 | -5.30(-6.30,-4.30) |
| Australia | APMP | 4 | 1 | 1998 | 2005 | 0.581 | -0.40(-2.00,1.20) |
| Australia | APMP | 4 | 2 | 2005 | 2014 | 0.000 | -3.20(-4.20,-2.20) |
| Australia | APMP | 4 | 3 | 2014 | 2019 | 0.010 | 4.20(1.10,7.30) |
| Australia | APMP | 4 | 4 | 2019 | 2021 | 0.009 | -12.30(-20.20,-3.70) |
| Australia | HAP | 4 | 0 | 1990 | 2000 | 0.000 | -12.30(-13.50,-11.10) |
| Australia | HAP | 4 | 1 | 2000 | 2007 | 0.000 | -17.00(-19.40,-14.40) |
| Australia | HAP | 4 | 2 | 2007 | 2012 | 0.005 | -8.10(-13.20,-2.80) |
| Australia | HAP | 4 | 3 | 2012 | 2015 | 0.014 | -20.80(-33.80,-5.20) |
| Australia | HAP | 4 | 4 | 2015 | 2021 | 0.051 | -3.00(-5.90,0.00) |
| Australia | PMP | 4 | 0 | 1990 | 1998 | 0.000 | -5.40(-6.40,-4.40) |
| Australia | PMP | 4 | 1 | 1998 | 2005 | 0.496 | -0.50(-2.10,1.10) |
| Australia | PMP | 4 | 2 | 2005 | 2014 | 0.000 | -3.20(-4.20,-2.20) |
| Australia | PMP | 4 | 3 | 2014 | 2019 | 0.010 | 4.20(1.10,7.30) |
| Australia | PMP | 4 | 4 | 2019 | 2021 | 0.009 | -12.30(-20.20,-3.60) |
| Austria | APMP | 4 | 0 | 1990 | 1994 | 0.005 | -4.10(-6.80,-1.40) |
| Austria | APMP | 4 | 1 | 1994 | 1999 | 0.000 | -8.80(-11.40,-6.20) |
| Austria | APMP | 4 | 2 | 1999 | 2004 | 0.279 | 1.50(-1.30,4.40) |
| Austria | APMP | 4 | 3 | 2004 | 2013 | 0.000 | -7.30(-8.10,-6.40) |
| Austria | APMP | 4 | 4 | 2013 | 2021 | 0.000 | -4.30(-5.20,-3.40) |
| Austria | HAP | 3 | 0 | 1990 | 1998 | 0.000 | -15.40(-17.10,-13.70) |
| Austria | HAP | 3 | 1 | 1998 | 2001 | 0.620 | -4.50(-20.90,15.40) |
| Austria | HAP | 3 | 2 | 2001 | 2015 | 0.000 | -11.30(-12.10,-10.40) |
| Austria | HAP | 3 | 3 | 2015 | 2021 | 0.428 | -1.20(-4.30,2.00) |
| Austria | PMP | 4 | 0 | 1990 | 1994 | 0.005 | -4.10(-6.80,-1.40) |
| Austria | PMP | 4 | 1 | 1994 | 1999 | 0.000 | -8.80(-11.40,-6.30) |
| Austria | PMP | 4 | 2 | 1999 | 2004 | 0.281 | 1.50(-1.30,4.40) |
| Austria | PMP | 4 | 3 | 2004 | 2013 | 0.000 | -7.30(-8.10,-6.40) |
| Austria | PMP | 4 | 4 | 2013 | 2021 | 0.000 | -4.30(-5.20,-3.40) |
| Azerbaijan | APMP | 5 | 0 | 1990 | 1996 | 0.000 | -3.00(-3.40,-2.50) |
| Azerbaijan | APMP | 5 | 1 | 1996 | 2000 | 0.000 | -5.30(-6.60,-4.00) |
| Azerbaijan | APMP | 5 | 2 | 2000 | 2005 | 0.000 | 7.60(6.70,8.50) |
| Azerbaijan | APMP | 5 | 3 | 2005 | 2009 | 0.000 | 13.00(11.50,14.60) |
| Azerbaijan | APMP | 5 | 4 | 2009 | 2014 | 0.023 | -1.00(-1.80,-0.20) |
| Azerbaijan | APMP | 5 | 5 | 2014 | 2021 | 0.000 | -3.90(-4.20,-3.50) |
| Azerbaijan | HAP | 5 | 0 | 1990 | 2000 | 0.000 | 6.30(6.00,6.60) |
| Azerbaijan | HAP | 5 | 1 | 2000 | 2004 | 0.000 | -5.50(-7.30,-3.60) |
| Azerbaijan | HAP | 5 | 2 | 2004 | 2007 | 0.000 | -18.20(-21.30,-15.00) |
| Azerbaijan | HAP | 5 | 3 | 2007 | 2010 | 0.000 | -31.30(-33.90,-28.60) |
| Azerbaijan | HAP | 5 | 4 | 2010 | 2015 | 0.000 | -23.30(-24.20,-22.40) |
| Azerbaijan | HAP | 5 | 5 | 2015 | 2021 | 0.000 | -4.60(-5.20,-3.90) |
| Azerbaijan | PMP | 5 | 0 | 1990 | 1993 | 0.000 | 2.10(1.20,3.00) |
| Azerbaijan | PMP | 5 | 1 | 1993 | 1997 | 0.000 | 3.30(2.40,4.30) |
| Azerbaijan | PMP | 5 | 2 | 1997 | 2000 | 0.082 | 1.60(-0.20,3.40) |
| Azerbaijan | PMP | 5 | 3 | 2000 | 2004 | 0.000 | -2.30(-3.10,-1.40) |
| Azerbaijan | PMP | 5 | 4 | 2004 | 2019 | 0.000 | -4.20(-4.30,-4.10) |
| Azerbaijan | PMP | 5 | 5 | 2019 | 2021 | 0.002 | -3.10(-4.80,-1.40) |
| Bahamas | APMP | 3 | 0 | 1990 | 1994 | 0.000 | -6.70(-7.80,-5.60) |
| Bahamas | APMP | 3 | 1 | 1994 | 2009 | 0.000 | -2.40(-2.60,-2.20) |
| Bahamas | APMP | 3 | 2 | 2009 | 2014 | 0.352 | 0.50(-0.60,1.70) |
| Bahamas | APMP | 3 | 3 | 2014 | 2021 | 0.000 | -4.30(-4.80,-3.90) |
| Bahamas | HAP | 5 | 0 | 1990 | 1996 | 0.000 | -7.50(-8.20,-6.80) |
| Bahamas | HAP | 5 | 1 | 1996 | 2003 | 0.000 | -11.30(-12.00,-10.60) |
| Bahamas | HAP | 5 | 2 | 2003 | 2010 | 0.000 | -6.10(-6.80,-5.40) |
| Bahamas | HAP | 5 | 3 | 2010 | 2015 | 0.000 | 3.10(1.60,4.60) |
| Bahamas | HAP | 5 | 4 | 2015 | 2019 | 0.000 | -10.20(-12.20,-8.10) |
| Bahamas | HAP | 5 | 5 | 2019 | 2021 | 0.019 | -5.40(-9.60,-1.00) |
| Bahamas | PMP | 3 | 0 | 1990 | 1994 | 0.000 | -6.80(-7.90,-5.70) |
| Bahamas | PMP | 3 | 1 | 1994 | 2009 | 0.000 | -2.40(-2.60,-2.30) |
| Bahamas | PMP | 3 | 2 | 2009 | 2014 | 0.336 | 0.60(-0.60,1.80) |
| Bahamas | PMP | 3 | 3 | 2014 | 2021 | 0.000 | -4.30(-4.80,-3.90) |
| Bahrain | APMP | 5 | 0 | 1990 | 1993 | 0.004 | -4.70(-7.60,-1.70) |
| Bahrain | APMP | 5 | 1 | 1993 | 1998 | 0.000 | -12.40(-14.10,-10.70) |
| Bahrain | APMP | 5 | 2 | 1998 | 2006 | 0.000 | -3.90(-4.70,-3.10) |
| Bahrain | APMP | 5 | 3 | 2006 | 2010 | 0.000 | -7.20(-10.00,-4.30) |
| Bahrain | APMP | 5 | 4 | 2010 | 2016 | 0.003 | 2.30(0.90,3.70) |
| Bahrain | APMP | 5 | 5 | 2016 | 2021 | 0.000 | -8.90(-10.10,-7.60) |
| Bahrain | HAP | 3 | 0 | 1990 | 1993 | 0.000 | -11.00(-16.00,-5.80) |
| Bahrain | HAP | 3 | 1 | 1993 | 1998 | 0.000 | -21.30(-24.10,-18.40) |
| Bahrain | HAP | 3 | 2 | 1998 | 2005 | 0.000 | -18.70(-20.20,-17.10) |
| Bahrain | HAP | 3 | 3 | 2005 | 2021 | 0.000 | -11.10(-11.50,-10.70) |
| Bahrain | PMP | 5 | 0 | 1990 | 1993 | 0.004 | -4.80(-7.70,-1.80) |
| Bahrain | PMP | 5 | 1 | 1993 | 1998 | 0.000 | -12.40(-14.10,-10.70) |
| Bahrain | PMP | 5 | 2 | 1998 | 2006 | 0.000 | -4.00(-4.80,-3.20) |
| Bahrain | PMP | 5 | 3 | 2006 | 2010 | 0.000 | -7.20(-10.00,-4.30) |
| Bahrain | PMP | 5 | 4 | 2010 | 2016 | 0.003 | 2.30(0.90,3.70) |
| Bahrain | PMP | 5 | 5 | 2016 | 2021 | 0.000 | -8.90(-10.10,-7.60) |
| Bangladesh | APMP | 3 | 0 | 1990 | 2006 | 0.000 | -1.60(-1.80,-1.30) |
| Bangladesh | APMP | 3 | 1 | 2006 | 2010 | 0.002 | -5.80(-9.00,-2.50) |
| Bangladesh | APMP | 3 | 2 | 2010 | 2015 | 0.000 | 9.50(7.20,12.00) |
| Bangladesh | APMP | 3 | 3 | 2015 | 2021 | 0.000 | -6.50(-7.50,-5.40) |
| Bangladesh | HAP | 5 | 0 | 1990 | 2002 | 0.000 | -2.50(-2.60,-2.30) |
| Bangladesh | HAP | 5 | 1 | 2002 | 2007 | 0.006 | -1.10(-1.80,-0.40) |
| Bangladesh | HAP | 5 | 2 | 2007 | 2011 | 0.001 | -2.30(-3.50,-1.20) |
| Bangladesh | HAP | 5 | 3 | 2011 | 2014 | 0.000 | -6.20(-8.40,-4.00) |
| Bangladesh | HAP | 5 | 4 | 2014 | 2017 | 0.002 | -4.00(-6.20,-1.80) |
| Bangladesh | HAP | 5 | 5 | 2017 | 2021 | 0.000 | -6.70(-7.40,-6.00) |
| Bangladesh | PMP | 5 | 0 | 1990 | 1993 | 0.001 | -1.50(-2.30,-0.70) |
| Bangladesh | PMP | 5 | 1 | 1993 | 2001 | 0.000 | -2.70(-2.90,-2.50) |
| Bangladesh | PMP | 5 | 2 | 2001 | 2006 | 0.000 | -1.10(-1.60,-0.60) |
| Bangladesh | PMP | 5 | 3 | 2006 | 2011 | 0.000 | -2.50(-2.90,-2.00) |
| Bangladesh | PMP | 5 | 4 | 2011 | 2017 | 0.000 | -3.90(-4.30,-3.60) |
| Bangladesh | PMP | 5 | 5 | 2017 | 2021 | 0.000 | -6.80(-7.20,-6.30) |
| Barbados | APMP | 0 | 0 | 1990 | 2021 | 0.000 | -1.00(-1.30,-0.70) |
| Barbados | HAP | 1 | 0 | 1990 | 2001 | 0.000 | -10.30(-11.80,-8.70) |
| Barbados | HAP | 1 | 1 | 2001 | 2021 | 0.000 | -2.50(-3.20,-1.80) |
| Barbados | PMP | 0 | 0 | 1990 | 2021 | 0.000 | -1.00(-1.30,-0.70) |
| Belarus | APMP | 3 | 0 | 1990 | 1999 | 0.000 | -2.50(-3.50,-1.30) |
| Belarus | APMP | 3 | 1 | 1999 | 2004 | 0.000 | -12.00(-15.40,-8.50) |
| Belarus | APMP | 3 | 2 | 2004 | 2007 | 0.751 | -1.90(-13.30,11.00) |
| Belarus | APMP | 3 | 3 | 2007 | 2021 | 0.000 | -9.70(-10.20,-9.10) |
| Belarus | HAP | 3 | 0 | 1990 | 1999 | 0.003 | 2.20(0.80,3.50) |
| Belarus | HAP | 3 | 1 | 1999 | 2006 | 0.000 | -13.90(-15.90,-11.70) |
| Belarus | HAP | 3 | 2 | 2006 | 2013 | 0.000 | -28.10(-29.80,-26.40) |
| Belarus | HAP | 3 | 3 | 2013 | 2021 | 0.000 | -13.70(-15.10,-12.40) |
| Belarus | PMP | 3 | 0 | 1990 | 1999 | 0.000 | -2.20(-3.30,-1.10) |
| Belarus | PMP | 3 | 1 | 1999 | 2004 | 0.000 | -12.00(-15.40,-8.50) |
| Belarus | PMP | 3 | 2 | 2004 | 2007 | 0.589 | -3.20(-14.30,9.40) |
| Belarus | PMP | 3 | 3 | 2007 | 2021 | 0.000 | -9.90(-10.40,-9.30) |
| Belgium | APMP | 4 | 0 | 1990 | 1993 | 0.284 | 2.10(-1.80,6.10) |
| Belgium | APMP | 4 | 1 | 1993 | 1996 | 0.005 | -11.10(-17.70,-3.90) |
| Belgium | APMP | 4 | 2 | 1996 | 2004 | 0.000 | -5.90(-6.90,-4.90) |
| Belgium | APMP | 4 | 3 | 2004 | 2007 | 0.986 | 0.10(-7.40,8.10) |
| Belgium | APMP | 4 | 4 | 2007 | 2021 | 0.000 | -4.40(-4.80,-4.10) |
| Belgium | HAP | 1 | 0 | 1990 | 2004 | 0.000 | -15.70(-16.60,-14.70) |
| Belgium | HAP | 1 | 1 | 2004 | 2021 | 0.000 | -6.90(-7.70,-6.10) |
| Belgium | PMP | 4 | 0 | 1990 | 1993 | 0.291 | 2.00(-1.90,6.10) |
| Belgium | PMP | 4 | 1 | 1993 | 1996 | 0.005 | -11.10(-17.70,-3.90) |
| Belgium | PMP | 4 | 2 | 1996 | 2004 | 0.000 | -5.90(-6.90,-4.90) |
| Belgium | PMP | 4 | 3 | 2004 | 2007 | 0.986 | 0.10(-7.40,8.10) |
| Belgium | PMP | 4 | 4 | 2007 | 2021 | 0.000 | -4.40(-4.80,-4.10) |
| Belize | APMP | 3 | 0 | 1990 | 2001 | 0.791 | -0.10(-0.60,0.50) |
| Belize | APMP | 3 | 1 | 2001 | 2010 | 0.000 | -4.30(-5.20,-3.40) |
| Belize | APMP | 3 | 2 | 2010 | 2015 | 0.027 | 3.10(0.40,5.90) |
| Belize | APMP | 3 | 3 | 2015 | 2021 | 0.003 | -2.30(-3.70,-0.90) |
| Belize | HAP | 3 | 0 | 1990 | 1994 | 0.000 | -8.60(-10.30,-6.80) |
| Belize | HAP | 3 | 1 | 1994 | 2000 | 0.000 | -3.80(-5.10,-2.50) |
| Belize | HAP | 3 | 2 | 2000 | 2009 | 0.000 | -6.60(-7.20,-6.00) |
| Belize | HAP | 3 | 3 | 2009 | 2021 | 0.000 | -5.00(-5.40,-4.70) |
| Belize | PMP | 3 | 0 | 1990 | 2003 | 0.000 | -2.80(-3.30,-2.40) |
| Belize | PMP | 3 | 1 | 2003 | 2010 | 0.000 | -5.50(-6.90,-4.10) |
| Belize | PMP | 3 | 2 | 2010 | 2015 | 0.470 | 1.00(-1.80,3.90) |
| Belize | PMP | 3 | 3 | 2015 | 2021 | 0.000 | -3.10(-4.60,-1.70) |
| Benin | APMP | 5 | 0 | 1990 | 1995 | 0.010 | -2.70(-4.60,-0.70) |
| Benin | APMP | 5 | 1 | 1995 | 2000 | 0.020 | 3.50(0.60,6.40) |
| Benin | APMP | 5 | 2 | 2000 | 2005 | 0.003 | -4.50(-7.10,-1.70) |
| Benin | APMP | 5 | 3 | 2005 | 2010 | 0.646 | 0.60(-2.20,3.50) |
| Benin | APMP | 5 | 4 | 2010 | 2015 | 0.001 | 5.90(2.90,8.90) |
| Benin | APMP | 5 | 5 | 2015 | 2021 | 0.000 | -7.30(-8.60,-5.90) |
| Benin | HAP | 5 | 0 | 1990 | 1994 | 0.084 | 0.50(-0.10,1.10) |
| Benin | HAP | 5 | 1 | 1994 | 2000 | 0.000 | -1.70(-2.10,-1.30) |
| Benin | HAP | 5 | 2 | 2000 | 2009 | 0.840 | 0.00(-0.20,0.20) |
| Benin | HAP | 5 | 3 | 2009 | 2015 | 0.000 | -2.30(-2.70,-1.90) |
| Benin | HAP | 5 | 4 | 2015 | 2018 | 0.767 | -0.30(-2.00,1.60) |
| Benin | HAP | 5 | 5 | 2018 | 2021 | 0.000 | -2.70(-3.60,-1.80) |
| Benin | PMP | 5 | 0 | 1990 | 1994 | 0.062 | -0.10(-0.20,0.00) |
| Benin | PMP | 5 | 1 | 1994 | 2001 | 0.000 | -1.10(-1.10,-1.00) |
| Benin | PMP | 5 | 2 | 2001 | 2008 | 0.000 | -0.40(-0.40,-0.30) |
| Benin | PMP | 5 | 3 | 2008 | 2012 | 0.000 | -0.70(-0.90,-0.50) |
| Benin | PMP | 5 | 4 | 2012 | 2017 | 0.000 | -1.30(-1.40,-1.20) |
| Benin | PMP | 5 | 5 | 2017 | 2021 | 0.000 | -2.80(-3.00,-2.70) |
| Bermuda | APMP | 3 | 0 | 1990 | 1994 | 0.000 | -8.10(-9.90,-6.30) |
| Bermuda | APMP | 3 | 1 | 1994 | 1998 | 0.000 | -12.00(-14.60,-9.30) |
| Bermuda | APMP | 3 | 2 | 1998 | 2016 | 0.000 | -2.70(-2.90,-2.50) |
| Bermuda | APMP | 3 | 3 | 2016 | 2021 | 0.000 | -5.20(-6.50,-3.90) |
| Bermuda | HAP | 5 | 0 | 1990 | 1998 | 0.000 | -16.20(-17.00,-15.30) |
| Bermuda | HAP | 5 | 1 | 1998 | 2007 | 0.000 | -11.10(-12.00,-10.20) |
| Bermuda | HAP | 5 | 2 | 2007 | 2010 | 0.000 | -25.40(-32.10,-18.00) |
| Bermuda | HAP | 5 | 3 | 2010 | 2015 | 0.000 | 6.60(3.50,9.90) |
| Bermuda | HAP | 5 | 4 | 2015 | 2019 | 0.000 | -18.60(-22.40,-14.70) |
| Bermuda | HAP | 5 | 5 | 2019 | 2021 | 0.091 | -7.70(-16.00,1.40) |
| Bermuda | PMP | 3 | 0 | 1990 | 1994 | 0.000 | -8.20(-10.00,-6.40) |
| Bermuda | PMP | 3 | 1 | 1994 | 1998 | 0.000 | -12.10(-14.70,-9.30) |
| Bermuda | PMP | 3 | 2 | 1998 | 2016 | 0.000 | -2.70(-2.90,-2.50) |
| Bermuda | PMP | 3 | 3 | 2016 | 2021 | 0.000 | -5.20(-6.50,-3.90) |
| Bhutan | APMP | 4 | 0 | 1990 | 1998 | 0.043 | 1.20(0.00,2.30) |
| Bhutan | APMP | 4 | 1 | 1998 | 2005 | 0.000 | 4.20(2.40,6.00) |
| Bhutan | APMP | 4 | 2 | 2005 | 2010 | 0.251 | -1.80(-5.00,1.40) |
| Bhutan | APMP | 4 | 3 | 2010 | 2015 | 0.000 | 8.60(5.10,12.30) |
| Bhutan | APMP | 4 | 4 | 2015 | 2021 | 0.000 | -6.30(-8.00,-4.70) |
| Bhutan | HAP | 5 | 0 | 1990 | 1993 | 0.002 | -1.50(-2.30,-0.60) |
| Bhutan | HAP | 5 | 1 | 1993 | 2001 | 0.000 | -4.00(-4.30,-3.80) |
| Bhutan | HAP | 5 | 2 | 2001 | 2006 | 0.000 | -7.90(-8.30,-7.40) |
| Bhutan | HAP | 5 | 3 | 2006 | 2009 | 0.000 | -11.10(-12.60,-9.70) |
| Bhutan | HAP | 5 | 4 | 2009 | 2019 | 0.000 | -13.70(-13.90,-13.60) |
| Bhutan | HAP | 5 | 5 | 2019 | 2021 | 0.013 | -2.20(-3.80,-0.50) |
| Bhutan | PMP | 3 | 0 | 1990 | 2003 | 0.000 | -3.00(-3.40,-2.50) |
| Bhutan | PMP | 3 | 1 | 2003 | 2010 | 0.000 | -7.80(-9.20,-6.30) |
| Bhutan | PMP | 3 | 2 | 2010 | 2016 | 0.001 | -3.60(-5.60,-1.60) |
| Bhutan | PMP | 3 | 3 | 2016 | 2021 | 0.000 | -7.90(-9.80,-6.00) |
| Bolivia (Plurinational State of) | APMP | 4 | 0 | 1990 | 1995 | 0.009 | -0.80(-1.40,-0.20) |
| Bolivia (Plurinational State of) | APMP | 4 | 1 | 1995 | 2006 | 0.000 | -2.90(-3.10,-2.70) |
| Bolivia (Plurinational State of) | APMP | 4 | 2 | 2006 | 2010 | 0.000 | -10.80(-11.90,-9.60) |
| Bolivia (Plurinational State of) | APMP | 4 | 3 | 2010 | 2017 | 0.036 | -0.50(-0.90,0.00) |
| Bolivia (Plurinational State of) | APMP | 4 | 4 | 2017 | 2021 | 0.000 | -4.10(-4.90,-3.30) |
| Bolivia (Plurinational State of) | HAP | 4 | 0 | 1990 | 1994 | 0.000 | -4.90(-5.50,-4.30) |
| Bolivia (Plurinational State of) | HAP | 4 | 1 | 1994 | 2000 | 0.000 | -3.50(-3.90,-3.10) |
| Bolivia (Plurinational State of) | HAP | 4 | 2 | 2000 | 2010 | 0.000 | -4.40(-4.60,-4.30) |
| Bolivia (Plurinational State of) | HAP | 4 | 3 | 2010 | 2019 | 0.000 | -9.30(-9.50,-9.10) |
| Bolivia (Plurinational State of) | HAP | 4 | 4 | 2019 | 2021 | 0.000 | -4.30(-6.10,-2.40) |
| Bolivia (Plurinational State of) | PMP | 2 | 0 | 1990 | 2005 | 0.000 | -3.20(-3.30,-3.10) |
| Bolivia (Plurinational State of) | PMP | 2 | 1 | 2005 | 2011 | 0.000 | -6.90(-7.50,-6.30) |
| Bolivia (Plurinational State of) | PMP | 2 | 2 | 2011 | 2021 | 0.000 | -5.00(-5.20,-4.80) |
| Bosnia and Herzegovina | APMP | 5 | 0 | 1990 | 1996 | 0.398 | 0.70(-1.00,2.50) |
| Bosnia and Herzegovina | APMP | 5 | 1 | 1996 | 1999 | 0.154 | 7.50(-3.00,19.20) |
| Bosnia and Herzegovina | APMP | 5 | 2 | 1999 | 2007 | 0.261 | 0.80(-0.60,2.20) |
| Bosnia and Herzegovina | APMP | 5 | 3 | 2007 | 2014 | 0.002 | -3.10(-4.80,-1.40) |
| Bosnia and Herzegovina | APMP | 5 | 4 | 2014 | 2019 | 0.066 | 3.10(-0.20,6.50) |
| Bosnia and Herzegovina | APMP | 5 | 5 | 2019 | 2021 | 0.016 | -12.30(-20.90,-2.80) |
| Bosnia and Herzegovina | HAP | 4 | 0 | 1990 | 1995 | 0.640 | 0.50(-1.70,2.80) |
| Bosnia and Herzegovina | HAP | 4 | 1 | 1995 | 2004 | 0.000 | -14.20(-15.10,-13.20) |
| Bosnia and Herzegovina | HAP | 4 | 2 | 2004 | 2014 | 0.000 | -11.20(-12.00,-10.40) |
| Bosnia and Herzegovina | HAP | 4 | 3 | 2014 | 2018 | 0.555 | -1.40(-6.30,3.60) |
| Bosnia and Herzegovina | HAP | 4 | 4 | 2018 | 2021 | 0.001 | -9.30(-13.80,-4.60) |
| Bosnia and Herzegovina | PMP | 4 | 0 | 1990 | 1995 | 0.596 | 0.50(-1.50,2.50) |
| Bosnia and Herzegovina | PMP | 4 | 1 | 1995 | 2001 | 0.000 | -11.00(-12.70,-9.20) |
| Bosnia and Herzegovina | PMP | 4 | 2 | 2001 | 2014 | 0.000 | -6.60(-7.10,-6.10) |
| Bosnia and Herzegovina | PMP | 4 | 3 | 2014 | 2018 | 0.170 | 3.00(-1.40,7.70) |
| Bosnia and Herzegovina | PMP | 4 | 4 | 2018 | 2021 | 0.001 | -8.40(-12.30,-4.30) |
| Botswana | APMP | 5 | 0 | 1990 | 1993 | 0.000 | 6.10(4.60,7.60) |
| Botswana | APMP | 5 | 1 | 1993 | 1999 | 0.000 | 3.30(2.70,4.00) |
| Botswana | APMP | 5 | 2 | 1999 | 2005 | 0.000 | 4.80(4.10,5.40) |
| Botswana | APMP | 5 | 3 | 2005 | 2010 | 0.224 | 0.50(-0.40,1.40) |
| Botswana | APMP | 5 | 4 | 2010 | 2015 | 0.000 | -2.00(-2.80,-1.10) |
| Botswana | APMP | 5 | 5 | 2015 | 2021 | 0.000 | -3.10(-3.60,-2.70) |
| Botswana | HAP | 5 | 0 | 1990 | 1993 | 0.000 | -5.80(-6.30,-5.40) |
| Botswana | HAP | 5 | 1 | 1993 | 1999 | 0.000 | -7.10(-7.30,-6.90) |
| Botswana | HAP | 5 | 2 | 1999 | 2005 | 0.000 | -5.20(-5.40,-5.00) |
| Botswana | HAP | 5 | 3 | 2005 | 2010 | 0.000 | -8.80(-9.10,-8.50) |
| Botswana | HAP | 5 | 4 | 2010 | 2019 | 0.000 | -9.90(-10.00,-9.80) |
| Botswana | HAP | 5 | 5 | 2019 | 2021 | 0.344 | -0.40(-1.40,0.50) |
| Botswana | PMP | 5 | 0 | 1990 | 1999 | 0.000 | -3.40(-3.50,-3.30) |
| Botswana | PMP | 5 | 1 | 1999 | 2005 | 0.053 | -0.30(-0.50,0.00) |
| Botswana | PMP | 5 | 2 | 2005 | 2010 | 0.000 | -2.90(-3.20,-2.50) |
| Botswana | PMP | 5 | 3 | 2010 | 2015 | 0.000 | -4.00(-4.40,-3.60) |
| Botswana | PMP | 5 | 4 | 2015 | 2019 | 0.000 | -5.00(-5.60,-4.50) |
| Botswana | PMP | 5 | 5 | 2019 | 2021 | 0.098 | -1.00(-2.20,0.20) |
| Brazil | APMP | 5 | 0 | 1990 | 1993 | 0.000 | -2.90(-4.00,-1.70) |
| Brazil | APMP | 5 | 1 | 1993 | 2000 | 0.000 | -1.30(-1.70,-0.90) |
| Brazil | APMP | 5 | 2 | 2000 | 2005 | 0.000 | -3.50(-4.30,-2.80) |
| Brazil | APMP | 5 | 3 | 2005 | 2010 | 0.000 | -2.70(-3.40,-2.00) |
| Brazil | APMP | 5 | 4 | 2010 | 2015 | 0.000 | -7.90(-8.60,-7.20) |
| Brazil | APMP | 5 | 5 | 2015 | 2021 | 0.000 | -5.00(-5.30,-4.60) |
| Brazil | HAP | 4 | 0 | 1990 | 1995 | 0.000 | -8.00(-9.10,-6.90) |
| Brazil | HAP | 4 | 1 | 1995 | 2005 | 0.000 | -6.70(-7.20,-6.20) |
| Brazil | HAP | 4 | 2 | 2005 | 2010 | 0.000 | -10.50(-12.10,-8.90) |
| Brazil | HAP | 4 | 3 | 2010 | 2015 | 0.000 | -14.50(-16.00,-13.00) |
| Brazil | HAP | 4 | 4 | 2015 | 2021 | 0.000 | -10.70(-11.50,-9.80) |
| Brazil | PMP | 4 | 0 | 1990 | 1994 | 0.000 | -6.20(-7.10,-5.40) |
| Brazil | PMP | 4 | 1 | 1994 | 2002 | 0.000 | -4.40(-4.80,-4.10) |
| Brazil | PMP | 4 | 2 | 2002 | 2010 | 0.000 | -5.90(-6.30,-5.60) |
| Brazil | PMP | 4 | 3 | 2010 | 2015 | 0.000 | -10.30(-11.10,-9.40) |
| Brazil | PMP | 4 | 4 | 2015 | 2021 | 0.000 | -6.50(-6.90,-6.00) |
| Brunei Darussalam | APMP | 4 | 0 | 1990 | 1999 | 0.000 | -3.00(-3.70,-2.40) |
| Brunei Darussalam | APMP | 4 | 1 | 1999 | 2003 | 0.137 | 2.80(-1.00,6.70) |
| Brunei Darussalam | APMP | 4 | 2 | 2003 | 2010 | 0.000 | -3.00(-4.30,-1.80) |
| Brunei Darussalam | APMP | 4 | 3 | 2010 | 2018 | 0.000 | 5.80(4.70,6.80) |
| Brunei Darussalam | APMP | 4 | 4 | 2018 | 2021 | 0.016 | -4.60(-8.10,-1.00) |
| Brunei Darussalam | HAP | 5 | 0 | 1990 | 1994 | 0.260 | 4.70(-3.70,13.90) |
| Brunei Darussalam | HAP | 5 | 1 | 1994 | 1999 | 0.005 | -12.10(-19.20,-4.30) |
| Brunei Darussalam | HAP | 5 | 2 | 1999 | 2006 | 0.154 | -3.10(-7.40,1.30) |
| Brunei Darussalam | HAP | 5 | 3 | 2006 | 2009 | 0.199 | -15.50(-35.20,10.30) |
| Brunei Darussalam | HAP | 5 | 4 | 2009 | 2013 | 0.244 | 7.90(-5.60,23.20) |
| Brunei Darussalam | HAP | 5 | 5 | 2013 | 2021 | 0.482 | -1.00(-3.80,1.90) |
| Brunei Darussalam | PMP | 4 | 0 | 1990 | 1999 | 0.000 | -3.00(-3.70,-2.40) |
| Brunei Darussalam | PMP | 4 | 1 | 1999 | 2003 | 0.142 | 2.80(-1.00,6.70) |
| Brunei Darussalam | PMP | 4 | 2 | 2003 | 2010 | 0.000 | -3.10(-4.30,-1.80) |
| Brunei Darussalam | PMP | 4 | 3 | 2010 | 2018 | 0.000 | 5.70(4.70,6.80) |
| Brunei Darussalam | PMP | 4 | 4 | 2018 | 2021 | 0.016 | -4.60(-8.10,-1.00) |
| Bulgaria | APMP | 2 | 0 | 1990 | 1992 | 0.475 | 5.20(-9.00,21.70) |
| Bulgaria | APMP | 2 | 1 | 1992 | 2012 | 0.000 | -2.40(-2.90,-2.00) |
| Bulgaria | APMP | 2 | 2 | 2012 | 2021 | 0.000 | -8.30(-9.50,-7.10) |
| Bulgaria | HAP | 3 | 0 | 1990 | 2000 | 0.000 | 3.10(2.00,4.20) |
| Bulgaria | HAP | 3 | 1 | 2000 | 2010 | 0.000 | -10.80(-11.90,-9.70) |
| Bulgaria | HAP | 3 | 2 | 2010 | 2019 | 0.000 | -15.20(-16.50,-14.00) |
| Bulgaria | HAP | 3 | 3 | 2019 | 2021 | 0.444 | -5.00(-17.10,8.90) |
| Bulgaria | PMP | 2 | 0 | 1990 | 1997 | 0.537 | 0.50(-1.20,2.40) |
| Bulgaria | PMP | 2 | 1 | 1997 | 2011 | 0.000 | -3.70(-4.40,-3.00) |
| Bulgaria | PMP | 2 | 2 | 2011 | 2021 | 0.000 | -8.50(-9.50,-7.60) |
| Burkina Faso | APMP | 4 | 0 | 1990 | 1994 | 0.015 | -3.70(-6.50,-0.80) |
| Burkina Faso | APMP | 4 | 1 | 1994 | 2011 | 0.000 | 1.20(0.80,1.60) |
| Burkina Faso | APMP | 4 | 2 | 2011 | 2015 | 0.080 | 4.20(-0.50,9.10) |
| Burkina Faso | APMP | 4 | 3 | 2015 | 2018 | 0.065 | -8.30(-16.40,0.60) |
| Burkina Faso | APMP | 4 | 4 | 2018 | 2021 | 0.323 | -2.20(-6.70,2.40) |
| Burkina Faso | HAP | 2 | 0 | 1990 | 2001 | 0.000 | -1.70(-1.80,-1.50) |
| Burkina Faso | HAP | 2 | 1 | 2001 | 2007 | 0.000 | 1.70(1.20,2.20) |
| Burkina Faso | HAP | 2 | 2 | 2007 | 2021 | 0.000 | -1.40(-1.50,-1.30) |
| Burkina Faso | PMP | 4 | 0 | 1990 | 1993 | 0.000 | -2.40(-2.70,-2.00) |
| Burkina Faso | PMP | 4 | 1 | 1993 | 2002 | 0.000 | -1.20(-1.20,-1.10) |
| Burkina Faso | PMP | 4 | 2 | 2002 | 2006 | 0.000 | 2.50(2.10,2.90) |
| Burkina Faso | PMP | 4 | 3 | 2006 | 2015 | 0.000 | -0.80(-0.80,-0.70) |
| Burkina Faso | PMP | 4 | 4 | 2015 | 2021 | 0.000 | -1.90(-2.10,-1.80) |
| Burundi | APMP | 5 | 0 | 1990 | 1993 | 0.272 | 0.60(-0.50,1.60) |
| Burundi | APMP | 5 | 1 | 1993 | 1997 | 0.077 | -0.90(-1.90,0.10) |
| Burundi | APMP | 5 | 2 | 1997 | 2004 | 0.000 | -2.60(-3.00,-2.30) |
| Burundi | APMP | 5 | 3 | 2004 | 2011 | 0.000 | -1.10(-1.50,-0.80) |
| Burundi | APMP | 5 | 4 | 2011 | 2016 | 0.000 | -2.30(-3.00,-1.70) |
| Burundi | APMP | 5 | 5 | 2016 | 2021 | 0.000 | -3.50(-3.90,-3.00) |
| Burundi | HAP | 3 | 0 | 1990 | 1994 | 0.015 | 0.90(0.20,1.60) |
| Burundi | HAP | 3 | 1 | 1994 | 2008 | 0.000 | -0.70(-0.80,-0.60) |
| Burundi | HAP | 3 | 2 | 2008 | 2016 | 0.000 | -2.00(-2.20,-1.70) |
| Burundi | HAP | 3 | 3 | 2016 | 2021 | 0.000 | -3.30(-3.80,-2.80) |
| Burundi | PMP | 3 | 0 | 1990 | 1994 | 0.010 | 0.90(0.20,1.60) |
| Burundi | PMP | 3 | 1 | 1994 | 2009 | 0.000 | -0.80(-0.90,-0.70) |
| Burundi | PMP | 3 | 2 | 2009 | 2016 | 0.000 | -2.10(-2.40,-1.70) |
| Burundi | PMP | 3 | 3 | 2016 | 2021 | 0.000 | -3.30(-3.70,-2.80) |
| Cabo Verde | APMP | 5 | 0 | 1990 | 1995 | 0.000 | 2.60(1.30,3.90) |
| Cabo Verde | APMP | 5 | 1 | 1995 | 1999 | 0.000 | 11.60(8.60,14.70) |
| Cabo Verde | APMP | 5 | 2 | 1999 | 2005 | 0.047 | 1.30(0.00,2.50) |
| Cabo Verde | APMP | 5 | 3 | 2005 | 2010 | 0.000 | -3.70(-5.40,-2.00) |
| Cabo Verde | APMP | 5 | 4 | 2010 | 2014 | 0.000 | 15.30(12.20,18.50) |
| Cabo Verde | APMP | 5 | 5 | 2014 | 2021 | 0.000 | -5.70(-6.40,-5.00) |
| Cabo Verde | HAP | 2 | 0 | 1990 | 1999 | 0.001 | -1.20(-1.80,-0.60) |
| Cabo Verde | HAP | 2 | 1 | 1999 | 2006 | 0.000 | -4.50(-5.60,-3.30) |
| Cabo Verde | HAP | 2 | 2 | 2006 | 2021 | 0.000 | -9.70(-10.00,-9.50) |
| Cabo Verde | PMP | 5 | 0 | 1990 | 1992 | 0.078 | -2.80(-5.80,0.40) |
| Cabo Verde | PMP | 5 | 1 | 1992 | 2000 | 0.005 | 0.70(0.20,1.10) |
| Cabo Verde | PMP | 5 | 2 | 2000 | 2004 | 0.005 | -2.40(-4.00,-0.90) |
| Cabo Verde | PMP | 5 | 3 | 2004 | 2010 | 0.000 | -6.10(-6.80,-5.50) |
| Cabo Verde | PMP | 5 | 4 | 2010 | 2015 | 0.087 | 0.90(-0.10,1.90) |
| Cabo Verde | PMP | 5 | 5 | 2015 | 2021 | 0.000 | -7.50(-8.00,-7.00) |
| Cambodia | APMP | 3 | 0 | 1990 | 1995 | 0.013 | -2.10(-3.60,-0.50) |
| Cambodia | APMP | 3 | 1 | 1995 | 2005 | 0.804 | 0.10(-0.60,0.70) |
| Cambodia | APMP | 3 | 2 | 2005 | 2014 | 0.000 | -2.50(-3.30,-1.80) |
| Cambodia | APMP | 3 | 3 | 2014 | 2021 | 0.000 | 3.50(2.50,4.50) |
| Cambodia | HAP | 5 | 0 | 1990 | 1999 | 0.000 | -1.20(-1.30,-1.10) |
| Cambodia | HAP | 5 | 1 | 1999 | 2002 | 0.001 | -1.90(-2.80,-0.90) |
| Cambodia | HAP | 5 | 2 | 2002 | 2006 | 0.000 | -3.10(-3.60,-2.60) |
| Cambodia | HAP | 5 | 3 | 2006 | 2013 | 0.000 | -3.90(-4.10,-3.80) |
| Cambodia | HAP | 5 | 4 | 2013 | 2019 | 0.000 | -3.30(-3.50,-3.10) |
| Cambodia | HAP | 5 | 5 | 2019 | 2021 | 0.001 | -1.90(-2.80,-0.90) |
| Cambodia | PMP | 5 | 0 | 1990 | 1992 | 0.000 | -1.90(-2.70,-1.20) |
| Cambodia | PMP | 5 | 1 | 1992 | 1997 | 0.000 | -0.90(-1.20,-0.70) |
| Cambodia | PMP | 5 | 2 | 1997 | 2002 | 0.000 | -1.50(-1.80,-1.30) |
| Cambodia | PMP | 5 | 3 | 2002 | 2006 | 0.000 | -3.00(-3.30,-2.60) |
| Cambodia | PMP | 5 | 4 | 2006 | 2014 | 0.000 | -3.80(-3.90,-3.70) |
| Cambodia | PMP | 5 | 5 | 2014 | 2021 | 0.000 | -2.20(-2.30,-2.10) |
| Cameroon | APMP | 5 | 0 | 1990 | 1994 | 0.000 | -6.50(-7.10,-5.80) |
| Cameroon | APMP | 5 | 1 | 1994 | 2000 | 0.000 | -1.10(-1.60,-0.60) |
| Cameroon | APMP | 5 | 2 | 2000 | 2005 | 0.000 | -2.60(-3.30,-1.90) |
| Cameroon | APMP | 5 | 3 | 2005 | 2011 | 0.006 | 0.80(0.30,1.30) |
| Cameroon | APMP | 5 | 4 | 2011 | 2014 | 0.000 | 9.80(7.30,12.40) |
| Cameroon | APMP | 5 | 5 | 2014 | 2021 | 0.000 | -3.20(-3.50,-2.90) |
| Cameroon | HAP | 3 | 0 | 1990 | 1994 | 0.135 | 0.20(-0.10,0.50) |
| Cameroon | HAP | 3 | 1 | 1994 | 2001 | 0.000 | -2.60(-2.70,-2.40) |
| Cameroon | HAP | 3 | 2 | 2001 | 2010 | 0.000 | -0.30(-0.40,-0.20) |
| Cameroon | HAP | 3 | 3 | 2010 | 2021 | 0.000 | -2.20(-2.30,-2.20) |
| Cameroon | PMP | 4 | 0 | 1990 | 1994 | 0.000 | -0.90(-1.10,-0.70) |
| Cameroon | PMP | 4 | 1 | 1994 | 2001 | 0.000 | -2.40(-2.50,-2.30) |
| Cameroon | PMP | 4 | 2 | 2001 | 2012 | 0.000 | -0.40(-0.50,-0.40) |
| Cameroon | PMP | 4 | 3 | 2012 | 2015 | 0.010 | -0.80(-1.50,-0.20) |
| Cameroon | PMP | 4 | 4 | 2015 | 2021 | 0.000 | -2.50(-2.60,-2.40) |
| Canada | APMP | 2 | 0 | 1990 | 2000 | 0.000 | -2.50(-3.40,-1.70) |
| Canada | APMP | 2 | 1 | 2000 | 2004 | 0.271 | 3.20(-2.60,9.20) |
| Canada | APMP | 2 | 2 | 2004 | 2021 | 0.000 | -3.70(-4.10,-3.40) |
| Canada | HAP | 3 | 0 | 1990 | 1995 | 0.572 | -2.00(-9.00,5.50) |
| Canada | HAP | 3 | 1 | 1995 | 2005 | 0.000 | -12.10(-14.70,-9.40) |
| Canada | HAP | 3 | 2 | 2005 | 2010 | 0.000 | -24.80(-32.30,-16.40) |
| Canada | HAP | 3 | 3 | 2010 | 2021 | 0.003 | -3.50(-5.70,-1.40) |
| Canada | PMP | 2 | 0 | 1990 | 2000 | 0.000 | -2.50(-3.40,-1.70) |
| Canada | PMP | 2 | 1 | 2000 | 2004 | 0.281 | 3.10(-2.60,9.20) |
| Canada | PMP | 2 | 2 | 2004 | 2021 | 0.000 | -3.70(-4.10,-3.40) |
| Central African Republic | APMP | 4 | 0 | 1990 | 1993 | 0.048 | -1.50(-2.90,0.00) |
| Central African Republic | APMP | 4 | 1 | 1993 | 2010 | 0.355 | -0.10(-0.20,0.10) |
| Central African Republic | APMP | 4 | 2 | 2010 | 2015 | 0.000 | 2.00(1.10,3.00) |
| Central African Republic | APMP | 4 | 3 | 2015 | 2019 | 0.000 | -4.90(-6.30,-3.50) |
| Central African Republic | APMP | 4 | 4 | 2019 | 2021 | 0.309 | -1.50(-4.40,1.50) |
| Central African Republic | HAP | 3 | 0 | 1990 | 1999 | 0.000 | -0.40(-0.50,-0.20) |
| Central African Republic | HAP | 3 | 1 | 1999 | 2007 | 0.000 | 0.60(0.40,0.70) |
| Central African Republic | HAP | 3 | 2 | 2007 | 2015 | 0.000 | -0.70(-0.90,-0.50) |
| Central African Republic | HAP | 3 | 3 | 2015 | 2021 | 0.000 | -1.50(-1.80,-1.30) |
| Central African Republic | PMP | 3 | 0 | 1990 | 1998 | 0.000 | -0.40(-0.60,-0.20) |
| Central African Republic | PMP | 3 | 1 | 1998 | 2007 | 0.000 | 0.40(0.30,0.60) |
| Central African Republic | PMP | 3 | 2 | 2007 | 2015 | 0.000 | -0.60(-0.80,-0.40) |
| Central African Republic | PMP | 3 | 3 | 2015 | 2021 | 0.000 | -1.70(-2.00,-1.50) |
| Chad | APMP | 3 | 0 | 1990 | 1994 | 0.001 | -3.00(-4.60,-1.40) |
| Chad | APMP | 3 | 1 | 1994 | 2010 | 0.002 | 0.40(0.20,0.60) |
| Chad | APMP | 3 | 2 | 2010 | 2015 | 0.000 | 5.40(3.70,7.20) |
| Chad | APMP | 3 | 3 | 2015 | 2021 | 0.000 | -4.30(-5.10,-3.40) |
| Chad | HAP | 5 | 0 | 1990 | 1994 | 0.000 | -0.80(-1.00,-0.60) |
| Chad | HAP | 5 | 1 | 1994 | 2000 | 0.000 | -1.70(-1.80,-1.60) |
| Chad | HAP | 5 | 2 | 2000 | 2004 | 0.317 | -0.20(-0.50,0.20) |
| Chad | HAP | 5 | 3 | 2004 | 2015 | 0.000 | -0.90(-1.00,-0.90) |
| Chad | HAP | 5 | 4 | 2015 | 2019 | 0.000 | 0.80(0.50,1.10) |
| Chad | HAP | 5 | 5 | 2019 | 2021 | 0.168 | -0.40(-1.10,0.20) |
| Chad | PMP | 5 | 0 | 1990 | 1994 | 0.000 | -1.10(-1.30,-0.90) |
| Chad | PMP | 5 | 1 | 1994 | 2000 | 0.000 | -1.40(-1.60,-1.30) |
| Chad | PMP | 5 | 2 | 2000 | 2004 | 0.491 | -0.10(-0.40,0.20) |
| Chad | PMP | 5 | 3 | 2004 | 2008 | 0.000 | -0.90(-1.20,-0.70) |
| Chad | PMP | 5 | 4 | 2008 | 2018 | 0.001 | -0.10(-0.20,-0.10) |
| Chad | PMP | 5 | 5 | 2018 | 2021 | 0.005 | -0.40(-0.70,-0.20) |
| Chile | APMP | 5 | 0 | 1990 | 1996 | 0.599 | 0.30(-1.00,1.70) |
| Chile | APMP | 5 | 1 | 1996 | 1999 | 0.123 | 6.20(-1.80,14.80) |
| Chile | APMP | 5 | 2 | 1999 | 2002 | 0.249 | -4.30(-11.50,3.50) |
| Chile | APMP | 5 | 3 | 2002 | 2012 | 0.001 | -1.40(-2.10,-0.70) |
| Chile | APMP | 5 | 4 | 2012 | 2019 | 0.183 | 0.90(-0.50,2.20) |
| Chile | APMP | 5 | 5 | 2019 | 2021 | 0.001 | -13.90(-20.40,-6.90) |
| Chile | HAP | 3 | 0 | 1990 | 2003 | 0.000 | -12.00(-12.50,-11.50) |
| Chile | HAP | 3 | 1 | 2003 | 2006 | 0.101 | -8.00(-16.80,1.80) |
| Chile | HAP | 3 | 2 | 2006 | 2014 | 0.000 | -15.20(-16.30,-14.00) |
| Chile | HAP | 3 | 3 | 2014 | 2021 | 0.000 | -8.30(-9.50,-7.10) |
| Chile | PMP | 5 | 0 | 1990 | 1995 | 0.000 | -7.10(-8.70,-5.40) |
| Chile | PMP | 5 | 1 | 1995 | 1999 | 0.633 | -0.90(-4.80,3.20) |
| Chile | PMP | 5 | 2 | 1999 | 2002 | 0.114 | -6.20(-13.40,1.70) |
| Chile | PMP | 5 | 3 | 2002 | 2012 | 0.000 | -3.20(-3.90,-2.50) |
| Chile | PMP | 5 | 4 | 2012 | 2019 | 0.677 | 0.30(-1.10,1.70) |
| Chile | PMP | 5 | 5 | 2019 | 2021 | 0.001 | -13.70(-20.40,-6.40) |
| China | APMP | 3 | 0 | 1990 | 1998 | 0.000 | 1.70(1.20,2.30) |
| China | APMP | 3 | 1 | 1998 | 2014 | 0.348 | 0.10(-0.10,0.30) |
| China | APMP | 3 | 2 | 2014 | 2018 | 0.000 | -6.20(-8.40,-4.10) |
| China | APMP | 3 | 3 | 2018 | 2021 | 0.000 | -10.90(-12.90,-8.80) |
| China | HAP | 2 | 0 | 1990 | 1999 | 0.000 | -2.60(-3.50,-1.70) |
| China | HAP | 2 | 1 | 1999 | 2008 | 0.000 | -8.90(-10.00,-7.90) |
| China | HAP | 2 | 2 | 2008 | 2021 | 0.000 | -16.10(-16.60,-15.70) |
| China | PMP | 3 | 0 | 1990 | 1997 | 0.001 | -1.40(-2.10,-0.70) |
| China | PMP | 3 | 1 | 1997 | 2004 | 0.000 | -5.20(-6.00,-4.30) |
| China | PMP | 3 | 2 | 2004 | 2016 | 0.000 | -7.90(-8.20,-7.50) |
| China | PMP | 3 | 3 | 2016 | 2021 | 0.000 | -11.20(-12.20,-10.10) |
| Colombia | APMP | 3 | 0 | 1990 | 1998 | 0.399 | -0.30(-0.90,0.40) |
| Colombia | APMP | 3 | 1 | 1998 | 2015 | 0.000 | -4.30(-4.50,-4.10) |
| Colombia | APMP | 3 | 2 | 2015 | 2019 | 0.000 | -6.90(-9.50,-4.10) |
| Colombia | APMP | 3 | 3 | 2019 | 2021 | 0.000 | -20.40(-24.90,-15.70) |
| Colombia | HAP | 5 | 0 | 1990 | 1999 | 0.000 | -6.00(-6.20,-5.70) |
| Colombia | HAP | 5 | 1 | 1999 | 2006 | 0.000 | -4.70(-5.10,-4.20) |
| Colombia | HAP | 5 | 2 | 2006 | 2009 | 0.000 | -12.00(-14.50,-9.50) |
| Colombia | HAP | 5 | 3 | 2009 | 2016 | 0.000 | -16.40(-16.80,-16.00) |
| Colombia | HAP | 5 | 4 | 2016 | 2019 | 0.000 | -13.40(-15.80,-10.90) |
| Colombia | HAP | 5 | 5 | 2019 | 2021 | 0.000 | -20.80(-23.00,-18.50) |
| Colombia | PMP | 3 | 0 | 1990 | 1999 | 0.000 | -2.60(-3.00,-2.20) |
| Colombia | PMP | 3 | 1 | 1999 | 2008 | 0.000 | -4.90(-5.40,-4.50) |
| Colombia | PMP | 3 | 2 | 2008 | 2019 | 0.000 | -7.40(-7.70,-7.10) |
| Colombia | PMP | 3 | 3 | 2019 | 2021 | 0.000 | -20.60(-24.10,-16.90) |
| Comoros | APMP | 1 | 0 | 1990 | 2004 | 0.002 | 0.80(0.30,1.20) |
| Comoros | APMP | 1 | 1 | 2004 | 2021 | 0.000 | -2.70(-3.00,-2.30) |
| Comoros | HAP | 4 | 0 | 1990 | 1995 | 0.999 | 0.00(-0.10,0.10) |
| Comoros | HAP | 4 | 1 | 1995 | 2000 | 0.000 | -1.20(-1.40,-1.10) |
| Comoros | HAP | 4 | 2 | 2000 | 2006 | 0.000 | -1.90(-2.00,-1.80) |
| Comoros | HAP | 4 | 3 | 2006 | 2011 | 0.000 | -1.80(-1.90,-1.70) |
| Comoros | HAP | 4 | 4 | 2011 | 2021 | 0.000 | -2.80(-2.80,-2.80) |
| Comoros | PMP | 3 | 0 | 1990 | 1995 | 0.280 | -0.10(-0.10,0.00) |
| Comoros | PMP | 3 | 1 | 1995 | 2000 | 0.000 | -1.10(-1.20,-0.90) |
| Comoros | PMP | 3 | 2 | 2000 | 2011 | 0.000 | -1.90(-1.90,-1.80) |
| Comoros | PMP | 3 | 3 | 2011 | 2021 | 0.000 | -2.80(-2.80,-2.80) |
| Congo | APMP | 5 | 0 | 1990 | 1995 | 0.099 | -0.20(-0.50,0.10) |
| Congo | APMP | 5 | 1 | 1995 | 2001 | 0.000 | 1.60(1.30,1.90) |
| Congo | APMP | 5 | 2 | 2001 | 2004 | 0.000 | 3.40(2.00,4.80) |
| Congo | APMP | 5 | 3 | 2004 | 2010 | 0.000 | 1.10(0.80,1.40) |
| Congo | APMP | 5 | 4 | 2010 | 2014 | 0.000 | 6.70(6.00,7.40) |
| Congo | APMP | 5 | 5 | 2014 | 2021 | 0.000 | -2.40(-2.60,-2.30) |
| Congo | HAP | 4 | 0 | 1990 | 2001 | 0.000 | 0.40(0.30,0.50) |
| Congo | HAP | 4 | 1 | 2001 | 2004 | 0.015 | -1.50(-2.60,-0.30) |
| Congo | HAP | 4 | 2 | 2004 | 2011 | 0.000 | -4.00(-4.20,-3.80) |
| Congo | HAP | 4 | 3 | 2011 | 2018 | 0.000 | -5.50(-5.70,-5.40) |
| Congo | HAP | 4 | 4 | 2018 | 2021 | 0.000 | -3.70(-4.30,-3.10) |
| Congo | PMP | 5 | 0 | 1990 | 1996 | 0.066 | 0.20(0.00,0.40) |
| Congo | PMP | 5 | 1 | 1996 | 2000 | 0.015 | 0.90(0.20,1.60) |
| Congo | PMP | 5 | 2 | 2000 | 2004 | 0.116 | -0.50(-1.20,0.10) |
| Congo | PMP | 5 | 3 | 2004 | 2008 | 0.000 | -3.30(-3.90,-2.60) |
| Congo | PMP | 5 | 4 | 2008 | 2014 | 0.000 | -2.50(-2.80,-2.20) |
| Congo | PMP | 5 | 5 | 2014 | 2021 | 0.000 | -4.00(-4.20,-3.90) |
| Cook Islands | APMP | 3 | 0 | 1990 | 2003 | 0.000 | -6.00(-6.30,-5.80) |
| Cook Islands | APMP | 3 | 1 | 2003 | 2014 | 0.000 | -11.20(-11.50,-10.90) |
| Cook Islands | APMP | 3 | 2 | 2014 | 2019 | 0.052 | -1.40(-2.90,0.00) |
| Cook Islands | APMP | 3 | 3 | 2019 | 2021 | 0.000 | 18.70(13.30,24.30) |
| Cook Islands | HAP | 5 | 0 | 1990 | 1995 | 0.000 | -19.10(-20.40,-17.80) |
| Cook Islands | HAP | 5 | 1 | 1995 | 2000 | 0.000 | -12.30(-14.20,-10.20) |
| Cook Islands | HAP | 5 | 2 | 2000 | 2013 | 0.000 | -16.30(-16.70,-16.00) |
| Cook Islands | HAP | 5 | 3 | 2013 | 2016 | 0.011 | -9.40(-15.80,-2.60) |
| Cook Islands | HAP | 5 | 4 | 2016 | 2019 | 0.000 | -15.40(-21.30,-9.00) |
| Cook Islands | HAP | 5 | 5 | 2019 | 2021 | 0.000 | 17.70(9.50,26.60) |
| Cook Islands | PMP | 3 | 0 | 1990 | 2004 | 0.000 | -7.60(-7.90,-7.30) |
| Cook Islands | PMP | 3 | 1 | 2004 | 2013 | 0.000 | -12.00(-12.70,-11.30) |
| Cook Islands | PMP | 3 | 2 | 2013 | 2019 | 0.000 | -3.20(-4.70,-1.70) |
| Cook Islands | PMP | 3 | 3 | 2019 | 2021 | 0.000 | 20.10(12.10,28.70) |
| Costa Rica | APMP | 1 | 0 | 1990 | 2001 | 0.102 | -0.70(-1.50,0.10) |
| Costa Rica | APMP | 1 | 1 | 2001 | 2021 | 0.000 | -3.70(-4.10,-3.40) |
| Costa Rica | HAP | 2 | 0 | 1990 | 2010 | 0.000 | -10.10(-10.40,-9.90) |
| Costa Rica | HAP | 2 | 1 | 2010 | 2017 | 0.000 | -12.10(-13.80,-10.40) |
| Costa Rica | HAP | 2 | 2 | 2017 | 2021 | 0.000 | -7.60(-10.80,-4.20) |
| Costa Rica | PMP | 0 | 0 | 1990 | 2021 | 0.000 | -4.40(-4.50,-4.30) |
| Coted'Ivoire | APMP | 4 | 0 | 1990 | 1994 | 0.000 | -6.00(-8.00,-3.90) |
| Coted'Ivoire | APMP | 4 | 1 | 1994 | 2000 | 0.000 | 4.30(2.70,5.90) |
| Coted'Ivoire | APMP | 4 | 2 | 2000 | 2010 | 0.000 | -3.20(-3.80,-2.60) |
| Coted'Ivoire | APMP | 4 | 3 | 2010 | 2015 | 0.000 | 8.00(5.70,10.40) |
| Coted'Ivoire | APMP | 4 | 4 | 2015 | 2021 | 0.000 | -4.10(-5.20,-3.00) |
| Coted'Ivoire | HAP | 2 | 0 | 1990 | 1995 | 0.001 | 1.60(0.80,2.40) |
| Coted'Ivoire | HAP | 2 | 1 | 1995 | 2009 | 0.000 | -0.40(-0.60,-0.20) |
| Coted'Ivoire | HAP | 2 | 2 | 2009 | 2021 | 0.000 | -3.00(-3.20,-2.80) |
| Coted'Ivoire | PMP | 4 | 0 | 1990 | 1992 | 0.061 | -1.20(-2.50,0.10) |
| Coted'Ivoire | PMP | 4 | 1 | 1992 | 1997 | 0.000 | 1.20(0.80,1.60) |
| Coted'Ivoire | PMP | 4 | 2 | 1997 | 2006 | 0.000 | -0.60(-0.70,-0.40) |
| Coted'Ivoire | PMP | 4 | 3 | 2006 | 2015 | 0.000 | -1.30(-1.50,-1.20) |
| Coted'Ivoire | PMP | 4 | 4 | 2015 | 2021 | 0.000 | -3.00(-3.20,-2.80) |
| Croatia | APMP | 2 | 0 | 1990 | 2003 | 0.000 | -3.60(-4.10,-3.10) |
| Croatia | APMP | 2 | 1 | 2003 | 2010 | 0.000 | -7.20(-8.80,-5.60) |
| Croatia | APMP | 2 | 2 | 2010 | 2021 | 0.000 | -5.50(-6.10,-4.80) |
| Croatia | HAP | 5 | 0 | 1990 | 1992 | 0.030 | 13.30(1.40,26.60) |
| Croatia | HAP | 5 | 1 | 1992 | 1997 | 0.013 | -4.50(-7.80,-1.10) |
| Croatia | HAP | 5 | 2 | 1997 | 2002 | 0.000 | -13.40(-16.40,-10.30) |
| Croatia | HAP | 5 | 3 | 2002 | 2005 | 0.000 | -21.70(-29.90,-12.50) |
| Croatia | HAP | 5 | 4 | 2005 | 2010 | 0.000 | -17.00(-19.80,-14.00) |
| Croatia | HAP | 5 | 5 | 2010 | 2021 | 0.000 | -9.60(-10.30,-9.00) |
| Croatia | PMP | 3 | 0 | 1990 | 1992 | 0.610 | 2.30(-6.70,12.20) |
| Croatia | PMP | 3 | 1 | 1992 | 2003 | 0.000 | -4.60(-5.30,-3.90) |
| Croatia | PMP | 3 | 2 | 2003 | 2010 | 0.000 | -7.80(-9.20,-6.30) |
| Croatia | PMP | 3 | 3 | 2010 | 2021 | 0.000 | -5.50(-6.10,-4.90) |
| Cuba | APMP | 1 | 0 | 1990 | 2000 | 0.000 | -7.50(-9.00,-6.00) |
| Cuba | APMP | 1 | 1 | 2000 | 2021 | 0.000 | -2.20(-2.80,-1.70) |
| Cuba | HAP | 4 | 0 | 1990 | 1995 | 0.862 | -0.30(-3.20,2.80) |
| Cuba | HAP | 4 | 1 | 1995 | 1998 | 0.025 | -14.60(-25.40,-2.20) |
| Cuba | HAP | 4 | 2 | 1998 | 2003 | 0.016 | -5.30(-9.30,-1.10) |
| Cuba | HAP | 4 | 3 | 2003 | 2017 | 0.000 | -11.60(-12.20,-10.90) |
| Cuba | HAP | 4 | 4 | 2017 | 2021 | 0.004 | -6.40(-10.30,-2.30) |
| Cuba | PMP | 1 | 0 | 1990 | 2007 | 0.000 | -5.90(-6.60,-5.20) |
| Cuba | PMP | 1 | 1 | 2007 | 2021 | 0.000 | -2.20(-3.10,-1.20) |
| Cyprus | APMP | 2 | 0 | 1990 | 1994 | 0.000 | -4.40(-6.20,-2.50) |
| Cyprus | APMP | 2 | 1 | 1994 | 2003 | 0.000 | -9.10(-9.70,-8.50) |
| Cyprus | APMP | 2 | 2 | 2003 | 2021 | 0.000 | -6.90(-7.10,-6.70) |
| Cyprus | HAP | 1 | 0 | 1990 | 2010 | 0.000 | -20.40(-20.60,-20.10) |
| Cyprus | HAP | 1 | 1 | 2010 | 2021 | 0.000 | -6.50(-7.20,-5.80) |
| Cyprus | PMP | 2 | 0 | 1990 | 1994 | 0.000 | -4.50(-6.30,-2.60) |
| Cyprus | PMP | 2 | 1 | 1994 | 2003 | 0.000 | -9.10(-9.70,-8.50) |
| Cyprus | PMP | 2 | 2 | 2003 | 2021 | 0.000 | -6.90(-7.10,-6.70) |
| Czechia | APMP | 4 | 0 | 1990 | 1995 | 0.000 | -8.30(-10.10,-6.40) |
| Czechia | APMP | 4 | 1 | 1995 | 1999 | 0.000 | -18.00(-21.60,-14.20) |
| Czechia | APMP | 4 | 2 | 1999 | 2004 | 0.026 | -3.30(-6.00,-0.40) |
| Czechia | APMP | 4 | 3 | 2004 | 2017 | 0.000 | -4.90(-5.40,-4.30) |
| Czechia | APMP | 4 | 4 | 2017 | 2021 | 0.000 | -9.30(-11.80,-6.60) |
| Czechia | HAP | 4 | 0 | 1990 | 1995 | 0.000 | -7.40(-9.60,-5.00) |
| Czechia | HAP | 4 | 1 | 1995 | 1999 | 0.000 | -20.10(-24.40,-15.50) |
| Czechia | HAP | 4 | 2 | 1999 | 2004 | 0.000 | -9.60(-12.80,-6.40) |
| Czechia | HAP | 4 | 3 | 2004 | 2012 | 0.000 | -14.90(-16.20,-13.70) |
| Czechia | HAP | 4 | 4 | 2012 | 2021 | 0.000 | -6.60(-7.60,-5.70) |
| Czechia | PMP | 4 | 0 | 1990 | 1995 | 0.000 | -8.30(-10.10,-6.40) |
| Czechia | PMP | 4 | 1 | 1995 | 1999 | 0.000 | -18.00(-21.70,-14.20) |
| Czechia | PMP | 4 | 2 | 1999 | 2004 | 0.024 | -3.30(-6.10,-0.50) |
| Czechia | PMP | 4 | 3 | 2004 | 2017 | 0.000 | -4.90(-5.40,-4.40) |
| Czechia | PMP | 4 | 4 | 2017 | 2021 | 0.000 | -9.20(-11.80,-6.60) |
| Democratic People's Republic of Korea | APMP | 5 | 0 | 1990 | 1992 | 0.031 | -5.00(-9.20,-0.50) |
| Democratic People's Republic of Korea | APMP | 5 | 1 | 1992 | 2007 | 0.000 | -1.80(-2.00,-1.60) |
| Democratic People's Republic of Korea | APMP | 5 | 2 | 2007 | 2010 | 0.950 | -0.10(-4.60,4.50) |
| Democratic People's Republic of Korea | APMP | 5 | 3 | 2010 | 2015 | 0.000 | -3.60(-4.90,-2.10) |
| Democratic People's Republic of Korea | APMP | 5 | 4 | 2015 | 2019 | 0.000 | -13.60(-15.50,-11.60) |
| Democratic People's Republic of Korea | APMP | 5 | 5 | 2019 | 2021 | 0.003 | -7.50(-11.60,-3.10) |
| Democratic People's Republic of Korea | HAP | 5 | 0 | 1990 | 1993 | 0.014 | -1.50(-2.60,-0.30) |
| Democratic People's Republic of Korea | HAP | 5 | 1 | 1993 | 1996 | 0.213 | 1.40(-0.90,3.70) |
| Democratic People's Republic of Korea | HAP | 5 | 2 | 1996 | 2002 | 0.045 | -0.50(-1.00,0.00) |
| Democratic People's Republic of Korea | HAP | 5 | 3 | 2002 | 2005 | 0.000 | -4.70(-6.80,-2.50) |
| Democratic People's Republic of Korea | HAP | 5 | 4 | 2005 | 2010 | 0.000 | 1.90(1.20,2.70) |
| Democratic People's Republic of Korea | HAP | 5 | 5 | 2010 | 2021 | 0.000 | -6.70(-6.90,-6.60) |
| Democratic People's Republic of Korea | PMP | 5 | 0 | 1990 | 1992 | 0.185 | -2.00(-5.10,1.10) |
| Democratic People's Republic of Korea | PMP | 5 | 1 | 1992 | 2001 | 0.812 | 0.00(-0.40,0.30) |
| Democratic People's Republic of Korea | PMP | 5 | 2 | 2001 | 2006 | 0.000 | -3.40(-4.30,-2.40) |
| Democratic People's Republic of Korea | PMP | 5 | 3 | 2006 | 2009 | 0.034 | 3.50(0.30,6.90) |
| Democratic People's Republic of Korea | PMP | 5 | 4 | 2009 | 2012 | 0.015 | -4.00(-7.00,-0.90) |
| Democratic People's Republic of Korea | PMP | 5 | 5 | 2012 | 2021 | 0.000 | -7.20(-7.40,-6.90) |
| Democratic Republic of the Congo | APMP | 5 | 0 | 1990 | 1998 | 0.000 | -3.30(-3.50,-3.10) |
| Democratic Republic of the Congo | APMP | 5 | 1 | 1998 | 2001 | 0.038 | -1.70(-3.40,-0.10) |
| Democratic Republic of the Congo | APMP | 5 | 2 | 2001 | 2005 | 0.000 | 4.40(3.50,5.20) |
| Democratic Republic of the Congo | APMP | 5 | 3 | 2005 | 2010 | 0.003 | -0.90(-1.40,-0.30) |
| Democratic Republic of the Congo | APMP | 5 | 4 | 2010 | 2015 | 0.000 | 1.20(0.70,1.70) |
| Democratic Republic of the Congo | APMP | 5 | 5 | 2015 | 2021 | 0.000 | -4.10(-4.40,-3.80) |
| Democratic Republic of the Congo | HAP | 5 | 0 | 1990 | 2001 | 0.000 | -0.90(-1.00,-0.90) |
| Democratic Republic of the Congo | HAP | 5 | 1 | 2001 | 2005 | 0.000 | 4.10(3.60,4.60) |
| Democratic Republic of the Congo | HAP | 5 | 2 | 2005 | 2008 | 0.992 | 0.00(-0.90,0.90) |
| Democratic Republic of the Congo | HAP | 5 | 3 | 2008 | 2013 | 0.000 | -1.40(-1.70,-1.10) |
| Democratic Republic of the Congo | HAP | 5 | 4 | 2013 | 2017 | 0.000 | -2.70(-3.10,-2.20) |
| Democratic Republic of the Congo | HAP | 5 | 5 | 2017 | 2021 | 0.000 | -4.40(-4.70,-4.20) |
| Democratic Republic of the Congo | PMP | 5 | 0 | 1990 | 2001 | 0.000 | -1.10(-1.10,-1.00) |
| Democratic Republic of the Congo | PMP | 5 | 1 | 2001 | 2005 | 0.000 | 4.20(3.60,4.70) |
| Democratic Republic of the Congo | PMP | 5 | 2 | 2005 | 2008 | 0.896 | -0.10(-1.10,1.00) |
| Democratic Republic of the Congo | PMP | 5 | 3 | 2008 | 2013 | 0.000 | -1.30(-1.60,-1.00) |
| Democratic Republic of the Congo | PMP | 5 | 4 | 2013 | 2016 | 0.001 | -2.20(-3.20,-1.10) |
| Democratic Republic of the Congo | PMP | 5 | 5 | 2016 | 2021 | 0.000 | -4.20(-4.40,-4.00) |
| Denmark | APMP | 5 | 0 | 1990 | 1993 | 0.007 | -8.40(-13.70,-2.80) |
| Denmark | APMP | 5 | 1 | 1993 | 1996 | 0.689 | 2.30(-9.20,15.20) |
| Denmark | APMP | 5 | 2 | 1996 | 1999 | 0.167 | -7.80(-18.10,3.90) |
| Denmark | APMP | 5 | 3 | 1999 | 2004 | 0.627 | 0.90(-2.80,4.70) |
| Denmark | APMP | 5 | 4 | 2004 | 2017 | 0.000 | -3.70(-4.40,-3.00) |
| Denmark | APMP | 5 | 5 | 2017 | 2021 | 0.001 | -6.90(-10.30,-3.30) |
| Denmark | HAP | 0 | 0 | 1990 | 2021 | 0.000 | -8.10(-9.20,-7.10) |
| Denmark | PMP | 5 | 0 | 1990 | 1993 | 0.007 | -8.40(-13.70,-2.80) |
| Denmark | PMP | 5 | 1 | 1993 | 1996 | 0.690 | 2.30(-9.20,15.20) |
| Denmark | PMP | 5 | 2 | 1996 | 1999 | 0.167 | -7.80(-18.10,3.90) |
| Denmark | PMP | 5 | 3 | 1999 | 2004 | 0.629 | 0.90(-2.80,4.70) |
| Denmark | PMP | 5 | 4 | 2004 | 2017 | 0.000 | -3.70(-4.40,-3.00) |
| Denmark | PMP | 5 | 5 | 2017 | 2021 | 0.001 | -6.90(-10.30,-3.30) |
| Djibouti | APMP | 4 | 0 | 1990 | 1996 | 0.524 | 0.20(-0.50,1.00) |
| Djibouti | APMP | 4 | 1 | 1996 | 2000 | 0.000 | 5.00(2.70,7.30) |
| Djibouti | APMP | 4 | 2 | 2000 | 2006 | 0.064 | 0.90(-0.10,2.00) |
| Djibouti | APMP | 4 | 3 | 2006 | 2016 | 0.001 | -0.80(-1.20,-0.40) |
| Djibouti | APMP | 4 | 4 | 2016 | 2021 | 0.000 | -4.70(-5.70,-3.80) |
| Djibouti | HAP | 5 | 0 | 1990 | 1993 | 0.034 | 1.40(0.10,2.70) |
| Djibouti | HAP | 5 | 1 | 1993 | 1996 | 0.009 | -3.50(-5.90,-1.00) |
| Djibouti | HAP | 5 | 2 | 1996 | 2001 | 0.000 | -4.90(-5.70,-4.20) |
| Djibouti | HAP | 5 | 3 | 2001 | 2004 | 0.000 | -6.70(-9.00,-4.30) |
| Djibouti | HAP | 5 | 4 | 2004 | 2015 | 0.000 | -2.20(-2.40,-2.00) |
| Djibouti | HAP | 5 | 5 | 2015 | 2021 | 0.000 | -5.10(-5.50,-4.70) |
| Djibouti | PMP | 3 | 0 | 1990 | 1993 | 0.227 | 1.10(-0.70,2.90) |
| Djibouti | PMP | 3 | 1 | 1993 | 2007 | 0.000 | -2.00(-2.20,-1.80) |
| Djibouti | PMP | 3 | 2 | 2007 | 2015 | 0.000 | -1.30(-1.70,-0.80) |
| Djibouti | PMP | 3 | 3 | 2015 | 2021 | 0.000 | -4.60(-5.20,-4.00) |
| Dominica | APMP | 5 | 0 | 1990 | 1994 | 0.000 | 5.10(3.50,6.80) |
| Dominica | APMP | 5 | 1 | 1994 | 1999 | 0.004 | 2.50(0.90,4.20) |
| Dominica | APMP | 5 | 2 | 1999 | 2004 | 0.541 | -0.50(-2.00,1.10) |
| Dominica | APMP | 5 | 3 | 2004 | 2008 | 0.098 | 2.10(-0.40,4.70) |
| Dominica | APMP | 5 | 4 | 2008 | 2014 | 0.000 | 8.60(7.40,9.80) |
| Dominica | APMP | 5 | 5 | 2014 | 2021 | 0.001 | -1.20(-1.90,-0.60) |
| Dominica | HAP | 5 | 0 | 1990 | 2000 | 0.000 | -8.40(-8.80,-8.00) |
| Dominica | HAP | 5 | 1 | 2000 | 2005 | 0.000 | -4.20(-5.90,-2.50) |
| Dominica | HAP | 5 | 2 | 2005 | 2008 | 0.010 | -7.40(-12.40,-2.10) |
| Dominica | HAP | 5 | 3 | 2008 | 2013 | 0.005 | -2.70(-4.40,-0.90) |
| Dominica | HAP | 5 | 4 | 2013 | 2018 | 0.000 | -4.70(-6.40,-3.00) |
| Dominica | HAP | 5 | 5 | 2018 | 2021 | 0.753 | -0.40(-3.10,2.40) |
| Dominica | PMP | 4 | 0 | 1990 | 2002 | 0.000 | -2.60(-2.90,-2.40) |
| Dominica | PMP | 4 | 1 | 2002 | 2008 | 0.081 | -0.80(-1.80,0.10) |
| Dominica | PMP | 4 | 2 | 2008 | 2014 | 0.000 | 6.50(5.50,7.50) |
| Dominica | PMP | 4 | 3 | 2014 | 2018 | 0.028 | -2.40(-4.40,-0.30) |
| Dominica | PMP | 4 | 4 | 2018 | 2021 | 0.858 | 0.20(-1.90,2.30) |
| Dominican Republic | APMP | 3 | 0 | 1990 | 1995 | 0.643 | 0.40(-1.30,2.10) |
| Dominican Republic | APMP | 3 | 1 | 1995 | 2000 | 0.000 | 6.10(3.50,8.70) |
| Dominican Republic | APMP | 3 | 2 | 2000 | 2013 | 0.000 | 0.90(0.50,1.40) |
| Dominican Republic | APMP | 3 | 3 | 2013 | 2021 | 0.000 | -1.70(-2.50,-0.80) |
| Dominican Republic | HAP | 4 | 0 | 1990 | 2002 | 0.000 | -10.10(-10.60,-9.70) |
| Dominican Republic | HAP | 4 | 1 | 2002 | 2006 | 0.002 | -7.20(-11.10,-3.10) |
| Dominican Republic | HAP | 4 | 2 | 2006 | 2013 | 0.000 | -11.60(-12.90,-10.30) |
| Dominican Republic | HAP | 4 | 3 | 2013 | 2019 | 0.000 | -14.90(-16.50,-13.20) |
| Dominican Republic | HAP | 4 | 4 | 2019 | 2021 | 0.802 | -1.00(-9.20,7.80) |
| Dominican Republic | PMP | 1 | 0 | 1990 | 1997 | 0.000 | -6.10(-7.10,-5.00) |
| Dominican Republic | PMP | 1 | 1 | 1997 | 2021 | 0.000 | -2.40(-2.60,-2.30) |
| Ecuador | APMP | 5 | 0 | 1990 | 1993 | 0.379 | -1.30(-4.40,1.80) |
| Ecuador | APMP | 5 | 1 | 1993 | 1997 | 0.015 | 4.20(0.90,7.50) |
| Ecuador | APMP | 5 | 2 | 1997 | 2005 | 0.018 | -1.10(-1.90,-0.20) |
| Ecuador | APMP | 5 | 3 | 2005 | 2010 | 0.000 | -14.20(-15.90,-12.50) |
| Ecuador | APMP | 5 | 4 | 2010 | 2018 | 0.000 | -5.00(-5.80,-4.20) |
| Ecuador | APMP | 5 | 5 | 2018 | 2021 | 0.000 | -9.80(-12.60,-6.90) |
| Ecuador | HAP | 5 | 0 | 1990 | 1993 | 0.000 | -7.70(-10.10,-5.20) |
| Ecuador | HAP | 5 | 1 | 1993 | 1998 | 0.083 | -1.40(-3.10,0.20) |
| Ecuador | HAP | 5 | 2 | 1998 | 2003 | 0.000 | -7.00(-8.50,-5.40) |
| Ecuador | HAP | 5 | 3 | 2003 | 2007 | 0.000 | -12.30(-14.60,-9.90) |
| Ecuador | HAP | 5 | 4 | 2007 | 2015 | 0.000 | -14.30(-14.90,-13.70) |
| Ecuador | HAP | 5 | 5 | 2015 | 2021 | 0.000 | -9.70(-10.50,-8.90) |
| Ecuador | PMP | 4 | 0 | 1990 | 1993 | 0.060 | -3.80(-7.50,0.20) |
| Ecuador | PMP | 4 | 1 | 1993 | 1997 | 0.157 | 2.90(-1.20,7.10) |
| Ecuador | PMP | 4 | 2 | 1997 | 2005 | 0.000 | -2.60(-3.60,-1.50) |
| Ecuador | PMP | 4 | 3 | 2005 | 2010 | 0.000 | -14.10(-16.20,-11.80) |
| Ecuador | PMP | 4 | 4 | 2010 | 2021 | 0.000 | -6.80(-7.30,-6.30) |
| Egypt | APMP | 3 | 0 | 1990 | 2006 | 0.023 | -0.50(-1.00,-0.10) |
| Egypt | APMP | 3 | 1 | 2006 | 2011 | 0.015 | -4.60(-8.10,-1.00) |
| Egypt | APMP | 3 | 2 | 2011 | 2014 | 0.001 | -19.10(-28.10,-9.10) |
| Egypt | APMP | 3 | 3 | 2014 | 2021 | 0.000 | -7.10(-8.60,-5.70) |
| Egypt | HAP | 5 | 0 | 1990 | 1995 | 0.000 | -10.40(-12.10,-8.70) |
| Egypt | HAP | 5 | 1 | 1995 | 2001 | 0.000 | -16.00(-17.60,-14.40) |
| Egypt | HAP | 5 | 2 | 2001 | 2006 | 0.000 | -24.00(-26.00,-21.90) |
| Egypt | HAP | 5 | 3 | 2006 | 2014 | 0.000 | -27.90(-28.70,-27.10) |
| Egypt | HAP | 5 | 4 | 2014 | 2018 | 0.000 | -21.10(-24.40,-17.70) |
| Egypt | HAP | 5 | 5 | 2018 | 2021 | 0.000 | -11.40(-15.00,-7.60) |
| Egypt | PMP | 2 | 0 | 1990 | 2011 | 0.000 | -2.90(-3.20,-2.60) |
| Egypt | PMP | 2 | 1 | 2011 | 2014 | 0.000 | -20.70(-29.20,-11.20) |
| Egypt | PMP | 2 | 2 | 2014 | 2021 | 0.000 | -7.00(-8.40,-5.60) |
| El Salvador | APMP | 4 | 0 | 1990 | 1995 | 0.035 | -1.70(-3.30,-0.10) |
| El Salvador | APMP | 4 | 1 | 1995 | 1998 | 0.495 | 2.40(-4.60,9.90) |
| El Salvador | APMP | 4 | 2 | 1998 | 2011 | 0.000 | -2.40(-2.80,-2.00) |
| El Salvador | APMP | 4 | 3 | 2011 | 2014 | 0.673 | 1.50(-5.50,9.00) |
| El Salvador | APMP | 4 | 4 | 2014 | 2021 | 0.000 | -9.60(-10.50,-8.80) |
| El Salvador | HAP | 4 | 0 | 1990 | 2002 | 0.000 | -7.30(-7.50,-7.10) |
| El Salvador | HAP | 4 | 1 | 2002 | 2009 | 0.000 | -6.10(-6.70,-5.40) |
| El Salvador | HAP | 4 | 2 | 2009 | 2013 | 0.000 | -10.50(-12.40,-8.60) |
| El Salvador | HAP | 4 | 3 | 2013 | 2019 | 0.000 | -16.70(-17.50,-15.90) |
| El Salvador | HAP | 4 | 4 | 2019 | 2021 | 0.001 | -7.70(-11.60,-3.70) |
| El Salvador | PMP | 4 | 0 | 1990 | 2003 | 0.000 | -5.40(-5.60,-5.20) |
| El Salvador | PMP | 4 | 1 | 2003 | 2006 | 0.038 | -4.00(-7.60,-0.20) |
| El Salvador | PMP | 4 | 2 | 2006 | 2014 | 0.000 | -5.40(-5.90,-4.90) |
| El Salvador | PMP | 4 | 3 | 2014 | 2018 | 0.000 | -13.60(-15.20,-11.90) |
| El Salvador | PMP | 4 | 4 | 2018 | 2021 | 0.000 | -8.40(-10.10,-6.60) |
| Equatorial Guinea | APMP | 5 | 0 | 1990 | 1995 | 0.009 | -1.50(-2.60,-0.40) |
| Equatorial Guinea | APMP | 5 | 1 | 1995 | 2000 | 0.000 | 24.40(22.50,26.40) |
| Equatorial Guinea | APMP | 5 | 2 | 2000 | 2004 | 0.000 | 10.30(7.60,13.00) |
| Equatorial Guinea | APMP | 5 | 3 | 2004 | 2010 | 0.000 | -3.10(-4.20,-2.10) |
| Equatorial Guinea | APMP | 5 | 4 | 2010 | 2014 | 0.061 | 2.30(-0.10,4.80) |
| Equatorial Guinea | APMP | 5 | 5 | 2014 | 2021 | 0.000 | -2.60(-3.30,-2.00) |
| Equatorial Guinea | HAP | 5 | 0 | 1990 | 1998 | 0.007 | -2.00(-3.30,-0.60) |
| Equatorial Guinea | HAP | 5 | 1 | 1998 | 2002 | 0.000 | -18.80(-23.70,-13.60) |
| Equatorial Guinea | HAP | 5 | 2 | 2002 | 2005 | 0.000 | -45.40(-51.90,-38.20) |
| Equatorial Guinea | HAP | 5 | 3 | 2005 | 2010 | 0.000 | -29.00(-31.70,-26.10) |
| Equatorial Guinea | HAP | 5 | 4 | 2010 | 2015 | 0.002 | -6.90(-10.50,-3.10) |
| Equatorial Guinea | HAP | 5 | 5 | 2015 | 2021 | 0.000 | 6.90(4.60,9.10) |
| Equatorial Guinea | PMP | 5 | 0 | 1990 | 1996 | 0.200 | -0.50(-1.20,0.30) |
| Equatorial Guinea | PMP | 5 | 1 | 1996 | 2000 | 0.003 | -3.50(-5.60,-1.40) |
| Equatorial Guinea | PMP | 5 | 2 | 2000 | 2005 | 0.000 | -10.90(-12.20,-9.70) |
| Equatorial Guinea | PMP | 5 | 3 | 2005 | 2010 | 0.000 | -5.30(-6.60,-3.90) |
| Equatorial Guinea | PMP | 5 | 4 | 2010 | 2014 | 0.028 | 2.50(0.30,4.80) |
| Equatorial Guinea | PMP | 5 | 5 | 2014 | 2021 | 0.000 | -2.50(-3.10,-1.90) |
| Eritrea | APMP | 3 | 0 | 1990 | 1995 | 0.958 | 0.00(-1.40,1.30) |
| Eritrea | APMP | 3 | 1 | 1995 | 1999 | 0.000 | 6.80(3.60,10.10) |
| Eritrea | APMP | 3 | 2 | 1999 | 2016 | 0.000 | -1.50(-1.70,-1.30) |
| Eritrea | APMP | 3 | 3 | 2016 | 2021 | 0.000 | -4.30(-5.60,-3.00) |
| Eritrea | HAP | 5 | 0 | 1990 | 1992 | 0.182 | 0.80(-0.40,1.90) |
| Eritrea | HAP | 5 | 1 | 1992 | 1995 | 0.000 | -3.00(-4.10,-1.80) |
| Eritrea | HAP | 5 | 2 | 1995 | 2000 | 0.000 | -1.90(-2.20,-1.50) |
| Eritrea | HAP | 5 | 3 | 2000 | 2004 | 0.003 | -0.90(-1.50,-0.40) |
| Eritrea | HAP | 5 | 4 | 2004 | 2010 | 0.029 | 0.30(0.00,0.60) |
| Eritrea | HAP | 5 | 5 | 2010 | 2021 | 0.000 | -2.30(-2.40,-2.30) |
| Eritrea | PMP | 5 | 0 | 1990 | 1992 | 0.073 | 0.80(-0.10,1.70) |
| Eritrea | PMP | 5 | 1 | 1992 | 1995 | 0.000 | -2.60(-3.50,-1.80) |
| Eritrea | PMP | 5 | 2 | 1995 | 2004 | 0.000 | -1.00(-1.10,-0.90) |
| Eritrea | PMP | 5 | 3 | 2004 | 2010 | 0.391 | -0.10(-0.30,0.10) |
| Eritrea | PMP | 5 | 4 | 2010 | 2016 | 0.000 | -2.00(-2.20,-1.90) |
| Eritrea | PMP | 5 | 5 | 2016 | 2021 | 0.000 | -2.80(-3.00,-2.70) |
| Estonia | APMP | 2 | 0 | 1990 | 1993 | 0.402 | 2.90(-4.00,10.30) |
| Estonia | APMP | 2 | 1 | 1993 | 2013 | 0.000 | -11.40(-11.70,-11.00) |
| Estonia | APMP | 2 | 2 | 2013 | 2021 | 0.000 | -8.00(-9.40,-6.60) |
| Estonia | HAP | 5 | 0 | 1990 | 1994 | 0.107 | 2.50(-0.60,5.70) |
| Estonia | HAP | 5 | 1 | 1994 | 2000 | 0.000 | -15.00(-16.80,-13.20) |
| Estonia | HAP | 5 | 2 | 2000 | 2011 | 0.000 | -23.60(-24.20,-23.10) |
| Estonia | HAP | 5 | 3 | 2011 | 2016 | 0.000 | -14.10(-16.70,-11.50) |
| Estonia | HAP | 5 | 4 | 2016 | 2019 | 0.000 | -19.30(-26.70,-11.10) |
| Estonia | HAP | 5 | 5 | 2019 | 2021 | 0.241 | -5.40(-14.10,4.20) |
| Estonia | PMP | 2 | 0 | 1990 | 1994 | 0.825 | 0.50(-3.80,4.90) |
| Estonia | PMP | 2 | 1 | 1994 | 2011 | 0.000 | -13.30(-13.70,-12.80) |
| Estonia | PMP | 2 | 2 | 2011 | 2021 | 0.000 | -8.80(-9.80,-7.90) |
| Eswatini | APMP | 4 | 0 | 1990 | 1995 | 0.000 | 3.30(2.00,4.50) |
| Eswatini | APMP | 4 | 1 | 1995 | 2006 | 0.000 | -1.80(-2.20,-1.40) |
| Eswatini | APMP | 4 | 2 | 2006 | 2010 | 0.001 | 5.30(2.60,8.20) |
| Eswatini | APMP | 4 | 3 | 2010 | 2018 | 0.033 | 0.80(0.10,1.50) |
| Eswatini | APMP | 4 | 4 | 2018 | 2021 | 0.060 | -2.50(-5.10,0.10) |
| Eswatini | HAP | 4 | 0 | 1990 | 2000 | 0.000 | -4.80(-4.90,-4.80) |
| Eswatini | HAP | 4 | 1 | 2000 | 2007 | 0.000 | -1.40(-1.60,-1.20) |
| Eswatini | HAP | 4 | 2 | 2007 | 2010 | 0.327 | -0.60(-1.70,0.60) |
| Eswatini | HAP | 4 | 3 | 2010 | 2018 | 0.000 | -4.70(-4.90,-4.60) |
| Eswatini | HAP | 4 | 4 | 2018 | 2021 | 0.000 | -2.90(-3.50,-2.40) |
| Eswatini | PMP | 3 | 0 | 1990 | 2001 | 0.000 | -3.40(-3.60,-3.30) |
| Eswatini | PMP | 3 | 1 | 2001 | 2006 | 0.001 | -1.40(-2.20,-0.60) |
| Eswatini | PMP | 3 | 2 | 2006 | 2010 | 0.117 | 1.00(-0.30,2.30) |
| Eswatini | PMP | 3 | 3 | 2010 | 2021 | 0.000 | -2.60(-2.70,-2.40) |
| Ethiopia | APMP | 5 | 0 | 1990 | 1992 | 0.000 | -6.90(-8.40,-5.30) |
| Ethiopia | APMP | 5 | 1 | 1992 | 1995 | 0.001 | -3.40(-5.00,-1.80) |
| Ethiopia | APMP | 5 | 2 | 1995 | 2000 | 0.000 | 3.30(2.80,3.90) |
| Ethiopia | APMP | 5 | 3 | 2000 | 2004 | 0.007 | -1.20(-2.10,-0.40) |
| Ethiopia | APMP | 5 | 4 | 2004 | 2014 | 0.000 | 0.80(0.60,0.90) |
| Ethiopia | APMP | 5 | 5 | 2014 | 2021 | 0.000 | -2.50(-2.80,-2.30) |
| Ethiopia | HAP | 4 | 0 | 1990 | 1994 | 0.000 | -0.50(-0.70,-0.30) |
| Ethiopia | HAP | 4 | 1 | 1994 | 1998 | 0.000 | -1.00(-1.30,-0.70) |
| Ethiopia | HAP | 4 | 2 | 1998 | 2007 | 0.000 | -1.60(-1.70,-1.60) |
| Ethiopia | HAP | 4 | 3 | 2007 | 2019 | 0.000 | -3.00(-3.10,-3.00) |
| Ethiopia | HAP | 4 | 4 | 2019 | 2021 | 0.000 | -2.50(-3.10,-1.90) |
| Ethiopia | PMP | 4 | 0 | 1990 | 1998 | 0.000 | -0.80(-0.80,-0.70) |
| Ethiopia | PMP | 4 | 1 | 1998 | 2007 | 0.000 | -1.50(-1.60,-1.50) |
| Ethiopia | PMP | 4 | 2 | 2007 | 2016 | 0.000 | -2.80(-2.90,-2.70) |
| Ethiopia | PMP | 4 | 3 | 2016 | 2019 | 0.000 | -3.20(-3.80,-2.50) |
| Ethiopia | PMP | 4 | 4 | 2019 | 2021 | 0.000 | -2.40(-3.10,-1.70) |
| Fiji | APMP | 5 | 0 | 1990 | 1995 | 0.000 | 3.20(1.70,4.60) |
| Fiji | APMP | 5 | 1 | 1995 | 1999 | 0.000 | 10.20(6.80,13.70) |
| Fiji | APMP | 5 | 2 | 1999 | 2002 | 0.628 | 1.50(-4.70,8.10) |
| Fiji | APMP | 5 | 3 | 2002 | 2011 | 0.001 | -1.30(-2.00,-0.70) |
| Fiji | APMP | 5 | 4 | 2011 | 2019 | 0.000 | 3.50(2.70,4.40) |
| Fiji | APMP | 5 | 5 | 2019 | 2021 | 0.415 | -2.40(-8.40,3.90) |
| Fiji | HAP | 3 | 0 | 1990 | 2003 | 0.000 | -2.20(-2.50,-2.00) |
| Fiji | HAP | 3 | 1 | 2003 | 2016 | 0.000 | -4.70(-5.00,-4.40) |
| Fiji | HAP | 3 | 2 | 2016 | 2019 | 0.000 | -9.60(-13.80,-5.20) |
| Fiji | HAP | 3 | 3 | 2019 | 2021 | 0.313 | -2.30(-6.90,2.40) |
| Fiji | PMP | 3 | 0 | 1990 | 1992 | 0.012 | -4.10(-7.10,-1.00) |
| Fiji | PMP | 3 | 1 | 1992 | 2003 | 0.006 | -0.40(-0.60,-0.10) |
| Fiji | PMP | 3 | 2 | 2003 | 2007 | 0.000 | -5.00(-6.50,-3.50) |
| Fiji | PMP | 3 | 3 | 2007 | 2021 | 0.000 | -2.60(-2.70,-2.40) |
| Finland | APMP | 4 | 0 | 1990 | 2001 | 0.000 | -6.10(-6.80,-5.40) |
| Finland | APMP | 4 | 1 | 2001 | 2005 | 0.901 | 0.30(-4.90,5.80) |
| Finland | APMP | 4 | 2 | 2005 | 2015 | 0.000 | -7.40(-8.30,-6.50) |
| Finland | APMP | 4 | 3 | 2015 | 2018 | 0.477 | 3.80(-6.80,15.50) |
| Finland | APMP | 4 | 4 | 2018 | 2021 | 0.013 | -6.70(-11.60,-1.60) |
| Finland | HAP | 4 | 0 | 1990 | 1997 | 0.000 | -11.20(-13.30,-9.00) |
| Finland | HAP | 4 | 1 | 1997 | 2001 | 0.000 | -17.70(-24.80,-10.00) |
| Finland | HAP | 4 | 2 | 2001 | 2005 | 0.127 | -6.70(-14.70,2.20) |
| Finland | HAP | 4 | 3 | 2005 | 2010 | 0.000 | -19.50(-24.00,-14.80) |
| Finland | HAP | 4 | 4 | 2010 | 2021 | 0.000 | -3.20(-4.40,-2.00) |
| Finland | PMP | 4 | 0 | 1990 | 2001 | 0.000 | -6.10(-6.80,-5.40) |
| Finland | PMP | 4 | 1 | 2001 | 2005 | 0.906 | 0.30(-4.90,5.80) |
| Finland | PMP | 4 | 2 | 2005 | 2015 | 0.000 | -7.40(-8.30,-6.50) |
| Finland | PMP | 4 | 3 | 2015 | 2018 | 0.476 | 3.80(-6.70,15.50) |
| Finland | PMP | 4 | 4 | 2018 | 2021 | 0.013 | -6.70(-11.60,-1.60) |
| France | APMP | 0 | 0 | 1990 | 2021 | 0.000 | -2.80(-2.90,-2.60) |
| France | HAP | 3 | 0 | 1990 | 1994 | 0.000 | -18.80(-22.10,-15.30) |
| France | HAP | 3 | 1 | 1994 | 2001 | 0.000 | -8.80(-10.80,-6.70) |
| France | HAP | 3 | 2 | 2001 | 2009 | 0.000 | -11.60(-13.20,-10.00) |
| France | HAP | 3 | 3 | 2009 | 2021 | 0.000 | -3.40(-4.20,-2.70) |
| France | PMP | 0 | 0 | 1990 | 2021 | 0.000 | -2.80(-3.00,-2.60) |
| Gabon | APMP | 4 | 0 | 1990 | 1994 | 0.002 | -2.80(-4.30,-1.20) |
| Gabon | APMP | 4 | 1 | 1994 | 2001 | 0.027 | 1.00(0.10,1.90) |
| Gabon | APMP | 4 | 2 | 2001 | 2011 | 0.003 | -0.80(-1.20,-0.30) |
| Gabon | APMP | 4 | 3 | 2011 | 2014 | 0.140 | 3.80(-1.30,9.20) |
| Gabon | APMP | 4 | 4 | 2014 | 2021 | 0.000 | -4.10(-4.80,-3.50) |
| Gabon | HAP | 2 | 0 | 1990 | 1994 | 0.000 | -5.00(-6.30,-3.70) |
| Gabon | HAP | 2 | 1 | 1994 | 2002 | 0.000 | -2.40(-3.00,-1.90) |
| Gabon | HAP | 2 | 2 | 2002 | 2021 | 0.000 | -7.40(-7.50,-7.30) |
| Gabon | PMP | 4 | 0 | 1990 | 1994 | 0.000 | -3.30(-4.70,-1.90) |
| Gabon | PMP | 4 | 1 | 1994 | 2001 | 0.227 | 0.50(-0.30,1.20) |
| Gabon | PMP | 4 | 2 | 2001 | 2010 | 0.000 | -1.60(-2.10,-1.20) |
| Gabon | PMP | 4 | 3 | 2010 | 2015 | 0.149 | 1.00(-0.40,2.50) |
| Gabon | PMP | 4 | 4 | 2015 | 2021 | 0.000 | -4.80(-5.50,-4.00) |
| Gambia | APMP | 3 | 0 | 1990 | 1999 | 0.000 | -1.00(-1.40,-0.70) |
| Gambia | APMP | 3 | 1 | 1999 | 2010 | 0.000 | -2.70(-3.00,-2.40) |
| Gambia | APMP | 3 | 2 | 2010 | 2014 | 0.011 | 2.80(0.70,4.90) |
| Gambia | APMP | 3 | 3 | 2014 | 2021 | 0.000 | -3.90(-4.50,-3.40) |
| Gambia | HAP | 3 | 0 | 1990 | 1995 | 0.018 | -0.20(-0.40,0.00) |
| Gambia | HAP | 3 | 1 | 1995 | 2005 | 0.000 | -1.00(-1.10,-0.90) |
| Gambia | HAP | 3 | 2 | 2005 | 2018 | 0.000 | -2.20(-2.20,-2.10) |
| Gambia | HAP | 3 | 3 | 2018 | 2021 | 0.000 | -3.10(-3.50,-2.70) |
| Gambia | PMP | 5 | 0 | 1990 | 1995 | 0.001 | -0.30(-0.50,-0.10) |
| Gambia | PMP | 5 | 1 | 1995 | 2004 | 0.000 | -1.10(-1.20,-1.00) |
| Gambia | PMP | 5 | 2 | 2004 | 2011 | 0.000 | -2.10(-2.20,-1.90) |
| Gambia | PMP | 5 | 3 | 2011 | 2014 | 0.003 | -1.20(-2.00,-0.50) |
| Gambia | PMP | 5 | 4 | 2014 | 2018 | 0.000 | -2.50(-2.80,-2.10) |
| Gambia | PMP | 5 | 5 | 2018 | 2021 | 0.000 | -3.30(-3.60,-2.90) |
| Georgia | APMP | 5 | 0 | 1990 | 1994 | 0.000 | -7.20(-9.10,-5.20) |
| Georgia | APMP | 5 | 1 | 1994 | 2001 | 0.000 | -9.90(-10.90,-8.90) |
| Georgia | APMP | 5 | 2 | 2001 | 2006 | 0.086 | 1.80(-0.30,4.00) |
| Georgia | APMP | 5 | 3 | 2006 | 2009 | 0.003 | 11.50(4.40,19.10) |
| Georgia | APMP | 5 | 4 | 2009 | 2016 | 0.000 | -2.60(-3.70,-1.50) |
| Georgia | APMP | 5 | 5 | 2016 | 2021 | 0.000 | -8.00(-9.30,-6.60) |
| Georgia | HAP | 5 | 0 | 1990 | 1993 | 0.000 | 25.10(19.60,30.70) |
| Georgia | HAP | 5 | 1 | 1993 | 1999 | 0.000 | 7.30(5.20,9.40) |
| Georgia | HAP | 5 | 2 | 1999 | 2005 | 0.000 | -4.40(-6.30,-2.50) |
| Georgia | HAP | 5 | 3 | 2005 | 2008 | 0.027 | -9.70(-17.30,-1.30) |
| Georgia | HAP | 5 | 4 | 2008 | 2019 | 0.000 | -19.40(-20.00,-18.90) |
| Georgia | HAP | 5 | 5 | 2019 | 2021 | 0.364 | -3.80(-12.00,5.10) |
| Georgia | PMP | 4 | 0 | 1990 | 1994 | 0.000 | 8.50(5.20,11.90) |
| Georgia | PMP | 4 | 1 | 1994 | 1999 | 0.071 | 2.80(-0.30,6.00) |
| Georgia | PMP | 4 | 2 | 1999 | 2008 | 0.000 | -4.50(-5.50,-3.50) |
| Georgia | PMP | 4 | 3 | 2008 | 2014 | 0.000 | -13.00(-14.90,-11.10) |
| Georgia | PMP | 4 | 4 | 2014 | 2021 | 0.000 | -9.80(-10.90,-8.60) |
| Germany | APMP | 2 | 0 | 1990 | 1992 | 0.004 | -12.50(-19.70,-4.60) |
| Germany | APMP | 2 | 1 | 1992 | 2007 | 0.000 | -2.40(-2.80,-2.00) |
| Germany | APMP | 2 | 2 | 2007 | 2021 | 0.000 | -5.30(-5.70,-4.90) |
| Germany | HAP | 2 | 0 | 1990 | 1995 | 0.000 | -15.50(-19.30,-11.50) |
| Germany | HAP | 2 | 1 | 1995 | 2004 | 0.000 | -5.10(-7.20,-2.90) |
| Germany | HAP | 2 | 2 | 2004 | 2021 | 0.000 | -8.60(-9.20,-7.90) |
| Germany | PMP | 2 | 0 | 1990 | 1992 | 0.004 | -12.50(-19.70,-4.60) |
| Germany | PMP | 2 | 1 | 1992 | 2007 | 0.000 | -2.40(-2.80,-2.00) |
| Germany | PMP | 2 | 2 | 2007 | 2021 | 0.000 | -5.30(-5.70,-4.90) |
| Ghana | APMP | 5 | 0 | 1990 | 1995 | 0.000 | -3.60(-5.00,-2.30) |
| Ghana | APMP | 5 | 1 | 1995 | 2000 | 0.000 | 4.70(2.70,6.80) |
| Ghana | APMP | 5 | 2 | 2000 | 2005 | 0.000 | -4.30(-6.10,-2.40) |
| Ghana | APMP | 5 | 3 | 2005 | 2010 | 0.847 | 0.20(-1.70,2.10) |
| Ghana | APMP | 5 | 4 | 2010 | 2015 | 0.000 | 9.30(7.20,11.40) |
| Ghana | APMP | 5 | 5 | 2015 | 2021 | 0.000 | -5.20(-6.20,-4.20) |
| Ghana | HAP | 5 | 0 | 1990 | 2001 | 0.000 | -1.60(-1.90,-1.30) |
| Ghana | HAP | 5 | 1 | 2001 | 2006 | 0.626 | -0.30(-1.60,1.00) |
| Ghana | HAP | 5 | 2 | 2006 | 2011 | 0.000 | -3.40(-4.70,-2.20) |
| Ghana | HAP | 5 | 3 | 2011 | 2014 | 0.001 | -7.40(-11.20,-3.40) |
| Ghana | HAP | 5 | 4 | 2014 | 2017 | 0.233 | -2.40(-6.40,1.80) |
| Ghana | HAP | 5 | 5 | 2017 | 2021 | 0.000 | -5.30(-6.60,-4.10) |
| Ghana | PMP | 3 | 0 | 1990 | 1994 | 0.000 | -1.70(-2.10,-1.30) |
| Ghana | PMP | 3 | 1 | 1994 | 2005 | 0.000 | -0.90(-1.00,-0.90) |
| Ghana | PMP | 3 | 2 | 2005 | 2017 | 0.000 | -2.20(-2.30,-2.20) |
| Ghana | PMP | 3 | 3 | 2017 | 2021 | 0.000 | -5.40(-5.70,-5.00) |
| Greece | APMP | 4 | 0 | 1990 | 1997 | 0.659 | 0.50(-1.70,2.70) |
| Greece | APMP | 4 | 1 | 1997 | 2007 | 0.000 | -8.60(-9.90,-7.20) |
| Greece | APMP | 4 | 2 | 2007 | 2012 | 0.125 | -3.90(-8.70,1.20) |
| Greece | APMP | 4 | 3 | 2012 | 2015 | 0.316 | 8.30(-7.90,27.30) |
| Greece | APMP | 4 | 4 | 2015 | 2021 | 0.000 | -7.00(-9.50,-4.40) |
| Greece | HAP | 4 | 0 | 1990 | 1996 | 0.000 | -10.60(-12.90,-8.20) |
| Greece | HAP | 4 | 1 | 1996 | 2008 | 0.000 | -19.90(-20.70,-19.00) |
| Greece | HAP | 4 | 2 | 2008 | 2012 | 0.440 | -2.90(-10.30,5.10) |
| Greece | HAP | 4 | 3 | 2012 | 2015 | 0.113 | 13.30(-3.20,32.80) |
| Greece | HAP | 4 | 4 | 2015 | 2021 | 0.000 | -6.50(-9.00,-4.00) |
| Greece | PMP | 4 | 0 | 1990 | 1997 | 0.787 | 0.30(-1.90,2.50) |
| Greece | PMP | 4 | 1 | 1997 | 2007 | 0.000 | -8.60(-10.00,-7.30) |
| Greece | PMP | 4 | 2 | 2007 | 2012 | 0.125 | -3.90(-8.70,1.20) |
| Greece | PMP | 4 | 3 | 2012 | 2015 | 0.315 | 8.30(-7.90,27.30) |
| Greece | PMP | 4 | 4 | 2015 | 2021 | 0.000 | -7.00(-9.50,-4.40) |
| Greenland | APMP | 1 | 0 | 1990 | 2010 | 0.000 | -5.40(-5.60,-5.10) |
| Greenland | APMP | 1 | 1 | 2010 | 2021 | 0.000 | -2.50(-3.10,-1.90) |
| Greenland | HAP | 0 | 0 | 1990 | 2021 | 0.000 | -10.80(-11.60,-10.00) |
| Greenland | PMP | 1 | 0 | 1990 | 2010 | 0.000 | -5.40(-5.60,-5.10) |
| Greenland | PMP | 1 | 1 | 2010 | 2021 | 0.000 | -2.50(-3.10,-1.90) |
| Grenada | APMP | 5 | 0 | 1990 | 1993 | 0.217 | -1.00(-2.70,0.70) |
| Grenada | APMP | 5 | 1 | 1993 | 2000 | 0.000 | 2.40(1.90,3.00) |
| Grenada | APMP | 5 | 2 | 2000 | 2005 | 0.000 | -3.50(-4.50,-2.40) |
| Grenada | APMP | 5 | 3 | 2005 | 2009 | 0.962 | 0.00(-1.70,1.70) |
| Grenada | APMP | 5 | 4 | 2009 | 2015 | 0.000 | 4.00(3.20,4.80) |
| Grenada | APMP | 5 | 5 | 2015 | 2021 | 0.000 | -3.10(-3.60,-2.50) |
| Grenada | HAP | 5 | 0 | 1990 | 2001 | 0.000 | -14.30(-14.50,-14.10) |
| Grenada | HAP | 5 | 1 | 2001 | 2005 | 0.000 | -19.00(-20.40,-17.70) |
| Grenada | HAP | 5 | 2 | 2005 | 2009 | 0.000 | -11.00(-12.50,-9.50) |
| Grenada | HAP | 5 | 3 | 2009 | 2016 | 0.000 | -5.90(-6.40,-5.30) |
| Grenada | HAP | 5 | 4 | 2016 | 2019 | 0.000 | -13.80(-16.70,-10.90) |
| Grenada | HAP | 5 | 5 | 2019 | 2021 | 0.058 | -3.20(-6.40,0.10) |
| Grenada | PMP | 5 | 0 | 1990 | 1994 | 0.000 | -7.20(-8.00,-6.40) |
| Grenada | PMP | 5 | 1 | 1994 | 2001 | 0.000 | -2.40(-2.80,-1.90) |
| Grenada | PMP | 5 | 2 | 2001 | 2005 | 0.000 | -5.80(-7.10,-4.50) |
| Grenada | PMP | 5 | 3 | 2005 | 2009 | 0.289 | -0.70(-2.00,0.70) |
| Grenada | PMP | 5 | 4 | 2009 | 2015 | 0.000 | 3.50(2.80,4.10) |
| Grenada | PMP | 5 | 5 | 2015 | 2021 | 0.000 | -3.20(-3.60,-2.80) |
| Guam | APMP | 3 | 0 | 1990 | 1996 | 0.000 | -3.20(-4.30,-2.10) |
| Guam | APMP | 3 | 1 | 1996 | 2010 | 0.437 | -0.10(-0.50,0.20) |
| Guam | APMP | 3 | 2 | 2010 | 2015 | 0.000 | 12.60(10.20,15.00) |
| Guam | APMP | 3 | 3 | 2015 | 2021 | 0.000 | -5.20(-6.30,-4.10) |
| Guam | HAP | 2 | 0 | 1990 | 2005 | 0.000 | -2.20(-2.90,-1.50) |
| Guam | HAP | 2 | 1 | 2005 | 2016 | 0.000 | 3.20(1.90,4.50) |
| Guam | HAP | 2 | 2 | 2016 | 2021 | 0.000 | -8.40(-11.80,-5.00) |
| Guam | PMP | 3 | 0 | 1990 | 1996 | 0.000 | -3.20(-4.30,-2.00) |
| Guam | PMP | 3 | 1 | 1996 | 2010 | 0.412 | -0.10(-0.50,0.20) |
| Guam | PMP | 3 | 2 | 2010 | 2015 | 0.000 | 12.50(10.20,15.00) |
| Guam | PMP | 3 | 3 | 2015 | 2021 | 0.000 | -5.20(-6.30,-4.10) |
| Guatemala | APMP | 5 | 0 | 1990 | 1996 | 0.000 | -2.00(-2.70,-1.40) |
| Guatemala | APMP | 5 | 1 | 1996 | 2000 | 0.000 | -4.00(-5.80,-2.10) |
| Guatemala | APMP | 5 | 2 | 2000 | 2007 | 0.000 | -8.70(-9.30,-8.20) |
| Guatemala | APMP | 5 | 3 | 2007 | 2012 | 0.000 | -3.40(-4.50,-2.20) |
| Guatemala | APMP | 5 | 4 | 2012 | 2015 | 0.029 | 4.40(0.50,8.50) |
| Guatemala | APMP | 5 | 5 | 2015 | 2021 | 0.000 | -5.40(-6.00,-4.80) |
| Guatemala | HAP | 3 | 0 | 1990 | 1996 | 0.000 | -4.30(-5.30,-3.30) |
| Guatemala | HAP | 3 | 1 | 1996 | 2012 | 0.000 | -6.50(-6.80,-6.30) |
| Guatemala | HAP | 3 | 2 | 2012 | 2017 | 0.011 | -2.60(-4.50,-0.70) |
| Guatemala | HAP | 3 | 3 | 2017 | 2021 | 0.000 | -6.90(-8.70,-5.00) |
| Guatemala | PMP | 5 | 0 | 1990 | 1996 | 0.000 | -3.70(-4.50,-2.90) |
| Guatemala | PMP | 5 | 1 | 1996 | 2002 | 0.000 | -5.70(-6.80,-4.70) |
| Guatemala | PMP | 5 | 2 | 2002 | 2006 | 0.000 | -8.40(-10.60,-6.10) |
| Guatemala | PMP | 5 | 3 | 2006 | 2012 | 0.000 | -5.50(-6.50,-4.40) |
| Guatemala | PMP | 5 | 4 | 2012 | 2016 | 0.641 | -0.60(-3.00,1.90) |
| Guatemala | PMP | 5 | 5 | 2016 | 2021 | 0.000 | -6.20(-7.30,-5.20) |
| Guinea | APMP | 5 | 0 | 1990 | 1992 | 0.011 | -5.30(-9.00,-1.40) |
| Guinea | APMP | 5 | 1 | 1992 | 2001 | 0.000 | -1.40(-1.80,-0.90) |
| Guinea | APMP | 5 | 2 | 2001 | 2004 | 0.026 | -4.50(-8.20,-0.60) |
| Guinea | APMP | 5 | 3 | 2004 | 2010 | 0.000 | -2.40(-3.30,-1.60) |
| Guinea | APMP | 5 | 4 | 2010 | 2015 | 0.000 | 3.30(2.00,4.60) |
| Guinea | APMP | 5 | 5 | 2015 | 2021 | 0.000 | -4.30(-4.90,-3.60) |
| Guinea | HAP | 4 | 0 | 1990 | 1997 | 0.000 | -0.90(-1.20,-0.60) |
| Guinea | HAP | 4 | 1 | 1997 | 2009 | 0.000 | -1.90(-2.00,-1.70) |
| Guinea | HAP | 4 | 2 | 2009 | 2014 | 0.000 | -2.40(-3.10,-1.80) |
| Guinea | HAP | 4 | 3 | 2014 | 2018 | 0.032 | -1.10(-2.20,-0.10) |
| Guinea | HAP | 4 | 4 | 2018 | 2021 | 0.000 | -2.40(-3.40,-1.40) |
| Guinea | PMP | 3 | 0 | 1990 | 1998 | 0.000 | -1.10(-1.30,-1.00) |
| Guinea | PMP | 3 | 1 | 1998 | 2013 | 0.000 | -2.00(-2.10,-1.90) |
| Guinea | PMP | 3 | 2 | 2013 | 2016 | 0.315 | -0.80(-2.50,0.90) |
| Guinea | PMP | 3 | 3 | 2016 | 2021 | 0.000 | -2.30(-2.70,-1.90) |
| Guinea-Bissau | APMP | 5 | 0 | 1990 | 1994 | 0.019 | -1.00(-1.90,-0.20) |
| Guinea-Bissau | APMP | 5 | 1 | 1994 | 2000 | 0.602 | 0.20(-0.50,0.80) |
| Guinea-Bissau | APMP | 5 | 2 | 2000 | 2010 | 0.000 | -3.00(-3.20,-2.70) |
| Guinea-Bissau | APMP | 5 | 3 | 2010 | 2015 | 0.031 | 1.00(0.10,1.80) |
| Guinea-Bissau | APMP | 5 | 4 | 2015 | 2018 | 0.000 | -6.00(-8.50,-3.40) |
| Guinea-Bissau | APMP | 5 | 5 | 2018 | 2021 | 0.000 | -3.60(-4.90,-2.30) |
| Guinea-Bissau | HAP | 2 | 0 | 1990 | 2005 | 0.000 | -0.20(-0.30,-0.20) |
| Guinea-Bissau | HAP | 2 | 1 | 2005 | 2018 | 0.000 | -2.80(-2.90,-2.70) |
| Guinea-Bissau | HAP | 2 | 2 | 2018 | 2021 | 0.000 | -3.40(-4.00,-2.70) |
| Guinea-Bissau | PMP | 4 | 0 | 1990 | 2002 | 0.000 | -0.30(-0.30,-0.20) |
| Guinea-Bissau | PMP | 4 | 1 | 2002 | 2006 | 0.000 | -1.10(-1.50,-0.60) |
| Guinea-Bissau | PMP | 4 | 2 | 2006 | 2011 | 0.000 | -3.00(-3.30,-2.70) |
| Guinea-Bissau | PMP | 4 | 3 | 2011 | 2015 | 0.000 | -2.10(-2.60,-1.60) |
| Guinea-Bissau | PMP | 4 | 4 | 2015 | 2021 | 0.000 | -3.40(-3.50,-3.20) |
| Guyana | APMP | 5 | 0 | 1990 | 1995 | 0.008 | -1.30(-2.30,-0.40) |
| Guyana | APMP | 5 | 1 | 1995 | 1998 | 0.070 | 3.90(-0.40,8.40) |
| Guyana | APMP | 5 | 2 | 1998 | 2010 | 0.000 | -1.20(-1.40,-0.90) |
| Guyana | APMP | 5 | 3 | 2010 | 2014 | 0.004 | 3.50(1.30,5.70) |
| Guyana | APMP | 5 | 4 | 2014 | 2018 | 0.263 | 1.20(-0.90,3.30) |
| Guyana | APMP | 5 | 5 | 2018 | 2021 | 0.000 | -5.40(-7.40,-3.40) |
| Guyana | HAP | 1 | 0 | 1990 | 2009 | 0.000 | -5.80(-6.30,-5.40) |
| Guyana | HAP | 1 | 1 | 2009 | 2021 | 0.000 | -10.50(-11.40,-9.60) |
| Guyana | PMP | 4 | 0 | 1990 | 2000 | 0.000 | -3.70(-4.10,-3.40) |
| Guyana | PMP | 4 | 1 | 2000 | 2004 | 0.249 | -1.40(-3.90,1.10) |
| Guyana | PMP | 4 | 2 | 2004 | 2010 | 0.000 | -4.00(-5.00,-2.90) |
| Guyana | PMP | 4 | 3 | 2010 | 2018 | 0.003 | -1.10(-1.70,-0.40) |
| Guyana | PMP | 4 | 4 | 2018 | 2021 | 0.000 | -6.30(-8.70,-4.00) |
| Haiti | APMP | 4 | 0 | 1990 | 1993 | 0.000 | -3.50(-4.70,-2.40) |
| Haiti | APMP | 4 | 1 | 1993 | 1999 | 0.000 | 1.30(0.80,1.80) |
| Haiti | APMP | 4 | 2 | 1999 | 2009 | 0.000 | -0.70(-0.90,-0.50) |
| Haiti | APMP | 4 | 3 | 2009 | 2015 | 0.000 | 3.60(3.10,4.20) |
| Haiti | APMP | 4 | 4 | 2015 | 2021 | 0.000 | -1.00(-1.40,-0.60) |
| Haiti | HAP | 5 | 0 | 1990 | 1992 | 0.148 | -1.10(-2.60,0.40) |
| Haiti | HAP | 5 | 1 | 1992 | 1997 | 0.069 | 0.50(0.00,1.00) |
| Haiti | HAP | 5 | 2 | 1997 | 2001 | 0.003 | -1.30(-2.10,-0.50) |
| Haiti | HAP | 5 | 3 | 2001 | 2009 | 0.008 | 0.30(0.10,0.50) |
| Haiti | HAP | 5 | 4 | 2009 | 2015 | 0.000 | 0.80(0.50,1.20) |
| Haiti | HAP | 5 | 5 | 2015 | 2021 | 0.000 | -1.10(-1.40,-0.90) |
| Haiti | PMP | 5 | 0 | 1990 | 1992 | 0.073 | -1.40(-2.90,0.10) |
| Haiti | PMP | 5 | 1 | 1992 | 1997 | 0.051 | 0.50(0.00,1.00) |
| Haiti | PMP | 5 | 2 | 1997 | 2001 | 0.004 | -1.20(-2.00,-0.40) |
| Haiti | PMP | 5 | 3 | 2001 | 2009 | 0.035 | 0.20(0.00,0.40) |
| Haiti | PMP | 5 | 4 | 2009 | 2015 | 0.000 | 1.00(0.60,1.30) |
| Haiti | PMP | 5 | 5 | 2015 | 2021 | 0.000 | -1.10(-1.40,-0.90) |
| Honduras | APMP | 3 | 0 | 1990 | 2000 | 0.292 | 0.30(-0.20,0.80) |
| Honduras | APMP | 3 | 1 | 2000 | 2010 | 0.000 | -2.80(-3.40,-2.20) |
| Honduras | APMP | 3 | 2 | 2010 | 2015 | 0.313 | -1.00(-3.00,1.00) |
| Honduras | APMP | 3 | 3 | 2015 | 2021 | 0.000 | -6.60(-7.60,-5.50) |
| Honduras | HAP | 2 | 0 | 1990 | 1999 | 0.000 | -1.80(-2.20,-1.50) |
| Honduras | HAP | 2 | 1 | 1999 | 2011 | 0.000 | -2.70(-2.90,-2.40) |
| Honduras | HAP | 2 | 2 | 2011 | 2021 | 0.000 | -5.60(-5.90,-5.30) |
| Honduras | PMP | 3 | 0 | 1990 | 1999 | 0.000 | -1.50(-1.70,-1.20) |
| Honduras | PMP | 3 | 1 | 1999 | 2010 | 0.000 | -2.60(-2.80,-2.40) |
| Honduras | PMP | 3 | 2 | 2010 | 2016 | 0.000 | -4.30(-4.80,-3.80) |
| Honduras | PMP | 3 | 3 | 2016 | 2021 | 0.000 | -6.30(-6.80,-5.80) |
| Hungary | APMP | 2 | 0 | 1990 | 1999 | 0.000 | -8.30(-9.30,-7.40) |
| Hungary | APMP | 2 | 1 | 1999 | 2013 | 0.000 | -6.40(-7.00,-5.80) |
| Hungary | APMP | 2 | 2 | 2013 | 2021 | 0.000 | -10.60(-11.70,-9.40) |
| Hungary | HAP | 4 | 0 | 1990 | 1998 | 0.000 | -8.40(-9.70,-7.10) |
| Hungary | HAP | 4 | 1 | 1998 | 2006 | 0.000 | -16.30(-17.70,-14.80) |
| Hungary | HAP | 4 | 2 | 2006 | 2013 | 0.000 | -10.80(-12.80,-8.90) |
| Hungary | HAP | 4 | 3 | 2013 | 2018 | 0.000 | -16.80(-20.10,-13.30) |
| Hungary | HAP | 4 | 4 | 2018 | 2021 | 0.010 | -8.50(-14.20,-2.40) |
| Hungary | PMP | 3 | 0 | 1990 | 2005 | 0.000 | -8.30(-8.70,-7.80) |
| Hungary | PMP | 3 | 1 | 2005 | 2013 | 0.000 | -6.20(-7.60,-4.90) |
| Hungary | PMP | 3 | 2 | 2013 | 2018 | 0.000 | -12.30(-15.20,-9.30) |
| Hungary | PMP | 3 | 3 | 2018 | 2021 | 0.011 | -6.90(-11.70,-1.80) |
| Iceland | APMP | 4 | 0 | 1990 | 2000 | 0.000 | -5.40(-5.80,-4.90) |
| Iceland | APMP | 4 | 1 | 2000 | 2004 | 0.000 | -8.70(-11.60,-5.60) |
| Iceland | APMP | 4 | 2 | 2004 | 2008 | 0.043 | -3.30(-6.40,-0.10) |
| Iceland | APMP | 4 | 3 | 2008 | 2014 | 0.000 | -6.20(-7.50,-4.80) |
| Iceland | APMP | 4 | 4 | 2014 | 2021 | 0.000 | -3.30(-4.20,-2.50) |
| Iceland | HAP | 4 | 0 | 1990 | 1996 | 0.000 | -7.50(-10.40,-4.40) |
| Iceland | HAP | 4 | 1 | 1996 | 2010 | 0.000 | -15.60(-16.50,-14.80) |
| Iceland | HAP | 4 | 2 | 2010 | 2013 | 0.154 | 14.40(-5.40,38.30) |
| Iceland | HAP | 4 | 3 | 2013 | 2016 | 0.028 | -19.40(-33.30,-2.60) |
| Iceland | HAP | 4 | 4 | 2016 | 2021 | 0.385 | 1.80(-2.40,6.20) |
| Iceland | PMP | 4 | 0 | 1990 | 2000 | 0.000 | -5.40(-5.90,-4.90) |
| Iceland | PMP | 4 | 1 | 2000 | 2004 | 0.000 | -8.70(-11.60,-5.70) |
| Iceland | PMP | 4 | 2 | 2004 | 2008 | 0.042 | -3.30(-6.40,-0.10) |
| Iceland | PMP | 4 | 3 | 2008 | 2014 | 0.000 | -6.20(-7.50,-4.80) |
| Iceland | PMP | 4 | 4 | 2014 | 2021 | 0.000 | -3.30(-4.20,-2.50) |
| India | APMP | 2 | 0 | 1990 | 2010 | 0.673 | 0.00(-0.10,0.20) |
| India | APMP | 2 | 1 | 2010 | 2015 | 0.000 | 9.70(7.40,12.00) |
| India | APMP | 2 | 2 | 2015 | 2021 | 0.000 | -4.10(-5.20,-3.00) |
| India | HAP | 4 | 0 | 1990 | 1994 | 0.060 | -0.90(-1.80,0.00) |
| India | HAP | 4 | 1 | 1994 | 2004 | 0.000 | -2.60(-2.80,-2.30) |
| India | HAP | 4 | 2 | 2004 | 2010 | 0.000 | -1.50(-2.20,-0.90) |
| India | HAP | 4 | 3 | 2010 | 2017 | 0.000 | -5.00(-5.50,-4.60) |
| India | HAP | 4 | 4 | 2017 | 2021 | 0.000 | -2.70(-3.60,-1.80) |
| India | PMP | 3 | 0 | 1990 | 1999 | 0.000 | -1.40(-1.60,-1.20) |
| India | PMP | 3 | 1 | 1999 | 2002 | 0.010 | -3.10(-5.40,-0.80) |
| India | PMP | 3 | 2 | 2002 | 2015 | 0.000 | -1.40(-1.50,-1.20) |
| India | PMP | 3 | 3 | 2015 | 2021 | 0.000 | -3.60(-4.00,-3.20) |
| Indonesia | APMP | 4 | 0 | 1990 | 1993 | 0.000 | 5.30(2.70,8.00) |
| Indonesia | APMP | 4 | 1 | 1993 | 2001 | 0.042 | -0.70(-1.40,0.00) |
| Indonesia | APMP | 4 | 2 | 2001 | 2010 | 0.000 | -2.60(-3.10,-2.10) |
| Indonesia | APMP | 4 | 3 | 2010 | 2018 | 0.000 | 1.90(1.30,2.60) |
| Indonesia | APMP | 4 | 4 | 2018 | 2021 | 0.015 | -3.20(-5.60,-0.70) |
| Indonesia | HAP | 4 | 0 | 1990 | 2000 | 0.000 | -3.90(-4.20,-3.50) |
| Indonesia | HAP | 4 | 1 | 2000 | 2006 | 0.000 | -2.20(-3.10,-1.30) |
| Indonesia | HAP | 4 | 2 | 2006 | 2011 | 0.000 | -5.30(-6.60,-4.10) |
| Indonesia | HAP | 4 | 3 | 2011 | 2019 | 0.000 | -11.00(-11.50,-10.50) |
| Indonesia | HAP | 4 | 4 | 2019 | 2021 | 0.090 | -3.50(-7.40,0.60) |
| Indonesia | PMP | 1 | 0 | 1990 | 2008 | 0.000 | -2.80(-2.90,-2.60) |
| Indonesia | PMP | 1 | 1 | 2008 | 2021 | 0.000 | -5.50(-5.80,-5.20) |
| Iran (Islamic Republic of) | APMP | 5 | 0 | 1990 | 1999 | 0.000 | -2.20(-2.60,-1.80) |
| Iran (Islamic Republic of) | APMP | 5 | 1 | 1999 | 2005 | 0.000 | -3.90(-4.80,-2.90) |
| Iran (Islamic Republic of) | APMP | 5 | 2 | 2005 | 2010 | 0.141 | -1.00(-2.40,0.40) |
| Iran (Islamic Republic of) | APMP | 5 | 3 | 2010 | 2014 | 0.004 | -3.50(-5.60,-1.30) |
| Iran (Islamic Republic of) | APMP | 5 | 4 | 2014 | 2019 | 0.000 | -15.10(-16.30,-13.90) |
| Iran (Islamic Republic of) | APMP | 5 | 5 | 2019 | 2021 | 0.000 | -28.20(-31.30,-25.00) |
| Iran (Islamic Republic of) | HAP | 5 | 0 | 1990 | 1996 | 0.000 | -13.00(-13.80,-12.20) |
| Iran (Islamic Republic of) | HAP | 5 | 1 | 1996 | 2001 | 0.000 | -17.20(-18.60,-15.90) |
| Iran (Islamic Republic of) | HAP | 5 | 2 | 2001 | 2010 | 0.000 | -20.70(-21.20,-20.30) |
| Iran (Islamic Republic of) | HAP | 5 | 3 | 2010 | 2014 | 0.000 | -12.30(-14.60,-10.00) |
| Iran (Islamic Republic of) | HAP | 5 | 4 | 2014 | 2019 | 0.000 | -16.80(-18.10,-15.40) |
| Iran (Islamic Republic of) | HAP | 5 | 5 | 2019 | 2021 | 0.000 | -22.50(-26.50,-18.40) |
| Iran (Islamic Republic of) | PMP | 3 | 0 | 1990 | 2005 | 0.000 | -3.70(-3.90,-3.50) |
| Iran (Islamic Republic of) | PMP | 3 | 1 | 2005 | 2014 | 0.000 | -2.30(-2.80,-1.80) |
| Iran (Islamic Republic of) | PMP | 3 | 2 | 2014 | 2019 | 0.000 | -15.50(-16.80,-14.20) |
| Iran (Islamic Republic of) | PMP | 3 | 3 | 2019 | 2021 | 0.000 | -28.00(-31.40,-24.40) |
| Iraq | APMP | 5 | 0 | 1990 | 1994 | 0.000 | -5.40(-6.70,-4.10) |
| Iraq | APMP | 5 | 1 | 1994 | 1999 | 0.888 | -0.10(-1.50,1.30) |
| Iraq | APMP | 5 | 2 | 1999 | 2005 | 0.002 | -1.70(-2.70,-0.70) |
| Iraq | APMP | 5 | 3 | 2005 | 2009 | 0.093 | 1.90(-0.40,4.20) |
| Iraq | APMP | 5 | 4 | 2009 | 2017 | 0.000 | -4.80(-5.30,-4.20) |
| Iraq | APMP | 5 | 5 | 2017 | 2021 | 0.000 | -7.30(-8.60,-6.00) |
| Iraq | HAP | 4 | 0 | 1990 | 1995 | 0.000 | 11.50(8.30,14.80) |
| Iraq | HAP | 4 | 1 | 1995 | 2004 | 0.000 | -7.60(-8.90,-6.30) |
| Iraq | HAP | 4 | 2 | 2004 | 2009 | 0.000 | -16.70(-20.10,-13.30) |
| Iraq | HAP | 4 | 3 | 2009 | 2019 | 0.000 | -26.70(-27.60,-25.80) |
| Iraq | HAP | 4 | 4 | 2019 | 2021 | 0.329 | -6.00(-17.40,7.00) |
| Iraq | PMP | 4 | 0 | 1990 | 1999 | 0.000 | -1.30(-1.60,-1.00) |
| Iraq | PMP | 4 | 1 | 1999 | 2005 | 0.000 | -3.20(-3.90,-2.50) |
| Iraq | PMP | 4 | 2 | 2005 | 2009 | 0.637 | -0.40(-2.00,1.20) |
| Iraq | PMP | 4 | 3 | 2009 | 2019 | 0.000 | -5.60(-5.90,-5.40) |
| Iraq | PMP | 4 | 4 | 2019 | 2021 | 0.000 | -9.10(-12.00,-6.10) |
| Ireland | APMP | 4 | 0 | 1990 | 1992 | 0.034 | -11.50(-20.90,-1.10) |
| Ireland | APMP | 4 | 1 | 1992 | 1995 | 0.694 | 2.10(-8.60,14.20) |
| Ireland | APMP | 4 | 2 | 1995 | 2002 | 0.000 | -4.00(-5.80,-2.10) |
| Ireland | APMP | 4 | 3 | 2002 | 2006 | 0.003 | -8.80(-13.70,-3.50) |
| Ireland | APMP | 4 | 4 | 2006 | 2021 | 0.000 | -4.40(-4.80,-3.90) |
| Ireland | HAP | 1 | 0 | 1990 | 2006 | 0.000 | -21.60(-22.90,-20.30) |
| Ireland | HAP | 1 | 1 | 2006 | 2021 | 0.000 | -9.80(-11.50,-8.10) |
| Ireland | PMP | 4 | 0 | 1990 | 1992 | 0.032 | -11.60(-21.00,-1.20) |
| Ireland | PMP | 4 | 1 | 1992 | 1995 | 0.712 | 2.00(-8.80,14.10) |
| Ireland | PMP | 4 | 2 | 1995 | 2002 | 0.000 | -4.00(-5.80,-2.20) |
| Ireland | PMP | 4 | 3 | 2002 | 2006 | 0.003 | -8.80(-13.70,-3.50) |
| Ireland | PMP | 4 | 4 | 2006 | 2021 | 0.000 | -4.40(-4.80,-3.90) |
| Israel | APMP | 4 | 0 | 1990 | 2003 | 0.000 | -5.40(-5.90,-4.90) |
| Israel | APMP | 4 | 1 | 2003 | 2007 | 0.001 | -8.50(-12.90,-3.80) |
| Israel | APMP | 4 | 2 | 2007 | 2010 | 0.901 | -0.60(-10.00,9.80) |
| Israel | APMP | 4 | 3 | 2010 | 2019 | 0.000 | -6.80(-7.80,-5.70) |
| Israel | APMP | 4 | 4 | 2019 | 2021 | 0.000 | -21.20(-28.60,-12.90) |
| Israel | HAP | 4 | 0 | 1990 | 1995 | 0.000 | -16.90(-18.80,-15.00) |
| Israel | HAP | 4 | 1 | 1995 | 2002 | 0.000 | -12.10(-13.60,-10.50) |
| Israel | HAP | 4 | 2 | 2002 | 2005 | 0.000 | -20.70(-28.40,-12.20) |
| Israel | HAP | 4 | 3 | 2005 | 2017 | 0.000 | -7.60(-8.20,-6.90) |
| Israel | HAP | 4 | 4 | 2017 | 2021 | 0.000 | -19.10(-21.70,-16.50) |
| Israel | PMP | 4 | 0 | 1990 | 2003 | 0.000 | -5.40(-5.90,-4.90) |
| Israel | PMP | 4 | 1 | 2003 | 2007 | 0.002 | -8.50(-12.90,-3.80) |
| Israel | PMP | 4 | 2 | 2007 | 2010 | 0.901 | -0.60(-10.00,9.80) |
| Israel | PMP | 4 | 3 | 2010 | 2019 | 0.000 | -6.80(-7.80,-5.70) |
| Israel | PMP | 4 | 4 | 2019 | 2021 | 0.000 | -21.20(-28.60,-12.90) |
| Italy | APMP | 2 | 0 | 1990 | 2005 | 0.000 | -6.30(-6.70,-5.90) |
| Italy | APMP | 2 | 1 | 2005 | 2016 | 0.000 | -5.10(-5.80,-4.30) |
| Italy | APMP | 2 | 2 | 2016 | 2021 | 0.000 | -8.10(-10.10,-6.00) |
| Italy | HAP | 1 | 0 | 1990 | 2005 | 0.000 | -17.20(-17.80,-16.70) |
| Italy | HAP | 1 | 1 | 2005 | 2021 | 0.000 | -6.80(-7.40,-6.20) |
| Italy | PMP | 2 | 0 | 1990 | 2005 | 0.000 | -6.40(-6.80,-6.00) |
| Italy | PMP | 2 | 1 | 2005 | 2016 | 0.000 | -5.10(-5.80,-4.30) |
| Italy | PMP | 2 | 2 | 2016 | 2021 | 0.000 | -8.10(-10.10,-6.00) |
| Jamaica | APMP | 4 | 0 | 1990 | 1993 | 0.000 | 5.40(3.30,7.50) |
| Jamaica | APMP | 4 | 1 | 1993 | 2003 | 0.000 | 1.20(0.90,1.60) |
| Jamaica | APMP | 4 | 2 | 2003 | 2009 | 0.000 | -2.20(-3.00,-1.30) |
| Jamaica | APMP | 4 | 3 | 2009 | 2014 | 0.004 | 2.00(0.70,3.30) |
| Jamaica | APMP | 4 | 4 | 2014 | 2021 | 0.000 | -2.80(-3.30,-2.20) |
| Jamaica | HAP | 5 | 0 | 1990 | 1994 | 0.000 | -6.90(-7.90,-5.90) |
| Jamaica | HAP | 5 | 1 | 1994 | 2000 | 0.000 | -12.60(-13.30,-11.90) |
| Jamaica | HAP | 5 | 2 | 2000 | 2006 | 0.000 | -6.10(-6.90,-5.40) |
| Jamaica | HAP | 5 | 3 | 2006 | 2014 | 0.000 | -1.10(-1.50,-0.60) |
| Jamaica | HAP | 5 | 4 | 2014 | 2019 | 0.000 | -9.30(-10.30,-8.30) |
| Jamaica | HAP | 5 | 5 | 2019 | 2021 | 0.058 | -3.30(-6.60,0.10) |
| Jamaica | PMP | 4 | 0 | 1990 | 1993 | 0.011 | -3.00(-5.20,-0.80) |
| Jamaica | PMP | 4 | 1 | 1993 | 1999 | 0.000 | -6.70(-7.70,-5.80) |
| Jamaica | PMP | 4 | 2 | 1999 | 2008 | 0.000 | -2.90(-3.40,-2.40) |
| Jamaica | PMP | 4 | 3 | 2008 | 2014 | 0.296 | 0.50(-0.50,1.50) |
| Jamaica | PMP | 4 | 4 | 2014 | 2021 | 0.000 | -4.30(-4.80,-3.70) |
| Japan | APMP | 4 | 0 | 1990 | 1994 | 0.334 | 0.90(-1.00,3.00) |
| Japan | APMP | 4 | 1 | 1994 | 2001 | 0.000 | -8.30(-9.30,-7.30) |
| Japan | APMP | 4 | 2 | 2001 | 2010 | 0.000 | -5.40(-6.10,-4.80) |
| Japan | APMP | 4 | 3 | 2010 | 2018 | 0.021 | -1.00(-1.80,-0.20) |
| Japan | APMP | 4 | 4 | 2018 | 2021 | 0.000 | -7.20(-10.10,-4.20) |
| Japan | HAP | 4 | 0 | 1990 | 1995 | 0.000 | -15.60(-17.10,-14.20) |
| Japan | HAP | 4 | 1 | 1995 | 2000 | 0.000 | -18.60(-20.60,-16.70) |
| Japan | HAP | 4 | 2 | 2000 | 2004 | 0.000 | -15.00(-18.20,-11.70) |
| Japan | HAP | 4 | 3 | 2004 | 2009 | 0.000 | -9.80(-11.90,-7.60) |
| Japan | HAP | 4 | 4 | 2009 | 2021 | 0.000 | -7.40(-7.80,-6.90) |
| Japan | PMP | 4 | 0 | 1990 | 1994 | 0.400 | 0.80(-1.20,2.90) |
| Japan | PMP | 4 | 1 | 1994 | 2001 | 0.000 | -8.30(-9.30,-7.30) |
| Japan | PMP | 4 | 2 | 2001 | 2010 | 0.000 | -5.40(-6.10,-4.80) |
| Japan | PMP | 4 | 3 | 2010 | 2018 | 0.020 | -1.00(-1.90,-0.20) |
| Japan | PMP | 4 | 4 | 2018 | 2021 | 0.000 | -7.20(-10.10,-4.30) |
| Jordan | APMP | 3 | 0 | 1990 | 1995 | 0.656 | 0.40(-1.40,2.30) |
| Jordan | APMP | 3 | 1 | 1995 | 2000 | 0.002 | -4.30(-6.80,-1.80) |
| Jordan | APMP | 3 | 2 | 2000 | 2010 | 0.183 | -0.50(-1.30,0.30) |
| Jordan | APMP | 3 | 3 | 2010 | 2021 | 0.000 | -5.20(-5.70,-4.70) |
| Jordan | HAP | 4 | 0 | 1990 | 1999 | 0.000 | -9.10(-9.40,-8.80) |
| Jordan | HAP | 4 | 1 | 1999 | 2003 | 0.000 | -12.40(-14.00,-10.80) |
| Jordan | HAP | 4 | 2 | 2003 | 2011 | 0.000 | -17.00(-17.40,-16.60) |
| Jordan | HAP | 4 | 3 | 2011 | 2019 | 0.000 | -12.60(-13.10,-12.20) |
| Jordan | HAP | 4 | 4 | 2019 | 2021 | 0.001 | -6.40(-9.70,-2.90) |
| Jordan | PMP | 3 | 0 | 1990 | 1995 | 0.786 | 0.20(-1.60,2.10) |
| Jordan | PMP | 3 | 1 | 1995 | 2000 | 0.002 | -4.40(-6.90,-1.90) |
| Jordan | PMP | 3 | 2 | 2000 | 2010 | 0.123 | -0.60(-1.30,0.20) |
| Jordan | PMP | 3 | 3 | 2010 | 2021 | 0.000 | -5.20(-5.70,-4.70) |
| Kazakhstan | APMP | 5 | 0 | 1990 | 1992 | 0.189 | 4.50(-2.40,11.90) |
| Kazakhstan | APMP | 5 | 1 | 1992 | 2000 | 0.000 | -3.50(-4.40,-2.60) |
| Kazakhstan | APMP | 5 | 2 | 2000 | 2006 | 0.005 | 2.40(0.80,3.90) |
| Kazakhstan | APMP | 5 | 3 | 2006 | 2009 | 0.016 | 9.10(1.90,16.80) |
| Kazakhstan | APMP | 5 | 4 | 2009 | 2017 | 0.000 | -9.80(-10.60,-9.00) |
| Kazakhstan | APMP | 5 | 5 | 2017 | 2021 | 0.001 | -4.20(-6.20,-2.10) |
| Kazakhstan | HAP | 5 | 0 | 1990 | 2000 | 0.000 | 5.90(5.30,6.60) |
| Kazakhstan | HAP | 5 | 1 | 2000 | 2003 | 0.039 | -8.20(-15.30,-0.50) |
| Kazakhstan | HAP | 5 | 2 | 2003 | 2009 | 0.000 | -19.20(-20.60,-17.70) |
| Kazakhstan | HAP | 5 | 3 | 2009 | 2014 | 0.000 | -31.70(-33.50,-30.00) |
| Kazakhstan | HAP | 5 | 4 | 2014 | 2017 | 0.000 | -20.30(-26.50,-13.60) |
| Kazakhstan | HAP | 5 | 5 | 2017 | 2021 | 0.000 | -6.70(-9.10,-4.30) |
| Kazakhstan | PMP | 4 | 0 | 1990 | 1998 | 0.010 | 1.10(0.30,1.80) |
| Kazakhstan | PMP | 4 | 1 | 1998 | 2006 | 0.000 | -2.70(-3.60,-1.70) |
| Kazakhstan | PMP | 4 | 2 | 2006 | 2009 | 0.493 | 2.40(-4.60,9.90) |
| Kazakhstan | PMP | 4 | 3 | 2009 | 2016 | 0.000 | -11.30(-12.30,-10.20) |
| Kazakhstan | PMP | 4 | 4 | 2016 | 2021 | 0.000 | -5.10(-6.60,-3.60) |
| Kenya | APMP | 3 | 0 | 1990 | 2001 | 0.000 | 2.70(1.80,3.60) |
| Kenya | APMP | 3 | 1 | 2001 | 2005 | 0.228 | -3.90(-10.10,2.70) |
| Kenya | APMP | 3 | 2 | 2005 | 2014 | 0.000 | 4.60(3.10,6.10) |
| Kenya | APMP | 3 | 3 | 2014 | 2021 | 0.000 | -3.70(-5.40,-2.00) |
| Kenya | HAP | 5 | 0 | 1990 | 1995 | 0.548 | 0.10(-0.20,0.30) |
| Kenya | HAP | 5 | 1 | 1995 | 1999 | 0.050 | -0.50(-1.10,0.00) |
| Kenya | HAP | 5 | 2 | 1999 | 2003 | 0.883 | 0.00(-0.60,0.50) |
| Kenya | HAP | 5 | 3 | 2003 | 2014 | 0.000 | -0.50(-0.50,-0.40) |
| Kenya | HAP | 5 | 4 | 2014 | 2017 | 0.045 | -1.10(-2.20,0.00) |
| Kenya | HAP | 5 | 5 | 2017 | 2021 | 0.000 | -3.10(-3.50,-2.80) |
| Kenya | PMP | 4 | 0 | 1990 | 2002 | 0.421 | 0.00(-0.10,0.00) |
| Kenya | PMP | 4 | 1 | 2002 | 2006 | 0.030 | -0.60(-1.20,-0.10) |
| Kenya | PMP | 4 | 2 | 2006 | 2014 | 0.757 | 0.00(-0.10,0.20) |
| Kenya | PMP | 4 | 3 | 2014 | 2017 | 0.012 | -1.40(-2.50,-0.40) |
| Kenya | PMP | 4 | 4 | 2017 | 2021 | 0.000 | -3.10(-3.40,-2.80) |
| Kiribati | APMP | 5 | 0 | 1990 | 1995 | 0.000 | -1.40(-1.70,-1.20) |
| Kiribati | APMP | 5 | 1 | 1995 | 2003 | 0.900 | 0.00(-0.10,0.20) |
| Kiribati | APMP | 5 | 2 | 2003 | 2009 | 0.000 | -3.30(-3.50,-3.00) |
| Kiribati | APMP | 5 | 3 | 2009 | 2015 | 0.000 | 0.50(0.30,0.80) |
| Kiribati | APMP | 5 | 4 | 2015 | 2019 | 0.000 | 4.30(3.70,4.90) |
| Kiribati | APMP | 5 | 5 | 2019 | 2021 | 0.010 | -1.60(-2.70,-0.50) |
| Kiribati | HAP | 5 | 0 | 1990 | 1993 | 0.002 | -1.20(-1.90,-0.50) |
| Kiribati | HAP | 5 | 1 | 1993 | 2004 | 0.000 | -2.20(-2.30,-2.10) |
| Kiribati | HAP | 5 | 2 | 2004 | 2008 | 0.000 | -3.90(-4.60,-3.20) |
| Kiribati | HAP | 5 | 3 | 2008 | 2015 | 0.000 | -2.00(-2.30,-1.80) |
| Kiribati | HAP | 5 | 4 | 2015 | 2019 | 0.000 | -4.40(-5.10,-3.70) |
| Kiribati | HAP | 5 | 5 | 2019 | 2021 | 0.093 | -1.20(-2.60,0.20) |
| Kiribati | PMP | 5 | 0 | 1990 | 1993 | 0.001 | -1.30(-1.90,-0.60) |
| Kiribati | PMP | 5 | 1 | 1993 | 2004 | 0.000 | -2.10(-2.20,-2.00) |
| Kiribati | PMP | 5 | 2 | 2004 | 2008 | 0.000 | -3.90(-4.60,-3.30) |
| Kiribati | PMP | 5 | 3 | 2008 | 2015 | 0.000 | -2.00(-2.20,-1.70) |
| Kiribati | PMP | 5 | 4 | 2015 | 2019 | 0.000 | -3.90(-4.60,-3.30) |
| Kiribati | PMP | 5 | 5 | 2019 | 2021 | 0.073 | -1.20(-2.50,0.10) |
| Kuwait | APMP | 1 | 0 | 1990 | 2015 | 0.483 | -0.20(-0.80,0.40) |
| Kuwait | APMP | 1 | 1 | 2015 | 2021 | 0.059 | -4.90(-9.70,0.20) |
| Kuwait | HAP | 1 | 0 | 1990 | 1998 | 0.000 | -15.20(-19.00,-11.20) |
| Kuwait | HAP | 1 | 1 | 1998 | 2021 | 0.000 | -8.60(-9.50,-7.80) |
| Kuwait | PMP | 1 | 0 | 1990 | 2015 | 0.477 | -0.20(-0.80,0.40) |
| Kuwait | PMP | 1 | 1 | 2015 | 2021 | 0.059 | -4.90(-9.70,0.20) |
| Kyrgyzstan | APMP | 3 | 0 | 1990 | 1999 | 0.000 | -8.80(-9.60,-8.10) |
| Kyrgyzstan | APMP | 3 | 1 | 1999 | 2011 | 0.011 | 0.80(0.20,1.50) |
| Kyrgyzstan | APMP | 3 | 2 | 2011 | 2014 | 0.079 | 8.60(-1.00,19.20) |
| Kyrgyzstan | APMP | 3 | 3 | 2014 | 2021 | 0.005 | 1.90(0.60,3.10) |
| Kyrgyzstan | HAP | 3 | 0 | 1990 | 1998 | 0.000 | -2.20(-2.90,-1.60) |
| Kyrgyzstan | HAP | 3 | 1 | 1998 | 2006 | 0.000 | 3.90(3.00,4.80) |
| Kyrgyzstan | HAP | 3 | 2 | 2006 | 2017 | 0.000 | -7.50(-8.00,-7.10) |
| Kyrgyzstan | HAP | 3 | 3 | 2017 | 2021 | 0.049 | -2.00(-3.90,0.00) |
| Kyrgyzstan | PMP | 3 | 0 | 1990 | 1998 | 0.000 | -3.70(-4.60,-2.90) |
| Kyrgyzstan | PMP | 3 | 1 | 1998 | 2006 | 0.000 | 3.00(1.90,4.10) |
| Kyrgyzstan | PMP | 3 | 2 | 2006 | 2017 | 0.000 | -5.20(-5.80,-4.60) |
| Kyrgyzstan | PMP | 3 | 3 | 2017 | 2021 | 0.993 | 0.00(-2.50,2.60) |
| Lao People's Democratic Republic | APMP | 5 | 0 | 1990 | 2000 | 0.000 | 0.20(0.10,0.20) |
| Lao People's Democratic Republic | APMP | 5 | 1 | 2000 | 2005 | 0.000 | 1.10(0.80,1.40) |
| Lao People's Democratic Republic | APMP | 5 | 2 | 2005 | 2012 | 0.000 | -0.80(-0.90,-0.60) |
| Lao People's Democratic Republic | APMP | 5 | 3 | 2012 | 2015 | 0.066 | 0.80(-0.10,1.70) |
| Lao People's Democratic Republic | APMP | 5 | 4 | 2015 | 2019 | 0.000 | 6.80(6.30,7.20) |
| Lao People's Democratic Republic | APMP | 5 | 5 | 2019 | 2021 | 0.000 | -2.70(-3.60,-1.80) |
| Lao People's Democratic Republic | HAP | 4 | 0 | 1990 | 1998 | 0.000 | -0.50(-0.70,-0.40) |
| Lao People's Democratic Republic | HAP | 4 | 1 | 1998 | 2005 | 0.000 | -2.20(-2.40,-2.00) |
| Lao People's Democratic Republic | HAP | 4 | 2 | 2005 | 2011 | 0.000 | -4.00(-4.20,-3.80) |
| Lao People's Democratic Republic | HAP | 4 | 3 | 2011 | 2019 | 0.000 | -6.20(-6.30,-6.00) |
| Lao People's Democratic Republic | HAP | 4 | 4 | 2019 | 2021 | 0.000 | -3.00(-4.00,-2.00) |
| Lao People's Democratic Republic | PMP | 5 | 0 | 1990 | 1998 | 0.000 | -0.50(-0.60,-0.40) |
| Lao People's Democratic Republic | PMP | 5 | 1 | 1998 | 2005 | 0.000 | -2.00(-2.10,-1.80) |
| Lao People's Democratic Republic | PMP | 5 | 2 | 2005 | 2010 | 0.000 | -3.50(-3.80,-3.30) |
| Lao People's Democratic Republic | PMP | 5 | 3 | 2010 | 2014 | 0.000 | -5.10(-5.50,-4.70) |
| Lao People's Democratic Republic | PMP | 5 | 4 | 2014 | 2019 | 0.000 | -4.20(-4.40,-4.00) |
| Lao People's Democratic Republic | PMP | 5 | 5 | 2019 | 2021 | 0.000 | -2.50(-3.20,-1.70) |
| Latvia | APMP | 4 | 0 | 1990 | 1997 | 0.074 | -2.10(-4.40,0.20) |
| Latvia | APMP | 4 | 1 | 1997 | 2001 | 0.015 | -10.80(-18.40,-2.40) |
| Latvia | APMP | 4 | 2 | 2001 | 2011 | 0.000 | -4.00(-5.50,-2.40) |
| Latvia | APMP | 4 | 3 | 2011 | 2015 | 0.000 | -17.10(-24.20,-9.30) |
| Latvia | APMP | 4 | 4 | 2015 | 2021 | 0.007 | -4.30(-7.10,-1.40) |
| Latvia | HAP | 3 | 0 | 1990 | 1997 | 0.000 | 8.50(5.50,11.60) |
| Latvia | HAP | 3 | 1 | 1997 | 2002 | 0.008 | -8.90(-14.70,-2.70) |
| Latvia | HAP | 3 | 2 | 2002 | 2016 | 0.000 | -21.80(-22.70,-20.90) |
| Latvia | HAP | 3 | 3 | 2016 | 2021 | 0.000 | -9.30(-13.40,-5.00) |
| Latvia | PMP | 3 | 0 | 1990 | 1997 | 0.883 | 0.20(-2.30,2.70) |
| Latvia | PMP | 3 | 1 | 1997 | 2012 | 0.000 | -8.60(-9.40,-7.80) |
| Latvia | PMP | 3 | 2 | 2012 | 2015 | 0.030 | -18.60(-32.20,-2.10) |
| Latvia | PMP | 3 | 3 | 2015 | 2021 | 0.006 | -4.40(-7.40,-1.40) |
| Lebanon | APMP | 5 | 0 | 1990 | 1994 | 0.582 | 0.60(-1.70,2.90) |
| Lebanon | APMP | 5 | 1 | 1994 | 2005 | 0.000 | -3.60(-4.20,-3.10) |
| Lebanon | APMP | 5 | 2 | 2005 | 2010 | 0.193 | -1.40(-3.70,0.80) |
| Lebanon | APMP | 5 | 3 | 2010 | 2014 | 0.000 | -9.40(-12.60,-6.00) |
| Lebanon | APMP | 5 | 4 | 2014 | 2017 | 0.692 | -1.40(-8.20,6.00) |
| Lebanon | APMP | 5 | 5 | 2017 | 2021 | 0.000 | -7.10(-9.20,-4.90) |
| Lebanon | HAP | 3 | 0 | 1990 | 1996 | 0.000 | -6.90(-7.90,-5.80) |
| Lebanon | HAP | 3 | 1 | 1996 | 2005 | 0.000 | -14.90(-15.60,-14.30) |
| Lebanon | HAP | 3 | 2 | 2005 | 2014 | 0.000 | -20.50(-21.10,-19.90) |
| Lebanon | HAP | 3 | 3 | 2014 | 2021 | 0.000 | -9.10(-9.90,-8.30) |
| Lebanon | PMP | 5 | 0 | 1990 | 1994 | 0.839 | -0.20(-2.50,2.10) |
| Lebanon | PMP | 5 | 1 | 1994 | 2005 | 0.000 | -4.40(-4.90,-3.80) |
| Lebanon | PMP | 5 | 2 | 2005 | 2010 | 0.106 | -1.80(-4.00,0.40) |
| Lebanon | PMP | 5 | 3 | 2010 | 2014 | 0.000 | -9.40(-12.60,-6.10) |
| Lebanon | PMP | 5 | 4 | 2014 | 2017 | 0.675 | -1.40(-8.30,5.90) |
| Lebanon | PMP | 5 | 5 | 2017 | 2021 | 0.000 | -7.10(-9.20,-4.90) |
| Lesotho | APMP | 3 | 0 | 1990 | 1995 | 0.037 | 1.90(0.10,3.60) |
| Lesotho | APMP | 3 | 1 | 1995 | 2005 | 0.000 | -3.10(-3.80,-2.40) |
| Lesotho | APMP | 3 | 2 | 2005 | 2010 | 0.000 | 6.90(4.30,9.50) |
| Lesotho | APMP | 3 | 3 | 2010 | 2021 | 0.752 | 0.10(-0.40,0.60) |
| Lesotho | HAP | 4 | 0 | 1990 | 1996 | 0.321 | -0.30(-1.00,0.40) |
| Lesotho | HAP | 4 | 1 | 1996 | 2002 | 0.000 | -2.80(-3.60,-1.90) |
| Lesotho | HAP | 4 | 2 | 2002 | 2011 | 0.045 | 0.50(0.00,0.90) |
| Lesotho | HAP | 4 | 3 | 2011 | 2014 | 0.087 | -3.50(-7.40,0.60) |
| Lesotho | HAP | 4 | 4 | 2014 | 2021 | 0.001 | -1.00(-1.50,-0.40) |
| Lesotho | PMP | 5 | 0 | 1990 | 1996 | 0.625 | -0.10(-0.70,0.40) |
| Lesotho | PMP | 5 | 1 | 1996 | 2001 | 0.000 | -3.00(-4.00,-2.00) |
| Lesotho | PMP | 5 | 2 | 2001 | 2006 | 0.238 | -0.60(-1.60,0.40) |
| Lesotho | PMP | 5 | 3 | 2006 | 2009 | 0.072 | 3.00(-0.30,6.50) |
| Lesotho | PMP | 5 | 4 | 2009 | 2014 | 0.001 | -2.10(-3.10,-1.10) |
| Lesotho | PMP | 5 | 5 | 2014 | 2021 | 0.000 | -0.90(-1.40,-0.50) |
| Liberia | APMP | 4 | 0 | 1990 | 1994 | 0.000 | -13.80(-16.70,-10.70) |
| Liberia | APMP | 4 | 1 | 1994 | 2004 | 0.005 | 1.60(0.50,2.60) |
| Liberia | APMP | 4 | 2 | 2004 | 2010 | 0.000 | -7.00(-9.30,-4.70) |
| Liberia | APMP | 4 | 3 | 2010 | 2014 | 0.000 | 12.30(6.20,18.80) |
| Liberia | APMP | 4 | 4 | 2014 | 2021 | 0.000 | -5.60(-7.00,-4.20) |
| Liberia | HAP | 3 | 0 | 1990 | 1993 | 0.004 | -5.80(-9.30,-2.10) |
| Liberia | HAP | 3 | 1 | 1993 | 2004 | 0.000 | -2.10(-2.70,-1.60) |
| Liberia | HAP | 3 | 2 | 2004 | 2007 | 0.188 | -4.90(-11.90,2.70) |
| Liberia | HAP | 3 | 3 | 2007 | 2021 | 0.000 | -1.20(-1.50,-0.80) |
| Liberia | PMP | 4 | 0 | 1990 | 1993 | 0.001 | -6.80(-10.10,-3.40) |
| Liberia | PMP | 4 | 1 | 1993 | 2005 | 0.000 | -2.00(-2.50,-1.50) |
| Liberia | PMP | 4 | 2 | 2005 | 2008 | 0.092 | -5.90(-12.50,1.10) |
| Liberia | PMP | 4 | 3 | 2008 | 2014 | 0.506 | 0.50(-1.10,2.20) |
| Liberia | PMP | 4 | 4 | 2014 | 2021 | 0.000 | -2.20(-3.10,-1.20) |
| Libya | APMP | 3 | 0 | 1990 | 2001 | 0.000 | -4.30(-5.00,-3.50) |
| Libya | APMP | 3 | 1 | 2001 | 2014 | 0.002 | -1.10(-1.70,-0.40) |
| Libya | APMP | 3 | 2 | 2014 | 2017 | 0.166 | 8.00(-3.40,20.70) |
| Libya | APMP | 3 | 3 | 2017 | 2021 | 0.001 | -6.10(-9.30,-2.70) |
| Libya | HAP | 4 | 0 | 1990 | 2002 | 0.000 | -12.10(-12.60,-11.60) |
| Libya | HAP | 4 | 1 | 2002 | 2008 | 0.000 | -16.10(-17.90,-14.20) |
| Libya | HAP | 4 | 2 | 2008 | 2011 | 0.088 | -8.10(-16.80,1.40) |
| Libya | HAP | 4 | 3 | 2011 | 2017 | 0.000 | 27.50(24.70,30.30) |
| Libya | HAP | 4 | 4 | 2017 | 2021 | 0.628 | 0.70(-2.40,3.90) |
| Libya | PMP | 3 | 0 | 1990 | 2001 | 0.000 | -4.30(-5.00,-3.60) |
| Libya | PMP | 3 | 1 | 2001 | 2014 | 0.002 | -1.10(-1.80,-0.50) |
| Libya | PMP | 3 | 2 | 2014 | 2017 | 0.159 | 8.10(-3.20,20.70) |
| Libya | PMP | 3 | 3 | 2017 | 2021 | 0.001 | -6.10(-9.30,-2.70) |
| Lithuania | APMP | 4 | 0 | 1990 | 1992 | 0.001 | 22.80(9.50,37.80) |
| Lithuania | APMP | 4 | 1 | 1992 | 1999 | 0.000 | -15.90(-17.50,-14.20) |
| Lithuania | APMP | 4 | 2 | 1999 | 2011 | 0.000 | -7.00(-7.70,-6.30) |
| Lithuania | APMP | 4 | 3 | 2011 | 2014 | 0.048 | -11.00(-20.70,-0.10) |
| Lithuania | APMP | 4 | 4 | 2014 | 2021 | 0.000 | -5.10(-6.50,-3.60) |
| Lithuania | HAP | 4 | 0 | 1990 | 1992 | 0.000 | 36.40(24.50,49.40) |
| Lithuania | HAP | 4 | 1 | 1992 | 2001 | 0.000 | -14.70(-15.50,-13.80) |
| Lithuania | HAP | 4 | 2 | 2001 | 2008 | 0.000 | -21.00(-22.20,-19.80) |
| Lithuania | HAP | 4 | 3 | 2008 | 2018 | 0.000 | -19.40(-20.00,-18.70) |
| Lithuania | HAP | 4 | 4 | 2018 | 2021 | 0.001 | -8.30(-12.40,-4.00) |
| Lithuania | PMP | 3 | 0 | 1990 | 1992 | 0.001 | 24.20(10.50,39.50) |
| Lithuania | PMP | 3 | 1 | 1992 | 1999 | 0.000 | -15.60(-17.20,-13.90) |
| Lithuania | PMP | 3 | 2 | 1999 | 2017 | 0.000 | -8.30(-8.70,-7.90) |
| Lithuania | PMP | 3 | 3 | 2017 | 2021 | 0.035 | -3.90(-7.40,-0.30) |
| Luxembourg | APMP | 1 | 0 | 1990 | 2008 | 0.000 | -7.00(-8.00,-6.00) |
| Luxembourg | APMP | 1 | 1 | 2008 | 2021 | 0.007 | -2.50(-4.10,-0.80) |
| Luxembourg | HAP | 2 | 0 | 1990 | 2005 | 0.000 | -11.90(-13.80,-9.90) |
| Luxembourg | HAP | 2 | 1 | 2005 | 2009 | 0.048 | -23.50(-41.20,-0.30) |
| Luxembourg | HAP | 2 | 2 | 2009 | 2021 | 0.003 | -4.90(-7.90,-1.90) |
| Luxembourg | PMP | 1 | 0 | 1990 | 2008 | 0.000 | -7.00(-8.00,-6.00) |
| Luxembourg | PMP | 1 | 1 | 2008 | 2021 | 0.007 | -2.50(-4.10,-0.80) |
| Madagascar | APMP | 5 | 0 | 1990 | 1995 | 0.000 | -4.00(-4.30,-3.60) |
| Madagascar | APMP | 5 | 1 | 1995 | 2000 | 0.000 | 4.90(4.40,5.50) |
| Madagascar | APMP | 5 | 2 | 2000 | 2013 | 0.000 | 3.30(3.20,3.40) |
| Madagascar | APMP | 5 | 3 | 2013 | 2016 | 0.459 | -0.60(-2.10,1.00) |
| Madagascar | APMP | 5 | 4 | 2016 | 2019 | 0.000 | -7.80(-9.30,-6.30) |
| Madagascar | APMP | 5 | 5 | 2019 | 2021 | 0.005 | -2.40(-4.00,-0.90) |
| Madagascar | HAP | 5 | 0 | 1990 | 1996 | 0.000 | -0.90(-1.10,-0.70) |
| Madagascar | HAP | 5 | 1 | 1996 | 2001 | 0.875 | 0.00(-0.30,0.30) |
| Madagascar | HAP | 5 | 2 | 2001 | 2005 | 0.001 | 1.00(0.50,1.50) |
| Madagascar | HAP | 5 | 3 | 2005 | 2010 | 0.160 | -0.20(-0.50,0.10) |
| Madagascar | HAP | 5 | 4 | 2010 | 2016 | 0.000 | -0.80(-1.10,-0.60) |
| Madagascar | HAP | 5 | 5 | 2016 | 2021 | 0.000 | -2.50(-2.70,-2.30) |
| Madagascar | PMP | 5 | 0 | 1990 | 1996 | 0.000 | -0.90(-1.10,-0.80) |
| Madagascar | PMP | 5 | 1 | 1996 | 2001 | 0.252 | 0.20(-0.10,0.50) |
| Madagascar | PMP | 5 | 2 | 2001 | 2005 | 0.000 | 1.00(0.60,1.50) |
| Madagascar | PMP | 5 | 3 | 2005 | 2010 | 0.592 | -0.10(-0.40,0.20) |
| Madagascar | PMP | 5 | 4 | 2010 | 2016 | 0.000 | -0.80(-1.00,-0.60) |
| Madagascar | PMP | 5 | 5 | 2016 | 2021 | 0.000 | -2.70(-2.90,-2.50) |
| Malawi | APMP | 4 | 0 | 1990 | 1996 | 0.000 | -2.30(-3.30,-1.30) |
| Malawi | APMP | 4 | 1 | 1996 | 1999 | 0.451 | 2.20(-3.70,8.40) |
| Malawi | APMP | 4 | 2 | 1999 | 2005 | 0.038 | -1.40(-2.70,-0.10) |
| Malawi | APMP | 4 | 3 | 2005 | 2013 | 0.634 | 0.20(-0.60,1.00) |
| Malawi | APMP | 4 | 4 | 2013 | 2021 | 0.000 | -4.30(-4.90,-3.70) |
| Malawi | HAP | 3 | 0 | 1990 | 1994 | 0.786 | -0.10(-0.70,0.50) |
| Malawi | HAP | 3 | 1 | 1994 | 1998 | 0.000 | -2.70(-3.60,-1.80) |
| Malawi | HAP | 3 | 2 | 1998 | 2012 | 0.000 | -1.50(-1.60,-1.40) |
| Malawi | HAP | 3 | 3 | 2012 | 2021 | 0.000 | -2.90(-3.10,-2.80) |
| Malawi | PMP | 3 | 0 | 1990 | 1994 | 0.471 | -0.20(-0.80,0.40) |
| Malawi | PMP | 3 | 1 | 1994 | 1998 | 0.000 | -2.60(-3.50,-1.60) |
| Malawi | PMP | 3 | 2 | 1998 | 2012 | 0.000 | -1.40(-1.50,-1.30) |
| Malawi | PMP | 3 | 3 | 2012 | 2021 | 0.000 | -3.00(-3.10,-2.80) |
| Malaysia | APMP | 3 | 0 | 1990 | 2002 | 0.000 | -8.20(-9.00,-7.30) |
| Malaysia | APMP | 3 | 1 | 2002 | 2013 | 0.000 | -3.00(-4.20,-1.80) |
| Malaysia | APMP | 3 | 2 | 2013 | 2017 | 0.263 | 4.50(-3.50,13.20) |
| Malaysia | APMP | 3 | 3 | 2017 | 2021 | 0.003 | -7.80(-12.30,-3.00) |
| Malaysia | HAP | 1 | 0 | 1990 | 2002 | 0.000 | -21.30(-22.20,-20.50) |
| Malaysia | HAP | 1 | 1 | 2002 | 2021 | 0.000 | -10.10(-10.60,-9.60) |
| Malaysia | PMP | 3 | 0 | 1990 | 2002 | 0.000 | -8.70(-9.60,-7.90) |
| Malaysia | PMP | 3 | 1 | 2002 | 2013 | 0.000 | -3.10(-4.20,-1.90) |
| Malaysia | PMP | 3 | 2 | 2013 | 2017 | 0.258 | 4.40(-3.40,12.80) |
| Malaysia | PMP | 3 | 3 | 2017 | 2021 | 0.002 | -7.80(-12.20,-3.20) |
| Maldives | APMP | 5 | 0 | 1990 | 1994 | 0.370 | 0.80(-1.10,2.80) |
| Maldives | APMP | 5 | 1 | 1994 | 2005 | 0.000 | -2.50(-2.90,-2.00) |
| Maldives | APMP | 5 | 2 | 2005 | 2011 | 0.000 | -5.30(-6.60,-4.00) |
| Maldives | APMP | 5 | 3 | 2011 | 2014 | 0.008 | -8.50(-14.00,-2.70) |
| Maldives | APMP | 5 | 4 | 2014 | 2018 | 0.161 | -2.10(-5.10,0.90) |
| Maldives | APMP | 5 | 5 | 2018 | 2021 | 0.001 | -5.60(-8.40,-2.60) |
| Maldives | HAP | 4 | 0 | 1990 | 1996 | 0.000 | -8.00(-8.50,-7.40) |
| Maldives | HAP | 4 | 1 | 1996 | 2002 | 0.000 | -13.20(-13.90,-12.50) |
| Maldives | HAP | 4 | 2 | 2002 | 2010 | 0.000 | -18.60(-19.00,-18.30) |
| Maldives | HAP | 4 | 3 | 2010 | 2019 | 0.000 | -17.00(-17.30,-16.60) |
| Maldives | HAP | 4 | 4 | 2019 | 2021 | 0.001 | -6.30(-9.40,-3.00) |
| Maldives | PMP | 2 | 0 | 1990 | 1996 | 0.000 | -6.20(-7.10,-5.20) |
| Maldives | PMP | 2 | 1 | 1996 | 2014 | 0.000 | -10.20(-10.40,-10.00) |
| Maldives | PMP | 2 | 2 | 2014 | 2021 | 0.000 | -4.80(-5.60,-4.10) |
| Mali | APMP | 5 | 0 | 1990 | 1996 | 0.000 | -2.20(-2.50,-1.90) |
| Mali | APMP | 5 | 1 | 1996 | 1999 | 0.032 | 1.90(0.20,3.60) |
| Mali | APMP | 5 | 2 | 1999 | 2005 | 0.004 | -0.60(-1.00,-0.20) |
| Mali | APMP | 5 | 3 | 2005 | 2015 | 0.000 | 1.80(1.60,1.90) |
| Mali | APMP | 5 | 4 | 2015 | 2019 | 0.000 | -6.20(-7.00,-5.50) |
| Mali | APMP | 5 | 5 | 2019 | 2021 | 0.033 | -1.80(-3.40,-0.20) |
| Mali | HAP | 3 | 0 | 1990 | 1994 | 0.000 | -0.90(-1.20,-0.50) |
| Mali | HAP | 3 | 1 | 1994 | 2001 | 0.000 | -2.10(-2.20,-1.90) |
| Mali | HAP | 3 | 2 | 2001 | 2008 | 0.000 | -0.70(-0.90,-0.50) |
| Mali | HAP | 3 | 3 | 2008 | 2021 | 0.000 | -1.40(-1.50,-1.40) |
| Mali | PMP | 5 | 0 | 1990 | 1994 | 0.000 | -1.10(-1.40,-0.80) |
| Mali | PMP | 5 | 1 | 1994 | 1997 | 0.001 | -2.00(-3.00,-1.00) |
| Mali | PMP | 5 | 2 | 1997 | 2002 | 0.000 | -1.50(-1.80,-1.20) |
| Mali | PMP | 5 | 3 | 2002 | 2008 | 0.001 | -0.50(-0.70,-0.20) |
| Mali | PMP | 5 | 4 | 2008 | 2015 | 0.000 | -0.90(-1.10,-0.70) |
| Mali | PMP | 5 | 5 | 2015 | 2021 | 0.000 | -2.10(-2.30,-2.00) |
| Malta | APMP | 4 | 0 | 1990 | 1995 | 0.137 | -1.30(-3.10,0.50) |
| Malta | APMP | 4 | 1 | 1995 | 2003 | 0.000 | -5.70(-6.70,-4.60) |
| Malta | APMP | 4 | 2 | 2003 | 2006 | 0.557 | 2.30(-5.60,11.00) |
| Malta | APMP | 4 | 3 | 2006 | 2019 | 0.000 | -4.60(-5.00,-4.10) |
| Malta | APMP | 4 | 4 | 2019 | 2021 | 0.016 | -9.80(-16.80,-2.20) |
| Malta | HAP | 3 | 0 | 1990 | 1995 | 0.000 | -15.60(-18.70,-12.50) |
| Malta | HAP | 3 | 1 | 1995 | 2000 | 0.000 | -22.00(-25.90,-17.90) |
| Malta | HAP | 3 | 2 | 2000 | 2013 | 0.000 | -14.80(-15.70,-14.00) |
| Malta | HAP | 3 | 3 | 2013 | 2021 | 0.000 | -10.70(-12.20,-9.10) |
| Malta | PMP | 4 | 0 | 1990 | 1995 | 0.068 | -1.70(-3.40,0.10) |
| Malta | PMP | 4 | 1 | 1995 | 2003 | 0.000 | -5.80(-6.80,-4.80) |
| Malta | PMP | 4 | 2 | 2003 | 2006 | 0.559 | 2.30(-5.60,11.00) |
| Malta | PMP | 4 | 3 | 2006 | 2019 | 0.000 | -4.60(-5.00,-4.10) |
| Malta | PMP | 4 | 4 | 2019 | 2021 | 0.016 | -9.80(-16.80,-2.20) |
| Marshall Islands | APMP | 5 | 0 | 1990 | 1994 | 0.002 | 1.50(0.70,2.40) |
| Marshall Islands | APMP | 5 | 1 | 1994 | 2001 | 0.000 | 3.40(2.90,3.90) |
| Marshall Islands | APMP | 5 | 2 | 2001 | 2009 | 0.000 | -4.10(-4.40,-3.80) |
| Marshall Islands | APMP | 5 | 3 | 2009 | 2015 | 0.000 | -1.60(-2.20,-1.00) |
| Marshall Islands | APMP | 5 | 4 | 2015 | 2019 | 0.003 | 2.30(0.90,3.60) |
| Marshall Islands | APMP | 5 | 5 | 2019 | 2021 | 0.053 | -2.60(-5.10,0.00) |
| Marshall Islands | HAP | 5 | 0 | 1990 | 1995 | 0.001 | -1.40(-2.00,-0.70) |
| Marshall Islands | HAP | 5 | 1 | 1995 | 2000 | 0.137 | 0.70(-0.30,1.70) |
| Marshall Islands | HAP | 5 | 2 | 2000 | 2006 | 0.000 | -3.40(-4.10,-2.80) |
| Marshall Islands | HAP | 5 | 3 | 2006 | 2011 | 0.399 | 0.40(-0.60,1.40) |
| Marshall Islands | HAP | 5 | 4 | 2011 | 2016 | 0.000 | -2.20(-3.10,-1.30) |
| Marshall Islands | HAP | 5 | 5 | 2016 | 2021 | 0.000 | -4.50(-5.20,-3.90) |
| Marshall Islands | PMP | 5 | 0 | 1990 | 1995 | 0.001 | -0.90(-1.30,-0.40) |
| Marshall Islands | PMP | 5 | 1 | 1995 | 2001 | 0.005 | 0.70(0.20,1.10) |
| Marshall Islands | PMP | 5 | 2 | 2001 | 2006 | 0.000 | -3.90(-4.50,-3.30) |
| Marshall Islands | PMP | 5 | 3 | 2006 | 2011 | 0.811 | -0.10(-0.70,0.60) |
| Marshall Islands | PMP | 5 | 4 | 2011 | 2016 | 0.000 | -2.00(-2.60,-1.40) |
| Marshall Islands | PMP | 5 | 5 | 2016 | 2021 | 0.000 | -3.70(-4.10,-3.30) |
| Mauritania | APMP | 3 | 0 | 1990 | 1999 | 0.000 | 1.00(0.90,1.20) |
| Mauritania | APMP | 3 | 1 | 1999 | 2007 | 0.000 | -1.80(-2.00,-1.70) |
| Mauritania | APMP | 3 | 2 | 2007 | 2015 | 0.000 | 1.40(1.20,1.60) |
| Mauritania | APMP | 3 | 3 | 2015 | 2021 | 0.000 | -3.90(-4.10,-3.70) |
| Mauritania | HAP | 4 | 0 | 1990 | 1994 | 0.000 | -1.20(-1.80,-0.70) |
| Mauritania | HAP | 4 | 1 | 1994 | 1999 | 0.000 | -2.30(-2.90,-1.80) |
| Mauritania | HAP | 4 | 2 | 1999 | 2006 | 0.000 | -1.30(-1.60,-1.00) |
| Mauritania | HAP | 4 | 3 | 2006 | 2016 | 0.000 | -4.10(-4.20,-3.90) |
| Mauritania | HAP | 4 | 4 | 2016 | 2021 | 0.000 | -4.70(-5.00,-4.30) |
| Mauritania | PMP | 4 | 0 | 1990 | 1995 | 0.000 | -0.70(-1.00,-0.50) |
| Mauritania | PMP | 4 | 1 | 1995 | 2005 | 0.000 | -1.40(-1.50,-1.30) |
| Mauritania | PMP | 4 | 2 | 2005 | 2008 | 0.000 | -2.50(-3.60,-1.50) |
| Mauritania | PMP | 4 | 3 | 2008 | 2015 | 0.000 | -2.00(-2.20,-1.90) |
| Mauritania | PMP | 4 | 4 | 2015 | 2021 | 0.000 | -4.20(-4.40,-4.00) |
| Mauritius | APMP | 4 | 0 | 1990 | 1997 | 0.002 | 4.60(1.90,7.20) |
| Mauritius | APMP | 4 | 1 | 1997 | 2001 | 0.017 | -11.20(-19.30,-2.40) |
| Mauritius | APMP | 4 | 2 | 2001 | 2006 | 0.215 | 3.70(-2.30,10.20) |
| Mauritius | APMP | 4 | 3 | 2006 | 2010 | 0.015 | -11.40(-19.40,-2.60) |
| Mauritius | APMP | 4 | 4 | 2010 | 2021 | 0.023 | 1.50(0.20,2.80) |
| Mauritius | HAP | 4 | 0 | 1990 | 1997 | 0.000 | -11.10(-13.10,-9.00) |
| Mauritius | HAP | 4 | 1 | 1997 | 2001 | 0.000 | -23.30(-29.60,-16.40) |
| Mauritius | HAP | 4 | 2 | 2001 | 2007 | 0.001 | -7.30(-10.80,-3.70) |
| Mauritius | HAP | 4 | 3 | 2007 | 2010 | 0.033 | -17.20(-30.30,-1.70) |
| Mauritius | HAP | 4 | 4 | 2010 | 2021 | 0.000 | -10.60(-11.60,-9.60) |
| Mauritius | PMP | 4 | 0 | 1990 | 1997 | 0.619 | 0.60(-1.90,3.20) |
| Mauritius | PMP | 4 | 1 | 1997 | 2001 | 0.009 | -12.50(-20.50,-3.70) |
| Mauritius | PMP | 4 | 2 | 2001 | 2006 | 0.330 | 2.90(-3.10,9.40) |
| Mauritius | PMP | 4 | 3 | 2006 | 2010 | 0.015 | -11.60(-19.70,-2.70) |
| Mauritius | PMP | 4 | 4 | 2010 | 2021 | 0.078 | 1.20(-0.10,2.50) |
| Mexico | APMP | 4 | 0 | 1990 | 2000 | 0.000 | -2.30(-2.40,-2.20) |
| Mexico | APMP | 4 | 1 | 2000 | 2010 | 0.000 | -5.90(-6.10,-5.80) |
| Mexico | APMP | 4 | 2 | 2010 | 2015 | 0.000 | -1.90(-2.40,-1.50) |
| Mexico | APMP | 4 | 3 | 2015 | 2019 | 0.000 | -9.80(-10.50,-9.00) |
| Mexico | APMP | 4 | 4 | 2019 | 2021 | 0.004 | -2.50(-4.10,-0.90) |
| Mexico | HAP | 3 | 0 | 1990 | 2004 | 0.000 | -4.60(-4.70,-4.40) |
| Mexico | HAP | 3 | 1 | 2004 | 2016 | 0.000 | -7.80(-8.00,-7.70) |
| Mexico | HAP | 3 | 2 | 2016 | 2019 | 0.000 | -9.30(-12.00,-6.60) |
| Mexico | HAP | 3 | 3 | 2019 | 2021 | 0.095 | -2.50(-5.40,0.50) |
| Mexico | PMP | 4 | 0 | 1990 | 2001 | 0.000 | -3.00(-3.10,-2.90) |
| Mexico | PMP | 4 | 1 | 2001 | 2010 | 0.000 | -6.30(-6.50,-6.20) |
| Mexico | PMP | 4 | 2 | 2010 | 2015 | 0.000 | -3.10(-3.50,-2.60) |
| Mexico | PMP | 4 | 3 | 2015 | 2019 | 0.000 | -9.60(-10.30,-9.00) |
| Mexico | PMP | 4 | 4 | 2019 | 2021 | 0.001 | -2.50(-3.90,-1.20) |
| Micronesia (Federated States of) | APMP | 4 | 0 | 1990 | 1999 | 0.003 | 0.40(0.20,0.70) |
| Micronesia (Federated States of) | APMP | 4 | 1 | 1999 | 2007 | 0.000 | -3.50(-3.90,-3.10) |
| Micronesia (Federated States of) | APMP | 4 | 2 | 2007 | 2010 | 0.096 | -2.40(-5.20,0.50) |
| Micronesia (Federated States of) | APMP | 4 | 3 | 2010 | 2015 | 0.000 | 2.00(1.00,2.90) |
| Micronesia (Federated States of) | APMP | 4 | 4 | 2015 | 2021 | 0.000 | -2.40(-2.80,-1.90) |
| Micronesia (Federated States of) | HAP | 5 | 0 | 1990 | 1994 | 0.000 | -2.00(-2.70,-1.40) |
| Micronesia (Federated States of) | HAP | 5 | 1 | 1994 | 1999 | 0.000 | -4.10(-4.80,-3.50) |
| Micronesia (Federated States of) | HAP | 5 | 2 | 1999 | 2008 | 0.000 | -6.00(-6.20,-5.80) |
| Micronesia (Federated States of) | HAP | 5 | 3 | 2008 | 2016 | 0.000 | -3.30(-3.60,-3.00) |
| Micronesia (Federated States of) | HAP | 5 | 4 | 2016 | 2019 | 0.000 | -6.50(-8.40,-4.50) |
| Micronesia (Federated States of) | HAP | 5 | 5 | 2019 | 2021 | 0.031 | -2.30(-4.30,-0.20) |
| Micronesia (Federated States of) | PMP | 5 | 0 | 1990 | 1994 | 0.000 | -1.80(-2.50,-1.20) |
| Micronesia (Federated States of) | PMP | 5 | 1 | 1994 | 1999 | 0.000 | -3.70(-4.40,-3.10) |
| Micronesia (Federated States of) | PMP | 5 | 2 | 1999 | 2008 | 0.000 | -5.80(-6.00,-5.60) |
| Micronesia (Federated States of) | PMP | 5 | 3 | 2008 | 2016 | 0.000 | -2.80(-3.00,-2.50) |
| Micronesia (Federated States of) | PMP | 5 | 4 | 2016 | 2019 | 0.000 | -5.80(-7.80,-3.80) |
| Micronesia (Federated States of) | PMP | 5 | 5 | 2019 | 2021 | 0.033 | -2.30(-4.30,-0.20) |
| Monaco | APMP | 5 | 0 | 1990 | 1995 | 0.000 | -4.30(-5.00,-3.60) |
| Monaco | APMP | 5 | 1 | 1995 | 1999 | 0.000 | 9.10(7.30,11.00) |
| Monaco | APMP | 5 | 2 | 1999 | 2002 | 0.027 | -3.80(-7.10,-0.50) |
| Monaco | APMP | 5 | 3 | 2002 | 2016 | 0.000 | -5.80(-6.00,-5.60) |
| Monaco | APMP | 5 | 4 | 2016 | 2019 | 0.000 | -7.50(-10.60,-4.30) |
| Monaco | APMP | 5 | 5 | 2019 | 2021 | 0.877 | 0.30(-3.10,3.70) |
| Monaco | HAP | 3 | 0 | 1990 | 1992 | 0.472 | 47.30(-50.90,341.80) |
| Monaco | HAP | 3 | 1 | 1992 | 1995 | 0.018 | -74.10(-91.40,-22.30) |
| Monaco | HAP | 3 | 2 | 1995 | 1998 | 0.095 | 151.80(-16.10,655.50) |
| Monaco | HAP | 3 | 3 | 1998 | 2021 | 0.000 | -7.80(-10.00,-5.50) |
| Monaco | PMP | 5 | 0 | 1990 | 1995 | 0.000 | -4.30(-5.00,-3.60) |
| Monaco | PMP | 5 | 1 | 1995 | 1999 | 0.000 | 9.10(7.30,11.00) |
| Monaco | PMP | 5 | 2 | 1999 | 2002 | 0.027 | -3.80(-7.10,-0.50) |
| Monaco | PMP | 5 | 3 | 2002 | 2016 | 0.000 | -5.80(-6.00,-5.60) |
| Monaco | PMP | 5 | 4 | 2016 | 2019 | 0.000 | -7.50(-10.60,-4.30) |
| Monaco | PMP | 5 | 5 | 2019 | 2021 | 0.876 | 0.30(-3.10,3.70) |
| Mongolia | APMP | 3 | 0 | 1990 | 1999 | 0.018 | -0.80(-1.40,-0.10) |
| Mongolia | APMP | 3 | 1 | 1999 | 2005 | 0.013 | 2.10(0.50,3.70) |
| Mongolia | APMP | 3 | 2 | 2005 | 2011 | 0.000 | 11.70(10.00,13.50) |
| Mongolia | APMP | 3 | 3 | 2011 | 2021 | 0.118 | -0.40(-1.00,0.10) |
| Mongolia | HAP | 4 | 0 | 1990 | 1997 | 0.423 | -0.40(-1.30,0.60) |
| Mongolia | HAP | 4 | 1 | 1997 | 2004 | 0.000 | -3.00(-4.10,-1.80) |
| Mongolia | HAP | 4 | 2 | 2004 | 2010 | 0.000 | -8.70(-10.20,-7.30) |
| Mongolia | HAP | 4 | 3 | 2010 | 2016 | 0.000 | -18.30(-19.60,-17.00) |
| Mongolia | HAP | 4 | 4 | 2016 | 2021 | 0.000 | -5.80(-7.30,-4.30) |
| Mongolia | PMP | 3 | 0 | 1990 | 2000 | 0.000 | -0.90(-1.30,-0.40) |
| Mongolia | PMP | 3 | 1 | 2000 | 2010 | 0.000 | -4.10(-4.50,-3.60) |
| Mongolia | PMP | 3 | 2 | 2010 | 2016 | 0.000 | -8.70(-9.80,-7.60) |
| Mongolia | PMP | 3 | 3 | 2016 | 2021 | 0.002 | -2.10(-3.30,-0.90) |
| Montenegro | APMP | 4 | 0 | 1990 | 1999 | 0.000 | -4.60(-5.60,-3.70) |
| Montenegro | APMP | 4 | 1 | 1999 | 2002 | 0.939 | -0.40(-10.70,11.10) |
| Montenegro | APMP | 4 | 2 | 2002 | 2010 | 0.000 | -6.30(-7.70,-4.90) |
| Montenegro | APMP | 4 | 3 | 2010 | 2018 | 0.000 | -12.10(-13.40,-10.80) |
| Montenegro | APMP | 4 | 4 | 2018 | 2021 | 0.087 | -4.60(-9.70,0.80) |
| Montenegro | HAP | 3 | 0 | 1990 | 1994 | 0.000 | 12.00(8.70,15.50) |
| Montenegro | HAP | 3 | 1 | 1994 | 2002 | 0.000 | -2.70(-3.90,-1.40) |
| Montenegro | HAP | 3 | 2 | 2002 | 2019 | 0.000 | -16.40(-16.70,-16.10) |
| Montenegro | HAP | 3 | 3 | 2019 | 2021 | 0.325 | -4.60(-13.30,5.10) |
| Montenegro | PMP | 4 | 0 | 1990 | 1995 | 0.746 | 0.40(-2.10,2.90) |
| Montenegro | PMP | 4 | 1 | 1995 | 2002 | 0.001 | -3.50(-5.30,-1.60) |
| Montenegro | PMP | 4 | 2 | 2002 | 2010 | 0.000 | -9.40(-10.70,-8.10) |
| Montenegro | PMP | 4 | 3 | 2010 | 2018 | 0.000 | -13.20(-14.40,-11.90) |
| Montenegro | PMP | 4 | 4 | 2018 | 2021 | 0.060 | -5.20(-10.30,0.20) |
| Morocco | APMP | 5 | 0 | 1990 | 1995 | 0.000 | 1.60(1.10,2.10) |
| Morocco | APMP | 5 | 1 | 1995 | 1999 | 0.000 | 4.80(3.70,5.90) |
| Morocco | APMP | 5 | 2 | 1999 | 2002 | 0.633 | 0.50(-1.70,2.70) |
| Morocco | APMP | 5 | 3 | 2002 | 2009 | 0.000 | -1.70(-2.00,-1.30) |
| Morocco | APMP | 5 | 4 | 2009 | 2015 | 0.000 | -4.30(-4.70,-3.80) |
| Morocco | APMP | 5 | 5 | 2015 | 2021 | 0.000 | -8.60(-9.00,-8.30) |
| Morocco | HAP | 2 | 0 | 1990 | 2004 | 0.000 | -5.40(-5.90,-4.80) |
| Morocco | HAP | 2 | 1 | 2004 | 2008 | 0.000 | -12.00(-17.10,-6.60) |
| Morocco | HAP | 2 | 2 | 2008 | 2021 | 0.000 | -16.30(-16.90,-15.80) |
| Morocco | PMP | 4 | 0 | 1990 | 1995 | 0.000 | -2.10(-2.80,-1.50) |
| Morocco | PMP | 4 | 1 | 1995 | 2000 | 0.873 | 0.10(-0.80,1.00) |
| Morocco | PMP | 4 | 2 | 2000 | 2005 | 0.000 | -3.00(-3.80,-2.10) |
| Morocco | PMP | 4 | 3 | 2005 | 2014 | 0.000 | -5.60(-5.90,-5.30) |
| Morocco | PMP | 4 | 4 | 2014 | 2021 | 0.000 | -9.10(-9.40,-8.70) |
| Mozambique | APMP | 5 | 0 | 1990 | 1995 | 0.000 | -2.60(-3.10,-2.10) |
| Mozambique | APMP | 5 | 1 | 1995 | 1999 | 0.000 | 2.50(1.30,3.70) |
| Mozambique | APMP | 5 | 2 | 1999 | 2005 | 0.000 | -2.10(-2.60,-1.60) |
| Mozambique | APMP | 5 | 3 | 2005 | 2009 | 0.001 | 2.20(1.00,3.40) |
| Mozambique | APMP | 5 | 4 | 2009 | 2014 | 0.157 | 0.50(-0.20,1.20) |
| Mozambique | APMP | 5 | 5 | 2014 | 2021 | 0.000 | -3.10(-3.40,-2.80) |
| Mozambique | HAP | 5 | 0 | 1990 | 1995 | 0.000 | -2.00(-2.30,-1.70) |
| Mozambique | HAP | 5 | 1 | 1995 | 1998 | 0.513 | 0.40(-0.90,1.80) |
| Mozambique | HAP | 5 | 2 | 1998 | 2005 | 0.000 | -2.30(-2.50,-2.10) |
| Mozambique | HAP | 5 | 3 | 2005 | 2009 | 0.645 | -0.20(-0.80,0.50) |
| Mozambique | HAP | 5 | 4 | 2009 | 2018 | 0.000 | -1.90(-2.10,-1.80) |
| Mozambique | HAP | 5 | 5 | 2018 | 2021 | 0.000 | -3.20(-3.90,-2.60) |
| Mozambique | PMP | 5 | 0 | 1990 | 1995 | 0.000 | -1.90(-2.30,-1.60) |
| Mozambique | PMP | 5 | 1 | 1995 | 1999 | 0.937 | 0.00(-0.70,0.70) |
| Mozambique | PMP | 5 | 2 | 1999 | 2005 | 0.000 | -2.50(-2.80,-2.20) |
| Mozambique | PMP | 5 | 3 | 2005 | 2009 | 0.969 | 0.00(-0.70,0.70) |
| Mozambique | PMP | 5 | 4 | 2009 | 2016 | 0.000 | -1.70(-2.00,-1.50) |
| Mozambique | PMP | 5 | 5 | 2016 | 2021 | 0.000 | -2.80(-3.10,-2.50) |
| Myanmar | APMP | 5 | 0 | 1990 | 2000 | 0.000 | -0.20(-0.30,-0.20) |
| Myanmar | APMP | 5 | 1 | 2000 | 2005 | 0.000 | 3.20(2.90,3.50) |
| Myanmar | APMP | 5 | 2 | 2005 | 2011 | 0.013 | -0.30(-0.40,-0.10) |
| Myanmar | APMP | 5 | 3 | 2011 | 2015 | 0.000 | 2.70(2.20,3.10) |
| Myanmar | APMP | 5 | 4 | 2015 | 2019 | 0.000 | 7.10(6.60,7.50) |
| Myanmar | APMP | 5 | 5 | 2019 | 2021 | 0.000 | -2.80(-3.60,-1.90) |
| Myanmar | HAP | 3 | 0 | 1990 | 1996 | 0.355 | -0.30(-1.10,0.40) |
| Myanmar | HAP | 3 | 1 | 1996 | 2005 | 0.000 | -1.40(-1.90,-0.90) |
| Myanmar | HAP | 3 | 2 | 2005 | 2012 | 0.000 | -3.70(-4.40,-2.90) |
| Myanmar | HAP | 3 | 3 | 2012 | 2021 | 0.000 | -5.30(-5.70,-4.90) |
| Myanmar | PMP | 2 | 0 | 1990 | 1995 | 0.143 | -0.30(-0.70,0.10) |
| Myanmar | PMP | 2 | 1 | 1995 | 2005 | 0.000 | -1.00(-1.20,-0.80) |
| Myanmar | PMP | 2 | 2 | 2005 | 2021 | 0.000 | -3.30(-3.40,-3.20) |
| Namibia | APMP | 5 | 0 | 1990 | 1996 | 0.000 | -3.50(-3.70,-3.20) |
| Namibia | APMP | 5 | 1 | 1996 | 2002 | 0.012 | 0.50(0.10,0.80) |
| Namibia | APMP | 5 | 2 | 2002 | 2005 | 0.002 | 2.80(1.20,4.40) |
| Namibia | APMP | 5 | 3 | 2005 | 2010 | 0.000 | 6.30(5.80,6.80) |
| Namibia | APMP | 5 | 4 | 2010 | 2014 | 0.000 | 3.60(2.80,4.40) |
| Namibia | APMP | 5 | 5 | 2014 | 2021 | 0.000 | -2.50(-2.70,-2.30) |
| Namibia | HAP | 4 | 0 | 1990 | 2000 | 0.000 | -3.60(-3.80,-3.50) |
| Namibia | HAP | 4 | 1 | 2000 | 2008 | 0.000 | -2.30(-2.60,-2.00) |
| Namibia | HAP | 4 | 2 | 2008 | 2011 | 0.000 | -5.90(-7.90,-3.80) |
| Namibia | HAP | 4 | 3 | 2011 | 2018 | 0.000 | -9.00(-9.30,-8.70) |
| Namibia | HAP | 4 | 4 | 2018 | 2021 | 0.000 | -3.70(-4.80,-2.70) |
| Namibia | PMP | 5 | 0 | 1990 | 1998 | 0.000 | -3.50(-3.60,-3.40) |
| Namibia | PMP | 5 | 1 | 1998 | 2003 | 0.000 | -2.00(-2.30,-1.60) |
| Namibia | PMP | 5 | 2 | 2003 | 2009 | 0.063 | -0.20(-0.50,0.00) |
| Namibia | PMP | 5 | 3 | 2009 | 2013 | 0.000 | -3.10(-3.60,-2.50) |
| Namibia | PMP | 5 | 4 | 2013 | 2019 | 0.000 | -5.00(-5.20,-4.80) |
| Namibia | PMP | 5 | 5 | 2019 | 2021 | 0.000 | -2.50(-3.60,-1.40) |
| Nauru | APMP | 5 | 0 | 1990 | 1997 | 0.008 | 0.50(0.10,0.80) |
| Nauru | APMP | 5 | 1 | 1997 | 2001 | 0.000 | -3.50(-4.60,-2.30) |
| Nauru | APMP | 5 | 2 | 2001 | 2005 | 0.000 | -6.30(-7.50,-5.20) |
| Nauru | APMP | 5 | 3 | 2005 | 2010 | 0.000 | -3.40(-4.20,-2.70) |
| Nauru | APMP | 5 | 4 | 2010 | 2018 | 0.000 | 3.80(3.50,4.20) |
| Nauru | APMP | 5 | 5 | 2018 | 2021 | 0.043 | -1.20(-2.40,0.00) |
| Nauru | HAP | 4 | 0 | 1990 | 1999 | 0.000 | 19.60(19.10,20.20) |
| Nauru | HAP | 4 | 1 | 1999 | 2005 | 0.000 | 3.10(2.00,4.20) |
| Nauru | HAP | 4 | 2 | 2005 | 2011 | 0.000 | -7.20(-8.20,-6.20) |
| Nauru | HAP | 4 | 3 | 2011 | 2019 | 0.000 | -16.80(-17.40,-16.30) |
| Nauru | HAP | 4 | 4 | 2019 | 2021 | 0.449 | -1.80(-6.40,3.10) |
| Nauru | PMP | 5 | 0 | 1990 | 1992 | 0.043 | 3.50(0.10,7.00) |
| Nauru | PMP | 5 | 1 | 1992 | 1999 | 0.000 | 6.30(5.70,6.90) |
| Nauru | PMP | 5 | 2 | 1999 | 2002 | 0.433 | 1.30(-2.10,4.70) |
| Nauru | PMP | 5 | 3 | 2002 | 2006 | 0.006 | -2.50(-4.10,-0.90) |
| Nauru | PMP | 5 | 4 | 2006 | 2015 | 0.000 | -5.90(-6.20,-5.50) |
| Nauru | PMP | 5 | 5 | 2015 | 2021 | 0.000 | -2.60(-3.10,-2.10) |
| Nepal | APMP | 3 | 0 | 1990 | 2005 | 0.000 | -0.90(-1.20,-0.50) |
| Nepal | APMP | 3 | 1 | 2005 | 2010 | 0.000 | -5.20(-7.70,-2.60) |
| Nepal | APMP | 3 | 2 | 2010 | 2015 | 0.000 | 7.80(5.00,10.80) |
| Nepal | APMP | 3 | 3 | 2015 | 2021 | 0.000 | -5.20(-6.50,-3.80) |
| Nepal | HAP | 4 | 0 | 1990 | 2000 | 0.000 | -2.40(-2.50,-2.20) |
| Nepal | HAP | 4 | 1 | 2000 | 2007 | 0.000 | -1.70(-2.00,-1.40) |
| Nepal | HAP | 4 | 2 | 2007 | 2010 | 0.009 | -2.80(-4.70,-0.80) |
| Nepal | HAP | 4 | 3 | 2010 | 2015 | 0.000 | -5.90(-6.50,-5.30) |
| Nepal | HAP | 4 | 4 | 2015 | 2021 | 0.000 | -3.30(-3.70,-3.00) |
| Nepal | PMP | 5 | 0 | 1990 | 1992 | 0.000 | -2.70(-3.60,-1.80) |
| Nepal | PMP | 5 | 1 | 1992 | 1997 | 0.000 | -1.90(-2.20,-1.60) |
| Nepal | PMP | 5 | 2 | 1997 | 2000 | 0.000 | -2.40(-3.30,-1.50) |
| Nepal | PMP | 5 | 3 | 2000 | 2004 | 0.000 | -1.40(-1.90,-1.00) |
| Nepal | PMP | 5 | 4 | 2004 | 2008 | 0.000 | -2.20(-2.70,-1.80) |
| Nepal | PMP | 5 | 5 | 2008 | 2021 | 0.000 | -3.60(-3.60,-3.50) |
| Netherlands | APMP | 2 | 0 | 1990 | 2005 | 0.000 | -3.80(-4.20,-3.50) |
| Netherlands | APMP | 2 | 1 | 2005 | 2008 | 0.116 | -6.90(-15.00,1.90) |
| Netherlands | APMP | 2 | 2 | 2008 | 2021 | 0.000 | -3.50(-4.00,-3.00) |
| Netherlands | HAP | 4 | 0 | 1990 | 1999 | 0.000 | -15.60(-16.70,-14.40) |
| Netherlands | HAP | 4 | 1 | 1999 | 2006 | 0.000 | -10.30(-12.50,-8.00) |
| Netherlands | HAP | 4 | 2 | 2006 | 2009 | 0.004 | -20.70(-31.60,-8.10) |
| Netherlands | HAP | 4 | 3 | 2009 | 2017 | 0.000 | -8.90(-10.70,-7.10) |
| Netherlands | HAP | 4 | 4 | 2017 | 2021 | 0.768 | -0.70(-5.20,4.10) |
| Netherlands | PMP | 2 | 0 | 1990 | 2005 | 0.000 | -3.80(-4.20,-3.50) |
| Netherlands | PMP | 2 | 1 | 2005 | 2008 | 0.116 | -6.90(-15.00,1.90) |
| Netherlands | PMP | 2 | 2 | 2008 | 2021 | 0.000 | -3.50(-4.00,-3.00) |
| New Zealand | APMP | 2 | 0 | 1990 | 2006 | 0.000 | -2.80(-3.60,-2.00) |
| New Zealand | APMP | 2 | 1 | 2006 | 2010 | 0.243 | 6.30(-4.30,18.00) |
| New Zealand | APMP | 2 | 2 | 2010 | 2021 | 0.005 | -2.10(-3.50,-0.70) |
| New Zealand | HAP | 2 | 0 | 1990 | 1996 | 0.000 | -9.90(-13.10,-6.70) |
| New Zealand | HAP | 2 | 1 | 1996 | 2006 | 0.000 | -14.10(-15.80,-12.50) |
| New Zealand | HAP | 2 | 2 | 2006 | 2021 | 0.000 | -6.90(-7.70,-6.10) |
| New Zealand | PMP | 2 | 0 | 1990 | 2006 | 0.000 | -2.90(-3.70,-2.20) |
| New Zealand | PMP | 2 | 1 | 2006 | 2010 | 0.242 | 6.30(-4.30,17.90) |
| New Zealand | PMP | 2 | 2 | 2010 | 2021 | 0.005 | -2.10(-3.50,-0.70) |
| Nicaragua | APMP | 4 | 0 | 1990 | 1994 | 0.001 | -5.30(-7.80,-2.60) |
| Nicaragua | APMP | 4 | 1 | 1994 | 2004 | 0.097 | -0.70(-1.40,0.10) |
| Nicaragua | APMP | 4 | 2 | 2004 | 2011 | 0.000 | -3.10(-4.50,-1.70) |
| Nicaragua | APMP | 4 | 3 | 2011 | 2014 | 0.335 | 4.20(-4.50,13.70) |
| Nicaragua | APMP | 4 | 4 | 2014 | 2021 | 0.000 | -5.70(-6.80,-4.60) |
| Nicaragua | HAP | 4 | 0 | 1990 | 1996 | 0.000 | -2.10(-2.30,-1.90) |
| Nicaragua | HAP | 4 | 1 | 1996 | 2001 | 0.000 | -4.60(-5.00,-4.10) |
| Nicaragua | HAP | 4 | 2 | 2001 | 2006 | 0.000 | -3.80(-4.30,-3.40) |
| Nicaragua | HAP | 4 | 3 | 2006 | 2014 | 0.000 | -4.50(-4.60,-4.30) |
| Nicaragua | HAP | 4 | 4 | 2014 | 2021 | 0.000 | -6.20(-6.30,-6.00) |
| Nicaragua | PMP | 2 | 0 | 1990 | 1996 | 0.000 | -2.40(-2.80,-1.90) |
| Nicaragua | PMP | 2 | 1 | 1996 | 2015 | 0.000 | -3.80(-3.90,-3.70) |
| Nicaragua | PMP | 2 | 2 | 2015 | 2021 | 0.000 | -6.20(-6.70,-5.70) |
| Niger | APMP | 3 | 0 | 1990 | 2004 | 0.455 | -0.20(-0.70,0.30) |
| Niger | APMP | 3 | 1 | 2004 | 2010 | 0.000 | -5.20(-7.60,-2.80) |
| Niger | APMP | 3 | 2 | 2010 | 2014 | 0.060 | 5.50(-0.20,11.60) |
| Niger | APMP | 3 | 3 | 2014 | 2021 | 0.000 | -4.60(-6.00,-3.20) |
| Niger | HAP | 2 | 0 | 1990 | 1998 | 0.037 | 0.80(0.10,1.50) |
| Niger | HAP | 2 | 1 | 1998 | 2010 | 0.000 | -2.90(-3.30,-2.40) |
| Niger | HAP | 2 | 2 | 2010 | 2021 | 0.154 | -0.30(-0.80,0.10) |
| Niger | PMP | 4 | 0 | 1990 | 1998 | 0.035 | 0.60(0.00,1.20) |
| Niger | PMP | 4 | 1 | 1998 | 2006 | 0.000 | -2.40(-3.00,-1.70) |
| Niger | PMP | 4 | 2 | 2006 | 2009 | 0.050 | -5.10(-9.90,0.00) |
| Niger | PMP | 4 | 3 | 2009 | 2015 | 0.460 | 0.40(-0.70,1.60) |
| Niger | PMP | 4 | 4 | 2015 | 2021 | 0.006 | -1.30(-2.20,-0.40) |
| Nigeria | APMP | 5 | 0 | 1990 | 1994 | 0.001 | -3.60(-5.40,-1.80) |
| Nigeria | APMP | 5 | 1 | 1994 | 2007 | 0.016 | 0.40(0.10,0.80) |
| Nigeria | APMP | 5 | 2 | 2007 | 2010 | 0.126 | 4.60(-1.40,10.90) |
| Nigeria | APMP | 5 | 3 | 2010 | 2015 | 0.000 | 9.70(7.70,11.80) |
| Nigeria | APMP | 5 | 4 | 2015 | 2018 | 0.005 | -8.80(-14.00,-3.20) |
| Nigeria | APMP | 5 | 5 | 2018 | 2021 | 0.022 | -3.40(-6.20,-0.60) |
| Nigeria | HAP | 5 | 0 | 1990 | 1994 | 0.007 | 1.10(0.40,1.90) |
| Nigeria | HAP | 5 | 1 | 1994 | 2005 | 0.002 | -0.30(-0.50,-0.10) |
| Nigeria | HAP | 5 | 2 | 2005 | 2010 | 0.001 | -1.50(-2.30,-0.80) |
| Nigeria | HAP | 5 | 3 | 2010 | 2015 | 0.000 | -4.10(-4.80,-3.30) |
| Nigeria | HAP | 5 | 4 | 2015 | 2018 | 0.376 | 1.00(-1.40,3.50) |
| Nigeria | HAP | 5 | 5 | 2018 | 2021 | 0.002 | -2.10(-3.20,-0.90) |
| Nigeria | PMP | 3 | 0 | 1990 | 2002 | 0.006 | -0.10(-0.10,0.00) |
| Nigeria | PMP | 3 | 1 | 2002 | 2009 | 0.000 | -0.60(-0.70,-0.50) |
| Nigeria | PMP | 3 | 2 | 2009 | 2015 | 0.000 | 0.30(0.20,0.50) |
| Nigeria | PMP | 3 | 3 | 2015 | 2021 | 0.000 | -2.50(-2.60,-2.40) |
| Niue | APMP | 1 | 0 | 1990 | 2018 | 0.043 | -0.30(-0.60,0.00) |
| Niue | APMP | 1 | 1 | 2018 | 2021 | 0.000 | 30.40(19.20,42.80) |
| Niue | HAP | 3 | 0 | 1990 | 2000 | 0.000 | -4.30(-4.50,-4.10) |
| Niue | HAP | 3 | 1 | 2000 | 2010 | 0.000 | -15.70(-16.00,-15.50) |
| Niue | HAP | 3 | 2 | 2010 | 2019 | 0.000 | -3.70(-4.00,-3.40) |
| Niue | HAP | 3 | 3 | 2019 | 2021 | 0.000 | 40.60(36.70,44.60) |
| Niue | PMP | 3 | 0 | 1990 | 2000 | 0.000 | -2.80(-4.00,-1.50) |
| Niue | PMP | 3 | 1 | 2000 | 2008 | 0.000 | -9.20(-11.20,-7.20) |
| Niue | PMP | 3 | 2 | 2008 | 2018 | 0.266 | -0.80(-2.30,0.70) |
| Niue | PMP | 3 | 3 | 2018 | 2021 | 0.000 | 27.90(17.70,38.80) |
| North Macedonia | APMP | 5 | 0 | 1990 | 2000 | 0.000 | -5.60(-6.80,-4.40) |
| North Macedonia | APMP | 5 | 1 | 2000 | 2008 | 0.823 | 0.20(-1.90,2.40) |
| North Macedonia | APMP | 5 | 2 | 2008 | 2011 | 0.403 | -6.40(-20.40,10.20) |
| North Macedonia | APMP | 5 | 3 | 2011 | 2016 | 0.035 | 5.80(0.50,11.40) |
| North Macedonia | APMP | 5 | 4 | 2016 | 2019 | 0.002 | -24.90(-36.20,-11.60) |
| North Macedonia | APMP | 5 | 5 | 2019 | 2021 | 0.414 | -6.20(-20.30,10.40) |
| North Macedonia | HAP | 4 | 0 | 1990 | 2006 | 0.000 | -4.70(-5.40,-4.10) |
| North Macedonia | HAP | 4 | 1 | 2006 | 2011 | 0.000 | -14.20(-19.00,-9.00) |
| North Macedonia | HAP | 4 | 2 | 2011 | 2016 | 0.081 | -5.00(-10.40,0.70) |
| North Macedonia | HAP | 4 | 3 | 2016 | 2019 | 0.001 | -29.60(-41.40,-15.40) |
| North Macedonia | HAP | 4 | 4 | 2019 | 2021 | 0.411 | -7.10(-22.70,11.70) |
| North Macedonia | PMP | 3 | 0 | 1990 | 2011 | 0.000 | -4.20(-4.70,-3.70) |
| North Macedonia | PMP | 3 | 1 | 2011 | 2016 | 0.574 | 1.80(-4.50,8.40) |
| North Macedonia | PMP | 3 | 2 | 2016 | 2019 | 0.007 | -24.80(-38.50,-8.20) |
| North Macedonia | PMP | 3 | 3 | 2019 | 2021 | 0.489 | -6.60(-23.50,14.20) |
| Northern Mariana Islands | APMP | 3 | 0 | 1990 | 2002 | 0.000 | -4.00(-4.30,-3.80) |
| Northern Mariana Islands | APMP | 3 | 1 | 2002 | 2010 | 0.120 | -0.50(-1.10,0.10) |
| Northern Mariana Islands | APMP | 3 | 2 | 2010 | 2015 | 0.000 | 12.40(10.80,14.00) |
| Northern Mariana Islands | APMP | 3 | 3 | 2015 | 2021 | 0.000 | -6.20(-7.00,-5.50) |
| Northern Mariana Islands | HAP | 4 | 0 | 1990 | 2002 | 0.000 | -4.40(-5.00,-3.70) |
| Northern Mariana Islands | HAP | 4 | 1 | 2002 | 2007 | 0.013 | 5.20(1.20,9.30) |
| Northern Mariana Islands | HAP | 4 | 2 | 2007 | 2015 | 0.000 | 21.40(19.50,23.40) |
| Northern Mariana Islands | HAP | 4 | 3 | 2015 | 2019 | 0.000 | -23.80(-28.30,-19.00) |
| Northern Mariana Islands | HAP | 4 | 4 | 2019 | 2021 | 0.121 | -9.00(-19.50,2.80) |
| Northern Mariana Islands | PMP | 3 | 0 | 1990 | 2003 | 0.000 | -3.90(-4.10,-3.70) |
| Northern Mariana Islands | PMP | 3 | 1 | 2003 | 2010 | 0.548 | 0.20(-0.50,1.00) |
| Northern Mariana Islands | PMP | 3 | 2 | 2010 | 2015 | 0.000 | 12.60(11.10,14.10) |
| Northern Mariana Islands | PMP | 3 | 3 | 2015 | 2021 | 0.000 | -6.80(-7.40,-6.10) |
| Norway | APMP | 2 | 0 | 1990 | 1996 | 0.000 | -10.10(-11.80,-8.50) |
| Norway | APMP | 2 | 1 | 1996 | 2000 | 0.478 | -1.90(-7.00,3.60) |
| Norway | APMP | 2 | 2 | 2000 | 2021 | 0.000 | -5.30(-5.50,-5.00) |
| Norway | HAP | 1 | 0 | 1990 | 2001 | 0.000 | -16.60(-18.00,-15.20) |
| Norway | HAP | 1 | 1 | 2001 | 2021 | 0.000 | -10.00(-10.70,-9.40) |
| Norway | PMP | 2 | 0 | 1990 | 1996 | 0.000 | -10.10(-11.80,-8.50) |
| Norway | PMP | 2 | 1 | 1996 | 2000 | 0.476 | -1.90(-7.00,3.60) |
| Norway | PMP | 2 | 2 | 2000 | 2021 | 0.000 | -5.30(-5.50,-5.00) |
| Oman | APMP | 3 | 0 | 1990 | 2000 | 0.000 | -6.70(-7.00,-6.30) |
| Oman | APMP | 3 | 1 | 2000 | 2005 | 0.000 | -9.10(-10.50,-7.60) |
| Oman | APMP | 3 | 2 | 2005 | 2017 | 0.000 | 3.20(2.80,3.50) |
| Oman | APMP | 3 | 3 | 2017 | 2021 | 0.000 | -11.30(-12.70,-9.90) |
| Oman | HAP | 3 | 0 | 1990 | 1996 | 0.000 | -17.80(-19.20,-16.30) |
| Oman | HAP | 3 | 1 | 1996 | 2005 | 0.000 | -24.80(-25.70,-23.90) |
| Oman | HAP | 3 | 2 | 2005 | 2009 | 0.000 | -15.70(-20.00,-11.20) |
| Oman | HAP | 3 | 3 | 2009 | 2021 | 0.000 | -9.00(-9.60,-8.50) |
| Oman | PMP | 4 | 0 | 1990 | 1995 | 0.000 | -7.70(-8.60,-6.80) |
| Oman | PMP | 4 | 1 | 1995 | 1999 | 0.000 | -5.70(-7.60,-3.60) |
| Oman | PMP | 4 | 2 | 1999 | 2005 | 0.000 | -9.10(-10.00,-8.30) |
| Oman | PMP | 4 | 3 | 2005 | 2017 | 0.000 | 3.20(2.90,3.50) |
| Oman | PMP | 4 | 4 | 2017 | 2021 | 0.000 | -11.30(-12.50,-10.10) |
| Pakistan | APMP | 3 | 0 | 1990 | 2003 | 0.000 | 1.30(0.90,1.70) |
| Pakistan | APMP | 3 | 1 | 2003 | 2010 | 0.000 | -2.40(-3.60,-1.20) |
| Pakistan | APMP | 3 | 2 | 2010 | 2015 | 0.000 | 9.70(7.20,12.20) |
| Pakistan | APMP | 3 | 3 | 2015 | 2021 | 0.000 | -2.80(-4.00,-1.70) |
| Pakistan | HAP | 4 | 0 | 1990 | 1995 | 0.001 | 0.70(0.30,1.10) |
| Pakistan | HAP | 4 | 1 | 1995 | 2003 | 0.000 | -0.90(-1.20,-0.70) |
| Pakistan | HAP | 4 | 2 | 2003 | 2010 | 0.000 | -2.40(-2.70,-2.20) |
| Pakistan | HAP | 4 | 3 | 2010 | 2019 | 0.000 | -3.30(-3.50,-3.10) |
| Pakistan | HAP | 4 | 4 | 2019 | 2021 | 0.001 | 3.10(1.40,4.80) |
| Pakistan | PMP | 5 | 0 | 1990 | 1995 | 0.000 | 1.00(0.70,1.30) |
| Pakistan | PMP | 5 | 1 | 1995 | 2004 | 0.000 | -0.60(-0.80,-0.50) |
| Pakistan | PMP | 5 | 2 | 2004 | 2009 | 0.000 | -2.90(-3.30,-2.50) |
| Pakistan | PMP | 5 | 3 | 2009 | 2016 | 0.313 | -0.10(-0.30,0.10) |
| Pakistan | PMP | 5 | 4 | 2016 | 2019 | 0.000 | -4.00(-5.30,-2.80) |
| Pakistan | PMP | 5 | 5 | 2019 | 2021 | 0.002 | 2.40(1.00,3.70) |
| Palau | APMP | 4 | 0 | 1990 | 1999 | 0.000 | -5.40(-6.10,-4.70) |
| Palau | APMP | 4 | 1 | 1999 | 2005 | 0.324 | -0.90(-2.70,1.00) |
| Palau | APMP | 4 | 2 | 2005 | 2010 | 0.004 | -4.00(-6.50,-1.50) |
| Palau | APMP | 4 | 3 | 2010 | 2015 | 0.000 | 12.00(9.10,15.00) |
| Palau | APMP | 4 | 4 | 2015 | 2021 | 0.000 | -6.40(-7.70,-5.00) |
| Palau | HAP | 5 | 0 | 1990 | 1998 | 0.000 | -12.20(-12.50,-11.80) |
| Palau | HAP | 5 | 1 | 1998 | 2004 | 0.000 | -3.60(-4.50,-2.80) |
| Palau | HAP | 5 | 2 | 2004 | 2013 | 0.024 | -0.50(-1.00,-0.10) |
| Palau | HAP | 5 | 3 | 2013 | 2016 | 0.021 | -4.80(-8.50,-0.90) |
| Palau | HAP | 5 | 4 | 2016 | 2019 | 0.000 | -12.80(-16.20,-9.20) |
| Palau | HAP | 5 | 5 | 2019 | 2021 | 0.495 | -1.30(-5.20,2.70) |
| Palau | PMP | 4 | 0 | 1990 | 1999 | 0.000 | -5.50(-6.20,-4.70) |
| Palau | PMP | 4 | 1 | 1999 | 2005 | 0.322 | -0.90(-2.70,1.00) |
| Palau | PMP | 4 | 2 | 2005 | 2010 | 0.004 | -4.00(-6.50,-1.50) |
| Palau | PMP | 4 | 3 | 2010 | 2015 | 0.000 | 12.00(9.10,14.90) |
| Palau | PMP | 4 | 4 | 2015 | 2021 | 0.000 | -6.40(-7.70,-5.00) |
| Palestine | APMP | 3 | 0 | 1990 | 1993 | 0.039 | 4.60(0.30,9.20) |
| Palestine | APMP | 3 | 1 | 1993 | 1998 | 0.316 | -1.30(-4.00,1.40) |
| Palestine | APMP | 3 | 2 | 1998 | 2009 | 0.173 | 0.50(-0.20,1.10) |
| Palestine | APMP | 3 | 3 | 2009 | 2021 | 0.000 | -6.30(-6.80,-5.80) |
| Palestine | HAP | 3 | 0 | 1990 | 1999 | 0.000 | -9.40(-10.00,-8.70) |
| Palestine | HAP | 3 | 1 | 1999 | 2005 | 0.000 | -5.00(-6.70,-3.40) |
| Palestine | HAP | 3 | 2 | 2005 | 2015 | 0.000 | -8.70(-9.40,-8.10) |
| Palestine | HAP | 3 | 3 | 2015 | 2021 | 0.000 | -13.10(-14.30,-12.00) |
| Palestine | PMP | 2 | 0 | 1990 | 1999 | 0.000 | -2.70(-3.40,-1.90) |
| Palestine | PMP | 2 | 1 | 1999 | 2009 | 0.049 | -0.70(-1.50,0.00) |
| Palestine | PMP | 2 | 2 | 2009 | 2021 | 0.000 | -6.70(-7.10,-6.30) |
| Panama | APMP | 3 | 0 | 1990 | 2000 | 0.496 | 0.20(-0.50,0.90) |
| Panama | APMP | 3 | 1 | 2000 | 2008 | 0.000 | -4.80(-6.00,-3.70) |
| Panama | APMP | 3 | 2 | 2008 | 2017 | 0.001 | -1.80(-2.80,-0.80) |
| Panama | APMP | 3 | 3 | 2017 | 2021 | 0.000 | -8.30(-10.90,-5.60) |
| Panama | HAP | 2 | 0 | 1990 | 1998 | 0.000 | -5.80(-7.50,-4.10) |
| Panama | HAP | 2 | 1 | 1998 | 2006 | 0.000 | -11.20(-13.20,-9.20) |
| Panama | HAP | 2 | 2 | 2006 | 2021 | 0.000 | -17.40(-17.90,-16.80) |
| Panama | PMP | 3 | 0 | 1990 | 2000 | 0.000 | -2.90(-3.50,-2.30) |
| Panama | PMP | 3 | 1 | 2000 | 2009 | 0.000 | -7.30(-8.10,-6.50) |
| Panama | PMP | 3 | 2 | 2009 | 2017 | 0.000 | -3.80(-4.80,-2.80) |
| Panama | PMP | 3 | 3 | 2017 | 2021 | 0.000 | -8.30(-10.50,-6.00) |
| Papua New Guinea | APMP | 5 | 0 | 1990 | 1996 | 0.000 | -0.40(-0.60,-0.30) |
| Papua New Guinea | APMP | 5 | 1 | 1996 | 2000 | 0.000 | 1.50(1.20,1.90) |
| Papua New Guinea | APMP | 5 | 2 | 2000 | 2006 | 0.000 | -3.90(-4.10,-3.70) |
| Papua New Guinea | APMP | 5 | 3 | 2006 | 2009 | 0.000 | 1.70(0.90,2.50) |
| Papua New Guinea | APMP | 5 | 4 | 2009 | 2019 | 0.000 | 3.70(3.60,3.80) |
| Papua New Guinea | APMP | 5 | 5 | 2019 | 2021 | 0.011 | 1.10(0.30,1.90) |
| Papua New Guinea | HAP | 5 | 0 | 1990 | 1996 | 0.000 | -1.80(-1.90,-1.70) |
| Papua New Guinea | HAP | 5 | 1 | 1996 | 2004 | 0.000 | -0.40(-0.50,-0.30) |
| Papua New Guinea | HAP | 5 | 2 | 2004 | 2007 | 0.000 | -2.60(-3.30,-1.90) |
| Papua New Guinea | HAP | 5 | 3 | 2007 | 2010 | 0.901 | 0.00(-0.60,0.70) |
| Papua New Guinea | HAP | 5 | 4 | 2010 | 2015 | 0.000 | 1.50(1.30,1.80) |
| Papua New Guinea | HAP | 5 | 5 | 2015 | 2021 | 0.000 | 0.60(0.50,0.70) |
| Papua New Guinea | PMP | 4 | 0 | 1990 | 1996 | 0.000 | -1.70(-1.80,-1.60) |
| Papua New Guinea | PMP | 4 | 1 | 1996 | 2003 | 0.000 | -0.40(-0.50,-0.20) |
| Papua New Guinea | PMP | 4 | 2 | 2003 | 2008 | 0.000 | -2.00(-2.30,-1.80) |
| Papua New Guinea | PMP | 4 | 3 | 2008 | 2018 | 0.000 | 1.40(1.30,1.50) |
| Papua New Guinea | PMP | 4 | 4 | 2018 | 2021 | 0.064 | 0.40(0.00,0.80) |
| Paraguay | APMP | 1 | 0 | 1990 | 1999 | 0.004 | 1.30(0.50,2.20) |
| Paraguay | APMP | 1 | 1 | 1999 | 2021 | 0.000 | -2.50(-2.70,-2.30) |
| Paraguay | HAP | 1 | 0 | 1990 | 2004 | 0.000 | -1.60(-2.20,-1.00) |
| Paraguay | HAP | 1 | 1 | 2004 | 2021 | 0.000 | -10.50(-10.90,-10.10) |
| Paraguay | PMP | 1 | 0 | 1990 | 2003 | 0.000 | -1.20(-1.70,-0.60) |
| Paraguay | PMP | 1 | 1 | 2003 | 2021 | 0.000 | -7.60(-7.90,-7.20) |
| Peru | APMP | 3 | 0 | 1990 | 2005 | 0.000 | -2.40(-2.80,-2.10) |
| Peru | APMP | 3 | 1 | 2005 | 2009 | 0.000 | -9.00(-12.60,-5.20) |
| Peru | APMP | 3 | 2 | 2009 | 2018 | 0.091 | -0.70(-1.60,0.10) |
| Peru | APMP | 3 | 3 | 2018 | 2021 | 0.000 | -14.00(-17.40,-10.50) |
| Peru | HAP | 2 | 0 | 1990 | 2006 | 0.000 | -5.90(-6.20,-5.50) |
| Peru | HAP | 2 | 1 | 2006 | 2011 | 0.000 | -7.50(-10.20,-4.60) |
| Peru | HAP | 2 | 2 | 2011 | 2021 | 0.000 | -15.70(-16.30,-15.10) |
| Peru | PMP | 4 | 0 | 1990 | 1999 | 0.000 | -4.60(-5.30,-4.00) |
| Peru | PMP | 4 | 1 | 1999 | 2005 | 0.001 | -2.90(-4.40,-1.30) |
| Peru | PMP | 4 | 2 | 2005 | 2009 | 0.000 | -8.90(-12.10,-5.50) |
| Peru | PMP | 4 | 3 | 2009 | 2018 | 0.000 | -4.40(-5.10,-3.60) |
| Peru | PMP | 4 | 4 | 2018 | 2021 | 0.000 | -14.20(-17.30,-11.10) |
| Philippines | APMP | 4 | 0 | 1990 | 1998 | 0.644 | -0.10(-0.50,0.30) |
| Philippines | APMP | 4 | 1 | 1998 | 2004 | 0.000 | 2.60(1.80,3.50) |
| Philippines | APMP | 4 | 2 | 2004 | 2012 | 0.000 | -3.50(-3.90,-3.00) |
| Philippines | APMP | 4 | 3 | 2012 | 2019 | 0.000 | 2.00(1.40,2.60) |
| Philippines | APMP | 4 | 4 | 2019 | 2021 | 0.000 | -7.90(-11.30,-4.50) |
| Philippines | HAP | 3 | 0 | 1990 | 1998 | 0.000 | -3.10(-3.60,-2.50) |
| Philippines | HAP | 3 | 1 | 1998 | 2004 | 0.699 | 0.20(-0.90,1.40) |
| Philippines | HAP | 3 | 2 | 2004 | 2014 | 0.000 | -2.00(-2.50,-1.60) |
| Philippines | HAP | 3 | 3 | 2014 | 2021 | 0.000 | -5.80(-6.50,-5.10) |
| Philippines | PMP | 3 | 0 | 1990 | 1998 | 0.000 | -2.40(-2.90,-1.90) |
| Philippines | PMP | 3 | 1 | 1998 | 2004 | 0.162 | 0.80(-0.30,1.90) |
| Philippines | PMP | 3 | 2 | 2004 | 2016 | 0.000 | -2.30(-2.60,-1.90) |
| Philippines | PMP | 3 | 3 | 2016 | 2021 | 0.000 | -4.50(-5.50,-3.40) |
| Poland | APMP | 3 | 0 | 1990 | 1994 | 0.598 | 0.60(-1.80,3.10) |
| Poland | APMP | 3 | 1 | 1994 | 2000 | 0.000 | -11.90(-13.40,-10.30) |
| Poland | APMP | 3 | 2 | 2000 | 2008 | 0.009 | -1.40(-2.40,-0.40) |
| Poland | APMP | 3 | 3 | 2008 | 2021 | 0.000 | -7.40(-7.70,-7.00) |
| Poland | HAP | 4 | 0 | 1990 | 1995 | 0.000 | -5.10(-7.50,-2.60) |
| Poland | HAP | 4 | 1 | 1995 | 2000 | 0.000 | -21.20(-24.10,-18.30) |
| Poland | HAP | 4 | 2 | 2000 | 2007 | 0.000 | -11.80(-13.50,-10.10) |
| Poland | HAP | 4 | 3 | 2007 | 2019 | 0.000 | -16.50(-17.20,-15.90) |
| Poland | HAP | 4 | 4 | 2019 | 2021 | 0.210 | -6.90(-17.10,4.50) |
| Poland | PMP | 3 | 0 | 1990 | 1995 | 0.038 | -2.80(-5.30,-0.20) |
| Poland | PMP | 3 | 1 | 1995 | 2000 | 0.000 | -15.10(-18.10,-11.80) |
| Poland | PMP | 3 | 2 | 2000 | 2008 | 0.001 | -2.90(-4.40,-1.40) |
| Poland | PMP | 3 | 3 | 2008 | 2021 | 0.000 | -7.90(-8.40,-7.30) |
| Portugal | APMP | 4 | 0 | 1990 | 1994 | 0.000 | -11.90(-15.80,-7.80) |
| Portugal | APMP | 4 | 1 | 1994 | 2005 | 0.000 | -4.90(-6.00,-3.80) |
| Portugal | APMP | 4 | 2 | 2005 | 2008 | 0.132 | -10.30(-22.30,3.70) |
| Portugal | APMP | 4 | 3 | 2008 | 2018 | 0.000 | -3.90(-5.20,-2.70) |
| Portugal | APMP | 4 | 4 | 2018 | 2021 | 0.008 | -9.80(-16.10,-3.10) |
| Portugal | HAP | 3 | 0 | 1990 | 1995 | 0.000 | -25.40(-27.90,-22.80) |
| Portugal | HAP | 3 | 1 | 1995 | 2008 | 0.000 | -17.20(-17.90,-16.40) |
| Portugal | HAP | 3 | 2 | 2008 | 2015 | 0.000 | -7.50(-9.90,-5.00) |
| Portugal | HAP | 3 | 3 | 2015 | 2021 | 0.000 | -12.30(-14.50,-9.90) |
| Portugal | PMP | 4 | 0 | 1990 | 1994 | 0.000 | -12.80(-16.60,-8.70) |
| Portugal | PMP | 4 | 1 | 1994 | 2005 | 0.000 | -5.10(-6.20,-4.10) |
| Portugal | PMP | 4 | 2 | 2005 | 2008 | 0.133 | -10.20(-22.30,3.70) |
| Portugal | PMP | 4 | 3 | 2008 | 2018 | 0.000 | -4.00(-5.20,-2.70) |
| Portugal | PMP | 4 | 4 | 2018 | 2021 | 0.008 | -9.80(-16.10,-3.10) |
| Puerto Rico | APMP | 2 | 0 | 1990 | 2004 | 0.000 | -5.20(-5.60,-4.70) |
| Puerto Rico | APMP | 2 | 1 | 2004 | 2012 | 0.105 | -1.00(-2.20,0.20) |
| Puerto Rico | APMP | 2 | 2 | 2012 | 2021 | 0.000 | -4.00(-4.80,-3.20) |
| Puerto Rico | HAP | 3 | 0 | 1990 | 2000 | 0.000 | -18.10(-19.00,-17.20) |
| Puerto Rico | HAP | 3 | 1 | 2000 | 2005 | 0.000 | -11.80(-15.80,-7.70) |
| Puerto Rico | HAP | 3 | 2 | 2005 | 2012 | 0.167 | -1.70(-4.00,0.80) |
| Puerto Rico | HAP | 3 | 3 | 2012 | 2021 | 0.000 | -8.10(-9.30,-6.90) |
| Puerto Rico | PMP | 2 | 0 | 1990 | 2004 | 0.000 | -5.20(-5.60,-4.80) |
| Puerto Rico | PMP | 2 | 1 | 2004 | 2012 | 0.108 | -1.00(-2.20,0.20) |
| Puerto Rico | PMP | 2 | 2 | 2012 | 2021 | 0.000 | -4.00(-4.80,-3.20) |
| Qatar | APMP | 4 | 0 | 1990 | 1997 | 0.000 | -4.20(-4.50,-3.90) |
| Qatar | APMP | 4 | 1 | 1997 | 2005 | 0.000 | -5.20(-5.40,-4.90) |
| Qatar | APMP | 4 | 2 | 2005 | 2010 | 0.000 | -6.80(-7.50,-6.10) |
| Qatar | APMP | 4 | 3 | 2010 | 2019 | 0.000 | -3.30(-3.50,-3.00) |
| Qatar | APMP | 4 | 4 | 2019 | 2021 | 0.000 | -11.10(-13.10,-9.10) |
| Qatar | HAP | 2 | 0 | 1990 | 2003 | 0.000 | -12.40(-13.60,-11.20) |
| Qatar | HAP | 2 | 1 | 2003 | 2015 | 0.000 | -17.90(-19.40,-16.40) |
| Qatar | HAP | 2 | 2 | 2015 | 2021 | 0.071 | -4.00(-8.20,0.40) |
| Qatar | PMP | 4 | 0 | 1990 | 1997 | 0.000 | -4.20(-4.50,-3.90) |
| Qatar | PMP | 4 | 1 | 1997 | 2005 | 0.000 | -5.20(-5.40,-4.90) |
| Qatar | PMP | 4 | 2 | 2005 | 2010 | 0.000 | -6.80(-7.50,-6.10) |
| Qatar | PMP | 4 | 3 | 2010 | 2019 | 0.000 | -3.30(-3.50,-3.00) |
| Qatar | PMP | 4 | 4 | 2019 | 2021 | 0.000 | -11.10(-13.10,-9.10) |
| Republic of Korea | APMP | 5 | 0 | 1990 | 1999 | 0.000 | -6.10(-6.70,-5.50) |
| Republic of Korea | APMP | 5 | 1 | 1999 | 2003 | 0.296 | 1.80(-1.70,5.40) |
| Republic of Korea | APMP | 5 | 2 | 2003 | 2009 | 0.000 | -10.60(-12.00,-9.20) |
| Republic of Korea | APMP | 5 | 3 | 2009 | 2016 | 0.005 | 1.80(0.60,3.00) |
| Republic of Korea | APMP | 5 | 4 | 2016 | 2019 | 0.509 | -2.20(-8.80,4.90) |
| Republic of Korea | APMP | 5 | 5 | 2019 | 2021 | 0.000 | -16.60(-22.30,-10.60) |
| Republic of Korea | HAP | 5 | 0 | 1990 | 1999 | 0.000 | -28.60(-29.10,-28.10) |
| Republic of Korea | HAP | 5 | 1 | 1999 | 2002 | 0.001 | -14.50(-21.10,-7.30) |
| Republic of Korea | HAP | 5 | 2 | 2002 | 2008 | 0.000 | -25.10(-26.50,-23.80) |
| Republic of Korea | HAP | 5 | 3 | 2008 | 2014 | 0.000 | -15.70(-17.20,-14.20) |
| Republic of Korea | HAP | 5 | 4 | 2014 | 2019 | 0.001 | -4.60(-7.00,-2.10) |
| Republic of Korea | HAP | 5 | 5 | 2019 | 2021 | 0.000 | -18.60(-24.90,-11.80) |
| Republic of Korea | PMP | 5 | 0 | 1990 | 1999 | 0.000 | -6.50(-7.10,-6.00) |
| Republic of Korea | PMP | 5 | 1 | 1999 | 2003 | 0.208 | 1.90(-1.10,4.90) |
| Republic of Korea | PMP | 5 | 2 | 2003 | 2009 | 0.000 | -10.70(-11.90,-9.50) |
| Republic of Korea | PMP | 5 | 3 | 2009 | 2016 | 0.002 | 1.80(0.80,2.80) |
| Republic of Korea | PMP | 5 | 4 | 2016 | 2019 | 0.439 | -2.20(-7.90,3.80) |
| Republic of Korea | PMP | 5 | 5 | 2019 | 2021 | 0.000 | -16.60(-21.50,-11.50) |
| Republic of Moldova | APMP | 5 | 0 | 1990 | 1995 | 0.000 | -4.60(-6.20,-2.90) |
| Republic of Moldova | APMP | 5 | 1 | 1995 | 2002 | 0.000 | -10.00(-11.10,-8.80) |
| Republic of Moldova | APMP | 5 | 2 | 2002 | 2006 | 0.773 | -0.50(-4.20,3.30) |
| Republic of Moldova | APMP | 5 | 3 | 2006 | 2009 | 0.000 | 20.30(11.50,29.70) |
| Republic of Moldova | APMP | 5 | 4 | 2009 | 2013 | 0.313 | 1.90(-1.90,5.80) |
| Republic of Moldova | APMP | 5 | 5 | 2013 | 2021 | 0.000 | -6.30(-7.10,-5.60) |
| Republic of Moldova | HAP | 5 | 0 | 1990 | 1995 | 0.000 | 3.90(2.40,5.50) |
| Republic of Moldova | HAP | 5 | 1 | 1995 | 2000 | 0.000 | -4.80(-6.80,-2.80) |
| Republic of Moldova | HAP | 5 | 2 | 2000 | 2006 | 0.000 | -14.10(-15.40,-12.80) |
| Republic of Moldova | HAP | 5 | 3 | 2006 | 2009 | 0.617 | 1.60(-5.00,8.70) |
| Republic of Moldova | HAP | 5 | 4 | 2009 | 2019 | 0.000 | -11.30(-11.90,-10.80) |
| Republic of Moldova | HAP | 5 | 5 | 2019 | 2021 | 0.102 | -5.30(-11.50,1.20) |
| Republic of Moldova | PMP | 5 | 0 | 1990 | 1995 | 0.013 | 2.10(0.50,3.80) |
| Republic of Moldova | PMP | 5 | 1 | 1995 | 2000 | 0.000 | -5.40(-7.50,-3.20) |
| Republic of Moldova | PMP | 5 | 2 | 2000 | 2003 | 0.000 | -14.80(-20.60,-8.50) |
| Republic of Moldova | PMP | 5 | 3 | 2003 | 2006 | 0.012 | -9.20(-15.40,-2.50) |
| Republic of Moldova | PMP | 5 | 4 | 2006 | 2010 | 0.043 | 3.80(0.10,7.50) |
| Republic of Moldova | PMP | 5 | 5 | 2010 | 2021 | 0.000 | -8.00(-8.50,-7.60) |
| Romania | APMP | 5 | 0 | 1990 | 1997 | 0.749 | 0.20(-1.00,1.40) |
| Romania | APMP | 5 | 1 | 1997 | 2002 | 0.080 | -2.40(-5.10,0.30) |
| Romania | APMP | 5 | 2 | 2002 | 2005 | 0.136 | 6.80(-2.30,16.70) |
| Romania | APMP | 5 | 3 | 2005 | 2008 | 0.001 | -14.90(-22.10,-7.00) |
| Romania | APMP | 5 | 4 | 2008 | 2014 | 0.000 | -4.90(-6.70,-3.00) |
| Romania | APMP | 5 | 5 | 2014 | 2021 | 0.000 | -7.80(-8.90,-6.70) |
| Romania | HAP | 3 | 0 | 1990 | 1997 | 0.041 | 2.40(0.10,4.80) |
| Romania | HAP | 3 | 1 | 1997 | 2005 | 0.000 | -8.50(-10.60,-6.40) |
| Romania | HAP | 3 | 2 | 2005 | 2010 | 0.000 | -28.30(-32.10,-24.30) |
| Romania | HAP | 3 | 3 | 2010 | 2021 | 0.000 | -14.60(-15.60,-13.60) |
| Romania | PMP | 3 | 0 | 1990 | 1994 | 0.199 | 2.70(-1.50,7.00) |
| Romania | PMP | 3 | 1 | 1994 | 2005 | 0.000 | -2.30(-3.30,-1.30) |
| Romania | PMP | 3 | 2 | 2005 | 2008 | 0.010 | -16.10(-26.40,-4.50) |
| Romania | PMP | 3 | 3 | 2008 | 2021 | 0.000 | -7.40(-8.10,-6.80) |
| Russian Federation | APMP | 3 | 0 | 1990 | 1998 | 0.001 | -2.90(-4.40,-1.40) |
| Russian Federation | APMP | 3 | 1 | 1998 | 2009 | 0.000 | -9.50(-10.40,-8.50) |
| Russian Federation | APMP | 3 | 2 | 2009 | 2012 | 0.530 | 4.40(-9.20,19.90) |
| Russian Federation | APMP | 3 | 3 | 2012 | 2021 | 0.000 | -11.10(-12.20,-9.90) |
| Russian Federation | HAP | 5 | 0 | 1990 | 2000 | 0.000 | 6.50(5.70,7.30) |
| Russian Federation | HAP | 5 | 1 | 2000 | 2006 | 0.000 | -16.50(-18.20,-14.70) |
| Russian Federation | HAP | 5 | 2 | 2006 | 2010 | 0.000 | -28.10(-31.40,-24.70) |
| Russian Federation | HAP | 5 | 3 | 2010 | 2013 | 0.008 | -12.50(-20.30,-4.00) |
| Russian Federation | HAP | 5 | 4 | 2013 | 2017 | 0.000 | -21.30(-24.90,-17.60) |
| Russian Federation | HAP | 5 | 5 | 2017 | 2021 | 0.000 | -7.80(-10.50,-5.00) |
| Russian Federation | PMP | 3 | 0 | 1990 | 1999 | 0.001 | -2.40(-3.60,-1.20) |
| Russian Federation | PMP | 3 | 1 | 1999 | 2009 | 0.000 | -10.50(-11.60,-9.40) |
| Russian Federation | PMP | 3 | 2 | 2009 | 2012 | 0.643 | 3.20(-10.10,18.40) |
| Russian Federation | PMP | 3 | 3 | 2012 | 2021 | 0.000 | -11.10(-12.30,-10.00) |
| Rwanda | APMP | 3 | 0 | 1990 | 1995 | 0.002 | -2.20(-3.40,-0.90) |
| Rwanda | APMP | 3 | 1 | 1995 | 2000 | 0.000 | -6.40(-8.10,-4.70) |
| Rwanda | APMP | 3 | 2 | 2000 | 2013 | 0.000 | -1.00(-1.30,-0.60) |
| Rwanda | APMP | 3 | 3 | 2013 | 2021 | 0.000 | -5.10(-5.60,-4.50) |
| Rwanda | HAP | 2 | 0 | 1990 | 1995 | 0.271 | -0.80(-2.30,0.70) |
| Rwanda | HAP | 2 | 1 | 1995 | 1998 | 0.057 | -6.20(-12.20,0.20) |
| Rwanda | HAP | 2 | 2 | 1998 | 2021 | 0.000 | -2.40(-2.60,-2.30) |
| Rwanda | PMP | 3 | 0 | 1990 | 1995 | 0.269 | -0.70(-2.00,0.60) |
| Rwanda | PMP | 3 | 1 | 1995 | 1998 | 0.013 | -7.50(-12.80,-1.80) |
| Rwanda | PMP | 3 | 2 | 1998 | 2005 | 0.003 | -1.60(-2.60,-0.60) |
| Rwanda | PMP | 3 | 3 | 2005 | 2021 | 0.000 | -2.70(-2.90,-2.50) |
| Saint Kitts and Nevis | APMP | 4 | 0 | 1990 | 1998 | 0.000 | -3.20(-3.70,-2.70) |
| Saint Kitts and Nevis | APMP | 4 | 1 | 1998 | 2004 | 0.000 | -5.80(-6.80,-4.80) |
| Saint Kitts and Nevis | APMP | 4 | 2 | 2004 | 2011 | 0.000 | -2.00(-2.80,-1.20) |
| Saint Kitts and Nevis | APMP | 4 | 3 | 2011 | 2016 | 0.001 | 2.80(1.30,4.40) |
| Saint Kitts and Nevis | APMP | 4 | 4 | 2016 | 2021 | 0.000 | -2.80(-3.90,-1.80) |
| Saint Kitts and Nevis | HAP | 2 | 0 | 1990 | 2000 | 0.000 | -16.70(-17.70,-15.80) |
| Saint Kitts and Nevis | HAP | 2 | 1 | 2000 | 2011 | 0.000 | -11.50(-12.40,-10.40) |
| Saint Kitts and Nevis | HAP | 2 | 2 | 2011 | 2021 | 0.000 | -7.80(-8.80,-6.70) |
| Saint Kitts and Nevis | PMP | 3 | 0 | 1990 | 2005 | 0.000 | -5.50(-5.70,-5.30) |
| Saint Kitts and Nevis | PMP | 3 | 1 | 2005 | 2011 | 0.001 | -2.10(-3.20,-1.00) |
| Saint Kitts and Nevis | PMP | 3 | 2 | 2011 | 2016 | 0.003 | 2.50(0.90,4.20) |
| Saint Kitts and Nevis | PMP | 3 | 3 | 2016 | 2021 | 0.000 | -2.90(-4.00,-1.80) |
| Saint Lucia | APMP | 3 | 0 | 1990 | 2003 | 0.000 | 1.40(1.10,1.60) |
| Saint Lucia | APMP | 3 | 1 | 2003 | 2008 | 0.556 | -0.40(-1.70,0.90) |
| Saint Lucia | APMP | 3 | 2 | 2008 | 2015 | 0.000 | 2.20(1.50,2.90) |
| Saint Lucia | APMP | 3 | 3 | 2015 | 2021 | 0.000 | -2.70(-3.30,-2.00) |
| Saint Lucia | HAP | 4 | 0 | 1990 | 1998 | 0.000 | -9.80(-10.30,-9.40) |
| Saint Lucia | HAP | 4 | 1 | 1998 | 2005 | 0.000 | -5.20(-5.90,-4.50) |
| Saint Lucia | HAP | 4 | 2 | 2005 | 2010 | 0.000 | -11.50(-12.70,-10.30) |
| Saint Lucia | HAP | 4 | 3 | 2010 | 2019 | 0.000 | -9.30(-9.70,-8.80) |
| Saint Lucia | HAP | 4 | 4 | 2019 | 2021 | 0.100 | -3.50(-7.60,0.80) |
| Saint Lucia | PMP | 4 | 0 | 1990 | 1997 | 0.000 | -3.10(-3.70,-2.50) |
| Saint Lucia | PMP | 4 | 1 | 1997 | 2005 | 0.441 | -0.20(-0.80,0.40) |
| Saint Lucia | PMP | 4 | 2 | 2005 | 2008 | 0.148 | -3.20(-7.40,1.30) |
| Saint Lucia | PMP | 4 | 3 | 2008 | 2015 | 0.005 | 1.20(0.40,1.90) |
| Saint Lucia | PMP | 4 | 4 | 2015 | 2021 | 0.000 | -2.90(-3.60,-2.20) |
| Saint Vincent and the Grenadines | APMP | 2 | 0 | 1990 | 1999 | 0.000 | 3.20(2.90,3.50) |
| Saint Vincent and the Grenadines | APMP | 2 | 1 | 1999 | 2014 | 0.000 | -1.30(-1.40,-1.10) |
| Saint Vincent and the Grenadines | APMP | 2 | 2 | 2014 | 2021 | 0.000 | -4.40(-4.80,-4.00) |
| Saint Vincent and the Grenadines | HAP | 5 | 0 | 1990 | 1994 | 0.000 | -7.60(-8.50,-6.80) |
| Saint Vincent and the Grenadines | HAP | 5 | 1 | 1994 | 2000 | 0.000 | -9.70(-10.20,-9.00) |
| Saint Vincent and the Grenadines | HAP | 5 | 2 | 2000 | 2010 | 0.000 | -12.20(-12.50,-12.00) |
| Saint Vincent and the Grenadines | HAP | 5 | 3 | 2010 | 2016 | 0.000 | -9.20(-9.80,-8.60) |
| Saint Vincent and the Grenadines | HAP | 5 | 4 | 2016 | 2019 | 0.000 | -11.90(-14.50,-9.30) |
| Saint Vincent and the Grenadines | HAP | 5 | 5 | 2019 | 2021 | 0.010 | -4.00(-6.90,-1.20) |
| Saint Vincent and the Grenadines | PMP | 3 | 0 | 1990 | 2001 | 0.000 | -1.80(-2.00,-1.60) |
| Saint Vincent and the Grenadines | PMP | 3 | 1 | 2001 | 2004 | 0.001 | -4.70(-7.20,-2.10) |
| Saint Vincent and the Grenadines | PMP | 3 | 2 | 2004 | 2015 | 0.000 | -2.40(-2.70,-2.20) |
| Saint Vincent and the Grenadines | PMP | 3 | 3 | 2015 | 2021 | 0.000 | -5.00(-5.40,-4.50) |
| Samoa | APMP | 5 | 0 | 1990 | 1995 | 0.000 | -3.40(-3.80,-3.10) |
| Samoa | APMP | 5 | 1 | 1995 | 2004 | 0.000 | 1.10(0.90,1.30) |
| Samoa | APMP | 5 | 2 | 2004 | 2007 | 0.119 | -1.30(-3.00,0.40) |
| Samoa | APMP | 5 | 3 | 2007 | 2015 | 0.112 | 0.20(0.00,0.40) |
| Samoa | APMP | 5 | 4 | 2015 | 2019 | 0.002 | 1.60(0.70,2.40) |
| Samoa | APMP | 5 | 5 | 2019 | 2021 | 0.001 | -3.30(-4.90,-1.60) |
| Samoa | HAP | 5 | 0 | 1990 | 2004 | 0.000 | -3.00(-3.10,-3.00) |
| Samoa | HAP | 5 | 1 | 2004 | 2007 | 0.000 | -4.10(-5.30,-2.90) |
| Samoa | HAP | 5 | 2 | 2007 | 2012 | 0.000 | -1.30(-1.70,-1.00) |
| Samoa | HAP | 5 | 3 | 2012 | 2016 | 0.000 | -2.60(-3.20,-2.00) |
| Samoa | HAP | 5 | 4 | 2016 | 2019 | 0.000 | -4.80(-5.90,-3.60) |
| Samoa | HAP | 5 | 5 | 2019 | 2021 | 0.000 | -3.20(-4.40,-2.10) |
| Samoa | PMP | 5 | 0 | 1990 | 1994 | 0.000 | -3.30(-3.70,-2.90) |
| Samoa | PMP | 5 | 1 | 1994 | 2004 | 0.000 | -2.60(-2.80,-2.50) |
| Samoa | PMP | 5 | 2 | 2004 | 2007 | 0.000 | -3.80(-5.00,-2.60) |
| Samoa | PMP | 5 | 3 | 2007 | 2012 | 0.000 | -1.10(-1.50,-0.70) |
| Samoa | PMP | 5 | 4 | 2012 | 2017 | 0.000 | -2.40(-2.80,-2.00) |
| Samoa | PMP | 5 | 5 | 2017 | 2021 | 0.000 | -3.80(-4.20,-3.40) |
| San Marino | APMP | 3 | 0 | 1990 | 1997 | 0.000 | -6.40(-6.80,-6.00) |
| San Marino | APMP | 3 | 1 | 1997 | 2010 | 0.000 | -8.60(-8.70,-8.40) |
| San Marino | APMP | 3 | 2 | 2010 | 2019 | 0.000 | -0.70(-1.10,-0.40) |
| San Marino | APMP | 3 | 3 | 2019 | 2021 | 0.000 | -21.60(-24.20,-19.00) |
| San Marino | HAP | 5 | 0 | 1990 | 1995 | 0.000 | -18.50(-23.10,-13.60) |
| San Marino | HAP | 5 | 1 | 1995 | 2001 | 0.001 | -10.50(-15.60,-5.10) |
| San Marino | HAP | 5 | 2 | 2001 | 2005 | 0.000 | -35.60(-43.60,-26.60) |
| San Marino | HAP | 5 | 3 | 2005 | 2008 | 0.005 | 50.50(15.70,95.80) |
| San Marino | HAP | 5 | 4 | 2008 | 2015 | 0.245 | -2.50(-6.70,1.90) |
| San Marino | HAP | 5 | 5 | 2015 | 2021 | 0.000 | -20.40(-23.80,-16.80) |
| San Marino | PMP | 3 | 0 | 1990 | 1997 | 0.000 | -6.40(-6.80,-6.00) |
| San Marino | PMP | 3 | 1 | 1997 | 2010 | 0.000 | -8.60(-8.70,-8.40) |
| San Marino | PMP | 3 | 2 | 2010 | 2019 | 0.000 | -0.70(-1.10,-0.40) |
| San Marino | PMP | 3 | 3 | 2019 | 2021 | 0.000 | -21.60(-24.20,-19.00) |
| Sao Tome and Principe | APMP | 2 | 0 | 1990 | 2010 | 0.019 | 0.60(0.10,1.10) |
| Sao Tome and Principe | APMP | 2 | 1 | 2010 | 2014 | 0.223 | 5.80(-3.60,16.10) |
| Sao Tome and Principe | APMP | 2 | 2 | 2014 | 2021 | 0.000 | -5.40(-7.80,-3.00) |
| Sao Tome and Principe | HAP | 4 | 0 | 1990 | 1998 | 0.002 | 0.40(0.20,0.60) |
| Sao Tome and Principe | HAP | 4 | 1 | 1998 | 2009 | 0.000 | -3.00(-3.20,-2.90) |
| Sao Tome and Principe | HAP | 4 | 2 | 2009 | 2014 | 0.000 | -6.20(-6.90,-5.60) |
| Sao Tome and Principe | HAP | 4 | 3 | 2014 | 2017 | 0.000 | -13.10(-14.90,-11.30) |
| Sao Tome and Principe | HAP | 4 | 4 | 2017 | 2021 | 0.000 | -6.00(-6.70,-5.40) |
| Sao Tome and Principe | PMP | 4 | 0 | 1990 | 1998 | 0.209 | 0.30(-0.20,0.90) |
| Sao Tome and Principe | PMP | 4 | 1 | 1998 | 2008 | 0.000 | -2.60(-3.10,-2.20) |
| Sao Tome and Principe | PMP | 4 | 2 | 2008 | 2014 | 0.000 | -4.40(-5.50,-3.40) |
| Sao Tome and Principe | PMP | 4 | 3 | 2014 | 2017 | 0.000 | -12.10(-16.30,-7.70) |
| Sao Tome and Principe | PMP | 4 | 4 | 2017 | 2021 | 0.000 | -5.40(-6.80,-3.90) |
| Saudi Arabia | APMP | 3 | 0 | 1990 | 2004 | 0.000 | -4.00(-4.30,-3.70) |
| Saudi Arabia | APMP | 3 | 1 | 2004 | 2013 | 0.000 | -9.40(-10.00,-8.80) |
| Saudi Arabia | APMP | 3 | 2 | 2013 | 2018 | 0.000 | -7.40(-9.10,-5.60) |
| Saudi Arabia | APMP | 3 | 3 | 2018 | 2021 | 0.000 | -11.90(-14.50,-9.20) |
| Saudi Arabia | HAP | 1 | 0 | 1990 | 2001 | 0.000 | -13.70(-14.70,-12.70) |
| Saudi Arabia | HAP | 1 | 1 | 2001 | 2021 | 0.000 | -22.70(-23.10,-22.30) |
| Saudi Arabia | PMP | 3 | 0 | 1990 | 2004 | 0.000 | -4.00(-4.30,-3.80) |
| Saudi Arabia | PMP | 3 | 1 | 2004 | 2013 | 0.000 | -9.40(-10.00,-8.80) |
| Saudi Arabia | PMP | 3 | 2 | 2013 | 2018 | 0.000 | -7.40(-9.10,-5.60) |
| Saudi Arabia | PMP | 3 | 3 | 2018 | 2021 | 0.000 | -11.90(-14.50,-9.20) |
| Senegal | APMP | 4 | 0 | 1990 | 1997 | 0.049 | -1.10(-2.20,0.00) |
| Senegal | APMP | 4 | 1 | 1997 | 2005 | 0.000 | -9.30(-10.30,-8.30) |
| Senegal | APMP | 4 | 2 | 2005 | 2010 | 0.010 | -3.50(-6.00,-1.00) |
| Senegal | APMP | 4 | 3 | 2010 | 2015 | 0.001 | 5.40(2.70,8.20) |
| Senegal | APMP | 4 | 4 | 2015 | 2021 | 0.002 | -2.40(-3.80,-1.00) |
| Senegal | HAP | 2 | 0 | 1990 | 1997 | 0.000 | 1.30(1.10,1.50) |
| Senegal | HAP | 2 | 1 | 1997 | 2016 | 0.000 | -1.00(-1.10,-1.00) |
| Senegal | HAP | 2 | 2 | 2016 | 2021 | 0.000 | -5.40(-5.70,-5.10) |
| Senegal | PMP | 4 | 0 | 1990 | 1997 | 0.000 | 0.60(0.40,0.80) |
| Senegal | PMP | 4 | 1 | 1997 | 2005 | 0.000 | -2.30(-2.50,-2.10) |
| Senegal | PMP | 4 | 2 | 2005 | 2011 | 0.000 | -1.20(-1.60,-0.90) |
| Senegal | PMP | 4 | 3 | 2011 | 2016 | 0.233 | -0.30(-0.80,0.20) |
| Senegal | PMP | 4 | 4 | 2016 | 2021 | 0.000 | -5.10(-5.40,-4.80) |
| Serbia | APMP | 3 | 0 | 1990 | 2000 | 0.000 | -8.30(-9.50,-7.10) |
| Serbia | APMP | 3 | 1 | 2000 | 2004 | 0.067 | 8.00(-0.60,17.30) |
| Serbia | APMP | 3 | 2 | 2004 | 2008 | 0.000 | -15.60(-22.30,-8.30) |
| Serbia | APMP | 3 | 3 | 2008 | 2021 | 0.000 | -4.50(-5.30,-3.70) |
| Serbia | HAP | 3 | 0 | 1990 | 1995 | 0.052 | 4.00(0.00,8.20) |
| Serbia | HAP | 3 | 1 | 1995 | 2005 | 0.000 | -6.10(-7.60,-4.50) |
| Serbia | HAP | 3 | 2 | 2005 | 2008 | 0.000 | -35.30(-45.80,-22.80) |
| Serbia | HAP | 3 | 3 | 2008 | 2021 | 0.000 | -10.70(-11.50,-9.80) |
| Serbia | PMP | 2 | 0 | 1990 | 2005 | 0.000 | -4.10(-5.00,-3.20) |
| Serbia | PMP | 2 | 1 | 2005 | 2008 | 0.033 | -21.90(-37.70,-2.10) |
| Serbia | PMP | 2 | 2 | 2008 | 2021 | 0.000 | -5.70(-6.80,-4.50) |
| Seychelles | APMP | 1 | 0 | 1990 | 2010 | 0.000 | -1.40(-1.90,-1.00) |
| Seychelles | APMP | 1 | 1 | 2010 | 2021 | 0.001 | 2.00(0.90,3.10) |
| Seychelles | HAP | 2 | 0 | 1990 | 1999 | 0.000 | -18.00(-19.90,-16.20) |
| Seychelles | HAP | 2 | 1 | 1999 | 2004 | 0.168 | -5.20(-12.40,2.50) |
| Seychelles | HAP | 2 | 2 | 2004 | 2021 | 0.000 | -10.80(-11.60,-10.00) |
| Seychelles | PMP | 1 | 0 | 1990 | 2010 | 0.000 | -1.90(-2.40,-1.40) |
| Seychelles | PMP | 1 | 1 | 2010 | 2021 | 0.001 | 2.10(0.90,3.20) |
| Sierra Leone | APMP | 3 | 0 | 1990 | 2010 | 0.000 | -2.50(-2.70,-2.30) |
| Sierra Leone | APMP | 3 | 1 | 2010 | 2015 | 0.000 | 6.60(4.10,9.20) |
| Sierra Leone | APMP | 3 | 2 | 2015 | 2018 | 0.024 | -8.50(-15.30,-1.30) |
| Sierra Leone | APMP | 3 | 3 | 2018 | 2021 | 0.069 | -3.50(-7.10,0.30) |
| Sierra Leone | HAP | 5 | 0 | 1990 | 1993 | 0.220 | 0.60(-0.40,1.60) |
| Sierra Leone | HAP | 5 | 1 | 1993 | 2002 | 0.000 | -2.20(-2.40,-2.00) |
| Sierra Leone | HAP | 5 | 2 | 2002 | 2008 | 0.000 | -0.90(-1.40,-0.50) |
| Sierra Leone | HAP | 5 | 3 | 2008 | 2015 | 0.000 | -2.60(-3.00,-2.30) |
| Sierra Leone | HAP | 5 | 4 | 2015 | 2018 | 0.369 | -0.80(-2.80,1.10) |
| Sierra Leone | HAP | 5 | 5 | 2018 | 2021 | 0.000 | -2.80(-3.80,-1.90) |
| Sierra Leone | PMP | 4 | 0 | 1990 | 1993 | 0.821 | 0.10(-0.60,0.80) |
| Sierra Leone | PMP | 4 | 1 | 1993 | 2002 | 0.000 | -2.30(-2.40,-2.10) |
| Sierra Leone | PMP | 4 | 2 | 2002 | 2006 | 0.013 | -0.90(-1.70,-0.20) |
| Sierra Leone | PMP | 4 | 3 | 2006 | 2018 | 0.000 | -1.80(-1.90,-1.70) |
| Sierra Leone | PMP | 4 | 4 | 2018 | 2021 | 0.000 | -2.80(-3.50,-2.10) |
| Singapore | APMP | 2 | 0 | 1990 | 1992 | 0.008 | -28.20(-43.30,-9.10) |
| Singapore | APMP | 2 | 1 | 1992 | 2004 | 0.000 | -8.10(-9.50,-6.60) |
| Singapore | APMP | 2 | 2 | 2004 | 2021 | 0.000 | -2.60(-3.40,-1.80) |
| Singapore | HAP | 3 | 0 | 1990 | 2004 | 0.000 | -25.40(-26.60,-24.30) |
| Singapore | HAP | 3 | 1 | 2004 | 2011 | 0.001 | -10.30(-15.10,-5.20) |
| Singapore | HAP | 3 | 2 | 2011 | 2015 | 0.000 | -30.50(-41.00,-18.20) |
| Singapore | HAP | 3 | 3 | 2015 | 2021 | 0.456 | -2.00(-7.30,3.60) |
| Singapore | PMP | 2 | 0 | 1990 | 1992 | 0.007 | -28.50(-43.50,-9.50) |
| Singapore | PMP | 2 | 1 | 1992 | 2004 | 0.000 | -8.20(-9.60,-6.70) |
| Singapore | PMP | 2 | 2 | 2004 | 2021 | 0.000 | -2.60(-3.40,-1.80) |
| Slovakia | APMP | 5 | 0 | 1990 | 1995 | 0.005 | -4.10(-6.60,-1.50) |
| Slovakia | APMP | 5 | 1 | 1995 | 2001 | 0.000 | -9.70(-12.00,-7.20) |
| Slovakia | APMP | 5 | 2 | 2001 | 2004 | 0.818 | 1.30(-10.00,14.00) |
| Slovakia | APMP | 5 | 3 | 2004 | 2008 | 0.007 | -8.20(-13.50,-2.70) |
| Slovakia | APMP | 5 | 4 | 2008 | 2014 | 0.014 | -3.40(-5.90,-0.80) |
| Slovakia | APMP | 5 | 5 | 2014 | 2021 | 0.000 | -6.80(-8.30,-5.40) |
| Slovakia | HAP | 5 | 0 | 1990 | 1995 | 0.265 | -1.50(-4.10,1.30) |
| Slovakia | HAP | 5 | 1 | 1995 | 2004 | 0.000 | -13.10(-14.30,-12.00) |
| Slovakia | HAP | 5 | 2 | 2004 | 2010 | 0.000 | -19.40(-21.60,-17.20) |
| Slovakia | HAP | 5 | 3 | 2010 | 2014 | 0.001 | -11.70(-16.90,-6.10) |
| Slovakia | HAP | 5 | 4 | 2014 | 2018 | 0.000 | -16.70(-21.60,-11.50) |
| Slovakia | HAP | 5 | 5 | 2018 | 2021 | 0.051 | -5.90(-11.50,0.00) |
| Slovakia | PMP | 5 | 0 | 1990 | 1995 | 0.005 | -4.00(-6.50,-1.40) |
| Slovakia | PMP | 5 | 1 | 1995 | 2001 | 0.000 | -9.70(-12.10,-7.30) |
| Slovakia | PMP | 5 | 2 | 2001 | 2004 | 0.849 | 1.10(-10.20,13.80) |
| Slovakia | PMP | 5 | 3 | 2004 | 2008 | 0.007 | -8.30(-13.60,-2.70) |
| Slovakia | PMP | 5 | 4 | 2008 | 2014 | 0.013 | -3.50(-6.00,-0.90) |
| Slovakia | PMP | 5 | 5 | 2014 | 2021 | 0.000 | -6.90(-8.30,-5.40) |
| Slovenia | APMP | 2 | 0 | 1990 | 2001 | 0.000 | -8.10(-8.80,-7.30) |
| Slovenia | APMP | 2 | 1 | 2001 | 2005 | 0.694 | 1.20(-4.80,7.60) |
| Slovenia | APMP | 2 | 2 | 2005 | 2021 | 0.000 | -8.40(-8.80,-7.90) |
| Slovenia | HAP | 2 | 0 | 1990 | 1994 | 0.132 | -3.80(-8.70,1.30) |
| Slovenia | HAP | 2 | 1 | 1994 | 2008 | 0.000 | -13.50(-14.20,-12.80) |
| Slovenia | HAP | 2 | 2 | 2008 | 2021 | 0.000 | -11.70(-12.50,-11.00) |
| Slovenia | PMP | 4 | 0 | 1990 | 2001 | 0.000 | -8.30(-8.90,-7.70) |
| Slovenia | PMP | 4 | 1 | 2001 | 2005 | 0.583 | 1.40(-3.70,6.70) |
| Slovenia | PMP | 4 | 2 | 2005 | 2008 | 0.019 | -11.80(-20.40,-2.30) |
| Slovenia | PMP | 4 | 3 | 2008 | 2011 | 0.359 | -4.50(-13.80,5.80) |
| Slovenia | PMP | 4 | 4 | 2011 | 2021 | 0.000 | -9.10(-9.80,-8.40) |
| Solomon Islands | APMP | 4 | 0 | 1990 | 2000 | 0.000 | -1.20(-1.30,-1.10) |
| Solomon Islands | APMP | 4 | 1 | 2000 | 2006 | 0.000 | -3.80(-4.10,-3.50) |
| Solomon Islands | APMP | 4 | 2 | 2006 | 2015 | 0.000 | 0.70(0.60,0.90) |
| Solomon Islands | APMP | 4 | 3 | 2015 | 2018 | 0.014 | 1.80(0.40,3.30) |
| Solomon Islands | APMP | 4 | 4 | 2018 | 2021 | 0.000 | -1.70(-2.40,-1.00) |
| Solomon Islands | HAP | 5 | 0 | 1990 | 1993 | 0.000 | -1.30(-1.60,-1.10) |
| Solomon Islands | HAP | 5 | 1 | 1993 | 2000 | 0.000 | -2.80(-2.90,-2.70) |
| Solomon Islands | HAP | 5 | 2 | 2000 | 2003 | 0.000 | -1.50(-2.10,-1.00) |
| Solomon Islands | HAP | 5 | 3 | 2003 | 2008 | 0.000 | -2.40(-2.50,-2.20) |
| Solomon Islands | HAP | 5 | 4 | 2008 | 2015 | 0.000 | -0.80(-0.90,-0.70) |
| Solomon Islands | HAP | 5 | 5 | 2015 | 2021 | 0.000 | -1.80(-1.90,-1.70) |
| Solomon Islands | PMP | 5 | 0 | 1990 | 1993 | 0.000 | -1.30(-1.60,-1.10) |
| Solomon Islands | PMP | 5 | 1 | 1993 | 2000 | 0.000 | -2.70(-2.80,-2.70) |
| Solomon Islands | PMP | 5 | 2 | 2000 | 2004 | 0.000 | -1.70(-2.00,-1.40) |
| Solomon Islands | PMP | 5 | 3 | 2004 | 2007 | 0.000 | -2.80(-3.40,-2.30) |
| Solomon Islands | PMP | 5 | 4 | 2007 | 2016 | 0.000 | -0.80(-0.90,-0.80) |
| Solomon Islands | PMP | 5 | 5 | 2016 | 2021 | 0.000 | -1.90(-2.00,-1.70) |
| Somalia | APMP | 5 | 0 | 1990 | 1992 | 0.000 | -11.60(-15.50,-7.40) |
| Somalia | APMP | 5 | 1 | 1992 | 1995 | 0.017 | -5.60(-9.90,-1.20) |
| Somalia | APMP | 5 | 2 | 1995 | 2000 | 0.000 | 12.80(11.20,14.50) |
| Somalia | APMP | 5 | 3 | 2000 | 2005 | 0.000 | -5.70(-7.10,-4.30) |
| Somalia | APMP | 5 | 4 | 2005 | 2012 | 0.000 | 2.70(1.90,3.50) |
| Somalia | APMP | 5 | 5 | 2012 | 2021 | 0.000 | -1.70(-2.10,-1.30) |
| Somalia | HAP | 3 | 0 | 1990 | 1992 | 0.504 | 0.60(-1.20,2.40) |
| Somalia | HAP | 3 | 1 | 1992 | 1995 | 0.101 | -1.50(-3.30,0.30) |
| Somalia | HAP | 3 | 2 | 1995 | 2012 | 0.334 | 0.00(-0.10,0.00) |
| Somalia | HAP | 3 | 3 | 2012 | 2021 | 0.000 | -1.60(-1.70,-1.40) |
| Somalia | PMP | 5 | 0 | 1990 | 1992 | 0.992 | 0.00(-1.20,1.20) |
| Somalia | PMP | 5 | 1 | 1992 | 1995 | 0.016 | -1.50(-2.70,-0.30) |
| Somalia | PMP | 5 | 2 | 1995 | 2003 | 0.003 | 0.30(0.10,0.40) |
| Somalia | PMP | 5 | 3 | 2003 | 2008 | 0.035 | -0.40(-0.80,0.00) |
| Somalia | PMP | 5 | 4 | 2008 | 2011 | 0.200 | 0.80(-0.40,2.00) |
| Somalia | PMP | 5 | 5 | 2011 | 2021 | 0.000 | -1.50(-1.60,-1.40) |
| South Africa | APMP | 3 | 0 | 1990 | 2004 | 0.000 | -0.80(-1.00,-0.60) |
| South Africa | APMP | 3 | 1 | 2004 | 2009 | 0.061 | 1.60(-0.10,3.20) |
| South Africa | APMP | 3 | 2 | 2009 | 2019 | 0.000 | -1.00(-1.50,-0.60) |
| South Africa | APMP | 3 | 3 | 2019 | 2021 | 0.001 | 10.00(4.50,15.80) |
| South Africa | HAP | 3 | 0 | 1990 | 2004 | 0.000 | -3.70(-4.00,-3.40) |
| South Africa | HAP | 3 | 1 | 2004 | 2013 | 0.000 | -9.30(-9.90,-8.60) |
| South Africa | HAP | 3 | 2 | 2013 | 2018 | 0.000 | -5.80(-7.70,-3.90) |
| South Africa | HAP | 3 | 3 | 2018 | 2021 | 0.000 | 7.40(4.00,10.90) |
| South Africa | PMP | 1 | 0 | 1990 | 2019 | 0.000 | -2.60(-2.70,-2.50) |
| South Africa | PMP | 1 | 1 | 2019 | 2021 | 0.000 | 11.50(6.10,17.20) |
| South Sudan | APMP | 5 | 0 | 1990 | 1995 | 0.000 | -4.50(-5.40,-3.60) |
| South Sudan | APMP | 5 | 1 | 1995 | 1999 | 0.001 | 4.50(2.30,6.70) |
| South Sudan | APMP | 5 | 2 | 1999 | 2011 | 0.000 | 0.80(0.50,1.10) |
| South Sudan | APMP | 5 | 3 | 2011 | 2016 | 0.000 | -4.80(-6.10,-3.50) |
| South Sudan | APMP | 5 | 4 | 2016 | 2019 | 0.000 | -12.10(-15.70,-8.20) |
| South Sudan | APMP | 5 | 5 | 2019 | 2021 | 0.246 | -2.40(-6.50,1.90) |
| South Sudan | HAP | 5 | 0 | 1990 | 1993 | 0.000 | 1.30(0.70,1.90) |
| South Sudan | HAP | 5 | 1 | 1993 | 1999 | 0.000 | -0.60(-0.80,-0.30) |
| South Sudan | HAP | 5 | 2 | 1999 | 2008 | 0.000 | 0.60(0.50,0.70) |
| South Sudan | HAP | 5 | 3 | 2008 | 2011 | 0.108 | -0.90(-2.10,0.20) |
| South Sudan | HAP | 5 | 4 | 2011 | 2017 | 0.000 | 4.00(3.70,4.30) |
| South Sudan | HAP | 5 | 5 | 2017 | 2021 | 0.073 | -0.30(-0.70,0.00) |
| South Sudan | PMP | 4 | 0 | 1990 | 1997 | 0.002 | -0.30(-0.50,-0.10) |
| South Sudan | PMP | 4 | 1 | 1997 | 2008 | 0.000 | 0.60(0.50,0.70) |
| South Sudan | PMP | 4 | 2 | 2008 | 2012 | 0.849 | -0.10(-0.80,0.70) |
| South Sudan | PMP | 4 | 3 | 2012 | 2017 | 0.000 | 2.40(1.90,2.90) |
| South Sudan | PMP | 4 | 4 | 2017 | 2021 | 0.000 | -1.40(-1.90,-0.90) |
| Spain | APMP | 5 | 0 | 1990 | 1995 | 0.000 | -10.20(-11.60,-8.80) |
| Spain | APMP | 5 | 1 | 1995 | 1999 | 0.002 | -6.00(-9.20,-2.70) |
| Spain | APMP | 5 | 2 | 1999 | 2002 | 0.823 | -0.70(-7.40,6.40) |
| Spain | APMP | 5 | 3 | 2002 | 2009 | 0.000 | -6.00(-7.00,-4.80) |
| Spain | APMP | 5 | 4 | 2009 | 2012 | 0.532 | -2.10(-8.60,5.00) |
| Spain | APMP | 5 | 5 | 2012 | 2021 | 0.000 | -5.50(-6.10,-4.90) |
| Spain | HAP | 2 | 0 | 1990 | 1998 | 0.000 | -21.20(-22.50,-19.80) |
| Spain | HAP | 2 | 1 | 1998 | 2008 | 0.000 | -16.30(-17.40,-15.10) |
| Spain | HAP | 2 | 2 | 2008 | 2021 | 0.000 | -6.90(-7.60,-6.10) |
| Spain | PMP | 5 | 0 | 1990 | 1995 | 0.000 | -10.60(-11.90,-9.20) |
| Spain | PMP | 5 | 1 | 1995 | 1999 | 0.001 | -6.20(-9.40,-2.90) |
| Spain | PMP | 5 | 2 | 1999 | 2002 | 0.789 | -0.90(-7.50,6.20) |
| Spain | PMP | 5 | 3 | 2002 | 2009 | 0.000 | -6.00(-7.10,-4.90) |
| Spain | PMP | 5 | 4 | 2009 | 2012 | 0.533 | -2.00(-8.60,5.00) |
| Spain | PMP | 5 | 5 | 2012 | 2021 | 0.000 | -5.50(-6.10,-4.90) |
| Sri Lanka | APMP | 3 | 0 | 1990 | 1997 | 0.000 | 6.10(4.20,8.00) |
| Sri Lanka | APMP | 3 | 1 | 1997 | 2007 | 0.000 | -2.80(-4.00,-1.60) |
| Sri Lanka | APMP | 3 | 2 | 2007 | 2010 | 0.668 | 2.90(-10.10,17.60) |
| Sri Lanka | APMP | 3 | 3 | 2010 | 2021 | 0.000 | -2.00(-2.90,-1.10) |
| Sri Lanka | HAP | 3 | 0 | 1990 | 1996 | 0.056 | 2.60(-0.10,5.40) |
| Sri Lanka | HAP | 3 | 1 | 1996 | 2007 | 0.000 | -8.00(-9.20,-6.90) |
| Sri Lanka | HAP | 3 | 2 | 2007 | 2010 | 0.766 | -2.30(-16.50,14.40) |
| Sri Lanka | HAP | 3 | 3 | 2010 | 2021 | 0.000 | -14.30(-15.20,-13.40) |
| Sri Lanka | PMP | 3 | 0 | 1990 | 1996 | 0.013 | 3.10(0.70,5.60) |
| Sri Lanka | PMP | 3 | 1 | 1996 | 2007 | 0.000 | -7.00(-8.00,-5.90) |
| Sri Lanka | PMP | 3 | 2 | 2007 | 2010 | 0.763 | -2.00(-14.80,12.70) |
| Sri Lanka | PMP | 3 | 3 | 2010 | 2021 | 0.000 | -9.10(-10.00,-8.30) |
| Sudan | APMP | 3 | 0 | 1990 | 1995 | 0.000 | -5.10(-6.00,-4.20) |
| Sudan | APMP | 3 | 1 | 1995 | 2014 | 0.000 | 2.90(2.70,3.00) |
| Sudan | APMP | 3 | 2 | 2014 | 2018 | 0.012 | -2.70(-4.70,-0.70) |
| Sudan | APMP | 3 | 3 | 2018 | 2021 | 0.000 | -5.20(-7.10,-3.20) |
| Sudan | HAP | 5 | 0 | 1990 | 1995 | 0.000 | -1.90(-2.00,-1.80) |
| Sudan | HAP | 5 | 1 | 1995 | 2005 | 0.000 | -3.20(-3.20,-3.20) |
| Sudan | HAP | 5 | 2 | 2005 | 2012 | 0.000 | -4.40(-4.50,-4.30) |
| Sudan | HAP | 5 | 3 | 2012 | 2015 | 0.000 | -6.20(-6.60,-5.70) |
| Sudan | HAP | 5 | 4 | 2015 | 2019 | 0.000 | -8.50(-8.70,-8.30) |
| Sudan | HAP | 5 | 5 | 2019 | 2021 | 0.000 | -4.90(-5.30,-4.40) |
| Sudan | PMP | 1 | 0 | 1990 | 2014 | 0.000 | -2.30(-2.30,-2.20) |
| Sudan | PMP | 1 | 1 | 2014 | 2021 | 0.000 | -6.00(-6.30,-5.80) |
| Suriname | APMP | 4 | 0 | 1990 | 1996 | 0.000 | -1.70(-2.50,-0.90) |
| Suriname | APMP | 4 | 1 | 1996 | 2000 | 0.786 | 0.30(-2.10,2.80) |
| Suriname | APMP | 4 | 2 | 2000 | 2011 | 0.000 | -2.90(-3.20,-2.50) |
| Suriname | APMP | 4 | 3 | 2011 | 2018 | 0.001 | 1.50(0.70,2.30) |
| Suriname | APMP | 4 | 4 | 2018 | 2021 | 0.038 | -2.50(-4.80,-0.20) |
| Suriname | HAP | 5 | 0 | 1990 | 1996 | 0.000 | -2.30(-2.80,-1.80) |
| Suriname | HAP | 5 | 1 | 1996 | 2002 | 0.000 | -7.00(-7.60,-6.40) |
| Suriname | HAP | 5 | 2 | 2002 | 2005 | 0.000 | -9.40(-12.00,-6.60) |
| Suriname | HAP | 5 | 3 | 2005 | 2012 | 0.000 | -15.10(-15.50,-14.70) |
| Suriname | HAP | 5 | 4 | 2012 | 2015 | 0.000 | -11.20(-13.80,-8.50) |
| Suriname | HAP | 5 | 5 | 2015 | 2021 | 0.000 | -3.10(-3.60,-2.60) |
| Suriname | PMP | 3 | 0 | 1990 | 2001 | 0.000 | -2.00(-2.20,-1.90) |
| Suriname | PMP | 3 | 1 | 2001 | 2011 | 0.000 | -4.90(-5.20,-4.70) |
| Suriname | PMP | 3 | 2 | 2011 | 2019 | 0.005 | 0.50(0.20,0.80) |
| Suriname | PMP | 3 | 3 | 2019 | 2021 | 0.005 | -3.70(-6.00,-1.30) |
| Sweden | APMP | 3 | 0 | 1990 | 1997 | 0.000 | -8.50(-10.00,-7.00) |
| Sweden | APMP | 3 | 1 | 1997 | 2002 | 0.547 | -1.10(-4.90,2.80) |
| Sweden | APMP | 3 | 2 | 2002 | 2005 | 0.114 | -9.30(-19.80,2.60) |
| Sweden | APMP | 3 | 3 | 2005 | 2021 | 0.000 | -3.80(-4.30,-3.40) |
| Sweden | HAP | 1 | 0 | 1990 | 2010 | 0.000 | -12.40(-13.00,-11.70) |
| Sweden | HAP | 1 | 1 | 2010 | 2021 | 0.000 | -6.30(-8.00,-4.70) |
| Sweden | PMP | 3 | 0 | 1990 | 1997 | 0.000 | -8.50(-10.00,-7.00) |
| Sweden | PMP | 3 | 1 | 1997 | 2002 | 0.544 | -1.10(-4.90,2.80) |
| Sweden | PMP | 3 | 2 | 2002 | 2005 | 0.113 | -9.30(-19.80,2.60) |
| Sweden | PMP | 3 | 3 | 2005 | 2021 | 0.000 | -3.80(-4.30,-3.40) |
| Switzerland | APMP | 3 | 0 | 1990 | 1993 | 0.116 | -5.30(-11.60,1.50) |
| Switzerland | APMP | 3 | 1 | 1993 | 2001 | 0.659 | -0.40(-2.20,1.50) |
| Switzerland | APMP | 3 | 2 | 2001 | 2014 | 0.000 | -4.00(-4.80,-3.20) |
| Switzerland | APMP | 3 | 3 | 2014 | 2021 | 0.000 | -8.50(-10.10,-6.80) |
| Switzerland | HAP | 4 | 0 | 1990 | 2001 | 0.030 | -1.40(-2.70,-0.20) |
| Switzerland | HAP | 4 | 1 | 2001 | 2004 | 0.049 | -17.60(-32.00,-0.10) |
| Switzerland | HAP | 4 | 2 | 2004 | 2016 | 0.000 | -4.20(-5.50,-3.00) |
| Switzerland | HAP | 4 | 3 | 2016 | 2019 | 0.002 | -28.30(-40.90,-13.10) |
| Switzerland | HAP | 4 | 4 | 2019 | 2021 | 0.529 | -5.70(-22.20,14.30) |
| Switzerland | PMP | 3 | 0 | 1990 | 1993 | 0.116 | -5.30(-11.60,1.50) |
| Switzerland | PMP | 3 | 1 | 1993 | 2001 | 0.658 | -0.40(-2.20,1.50) |
| Switzerland | PMP | 3 | 2 | 2001 | 2014 | 0.000 | -4.00(-4.80,-3.20) |
| Switzerland | PMP | 3 | 3 | 2014 | 2021 | 0.000 | -8.50(-10.10,-6.80) |
| Syrian Arab Republic | APMP | 5 | 0 | 1990 | 1995 | 0.000 | -4.30(-5.90,-2.70) |
| Syrian Arab Republic | APMP | 5 | 1 | 1995 | 2000 | 0.000 | -12.50(-14.50,-10.40) |
| Syrian Arab Republic | APMP | 5 | 2 | 2000 | 2003 | 0.049 | -7.20(-13.90,0.00) |
| Syrian Arab Republic | APMP | 5 | 3 | 2003 | 2008 | 0.000 | 6.30(3.80,8.80) |
| Syrian Arab Republic | APMP | 5 | 4 | 2008 | 2015 | 0.027 | 1.40(0.20,2.70) |
| Syrian Arab Republic | APMP | 5 | 5 | 2015 | 2021 | 0.000 | -10.10(-11.20,-8.90) |
| Syrian Arab Republic | HAP | 4 | 0 | 1990 | 1995 | 0.000 | -15.20(-17.90,-12.50) |
| Syrian Arab Republic | HAP | 4 | 1 | 1995 | 2003 | 0.000 | -20.90(-22.40,-19.40) |
| Syrian Arab Republic | HAP | 4 | 2 | 2003 | 2010 | 0.000 | -14.50(-16.50,-12.40) |
| Syrian Arab Republic | HAP | 4 | 3 | 2010 | 2015 | 0.041 | -4.60(-8.80,-0.20) |
| Syrian Arab Republic | HAP | 4 | 4 | 2015 | 2021 | 0.000 | -17.80(-19.70,-15.80) |
| Syrian Arab Republic | PMP | 5 | 0 | 1990 | 1995 | 0.000 | -6.10(-7.60,-4.60) |
| Syrian Arab Republic | PMP | 5 | 1 | 1995 | 2000 | 0.000 | -13.30(-15.20,-11.20) |
| Syrian Arab Republic | PMP | 5 | 2 | 2000 | 2003 | 0.027 | -8.00(-14.50,-1.10) |
| Syrian Arab Republic | PMP | 5 | 3 | 2003 | 2008 | 0.000 | 5.40(3.00,7.80) |
| Syrian Arab Republic | PMP | 5 | 4 | 2008 | 2015 | 0.028 | 1.40(0.20,2.70) |
| Syrian Arab Republic | PMP | 5 | 5 | 2015 | 2021 | 0.000 | -10.10(-11.20,-9.00) |
| Taiwan (Province of China) | APMP | 5 | 0 | 1990 | 1993 | 0.786 | 1.00(-6.30,8.80) |
| Taiwan (Province of China) | APMP | 5 | 1 | 1993 | 1997 | 0.000 | 20.80(12.00,30.20) |
| Taiwan (Province of China) | APMP | 5 | 2 | 1997 | 2007 | 0.001 | -2.50(-3.80,-1.10) |
| Taiwan (Province of China) | APMP | 5 | 3 | 2007 | 2010 | 0.111 | -11.20(-23.60,3.10) |
| Taiwan (Province of China) | APMP | 5 | 4 | 2010 | 2015 | 0.189 | 3.10(-1.70,8.10) |
| Taiwan (Province of China) | APMP | 5 | 5 | 2015 | 2021 | 0.000 | -6.30(-8.70,-3.90) |
| Taiwan (Province of China) | HAP | 3 | 0 | 1990 | 1993 | 0.002 | -18.70(-28.10,-8.10) |
| Taiwan (Province of China) | HAP | 3 | 1 | 1993 | 1996 | 0.876 | -1.80(-23.20,25.40) |
| Taiwan (Province of China) | HAP | 3 | 2 | 1996 | 2009 | 0.000 | -15.00(-16.30,-13.80) |
| Taiwan (Province of China) | HAP | 3 | 3 | 2009 | 2021 | 0.000 | -9.00(-10.30,-7.60) |
| Taiwan (Province of China) | PMP | 5 | 0 | 1990 | 1993 | 0.184 | -4.50(-10.90,2.40) |
| Taiwan (Province of China) | PMP | 5 | 1 | 1993 | 1996 | 0.004 | 25.30(9.00,44.10) |
| Taiwan (Province of China) | PMP | 5 | 2 | 1996 | 2006 | 0.008 | -1.80(-3.00,-0.50) |
| Taiwan (Province of China) | PMP | 5 | 3 | 2006 | 2010 | 0.006 | -9.90(-15.90,-3.30) |
| Taiwan (Province of China) | PMP | 5 | 4 | 2010 | 2015 | 0.208 | 2.80(-1.70,7.40) |
| Taiwan (Province of China) | PMP | 5 | 5 | 2015 | 2021 | 0.000 | -6.30(-8.50,-4.00) |
| Tajikistan | APMP | 3 | 0 | 1990 | 1999 | 0.000 | -5.80(-6.30,-5.20) |
| Tajikistan | APMP | 3 | 1 | 1999 | 2005 | 0.188 | 0.90(-0.50,2.40) |
| Tajikistan | APMP | 3 | 2 | 2005 | 2014 | 0.000 | 8.70(7.90,9.40) |
| Tajikistan | APMP | 3 | 3 | 2014 | 2021 | 0.006 | -1.30(-2.10,-0.40) |
| Tajikistan | HAP | 4 | 0 | 1990 | 1996 | 0.000 | 3.70(3.10,4.20) |
| Tajikistan | HAP | 4 | 1 | 1996 | 2001 | 0.442 | 0.40(-0.70,1.40) |
| Tajikistan | HAP | 4 | 2 | 2001 | 2006 | 0.000 | -2.50(-3.50,-1.40) |
| Tajikistan | HAP | 4 | 3 | 2006 | 2015 | 0.000 | -4.10(-4.40,-3.70) |
| Tajikistan | HAP | 4 | 4 | 2015 | 2021 | 0.000 | -3.10(-3.60,-2.50) |
| Tajikistan | PMP | 2 | 0 | 1990 | 1998 | 0.000 | 1.80(1.40,2.20) |
| Tajikistan | PMP | 2 | 1 | 1998 | 2017 | 0.000 | -1.60(-1.70,-1.50) |
| Tajikistan | PMP | 2 | 2 | 2017 | 2021 | 0.000 | -3.10(-4.20,-2.00) |
| Thailand | APMP | 5 | 0 | 1990 | 1994 | 0.001 | 2.10(1.00,3.20) |
| Thailand | APMP | 5 | 1 | 1994 | 2005 | 0.000 | -4.00(-4.30,-3.80) |
| Thailand | APMP | 5 | 2 | 2005 | 2010 | 0.000 | -8.20(-9.10,-7.20) |
| Thailand | APMP | 5 | 3 | 2010 | 2013 | 0.001 | -6.20(-9.30,-3.00) |
| Thailand | APMP | 5 | 4 | 2013 | 2018 | 0.101 | -0.90(-1.90,0.20) |
| Thailand | APMP | 5 | 5 | 2018 | 2021 | 0.000 | -6.90(-8.50,-5.30) |
| Thailand | HAP | 2 | 0 | 1990 | 2006 | 0.000 | -12.40(-12.70,-12.00) |
| Thailand | HAP | 2 | 1 | 2006 | 2010 | 0.000 | -18.10(-22.20,-13.80) |
| Thailand | HAP | 2 | 2 | 2010 | 2021 | 0.000 | -13.60(-14.20,-13.10) |
| Thailand | PMP | 4 | 0 | 1990 | 2006 | 0.000 | -7.20(-7.30,-7.00) |
| Thailand | PMP | 4 | 1 | 2006 | 2010 | 0.000 | -10.50(-12.70,-8.40) |
| Thailand | PMP | 4 | 2 | 2010 | 2013 | 0.002 | -7.80(-12.10,-3.30) |
| Thailand | PMP | 4 | 3 | 2013 | 2018 | 0.012 | -2.00(-3.50,-0.50) |
| Thailand | PMP | 4 | 4 | 2018 | 2021 | 0.000 | -7.60(-9.80,-5.40) |
| Timor-Leste | APMP | 3 | 0 | 1990 | 2005 | 0.259 | 0.10(-0.10,0.30) |
| Timor-Leste | APMP | 3 | 1 | 2005 | 2009 | 0.000 | 13.50(10.80,16.40) |
| Timor-Leste | APMP | 3 | 2 | 2009 | 2015 | 0.039 | -1.20(-2.20,-0.10) |
| Timor-Leste | APMP | 3 | 3 | 2015 | 2021 | 0.000 | -3.70(-4.50,-2.90) |
| Timor-Leste | HAP | 5 | 0 | 1990 | 2002 | 0.000 | -2.10(-2.20,-2.00) |
| Timor-Leste | HAP | 5 | 1 | 2002 | 2006 | 0.000 | -4.10(-4.70,-3.40) |
| Timor-Leste | HAP | 5 | 2 | 2006 | 2010 | 0.000 | -8.50(-9.10,-7.90) |
| Timor-Leste | HAP | 5 | 3 | 2010 | 2014 | 0.000 | -3.90(-4.50,-3.20) |
| Timor-Leste | HAP | 5 | 4 | 2014 | 2019 | 0.001 | 0.90(0.40,1.30) |
| Timor-Leste | HAP | 5 | 5 | 2019 | 2021 | 0.199 | -0.80(-2.20,0.50) |
| Timor-Leste | PMP | 5 | 0 | 1990 | 2001 | 0.000 | -1.80(-1.90,-1.80) |
| Timor-Leste | PMP | 5 | 1 | 2001 | 2006 | 0.000 | -3.20(-3.50,-2.80) |
| Timor-Leste | PMP | 5 | 2 | 2006 | 2010 | 0.000 | -5.30(-5.80,-4.80) |
| Timor-Leste | PMP | 5 | 3 | 2010 | 2014 | 0.000 | -3.40(-3.90,-2.90) |
| Timor-Leste | PMP | 5 | 4 | 2014 | 2019 | 0.516 | -0.10(-0.40,0.20) |
| Timor-Leste | PMP | 5 | 5 | 2019 | 2021 | 0.020 | -1.30(-2.30,-0.20) |
| Togo | APMP | 5 | 0 | 1990 | 1995 | 0.000 | -4.00(-5.80,-2.30) |
| Togo | APMP | 5 | 1 | 1995 | 2000 | 0.030 | 2.90(0.30,5.60) |
| Togo | APMP | 5 | 2 | 2000 | 2005 | 0.000 | -7.60(-9.90,-5.20) |
| Togo | APMP | 5 | 3 | 2005 | 2011 | 0.883 | 0.10(-1.70,2.00) |
| Togo | APMP | 5 | 4 | 2011 | 2014 | 0.016 | 10.90(2.20,20.20) |
| Togo | APMP | 5 | 5 | 2014 | 2021 | 0.000 | -5.90(-6.90,-4.80) |
| Togo | HAP | 5 | 0 | 1990 | 1994 | 0.023 | -0.70(-1.20,-0.10) |
| Togo | HAP | 5 | 1 | 1994 | 1999 | 0.000 | -1.60(-2.10,-1.00) |
| Togo | HAP | 5 | 2 | 1999 | 2007 | 0.000 | -0.70(-0.90,-0.40) |
| Togo | HAP | 5 | 3 | 2007 | 2015 | 0.000 | -2.60(-2.80,-2.40) |
| Togo | HAP | 5 | 4 | 2015 | 2018 | 0.094 | -1.50(-3.20,0.30) |
| Togo | HAP | 5 | 5 | 2018 | 2021 | 0.000 | -3.30(-4.10,-2.40) |
| Togo | PMP | 4 | 0 | 1990 | 1996 | 0.000 | -1.30(-1.30,-1.20) |
| Togo | PMP | 4 | 1 | 1996 | 2002 | 0.000 | -1.00(-1.10,-0.90) |
| Togo | PMP | 4 | 2 | 2002 | 2010 | 0.000 | -1.60(-1.70,-1.60) |
| Togo | PMP | 4 | 3 | 2010 | 2016 | 0.000 | -1.50(-1.60,-1.40) |
| Togo | PMP | 4 | 4 | 2016 | 2021 | 0.000 | -3.20(-3.30,-3.10) |
| Tokelau | APMP | 1 | 0 | 1990 | 2018 | 0.000 | -4.10(-4.70,-3.50) |
| Tokelau | APMP | 1 | 1 | 2018 | 2021 | 0.000 | 96.30(60.50,140.10) |
| Tokelau | HAP | 1 | 0 | 1990 | 2019 | 0.000 | -11.60(-11.90,-11.40) |
| Tokelau | HAP | 1 | 1 | 2019 | 2021 | 0.000 | 181.90(142.60,227.70) |
| Tokelau | PMP | 1 | 0 | 1990 | 2018 | 0.000 | -4.40(-5.00,-3.80) |
| Tokelau | PMP | 1 | 1 | 2018 | 2021 | 0.000 | 97.20(61.60,140.70) |
| Tonga | APMP | 5 | 0 | 1990 | 1999 | 0.000 | 2.00(1.80,2.10) |
| Tonga | APMP | 5 | 1 | 1999 | 2003 | 0.568 | -0.20(-1.00,0.60) |
| Tonga | APMP | 5 | 2 | 2003 | 2007 | 0.000 | -1.80(-2.60,-1.00) |
| Tonga | APMP | 5 | 3 | 2007 | 2014 | 0.000 | -0.60(-0.90,-0.30) |
| Tonga | APMP | 5 | 4 | 2014 | 2019 | 0.000 | 2.50(2.00,3.00) |
| Tonga | APMP | 5 | 5 | 2019 | 2021 | 0.000 | -3.70(-5.20,-2.10) |
| Tonga | HAP | 5 | 0 | 1990 | 1998 | 0.000 | -2.90(-3.10,-2.70) |
| Tonga | HAP | 5 | 1 | 1998 | 2005 | 0.000 | -5.00(-5.30,-4.70) |
| Tonga | HAP | 5 | 2 | 2005 | 2011 | 0.000 | -1.60(-2.00,-1.20) |
| Tonga | HAP | 5 | 3 | 2011 | 2016 | 0.000 | -3.10(-3.60,-2.50) |
| Tonga | HAP | 5 | 4 | 2016 | 2019 | 0.000 | -7.80(-9.40,-6.10) |
| Tonga | HAP | 5 | 5 | 2019 | 2021 | 0.001 | -3.50(-5.20,-1.80) |
| Tonga | PMP | 3 | 0 | 1990 | 1998 | 0.000 | -2.40(-2.80,-2.10) |
| Tonga | PMP | 3 | 1 | 1998 | 2005 | 0.000 | -4.30(-4.80,-3.70) |
| Tonga | PMP | 3 | 2 | 2005 | 2015 | 0.000 | -1.80(-2.20,-1.50) |
| Tonga | PMP | 3 | 3 | 2015 | 2021 | 0.000 | -4.70(-5.30,-4.10) |
| Trinidad and Tobago | APMP | 3 | 0 | 1990 | 1995 | 0.000 | -3.00(-4.20,-1.80) |
| Trinidad and Tobago | APMP | 3 | 1 | 1995 | 2001 | 0.014 | 1.60(0.40,2.90) |
| Trinidad and Tobago | APMP | 3 | 2 | 2001 | 2006 | 0.000 | -7.40(-9.00,-5.70) |
| Trinidad and Tobago | APMP | 3 | 3 | 2006 | 2021 | 0.000 | -2.10(-2.40,-1.90) |
| Trinidad and Tobago | HAP | 3 | 0 | 1990 | 2000 | 0.000 | -5.50(-7.00,-4.00) |
| Trinidad and Tobago | HAP | 3 | 1 | 2000 | 2009 | 0.000 | -27.10(-28.60,-25.50) |
| Trinidad and Tobago | HAP | 3 | 2 | 2009 | 2015 | 0.000 | -17.40(-21.00,-13.60) |
| Trinidad and Tobago | HAP | 3 | 3 | 2015 | 2021 | 0.006 | -4.90(-8.00,-1.60) |
| Trinidad and Tobago | PMP | 3 | 0 | 1990 | 1995 | 0.000 | -3.00(-4.20,-1.80) |
| Trinidad and Tobago | PMP | 3 | 1 | 1995 | 2001 | 0.022 | 1.50(0.20,2.80) |
| Trinidad and Tobago | PMP | 3 | 2 | 2001 | 2006 | 0.000 | -7.50(-9.10,-5.80) |
| Trinidad and Tobago | PMP | 3 | 3 | 2006 | 2021 | 0.000 | -2.10(-2.40,-1.90) |
| Tunisia | APMP | 5 | 0 | 1990 | 1995 | 0.001 | -2.10(-3.20,-1.00) |
| Tunisia | APMP | 5 | 1 | 1995 | 2000 | 0.000 | 4.30(2.70,5.90) |
| Tunisia | APMP | 5 | 2 | 2000 | 2005 | 0.000 | -7.40(-8.80,-5.90) |
| Tunisia | APMP | 5 | 3 | 2005 | 2010 | 0.082 | -1.40(-2.90,0.20) |
| Tunisia | APMP | 5 | 4 | 2010 | 2014 | 0.000 | -7.50(-9.70,-5.10) |
| Tunisia | APMP | 5 | 5 | 2014 | 2021 | 0.000 | -5.50(-6.10,-4.90) |
| Tunisia | HAP | 4 | 0 | 1990 | 1996 | 0.000 | -13.30(-14.10,-12.50) |
| Tunisia | HAP | 4 | 1 | 1996 | 2001 | 0.000 | -17.60(-19.00,-16.20) |
| Tunisia | HAP | 4 | 2 | 2001 | 2009 | 0.000 | -22.90(-23.50,-22.40) |
| Tunisia | HAP | 4 | 3 | 2009 | 2019 | 0.000 | -17.10(-17.50,-16.70) |
| Tunisia | HAP | 4 | 4 | 2019 | 2021 | 0.003 | -8.40(-13.20,-3.40) |
| Tunisia | PMP | 5 | 0 | 1990 | 1995 | 0.000 | -5.30(-6.40,-4.30) |
| Tunisia | PMP | 5 | 1 | 1995 | 2000 | 0.138 | 1.20(-0.40,2.80) |
| Tunisia | PMP | 5 | 2 | 2000 | 2005 | 0.000 | -8.20(-9.60,-6.70) |
| Tunisia | PMP | 5 | 3 | 2005 | 2010 | 0.020 | -1.90(-3.40,-0.30) |
| Tunisia | PMP | 5 | 4 | 2010 | 2014 | 0.000 | -7.50(-9.80,-5.20) |
| Tunisia | PMP | 5 | 5 | 2014 | 2021 | 0.000 | -5.60(-6.20,-4.90) |
| Turkey | APMP | 2 | 0 | 1990 | 2010 | 0.000 | -4.00(-4.20,-3.80) |
| Turkey | APMP | 2 | 1 | 2010 | 2018 | 0.000 | -6.20(-7.20,-5.30) |
| Turkey | APMP | 2 | 2 | 2018 | 2021 | 0.000 | -12.20(-15.50,-8.90) |
| Turkey | HAP | 3 | 0 | 1990 | 2006 | 0.000 | -12.40(-12.70,-12.10) |
| Turkey | HAP | 3 | 1 | 2006 | 2010 | 0.000 | -20.90(-24.00,-17.60) |
| Turkey | HAP | 3 | 2 | 2010 | 2019 | 0.000 | -24.30(-25.00,-23.70) |
| Turkey | HAP | 3 | 3 | 2019 | 2021 | 0.006 | -11.10(-18.00,-3.70) |
| Turkey | PMP | 1 | 0 | 1990 | 2015 | 0.000 | -5.30(-5.40,-5.10) |
| Turkey | PMP | 1 | 1 | 2015 | 2021 | 0.000 | -9.60(-10.90,-8.30) |
| Turkmenistan | APMP | 5 | 0 | 1990 | 1995 | 0.000 | -4.70(-6.60,-2.80) |
| Turkmenistan | APMP | 5 | 1 | 1995 | 2000 | 0.006 | 4.40(1.50,7.50) |
| Turkmenistan | APMP | 5 | 2 | 2000 | 2005 | 0.149 | -2.00(-4.80,0.80) |
| Turkmenistan | APMP | 5 | 3 | 2005 | 2010 | 0.002 | 5.20(2.20,8.20) |
| Turkmenistan | APMP | 5 | 4 | 2010 | 2016 | 0.331 | -1.00(-2.90,1.10) |
| Turkmenistan | APMP | 5 | 5 | 2016 | 2021 | 0.000 | -6.10(-8.00,-4.20) |
| Turkmenistan | HAP | 4 | 0 | 1990 | 1994 | 0.274 | -2.20(-6.30,2.00) |
| Turkmenistan | HAP | 4 | 1 | 1994 | 2001 | 0.000 | 8.80(6.30,11.20) |
| Turkmenistan | HAP | 4 | 2 | 2001 | 2007 | 0.000 | -12.50(-15.10,-9.80) |
| Turkmenistan | HAP | 4 | 3 | 2007 | 2018 | 0.000 | -23.10(-23.90,-22.30) |
| Turkmenistan | HAP | 4 | 4 | 2018 | 2021 | 0.024 | -7.50(-13.50,-1.10) |
| Turkmenistan | PMP | 5 | 0 | 1990 | 1995 | 0.000 | -4.60(-6.50,-2.70) |
| Turkmenistan | PMP | 5 | 1 | 1995 | 2000 | 0.004 | 4.60(1.70,7.70) |
| Turkmenistan | PMP | 5 | 2 | 2000 | 2005 | 0.111 | -2.30(-5.00,0.60) |
| Turkmenistan | PMP | 5 | 3 | 2005 | 2010 | 0.004 | 4.70(1.70,7.80) |
| Turkmenistan | PMP | 5 | 4 | 2010 | 2016 | 0.287 | -1.00(-3.00,1.00) |
| Turkmenistan | PMP | 5 | 5 | 2016 | 2021 | 0.000 | -6.10(-8.00,-4.20) |
| Tuvalu | APMP | 5 | 0 | 1990 | 1994 | 0.232 | -0.30(-0.90,0.20) |
| Tuvalu | APMP | 5 | 1 | 1994 | 1999 | 0.000 | 2.20(1.60,2.80) |
| Tuvalu | APMP | 5 | 2 | 1999 | 2009 | 0.000 | -4.50(-4.60,-4.30) |
| Tuvalu | APMP | 5 | 3 | 2009 | 2014 | 0.123 | -0.40(-1.00,0.10) |
| Tuvalu | APMP | 5 | 4 | 2014 | 2018 | 0.000 | 5.80(4.80,6.80) |
| Tuvalu | APMP | 5 | 5 | 2018 | 2021 | 0.000 | -2.10(-3.00,-1.20) |
| Tuvalu | HAP | 2 | 0 | 1990 | 1998 | 0.000 | -7.20(-7.70,-6.60) |
| Tuvalu | HAP | 2 | 1 | 1998 | 2008 | 0.000 | -10.20(-10.60,-9.70) |
| Tuvalu | HAP | 2 | 2 | 2008 | 2021 | 0.000 | -5.50(-5.80,-5.20) |
| Tuvalu | PMP | 2 | 0 | 1990 | 1998 | 0.000 | -7.00(-7.50,-6.50) |
| Tuvalu | PMP | 2 | 1 | 1998 | 2009 | 0.000 | -9.60(-10.00,-9.20) |
| Tuvalu | PMP | 2 | 2 | 2009 | 2021 | 0.000 | -4.40(-4.70,-4.20) |
| Uganda | APMP | 5 | 0 | 1990 | 1993 | 0.238 | -0.90(-2.40,0.60) |
| Uganda | APMP | 5 | 1 | 1993 | 2001 | 0.000 | 2.60(2.20,3.10) |
| Uganda | APMP | 5 | 2 | 2001 | 2004 | 0.029 | -3.40(-6.30,-0.40) |
| Uganda | APMP | 5 | 3 | 2004 | 2016 | 0.000 | 1.70(1.50,1.90) |
| Uganda | APMP | 5 | 4 | 2016 | 2019 | 0.000 | -8.20(-11.00,-5.40) |
| Uganda | APMP | 5 | 5 | 2019 | 2021 | 0.882 | -0.20(-3.20,2.90) |
| Uganda | HAP | 2 | 0 | 1990 | 2007 | 0.000 | -0.40(-0.50,-0.20) |
| Uganda | HAP | 2 | 1 | 2007 | 2016 | 0.000 | -2.00(-2.50,-1.60) |
| Uganda | HAP | 2 | 2 | 2016 | 2021 | 0.000 | -3.80(-4.70,-2.90) |
| Uganda | PMP | 5 | 0 | 1990 | 1993 | 0.022 | -1.80(-3.30,-0.30) |
| Uganda | PMP | 5 | 1 | 1993 | 2001 | 0.279 | 0.20(-0.20,0.60) |
| Uganda | PMP | 5 | 2 | 2001 | 2008 | 0.006 | -0.80(-1.30,-0.30) |
| Uganda | PMP | 5 | 3 | 2008 | 2016 | 0.000 | -1.60(-2.00,-1.20) |
| Uganda | PMP | 5 | 4 | 2016 | 2019 | 0.002 | -5.20(-8.00,-2.20) |
| Uganda | PMP | 5 | 5 | 2019 | 2021 | 0.344 | -1.40(-4.30,1.70) |
| Ukraine | APMP | 4 | 0 | 1990 | 1997 | 0.003 | -1.80(-2.90,-0.70) |
| Ukraine | APMP | 4 | 1 | 1997 | 2004 | 0.000 | -10.10(-11.30,-8.80) |
| Ukraine | APMP | 4 | 2 | 2004 | 2008 | 0.067 | 3.90(-0.30,8.20) |
| Ukraine | APMP | 4 | 3 | 2008 | 2016 | 0.000 | -5.30(-6.30,-4.20) |
| Ukraine | APMP | 4 | 4 | 2016 | 2021 | 0.000 | -8.40(-10.10,-6.80) |
| Ukraine | HAP | 5 | 0 | 1990 | 1995 | 0.000 | 6.90(4.80,9.00) |
| Ukraine | HAP | 5 | 1 | 1995 | 1999 | 0.000 | 11.40(6.70,16.40) |
| Ukraine | HAP | 5 | 2 | 1999 | 2004 | 0.000 | -10.00(-12.40,-7.50) |
| Ukraine | HAP | 5 | 3 | 2004 | 2007 | 0.263 | -4.70(-12.70,4.10) |
| Ukraine | HAP | 5 | 4 | 2007 | 2012 | 0.000 | -16.50(-18.80,-14.10) |
| Ukraine | HAP | 5 | 5 | 2012 | 2021 | 0.000 | -9.00(-9.70,-8.30) |
| Ukraine | PMP | 3 | 0 | 1990 | 1998 | 0.071 | -0.90(-2.00,0.10) |
| Ukraine | PMP | 3 | 1 | 1998 | 2004 | 0.000 | -9.60(-11.50,-7.70) |
| Ukraine | PMP | 3 | 2 | 2004 | 2008 | 0.586 | 1.30(-3.40,6.20) |
| Ukraine | PMP | 3 | 3 | 2008 | 2021 | 0.000 | -6.90(-7.40,-6.50) |
| United Arab Emirates | APMP | 5 | 0 | 1990 | 1992 | 0.243 | -4.90(-12.80,3.80) |
| United Arab Emirates | APMP | 5 | 1 | 1992 | 1996 | 0.000 | -14.60(-18.20,-10.80) |
| United Arab Emirates | APMP | 5 | 2 | 1996 | 2002 | 0.005 | 3.10(1.10,5.10) |
| United Arab Emirates | APMP | 5 | 3 | 2002 | 2010 | 0.000 | -5.30(-6.40,-4.20) |
| United Arab Emirates | APMP | 5 | 4 | 2010 | 2017 | 0.011 | -2.00(-3.40,-0.50) |
| United Arab Emirates | APMP | 5 | 5 | 2017 | 2021 | 0.000 | -13.80(-16.10,-11.40) |
| United Arab Emirates | HAP | 4 | 0 | 1990 | 1992 | 0.872 | 2.40(-24.70,39.40) |
| United Arab Emirates | HAP | 4 | 1 | 1992 | 1995 | 0.098 | -22.60(-43.10,5.30) |
| United Arab Emirates | HAP | 4 | 2 | 1995 | 2011 | 0.000 | -12.00(-13.10,-10.80) |
| United Arab Emirates | HAP | 4 | 3 | 2011 | 2016 | 0.435 | -3.60(-12.60,6.20) |
| United Arab Emirates | HAP | 4 | 4 | 2016 | 2021 | 0.000 | -22.00(-27.10,-16.40) |
| United Arab Emirates | PMP | 5 | 0 | 1990 | 1992 | 0.242 | -4.90(-12.80,3.80) |
| United Arab Emirates | PMP | 5 | 1 | 1992 | 1996 | 0.000 | -14.60(-18.20,-10.80) |
| United Arab Emirates | PMP | 5 | 2 | 1996 | 2002 | 0.005 | 3.10(1.10,5.10) |
| United Arab Emirates | PMP | 5 | 3 | 2002 | 2010 | 0.000 | -5.30(-6.40,-4.20) |
| United Arab Emirates | PMP | 5 | 4 | 2010 | 2017 | 0.011 | -2.00(-3.40,-0.50) |
| United Arab Emirates | PMP | 5 | 5 | 2017 | 2021 | 0.000 | -13.80(-16.10,-11.40) |
| United Kingdom | APMP | 5 | 0 | 1990 | 1992 | 0.035 | -5.90(-11.10,-0.50) |
| United Kingdom | APMP | 5 | 1 | 1992 | 1997 | 0.415 | -0.70(-2.40,1.10) |
| United Kingdom | APMP | 5 | 2 | 1997 | 2006 | 0.000 | -4.00(-4.60,-3.40) |
| United Kingdom | APMP | 5 | 3 | 2006 | 2014 | 0.000 | -7.30(-8.00,-6.60) |
| United Kingdom | APMP | 5 | 4 | 2014 | 2018 | 0.045 | -2.80(-5.50,-0.10) |
| United Kingdom | APMP | 5 | 5 | 2018 | 2021 | 0.546 | 0.80(-2.00,3.70) |
| United Kingdom | HAP | 2 | 0 | 1990 | 2010 | 0.000 | -11.10(-11.30,-10.90) |
| United Kingdom | HAP | 2 | 1 | 2010 | 2019 | 0.000 | -6.00(-7.00,-5.10) |
| United Kingdom | HAP | 2 | 2 | 2019 | 2021 | 0.960 | 0.20(-8.50,9.80) |
| United Kingdom | PMP | 5 | 0 | 1990 | 1992 | 0.035 | -5.90(-11.10,-0.50) |
| United Kingdom | PMP | 5 | 1 | 1992 | 1997 | 0.412 | -0.70(-2.50,1.10) |
| United Kingdom | PMP | 5 | 2 | 1997 | 2006 | 0.000 | -4.00(-4.60,-3.40) |
| United Kingdom | PMP | 5 | 3 | 2006 | 2014 | 0.000 | -7.30(-8.00,-6.60) |
| United Kingdom | PMP | 5 | 4 | 2014 | 2018 | 0.045 | -2.90(-5.50,-0.10) |
| United Kingdom | PMP | 5 | 5 | 2018 | 2021 | 0.546 | 0.80(-2.00,3.70) |
| United Republic of Tanzania | APMP | 2 | 0 | 1990 | 1994 | 0.012 | -4.10(-7.10,-1.00) |
| United Republic of Tanzania | APMP | 2 | 1 | 1994 | 2014 | 0.000 | 3.70(3.40,4.00) |
| United Republic of Tanzania | APMP | 2 | 2 | 2014 | 2021 | 0.001 | -2.40(-3.70,-1.00) |
| United Republic of Tanzania | HAP | 3 | 0 | 1990 | 1995 | 0.000 | -1.60(-1.90,-1.40) |
| United Republic of Tanzania | HAP | 3 | 1 | 1995 | 2010 | 0.004 | 0.10(0.00,0.10) |
| United Republic of Tanzania | HAP | 3 | 2 | 2010 | 2018 | 0.000 | -1.50(-1.60,-1.30) |
| United Republic of Tanzania | HAP | 3 | 3 | 2018 | 2021 | 0.000 | -3.90(-4.50,-3.30) |
| United Republic of Tanzania | PMP | 5 | 0 | 1990 | 1993 | 0.000 | -1.90(-2.30,-1.50) |
| United Republic of Tanzania | PMP | 5 | 1 | 1993 | 1996 | 0.018 | -1.00(-1.70,-0.20) |
| United Republic of Tanzania | PMP | 5 | 2 | 1996 | 2010 | 0.000 | 0.30(0.30,0.40) |
| United Republic of Tanzania | PMP | 5 | 3 | 2010 | 2016 | 0.000 | -1.10(-1.30,-0.90) |
| United Republic of Tanzania | PMP | 5 | 4 | 2016 | 2019 | 0.000 | -2.30(-3.10,-1.60) |
| United Republic of Tanzania | PMP | 5 | 5 | 2019 | 2021 | 0.000 | -4.20(-4.90,-3.40) |
| United States Virgin Islands | APMP | 1 | 0 | 1990 | 2005 | 0.000 | -6.70(-7.10,-6.30) |
| United States Virgin Islands | APMP | 1 | 1 | 2005 | 2021 | 0.000 | -2.50(-2.90,-2.10) |
| United States Virgin Islands | HAP | 5 | 0 | 1990 | 1995 | 0.000 | -26.80(-30.90,-22.50) |
| United States Virgin Islands | HAP | 5 | 1 | 1995 | 2001 | 0.000 | -16.50(-21.10,-11.60) |
| United States Virgin Islands | HAP | 5 | 2 | 2001 | 2005 | 0.000 | -29.10(-37.60,-19.50) |
| United States Virgin Islands | HAP | 5 | 3 | 2005 | 2012 | 0.057 | 4.30(-0.10,8.90) |
| United States Virgin Islands | HAP | 5 | 4 | 2012 | 2015 | 0.300 | -12.10(-31.90,13.50) |
| United States Virgin Islands | HAP | 5 | 5 | 2015 | 2021 | 0.395 | 1.80(-2.50,6.30) |
| United States Virgin Islands | PMP | 1 | 0 | 1990 | 2005 | 0.000 | -6.80(-7.20,-6.30) |
| United States Virgin Islands | PMP | 1 | 1 | 2005 | 2021 | 0.000 | -2.50(-2.90,-2.10) |
| United States of America | APMP | 3 | 0 | 1990 | 1996 | 0.000 | -4.60(-5.50,-3.80) |
| United States of America | APMP | 3 | 1 | 1996 | 2006 | 0.000 | -2.00(-2.50,-1.60) |
| United States of America | APMP | 3 | 2 | 2006 | 2010 | 0.000 | -8.20(-10.50,-5.80) |
| United States of America | APMP | 3 | 3 | 2010 | 2021 | 0.000 | -3.70(-4.00,-3.30) |
| United States of America | HAP | 4 | 0 | 1990 | 2000 | 0.000 | -7.70(-8.30,-7.20) |
| United States of America | HAP | 4 | 1 | 2000 | 2007 | 0.000 | -3.70(-4.90,-2.40) |
| United States of America | HAP | 4 | 2 | 2007 | 2010 | 0.005 | -11.20(-17.80,-4.10) |
| United States of America | HAP | 4 | 3 | 2010 | 2017 | 0.002 | -2.20(-3.50,-0.90) |
| United States of America | HAP | 4 | 4 | 2017 | 2021 | 0.000 | -7.20(-9.50,-5.00) |
| United States of America | PMP | 3 | 0 | 1990 | 1996 | 0.000 | -4.60(-5.50,-3.80) |
| United States of America | PMP | 3 | 1 | 1996 | 2006 | 0.000 | -2.00(-2.50,-1.60) |
| United States of America | PMP | 3 | 2 | 2006 | 2010 | 0.000 | -8.20(-10.50,-5.80) |
| United States of America | PMP | 3 | 3 | 2010 | 2021 | 0.000 | -3.60(-4.00,-3.30) |
| Uruguay | APMP | 5 | 0 | 1990 | 1994 | 0.815 | -0.40(-4.10,3.40) |
| Uruguay | APMP | 5 | 1 | 1994 | 2000 | 0.000 | -6.90(-9.30,-4.50) |
| Uruguay | APMP | 5 | 2 | 2000 | 2003 | 0.759 | -1.70(-12.60,10.60) |
| Uruguay | APMP | 5 | 3 | 2003 | 2009 | 0.000 | -10.90(-13.20,-8.50) |
| Uruguay | APMP | 5 | 4 | 2009 | 2012 | 0.737 | 1.90(-9.40,14.60) |
| Uruguay | APMP | 5 | 5 | 2012 | 2021 | 0.000 | -4.10(-5.10,-3.00) |
| Uruguay | HAP | 5 | 0 | 1990 | 1993 | 0.001 | -10.80(-16.10,-5.10) |
| Uruguay | HAP | 5 | 1 | 1993 | 2000 | 0.000 | -16.50(-18.30,-14.80) |
| Uruguay | HAP | 5 | 2 | 2000 | 2003 | 0.639 | 2.80(-9.10,16.30) |
| Uruguay | HAP | 5 | 3 | 2003 | 2007 | 0.001 | -11.50(-16.80,-5.90) |
| Uruguay | HAP | 5 | 4 | 2007 | 2017 | 0.000 | -16.90(-17.90,-16.00) |
| Uruguay | HAP | 5 | 5 | 2017 | 2021 | 0.001 | -7.50(-11.00,-3.80) |
| Uruguay | PMP | 5 | 0 | 1990 | 1994 | 0.105 | -2.90(-6.30,0.70) |
| Uruguay | PMP | 5 | 1 | 1994 | 2000 | 0.000 | -8.20(-10.50,-5.80) |
| Uruguay | PMP | 5 | 2 | 2000 | 2003 | 0.819 | -1.20(-11.90,10.70) |
| Uruguay | PMP | 5 | 3 | 2003 | 2009 | 0.000 | -11.10(-13.40,-8.90) |
| Uruguay | PMP | 5 | 4 | 2009 | 2012 | 0.918 | 0.60(-10.30,12.70) |
| Uruguay | PMP | 5 | 5 | 2012 | 2021 | 0.000 | -4.40(-5.40,-3.40) |
| Uzbekistan | APMP | 4 | 0 | 1990 | 1994 | 0.004 | -2.90(-4.70,-1.10) |
| Uzbekistan | APMP | 4 | 1 | 1994 | 2005 | 0.019 | 0.60(0.10,1.00) |
| Uzbekistan | APMP | 4 | 2 | 2005 | 2008 | 0.002 | 10.70(4.40,17.50) |
| Uzbekistan | APMP | 4 | 3 | 2008 | 2015 | 0.000 | 5.00(3.90,6.00) |
| Uzbekistan | APMP | 4 | 4 | 2015 | 2021 | 0.000 | -3.10(-4.10,-2.20) |
| Uzbekistan | HAP | 3 | 0 | 1990 | 1994 | 0.020 | -3.60(-6.60,-0.60) |
| Uzbekistan | HAP | 3 | 1 | 1994 | 2000 | 0.098 | 1.80(-0.40,4.10) |
| Uzbekistan | HAP | 3 | 2 | 2000 | 2008 | 0.004 | -2.00(-3.30,-0.70) |
| Uzbekistan | HAP | 3 | 3 | 2008 | 2021 | 0.000 | -9.80(-10.30,-9.40) |
| Uzbekistan | PMP | 5 | 0 | 1990 | 1994 | 0.010 | -3.30(-5.60,-0.90) |
| Uzbekistan | PMP | 5 | 1 | 1994 | 2001 | 0.067 | 1.20(-0.10,2.50) |
| Uzbekistan | PMP | 5 | 2 | 2001 | 2004 | 0.392 | -3.10(-10.20,4.60) |
| Uzbekistan | PMP | 5 | 3 | 2004 | 2007 | 0.194 | 5.00(-2.70,13.30) |
| Uzbekistan | PMP | 5 | 4 | 2007 | 2016 | 0.041 | -0.90(-1.70,0.00) |
| Uzbekistan | PMP | 5 | 5 | 2016 | 2021 | 0.000 | -4.80(-6.40,-3.10) |
| Vanuatu | APMP | 5 | 0 | 1990 | 1994 | 0.000 | -6.20(-6.70,-5.80) |
| Vanuatu | APMP | 5 | 1 | 1994 | 2001 | 0.000 | -0.90(-1.20,-0.70) |
| Vanuatu | APMP | 5 | 2 | 2001 | 2006 | 0.000 | -2.30(-2.80,-1.90) |
| Vanuatu | APMP | 5 | 3 | 2006 | 2013 | 0.000 | 6.30(6.00,6.50) |
| Vanuatu | APMP | 5 | 4 | 2013 | 2017 | 0.000 | 3.70(3.00,4.50) |
| Vanuatu | APMP | 5 | 5 | 2017 | 2021 | 0.788 | -0.10(-0.50,0.40) |
| Vanuatu | HAP | 4 | 0 | 1990 | 1996 | 0.000 | -1.20(-1.40,-1.00) |
| Vanuatu | HAP | 4 | 1 | 1996 | 2004 | 0.748 | 0.00(-0.10,0.20) |
| Vanuatu | HAP | 4 | 2 | 2004 | 2008 | 0.000 | -3.00(-3.60,-2.50) |
| Vanuatu | HAP | 4 | 3 | 2008 | 2014 | 0.000 | -2.50(-2.80,-2.30) |
| Vanuatu | HAP | 4 | 4 | 2014 | 2021 | 0.000 | -0.90(-1.10,-0.70) |
| Vanuatu | PMP | 4 | 0 | 1990 | 1995 | 0.000 | -1.50(-1.80,-1.20) |
| Vanuatu | PMP | 4 | 1 | 1995 | 2004 | 0.135 | -0.10(-0.20,0.00) |
| Vanuatu | PMP | 4 | 2 | 2004 | 2008 | 0.000 | -2.90(-3.40,-2.30) |
| Vanuatu | PMP | 4 | 3 | 2008 | 2014 | 0.000 | -2.10(-2.40,-1.90) |
| Vanuatu | PMP | 4 | 4 | 2014 | 2021 | 0.000 | -0.70(-0.90,-0.60) |
| Venezuela (Bolivarian Republic of) | APMP | 5 | 0 | 1990 | 1992 | 0.138 | -8.70(-19.30,3.30) |
| Venezuela (Bolivarian Republic of) | APMP | 5 | 1 | 1992 | 1995 | 0.552 | 3.60(-8.40,17.20) |
| Venezuela (Bolivarian Republic of) | APMP | 5 | 2 | 1995 | 2007 | 0.000 | -3.90(-4.70,-3.10) |
| Venezuela (Bolivarian Republic of) | APMP | 5 | 3 | 2007 | 2014 | 0.755 | -0.30(-2.40,1.80) |
| Venezuela (Bolivarian Republic of) | APMP | 5 | 4 | 2014 | 2017 | 0.373 | 5.50(-6.80,19.30) |
| Venezuela (Bolivarian Republic of) | APMP | 5 | 5 | 2017 | 2021 | 0.001 | -7.60(-11.10,-3.90) |
| Venezuela (Bolivarian Republic of) | HAP | 3 | 0 | 1990 | 2006 | 0.000 | -2.10(-2.70,-1.40) |
| Venezuela (Bolivarian Republic of) | HAP | 3 | 1 | 2006 | 2015 | 0.000 | -10.50(-12.20,-8.70) |
| Venezuela (Bolivarian Republic of) | HAP | 3 | 2 | 2015 | 2018 | 0.061 | 18.50(-0.80,41.60) |
| Venezuela (Bolivarian Republic of) | HAP | 3 | 3 | 2018 | 2021 | 0.222 | -5.20(-13.30,3.60) |
| Venezuela (Bolivarian Republic of) | PMP | 5 | 0 | 1990 | 1992 | 0.137 | -8.70(-19.20,3.30) |
| Venezuela (Bolivarian Republic of) | PMP | 5 | 1 | 1992 | 1995 | 0.555 | 3.50(-8.40,17.10) |
| Venezuela (Bolivarian Republic of) | PMP | 5 | 2 | 1995 | 2007 | 0.000 | -3.90(-4.70,-3.10) |
| Venezuela (Bolivarian Republic of) | PMP | 5 | 3 | 2007 | 2014 | 0.577 | -0.60(-2.60,1.50) |
| Venezuela (Bolivarian Republic of) | PMP | 5 | 4 | 2014 | 2017 | 0.364 | 5.60(-6.70,19.40) |
| Venezuela (Bolivarian Republic of) | PMP | 5 | 5 | 2017 | 2021 | 0.001 | -7.50(-11.00,-3.80) |
| Viet Nam | APMP | 5 | 0 | 1990 | 1996 | 0.000 | -1.80(-2.20,-1.50) |
| Viet Nam | APMP | 5 | 1 | 1996 | 2002 | 0.001 | 0.90(0.40,1.30) |
| Viet Nam | APMP | 5 | 2 | 2002 | 2011 | 0.000 | 3.90(3.70,4.20) |
| Viet Nam | APMP | 5 | 3 | 2011 | 2015 | 0.056 | 1.00(0.00,2.00) |
| Viet Nam | APMP | 5 | 4 | 2015 | 2019 | 0.000 | 5.40(4.30,6.40) |
| Viet Nam | APMP | 5 | 5 | 2019 | 2021 | 0.000 | -5.30(-7.20,-3.30) |
| Viet Nam | HAP | 4 | 0 | 1990 | 1995 | 0.000 | -2.80(-3.10,-2.60) |
| Viet Nam | HAP | 4 | 1 | 1995 | 1998 | 0.000 | -4.70(-5.80,-3.60) |
| Viet Nam | HAP | 4 | 2 | 1998 | 2011 | 0.000 | -7.00(-7.00,-6.90) |
| Viet Nam | HAP | 4 | 3 | 2011 | 2019 | 0.000 | -8.60(-8.70,-8.50) |
| Viet Nam | HAP | 4 | 4 | 2019 | 2021 | 0.000 | -5.40(-6.50,-4.30) |
| Viet Nam | PMP | 5 | 0 | 1990 | 1995 | 0.000 | -2.70(-3.00,-2.50) |
| Viet Nam | PMP | 5 | 1 | 1995 | 1999 | 0.000 | -4.90(-5.40,-4.30) |
| Viet Nam | PMP | 5 | 2 | 1999 | 2005 | 0.000 | -6.40(-6.70,-6.20) |
| Viet Nam | PMP | 5 | 3 | 2005 | 2011 | 0.000 | -5.30(-5.50,-5.00) |
| Viet Nam | PMP | 5 | 4 | 2011 | 2014 | 0.000 | -7.10(-8.20,-5.90) |
| Viet Nam | PMP | 5 | 5 | 2014 | 2021 | 0.000 | -4.60(-4.80,-4.40) |
| Yemen | APMP | 4 | 0 | 1990 | 1995 | 0.439 | -0.80(-2.80,1.30) |
| Yemen | APMP | 4 | 1 | 1995 | 1999 | 0.020 | 5.80(1.00,10.80) |
| Yemen | APMP | 4 | 2 | 1999 | 2006 | 0.057 | -1.50(-3.00,0.10) |
| Yemen | APMP | 4 | 3 | 2006 | 2015 | 0.057 | 1.00(0.00,2.00) |
| Yemen | APMP | 4 | 4 | 2015 | 2021 | 0.000 | -5.50(-7.00,-4.00) |
| Yemen | HAP | 3 | 0 | 1990 | 1997 | 0.000 | -1.70(-1.90,-1.50) |
| Yemen | HAP | 3 | 1 | 1997 | 2003 | 0.000 | -3.40(-3.70,-3.10) |
| Yemen | HAP | 3 | 2 | 2003 | 2011 | 0.000 | -4.70(-4.90,-4.50) |
| Yemen | HAP | 3 | 3 | 2011 | 2021 | 0.000 | -4.40(-4.50,-4.20) |
| Yemen | PMP | 2 | 0 | 1990 | 1999 | 0.000 | -1.00(-1.40,-0.50) |
| Yemen | PMP | 2 | 1 | 1999 | 2017 | 0.000 | -2.90(-3.10,-2.70) |
| Yemen | PMP | 2 | 2 | 2017 | 2021 | 0.000 | -5.40(-6.80,-3.90) |
| Zambia | APMP | 5 | 0 | 1990 | 1994 | 0.002 | -3.80(-5.90,-1.70) |
| Zambia | APMP | 5 | 1 | 1994 | 2001 | 0.133 | 0.90(-0.30,2.10) |
| Zambia | APMP | 5 | 2 | 2001 | 2004 | 0.068 | -6.20(-12.50,0.50) |
| Zambia | APMP | 5 | 3 | 2004 | 2010 | 0.422 | -0.60(-2.10,1.00) |
| Zambia | APMP | 5 | 4 | 2010 | 2015 | 0.000 | 11.70(9.30,14.20) |
| Zambia | APMP | 5 | 5 | 2015 | 2021 | 0.077 | 1.00(-0.10,2.20) |
| Zambia | HAP | 5 | 0 | 1990 | 1994 | 0.000 | -1.10(-1.40,-0.90) |
| Zambia | HAP | 5 | 1 | 1994 | 2000 | 0.000 | 1.10(0.90,1.20) |
| Zambia | HAP | 5 | 2 | 2000 | 2004 | 0.000 | 2.50(2.20,2.90) |
| Zambia | HAP | 5 | 3 | 2004 | 2007 | 0.023 | 0.90(0.10,1.70) |
| Zambia | HAP | 5 | 4 | 2007 | 2011 | 0.000 | -2.00(-2.30,-1.60) |
| Zambia | HAP | 5 | 5 | 2011 | 2021 | 0.000 | -3.50(-3.60,-3.50) |
| Zambia | PMP | 5 | 0 | 1990 | 1994 | 0.000 | -1.40(-1.70,-1.10) |
| Zambia | PMP | 5 | 1 | 1994 | 2000 | 0.000 | 1.10(0.90,1.30) |
| Zambia | PMP | 5 | 2 | 2000 | 2005 | 0.000 | 2.00(1.60,2.30) |
| Zambia | PMP | 5 | 3 | 2005 | 2008 | 0.681 | -0.20(-1.10,0.80) |
| Zambia | PMP | 5 | 4 | 2008 | 2016 | 0.000 | -2.10(-2.20,-2.00) |
| Zambia | PMP | 5 | 5 | 2016 | 2021 | 0.000 | -3.20(-3.40,-3.00) |
| Zimbabwe | APMP | 5 | 0 | 1990 | 1993 | 0.078 | -1.70(-3.50,0.20) |
| Zimbabwe | APMP | 5 | 1 | 1993 | 2000 | 0.000 | 1.70(1.10,2.40) |
| Zimbabwe | APMP | 5 | 2 | 2000 | 2009 | 0.000 | -1.30(-1.70,-0.90) |
| Zimbabwe | APMP | 5 | 3 | 2009 | 2015 | 0.000 | 2.20(1.30,3.00) |
| Zimbabwe | APMP | 5 | 4 | 2015 | 2018 | 0.010 | -5.10(-8.60,-1.40) |
| Zimbabwe | APMP | 5 | 5 | 2018 | 2021 | 0.198 | -1.20(-3.00,0.70) |
| Zimbabwe | HAP | 5 | 0 | 1990 | 1992 | 0.000 | -3.80(-5.00,-2.50) |
| Zimbabwe | HAP | 5 | 1 | 1992 | 1997 | 0.021 | -0.50(-0.90,-0.10) |
| Zimbabwe | HAP | 5 | 2 | 1997 | 2001 | 0.007 | 0.90(0.30,1.60) |
| Zimbabwe | HAP | 5 | 3 | 2001 | 2005 | 0.000 | 4.50(3.80,5.20) |
| Zimbabwe | HAP | 5 | 4 | 2005 | 2010 | 0.000 | 3.50(3.10,4.00) |
| Zimbabwe | HAP | 5 | 5 | 2010 | 2021 | 0.000 | -1.20(-1.30,-1.20) |
| Zimbabwe | PMP | 5 | 0 | 1990 | 1992 | 0.000 | -3.50(-5.00,-2.00) |
| Zimbabwe | PMP | 5 | 1 | 1992 | 1996 | 0.176 | -0.50(-1.30,0.30) |
| Zimbabwe | PMP | 5 | 2 | 1996 | 2001 | 0.001 | 0.90(0.40,1.40) |
| Zimbabwe | PMP | 5 | 3 | 2001 | 2009 | 0.000 | 3.60(3.40,3.80) |
| Zimbabwe | PMP | 5 | 4 | 2009 | 2012 | 0.615 | 0.40(-1.10,1.90) |
| Zimbabwe | PMP | 5 | 5 | 2012 | 2021 | 0.000 | -1.40(-1.50,-1.20) |

Particulate matter pollution (PMP); Household air pollution (HAP); Ambient particulate matter pollution (APMP).
