# Supplementary material for: Global, regional, and national burden of neonatal diseases attributable to particulate matter pollution from 1990 to 2021
Source: Front Public Health. 2025 Jun 9;13:1556340. doi: 10.3389/fpubh.2025.1556340 (PMC12183240; doi:10.3389/fpubh.2025.1556340)
Supplement: Supplementary file 5 [file Table_3.DOCX]

Table S3 Health inequities in DALYs attributable to particulate matter pollution, household air pollution and ambient particulate matter pollution from 1990 to 2021.

|  | **Year** | **Particulate matter pollution** | **Ambient particulate matter pollution** | **Household air pollution from solid fuels** |
| --- | --- | --- | --- | --- |
| **Slope index** | 1990 | -1824.34 (-1927.32, -1721.37) | -124.65 (-177.22, -72.09) | -1701.47 (-1799.28, -1603.67) |
|  | 2021 | -1051.39 (-1146.15, -956.64) | -139.45 (-166.94, -111.96) | -904.67 (-1005.40, -803.93) |
| **Concentration index** | 1990 | -0.43 (-0.45, -0.40) | -0.18 (-0.23, -0.13) | -0.49 (-0.53, -0.46) |
|  | 2021 | -0.51 (-0.56, -0.47) | -0.34 (-0.40, -0.28) | -0.60 (-0.65, -0.55) |
