# Supplementary material for: Global, regional, and national burden of neonatal diseases attributable to particulate matter pollution from 1990 to 2021
Source: Front Public Health. 2025 Jun 9;13:1556340. doi: 10.3389/fpubh.2025.1556340 (PMC12183240; doi:10.3389/fpubh.2025.1556340)
Supplement: Supplementary file 6 [file Table_4.DOCX]

Table S4 Frontier analysis based on SDI and DALYs in 204 countries and territories.

| Location | Risk | SDI | Rate of DALYs in 2021 | Frontier DALYs | Effective difference |
| --- | --- | --- | --- | --- | --- |
| Afghanistan | Particulate matter pollution | 0.3372 | 751.48 (461.89 to 1076.95) | 444.82 | 306.66 |
| Albania | Particulate matter pollution | 0.70685 | 130.35 (46.53 to 231.76) | 16.29 | 114.06 |
| Algeria | Particulate matter pollution | 0.659501 | 191.34 (81.89 to 324.47) | 24.66 | 166.68 |
| American Samoa | Particulate matter pollution | 0.723728 | 28.64 (3.83 to 65.2) | 13.04 | 15.6 |
| Andorra | Particulate matter pollution | 0.869444 | 2.57 (0.02 to 6.88) | 2.57 | 0 |
| Angola | Particulate matter pollution | 0.453722 | 642.67 (355 to 976.9) | 299.72 | 342.96 |
| Antigua and Barbuda | Particulate matter pollution | 0.749887 | 82.93 (28.79 to 141.51) | 6.93 | 76 |
| Argentina | Particulate matter pollution | 0.723123 | 64.01 (18.15 to 117.72) | 12.34 | 51.67 |
| Armenia | Particulate matter pollution | 0.701833 | 131.65 (68.18 to 205.86) | 18.56 | 113.09 |
| Australia | Particulate matter pollution | 0.844253 | 11.39 (0.5 to 30.31) | 3.96 | 7.43 |
| Austria | Particulate matter pollution | 0.853837 | 14.93 (1.11 to 33.62) | 4.46 | 10.47 |
| Azerbaijan | Particulate matter pollution | 0.694851 | 279.42 (106.52 to 494.85) | 18.86 | 260.55 |
| Bahamas | Particulate matter pollution | 0.805021 | 80.33 (29.3 to 148.23) | 6.63 | 73.71 |
| Bahrain | Particulate matter pollution | 0.753043 | 70.24 (44.11 to 103.28) | 6.82 | 63.43 |
| Bangladesh | Particulate matter pollution | 0.492421 | 979.67 (619.2 to 1421.56) | 243.3 | 736.38 |
| Barbados | Particulate matter pollution | 0.746749 | 161.18 (59.91 to 301.73) | 6.84 | 154.34 |
| Belarus | Particulate matter pollution | 0.784485 | 18.47 (3.73 to 35.87) | 6.58 | 11.89 |
| Belgium | Particulate matter pollution | 0.853654 | 15.29 (1.72 to 34.87) | 4.09 | 11.2 |
| Belize | Particulate matter pollution | 0.610229 | 216.63 (101.93 to 351.82) | 32.79 | 183.84 |
| Benin | Particulate matter pollution | 0.373487 | 1476.68 (922.62 to 2071.26) | 344.71 | 1131.97 |
| Bermuda | Particulate matter pollution | 0.821365 | 8.37 (0.02 to 24.09) | 6.62 | 1.74 |
| Bhutan | Particulate matter pollution | 0.473062 | 511.18 (269.4 to 770.75) | 294.59 | 216.58 |
| Bolivia (Plurinational State of) | Particulate matter pollution | 0.599011 | 239.06 (125.3 to 395.54) | 32.43 | 206.63 |
| Bosnia and Herzegovina | Particulate matter pollution | 0.723078 | 103.92 (45.51 to 165.11) | 10.43 | 93.5 |
| Botswana | Particulate matter pollution | 0.642722 | 515.71 (255.99 to 870.26) | 24.95 | 490.77 |
| Brazil | Particulate matter pollution | 0.653044 | 77.51 (55.3 to 103.55) | 24.25 | 53.26 |
| Brunei Darussalam | Particulate matter pollution | 0.810234 | 27.32 (2.58 to 63.23) | 6.62 | 20.7 |
| Bulgaria | Particulate matter pollution | 0.768151 | 42.23 (14.34 to 77.58) | 6.7 | 35.53 |
| Burkina Faso | Particulate matter pollution | 0.285118 | 1215.15 (826.62 to 1666.86) | 830.27 | 384.88 |
| Burundi | Particulate matter pollution | 0.289374 | 1104.6 (703.83 to 1615.4) | 837.78 | 266.83 |
| Cabo Verde | Particulate matter pollution | 0.533535 | 309.01 (169.88 to 495.27) | 85.22 | 223.79 |
| Cambodia | Particulate matter pollution | 0.473621 | 607.42 (374.41 to 907.11) | 293.63 | 313.79 |
| Cameroon | Particulate matter pollution | 0.479691 | 961.04 (589.94 to 1375.9) | 285.31 | 675.73 |
| Canada | Particulate matter pollution | 0.873171 | 12.87 (0.01 to 35.91) | 3.83 | 9.03 |
| Central African Republic | Particulate matter pollution | 0.309168 | 1725.64 (1104.67 to 2470.91) | 642.65 | 1082.99 |
| Chad | Particulate matter pollution | 0.240436 | 1451.21 (963.8 to 2064.39) | 1034.76 | 416.45 |
| Chile | Particulate matter pollution | 0.771515 | 59.3 (22.66 to 100.07) | 6.23 | 53.07 |
| China | Particulate matter pollution | 0.72163 | 78.04 (62.34 to 97.46) | 12.56 | 65.47 |
| Colombia | Particulate matter pollution | 0.655443 | 67.11 (23.48 to 128.58) | 21.8 | 45.31 |
| Comoros | Particulate matter pollution | 0.475979 | 1254.59 (796.51 to 1828.69) | 290.45 | 964.14 |
| Congo | Particulate matter pollution | 0.583075 | 622.02 (367.98 to 933.79) | 41.56 | 580.45 |
| Cook Islands | Particulate matter pollution | 0.77911 | 8.5 (0.74 to 20.59) | 6.65 | 1.85 |
| Costa Rica | Particulate matter pollution | 0.70034 | 51.32 (7.07 to 102.81) | 18.35 | 32.98 |
| Coted'Ivoire | Particulate matter pollution | 0.425942 | 1405.04 (900.6 to 1992.41) | 298.32 | 1106.72 |
| Croatia | Particulate matter pollution | 0.798341 | 27.01 (5.26 to 55.07) | 6.6 | 20.41 |
| Cuba | Particulate matter pollution | 0.66873 | 25.55 (7.02 to 46.23) | 20.42 | 5.14 |
| Cyprus | Particulate matter pollution | 0.835631 | 12.78 (3.89 to 23.96) | 5.76 | 7.02 |
| Czechia | Particulate matter pollution | 0.82845 | 12.46 (2.2 to 26.09) | 6.57 | 5.89 |
| Democratic People's Republic of Korea | Particulate matter pollution | 0.569855 | 196.77 (116.37 to 291.86) | 42.33 | 154.44 |
| Democratic Republic of the Congo | Particulate matter pollution | 0.38318 | 902.19 (553.36 to 1311.43) | 327.52 | 574.67 |
| Denmark | Particulate matter pollution | 0.896424 | 15.65 (0.28 to 38.8) | 3.63 | 12.02 |
| Djibouti | Particulate matter pollution | 0.487958 | 545.36 (308.72 to 899.29) | 244.6 | 300.76 |
| Dominica | Particulate matter pollution | 0.746967 | 321.51 (137.12 to 585.69) | 6.9 | 314.61 |
| Dominican Republic | Particulate matter pollution | 0.619388 | 335.67 (127.77 to 572.07) | 28.61 | 307.05 |
| Ecuador | Particulate matter pollution | 0.661017 | 89.96 (28.77 to 166.79) | 26.42 | 63.54 |
| Egypt | Particulate matter pollution | 0.606787 | 107.95 (63.9 to 164.65) | 32.07 | 75.88 |
| El Salvador | Particulate matter pollution | 0.563775 | 78.13 (28.21 to 146.32) | 48.03 | 30.1 |
| Equatorial Guinea | Particulate matter pollution | 0.657857 | 483.83 (237.2 to 830.15) | 21.81 | 462.03 |
| Eritrea | Particulate matter pollution | 0.403864 | 820.1 (520.43 to 1182.94) | 321.12 | 498.98 |
| Estonia | Particulate matter pollution | 0.844918 | 4.17 (0.18 to 10.6) | 3.93 | 0.24 |
| Eswatini | Particulate matter pollution | 0.58546 | 493.9 (268.49 to 766.02) | 36.32 | 457.58 |
| Ethiopia | Particulate matter pollution | 0.358823 | 1305.54 (988.86 to 1739.05) | 391.5 | 914.04 |
| Fiji | Particulate matter pollution | 0.675052 | 167.2 (60.65 to 301.3) | 21.3 | 145.9 |
| Finland | Particulate matter pollution | 0.859831 | 5.11 (0 to 15.52) | 4.47 | 0.64 |
| France | Particulate matter pollution | 0.838365 | 14.98 (1.12 to 38.04) | 5.94 | 9.04 |
| Gabon | Particulate matter pollution | 0.634691 | 396.13 (181.81 to 659.29) | 23.08 | 373.05 |
| Gambia | Particulate matter pollution | 0.409714 | 1203.5 (803.28 to 1666.45) | 321.84 | 881.65 |
| Georgia | Particulate matter pollution | 0.732474 | 91.94 (34.45 to 157.31) | 10.42 | 81.52 |
| Germany | Particulate matter pollution | 0.902957 | 17.26 (2.27 to 36.79) | 3.63 | 13.63 |
| Ghana | Particulate matter pollution | 0.56493 | 908.17 (560.21 to 1374.18) | 47.36 | 860.81 |
| Greece | Particulate matter pollution | 0.791854 | 25.22 (6.48 to 46.14) | 6.59 | 18.63 |
| Greenland | Particulate matter pollution | 0.82621 | 24.46 (0.02 to 66.16) | 6.61 | 17.85 |
| Grenada | Particulate matter pollution | 0.668993 | 181.52 (61.13 to 328.5) | 20.46 | 161.07 |
| Guam | Particulate matter pollution | 0.803982 | 48.85 (5.3 to 105.31) | 6.61 | 42.23 |
| Guatemala | Particulate matter pollution | 0.539972 | 271.4 (158.53 to 393.26) | 63.75 | 207.65 |
| Guinea | Particulate matter pollution | 0.336401 | 1328.94 (848.91 to 1886.34) | 443.5 | 885.44 |
| Guinea-Bissau | Particulate matter pollution | 0.35311 | 1468.43 (922.98 to 2057.32) | 404.57 | 1063.86 |
| Guyana | Particulate matter pollution | 0.650812 | 397 (173.69 to 715.42) | 24.88 | 372.12 |
| Haiti | Particulate matter pollution | 0.448278 | 1093.74 (723.84 to 1502.37) | 299.32 | 794.42 |
| Honduras | Particulate matter pollution | 0.513037 | 249.49 (145.86 to 383.63) | 185.67 | 63.82 |
| Hungary | Particulate matter pollution | 0.790755 | 21.07 (4.59 to 42.02) | 7.15 | 13.92 |
| Iceland | Particulate matter pollution | 0.876362 | 3.64 (0 to 10.24) | 3.64 | 0 |
| India | Particulate matter pollution | 0.575402 | 1046.74 (843.93 to 1309.48) | 46.89 | 999.85 |
| Indonesia | Particulate matter pollution | 0.656868 | 301.38 (227.23 to 386.92) | 23.88 | 277.5 |
| Iran (Islamic Republic of) | Particulate matter pollution | 0.697207 | 80.36 (62.57 to 101.33) | 18.07 | 62.28 |
| Iraq | Particulate matter pollution | 0.662626 | 321.61 (171.78 to 503.93) | 22.14 | 299.47 |
| Ireland | Particulate matter pollution | 0.873754 | 10.17 (0.01 to 28.99) | 3.84 | 6.33 |
| Israel | Particulate matter pollution | 0.809012 | 12.46 (4.3 to 23.2) | 6.6 | 5.86 |
| Italy | Particulate matter pollution | 0.805774 | 16.25 (11.05 to 21.85) | 6.5 | 9.75 |
| Jamaica | Particulate matter pollution | 0.683263 | 227.44 (92.45 to 416.57) | 18.5 | 208.94 |
| Japan | Particulate matter pollution | 0.871242 | 4.77 (3.67 to 5.96) | 4.12 | 0.65 |
| Jordan | Particulate matter pollution | 0.725307 | 152.11 (75.23 to 251.63) | 13.12 | 138.99 |
| Kazakhstan | Particulate matter pollution | 0.725144 | 68.21 (26.54 to 119.03) | 14.03 | 54.18 |
| Kenya | Particulate matter pollution | 0.523768 | 737.48 (585.53 to 913.59) | 103.31 | 634.17 |
| Kiribati | Particulate matter pollution | 0.527187 | 491.2 (294.64 to 733.15) | 96.49 | 394.71 |
| Kuwait | Particulate matter pollution | 0.846651 | 112.51 (59.44 to 171.26) | 4.2 | 108.31 |
| Kyrgyzstan | Particulate matter pollution | 0.603979 | 260.1 (128.93 to 408.05) | 32.71 | 227.38 |
| Lao People's Democratic Republic | Particulate matter pollution | 0.489136 | 769.63 (471.08 to 1154.22) | 244.68 | 524.95 |
| Latvia | Particulate matter pollution | 0.830664 | 17.3 (1.59 to 35.83) | 6.42 | 10.88 |
| Lebanon | Particulate matter pollution | 0.744746 | 64.94 (25.5 to 116.76) | 6.9 | 58.05 |
| Lesotho | Particulate matter pollution | 0.510393 | 1247.5 (789.51 to 1829) | 180.63 | 1066.88 |
| Liberia | Particulate matter pollution | 0.352442 | 1287.16 (829.97 to 1851.62) | 444.2 | 842.96 |
| Libya | Particulate matter pollution | 0.725771 | 233.2 (113.25 to 382.78) | 12.92 | 220.28 |
| Lithuania | Particulate matter pollution | 0.856484 | 10.06 (0.74 to 23.2) | 4.45 | 5.61 |
| Luxembourg | Particulate matter pollution | 0.884429 | 9.3 (0.75 to 22.12) | 3.64 | 5.67 |
| Madagascar | Particulate matter pollution | 0.400247 | 990.91 (673.93 to 1423.67) | 323.15 | 667.76 |
| Malawi | Particulate matter pollution | 0.384554 | 1024.13 (658.56 to 1374.94) | 333.64 | 690.49 |
| Malaysia | Particulate matter pollution | 0.742524 | 43.94 (15.17 to 79.59) | 7.39 | 36.54 |
| Maldives | Particulate matter pollution | 0.650887 | 86.4 (20.44 to 169.98) | 23.46 | 62.94 |
| Mali | Particulate matter pollution | 0.26858 | 2132.85 (1457.19 to 2849.72) | 949.52 | 1183.33 |
| Malta | Particulate matter pollution | 0.801585 | 27.87 (2.73 to 61.01) | 6.77 | 21.1 |
| Marshall Islands | Particulate matter pollution | 0.574091 | 196.85 (96.77 to 317.43) | 46.17 | 150.68 |
| Mauritania | Particulate matter pollution | 0.498945 | 882.92 (593.16 to 1205.14) | 227.88 | 655.03 |
| Mauritius | Particulate matter pollution | 0.71826 | 59.27 (12.35 to 120.97) | 13.86 | 45.41 |
| Mexico | Particulate matter pollution | 0.664575 | 95.3 (71.3 to 120.75) | 19.33 | 75.97 |
| Micronesia (Federated States of) | Particulate matter pollution | 0.587535 | 162.1 (73.2 to 259.64) | 41.44 | 120.66 |
| Monaco | Particulate matter pollution | 0.908263 | 7.99 (0.79 to 18.06) | 3.64 | 4.36 |
| Mongolia | Particulate matter pollution | 0.617622 | 210.96 (101.29 to 331.97) | 28.99 | 181.97 |
| Montenegro | Particulate matter pollution | 0.795801 | 34.91 (12.2 to 62.49) | 6.53 | 28.38 |
| Morocco | Particulate matter pollution | 0.562698 | 170.27 (70.92 to 276.66) | 47.9 | 122.37 |
| Mozambique | Particulate matter pollution | 0.326463 | 1318.67 (844.24 to 1882.54) | 477.81 | 840.86 |
| Myanmar | Particulate matter pollution | 0.533901 | 699.25 (434.9 to 1051.08) | 85.47 | 613.78 |
| Namibia | Particulate matter pollution | 0.617565 | 488.73 (260.23 to 805.78) | 30.2 | 458.54 |
| Nauru | Particulate matter pollution | 0.625178 | 54.96 (1.39 to 135.26) | 27.94 | 27.01 |
| Nepal | Particulate matter pollution | 0.433175 | 875.39 (480.36 to 1312.25) | 295.9 | 579.49 |
| Netherlands | Particulate matter pollution | 0.888464 | 18.56 (1.34 to 42.42) | 3.64 | 14.92 |
| New Zealand | Particulate matter pollution | 0.849442 | 14.29 (1.6 to 32.96) | 4.52 | 9.77 |
| Nicaragua | Particulate matter pollution | 0.523958 | 236.06 (123.18 to 366.89) | 107.95 | 128.11 |
| Niger | Particulate matter pollution | 0.168073 | 1226.23 (767.95 to 1724.18) | 1226.11 | 0.12 |
| Nigeria | Particulate matter pollution | 0.503391 | 1625.08 (1326.07 to 1958.31) | 233.86 | 1391.22 |
| Niue | Particulate matter pollution | 0.726222 | 103.44 (9.19 to 222.14) | 10.37 | 93.07 |
| North Macedonia | Particulate matter pollution | 0.75063 | 90.99 (38.24 to 146.98) | 7.15 | 83.84 |
| Northern Mariana Islands | Particulate matter pollution | 0.771535 | 26.83 (2.91 to 62.32) | 6.78 | 20.05 |
| Norway | Particulate matter pollution | 0.916133 | 5.75 (3.17 to 9.39) | 3.63 | 2.12 |
| Oman | Particulate matter pollution | 0.773392 | 127.47 (75.73 to 192.31) | 6.52 | 120.95 |
| Pakistan | Particulate matter pollution | 0.504029 | 1610.2 (1128.83 to 2093.59) | 240.29 | 1369.92 |
| Palau | Particulate matter pollution | 0.754047 | 51.79 (2.12 to 128.16) | 6.79 | 45 |
| Palestine | Particulate matter pollution | 0.631012 | 130.28 (65.7 to 217.65) | 23.95 | 106.33 |
| Panama | Particulate matter pollution | 0.708865 | 57.19 (13.54 to 114.56) | 16.8 | 40.38 |
| Papua New Guinea | Particulate matter pollution | 0.417797 | 648.93 (415.74 to 893) | 308.08 | 340.85 |
| Paraguay | Particulate matter pollution | 0.635718 | 127.06 (38.76 to 241.23) | 26.67 | 100.4 |
| Peru | Particulate matter pollution | 0.662054 | 152.88 (74.18 to 260.75) | 18.82 | 134.06 |
| Philippines | Particulate matter pollution | 0.651219 | 383.52 (296.32 to 478.42) | 25.85 | 357.67 |
| Poland | Particulate matter pollution | 0.812043 | 31.11 (22.14 to 41.74) | 6.56 | 24.54 |
| Portugal | Particulate matter pollution | 0.744152 | 8.88 (0.67 to 21.46) | 6.97 | 1.91 |
| Puerto Rico | Particulate matter pollution | 0.825526 | 19.82 (0.01 to 49.92) | 6.56 | 13.26 |
| Qatar | Particulate matter pollution | 0.846861 | 62.92 (37.83 to 92.53) | 3.98 | 58.93 |
| Republic of Korea | Particulate matter pollution | 0.886675 | 18.74 (6.89 to 33.16) | 3.63 | 15.11 |
| Republic of Moldova | Particulate matter pollution | 0.732215 | 64.44 (21.07 to 122.71) | 11.16 | 53.28 |
| Romania | Particulate matter pollution | 0.768454 | 40.96 (14.09 to 70.72) | 6.7 | 34.26 |
| Russian Federation | Particulate matter pollution | 0.808536 | 22.16 (17.78 to 27.03) | 6.89 | 15.27 |
| Rwanda | Particulate matter pollution | 0.435589 | 768.01 (491.68 to 1100.26) | 305.27 | 462.75 |
| Saint Kitts and Nevis | Particulate matter pollution | 0.754987 | 62.84 (3.65 to 148.4) | 6.81 | 56.03 |
| Saint Lucia | Particulate matter pollution | 0.67251 | 264.46 (96.24 to 493.57) | 19.08 | 245.39 |
| Saint Vincent and the Grenadines | Particulate matter pollution | 0.637196 | 180.94 (65.17 to 325.56) | 26.29 | 154.65 |
| Samoa | Particulate matter pollution | 0.593393 | 202.83 (115.44 to 307.55) | 35.56 | 167.27 |
| San Marino | Particulate matter pollution | 0.888005 | 5.59 (0.23 to 14.79) | 3.64 | 1.95 |
| Sao Tome and Principe | Particulate matter pollution | 0.505414 | 282.4 (156.49 to 430.36) | 240.27 | 42.12 |
| Saudi Arabia | Particulate matter pollution | 0.815143 | 62.28 (35.74 to 98.16) | 6.53 | 55.76 |
| Senegal | Particulate matter pollution | 0.408054 | 1011.67 (660.05 to 1443.53) | 319.11 | 692.57 |
| Serbia | Particulate matter pollution | 0.792416 | 67.67 (29.93 to 115.08) | 6.69 | 60.98 |
| Seychelles | Particulate matter pollution | 0.730151 | 58.14 (6.14 to 121.99) | 13.03 | 45.12 |
| Sierra Leone | Particulate matter pollution | 0.358666 | 1612.35 (1099.13 to 2197.43) | 380.4 | 1231.95 |
| Singapore | Particulate matter pollution | 0.856098 | 8.68 (2.62 to 15.91) | 4.09 | 4.6 |
| Slovakia | Particulate matter pollution | 0.810611 | 29.69 (8.06 to 55.77) | 6.52 | 23.17 |
| Slovenia | Particulate matter pollution | 0.842431 | 9.16 (1.79 to 19.64) | 3.98 | 5.18 |
| Solomon Islands | Particulate matter pollution | 0.42936 | 280.22 (164.04 to 412.87) | 280.22 | 0 |
| Somalia | Particulate matter pollution | 0.077688 | 1413.63 (932.26 to 1957.76) | 1413.63 | 0 |
| South Africa | Particulate matter pollution | 0.679627 | 484.63 (357.95 to 642.13) | 23.24 | 461.39 |
| South Sudan | Particulate matter pollution | 0.278371 | 1965.21 (1290.14 to 2746.51) | 938.59 | 1026.62 |
| Spain | Particulate matter pollution | 0.769284 | 10.53 (1.33 to 22.97) | 6.77 | 3.75 |
| Sri Lanka | Particulate matter pollution | 0.701535 | 105.14 (52.66 to 175.09) | 18.56 | 86.59 |
| Sudan | Particulate matter pollution | 0.54195 | 671.53 (372.93 to 1059.29) | 48.93 | 622.59 |
| Suriname | Particulate matter pollution | 0.633666 | 405.26 (204.34 to 664.01) | 32.02 | 373.24 |
| Sweden | Particulate matter pollution | 0.88688 | 5.11 (0.57 to 11.54) | 3.61 | 1.49 |
| Switzerland | Particulate matter pollution | 0.933059 | 13.28 (0.32 to 32.17) | 3.62 | 9.65 |
| Syrian Arab Republic | Particulate matter pollution | 0.623004 | 95.2 (46.62 to 152.26) | 29.12 | 66.07 |
| Taiwan (Province of China) | Particulate matter pollution | 0.874747 | 26.19 (6.96 to 50.03) | 3.83 | 22.36 |
| Tajikistan | Particulate matter pollution | 0.541511 | 467.7 (247.78 to 715.95) | 54.31 | 413.39 |
| Thailand | Particulate matter pollution | 0.682548 | 61.7 (32.6 to 96.5) | 18.55 | 43.14 |
| Timor-Leste | Particulate matter pollution | 0.444668 | 623.3 (374.45 to 886.15) | 308.17 | 315.13 |
| Togo | Particulate matter pollution | 0.408534 | 1132.24 (728.05 to 1641.84) | 318.22 | 814.02 |
| Tokelau | Particulate matter pollution | 0.686426 | 109.94 (0.02 to 272.45) | 19.1 | 90.83 |
| Tonga | Particulate matter pollution | 0.62635 | 116.21 (51.81 to 186.2) | 29.96 | 86.25 |
| Trinidad and Tobago | Particulate matter pollution | 0.768763 | 149.82 (50.85 to 297.04) | 6.68 | 143.14 |
| Tunisia | Particulate matter pollution | 0.682432 | 112.01 (49.92 to 177.74) | 19.11 | 92.9 |
| Turkey | Particulate matter pollution | 0.712693 | 93.71 (42.95 to 153.35) | 14.1 | 79.61 |
| Turkmenistan | Particulate matter pollution | 0.682161 | 170.95 (56.57 to 296.63) | 18.93 | 152.02 |
| Tuvalu | Particulate matter pollution | 0.576621 | 69.95 (9.04 to 157.37) | 40.99 | 28.96 |
| Uganda | Particulate matter pollution | 0.423261 | 1058.85 (704.17 to 1508.92) | 311.19 | 747.66 |
| Ukraine | Particulate matter pollution | 0.760774 | 42.15 (6.92 to 82.05) | 6.75 | 35.39 |
| United Arab Emirates | Particulate matter pollution | 0.849318 | 63.56 (38.96 to 98.16) | 4.19 | 59.37 |
| United Kingdom | Particulate matter pollution | 0.859 | 20.24 (15.89 to 24.93) | 4.35 | 15.88 |
| United Republic of Tanzania | Particulate matter pollution | 0.446568 | 1047.96 (651.57 to 1561.14) | 301.93 | 746.02 |
| United States Virgin Islands | Particulate matter pollution | 0.821831 | 17.36 (1.28 to 44.82) | 6.49 | 10.87 |
| United States of America | Particulate matter pollution | 0.862448 | 17.65 (12.53 to 23.68) | 3.98 | 13.66 |
| Uruguay | Particulate matter pollution | 0.719283 | 24.7 (4.64 to 54.57) | 14.62 | 10.08 |
| Uzbekistan | Particulate matter pollution | 0.662622 | 253.84 (133.79 to 405.05) | 20.04 | 233.8 |
| Vanuatu | Particulate matter pollution | 0.473101 | 418.21 (271.64 to 606.23) | 293.76 | 124.45 |
| Venezuela (Bolivarian Republic of) | Particulate matter pollution | 0.596513 | 149.55 (50.05 to 276.31) | 40.83 | 108.72 |
| Viet Nam | Particulate matter pollution | 0.627934 | 182.39 (89.74 to 293.49) | 28.98 | 153.41 |
| Yemen | Particulate matter pollution | 0.450376 | 786.04 (522.53 to 1168.97) | 297.28 | 488.76 |
| Zambia | Particulate matter pollution | 0.505949 | 857.42 (515.21 to 1263.76) | 217.54 | 639.89 |
| Zimbabwe | Particulate matter pollution | 0.473819 | 1014.66 (646.2 to 1454.45) | 293.27 | 721.39 |
| Afghanistan | Household air pollution from solid fuels | 0.3372 | 657.04 (396.75 to 945.84) | 431.17 | 225.87 |
| Albania | Household air pollution from solid fuels | 0.70685 | 35.09 (3.05 to 112.07) | 0.02 | 35.07 |
| Algeria | Household air pollution from solid fuels | 0.659501 | 0.44 (0.01 to 2.75) | 0.07 | 0.37 |
| American Samoa | Household air pollution from solid fuels | 0.723728 | 5.49 (0.09 to 25.18) | 0.01 | 5.48 |
| Andorra | Household air pollution from solid fuels | 0.869444 | 0 (0 to 0) | 0 | 0 |
| Angola | Household air pollution from solid fuels | 0.453722 | 348 (126.74 to 650.19) | 47.56 | 300.44 |
| Antigua and Barbuda | Household air pollution from solid fuels | 0.749887 | 0.4 (0 to 3.79) | 0.01 | 0.4 |
| Argentina | Household air pollution from solid fuels | 0.723123 | 1.39 (0.01 to 11.03) | 0.01 | 1.38 |
| Armenia | Household air pollution from solid fuels | 0.701833 | 6.71 (0.61 to 24.93) | 0.02 | 6.69 |
| Australia | Household air pollution from solid fuels | 0.844253 | 0.01 (0 to 0.04) | 0 | 0.01 |
| Austria | Household air pollution from solid fuels | 0.853837 | 0.01 (0 to 0.03) | 0 | 0.01 |
| Azerbaijan | Household air pollution from solid fuels | 0.694851 | 13.73 (0.21 to 83.45) | 0.02 | 13.7 |
| Bahamas | Household air pollution from solid fuels | 0.805021 | 0.27 (0 to 2.62) | 0 | 0.27 |
| Bahrain | Household air pollution from solid fuels | 0.753043 | 0.02 (0 to 0.11) | 0.01 | 0.01 |
| Bangladesh | Household air pollution from solid fuels | 0.492421 | 835.82 (522.22 to 1258.21) | 16.31 | 819.51 |
| Barbados | Household air pollution from solid fuels | 0.746749 | 0.15 (0 to 1.02) | 0.01 | 0.14 |
| Belarus | Household air pollution from solid fuels | 0.784485 | 0.13 (0 to 1.07) | 0 | 0.13 |
| Belgium | Household air pollution from solid fuels | 0.853654 | 0 (0 to 0.03) | 0 | 0 |
| Belize | Household air pollution from solid fuels | 0.610229 | 52.1 (7.63 to 138.59) | 0.26 | 51.84 |
| Benin | Household air pollution from solid fuels | 0.373487 | 1255.94 (765.69 to 1803.9) | 323.57 | 932.37 |
| Bermuda | Household air pollution from solid fuels | 0.821365 | 0.01 (0 to 0.07) | 0 | 0.01 |
| Bhutan | Household air pollution from solid fuels | 0.473062 | 140.07 (27.4 to 361.94) | 12.1 | 127.97 |
| Bolivia (Plurinational State of) | Household air pollution from solid fuels | 0.599011 | 94.25 (25.46 to 198.87) | 0.35 | 93.9 |
| Bosnia and Herzegovina | Household air pollution from solid fuels | 0.723078 | 28.07 (3.51 to 80.68) | 0.01 | 28.06 |
| Botswana | Household air pollution from solid fuels | 0.642722 | 95.52 (2.69 to 410.04) | 0.25 | 95.27 |
| Brazil | Household air pollution from solid fuels | 0.653044 | 17.84 (6.46 to 35.76) | 0.14 | 17.7 |
| Brunei Darussalam | Household air pollution from solid fuels | 0.810234 | 0.05 (0 to 0.06) | 0 | 0.04 |
| Bulgaria | Household air pollution from solid fuels | 0.768151 | 2.97 (0.03 to 20) | 0.01 | 2.97 |
| Burkina Faso | Household air pollution from solid fuels | 0.285118 | 1045.03 (695.87 to 1446.22) | 760.98 | 284.05 |
| Burundi | Household air pollution from solid fuels | 0.289374 | 1041.92 (649.45 to 1531.07) | 617.72 | 424.2 |
| Cabo Verde | Household air pollution from solid fuels | 0.533535 | 93.8 (25.87 to 209.53) | 1.17 | 92.63 |
| Cambodia | Household air pollution from solid fuels | 0.473621 | 525.82 (324.47 to 778.66) | 10.85 | 514.97 |
| Cameroon | Household air pollution from solid fuels | 0.479691 | 766.87 (436.02 to 1123.01) | 28.63 | 738.23 |
| Canada | Household air pollution from solid fuels | 0.873171 | 0 (0 to 0.02) | 0 | 0 |
| Central African Republic | Household air pollution from solid fuels | 0.309168 | 1599.06 (1014.53 to 2296.08) | 588.73 | 1010.32 |
| Chad | Household air pollution from solid fuels | 0.240436 | 1231.27 (798.26 to 1783.09) | 942.96 | 288.3 |
| Chile | Household air pollution from solid fuels | 0.771515 | 2.45 (0.01 to 25.14) | 0.01 | 2.45 |
| China | Household air pollution from solid fuels | 0.72163 | 19.45 (5.13 to 47.25) | 0.01 | 19.43 |
| Colombia | Household air pollution from solid fuels | 0.655443 | 7.48 (0.33 to 31.94) | 0.06 | 7.42 |
| Comoros | Household air pollution from solid fuels | 0.475979 | 1194.94 (754.98 to 1726.28) | 11.51 | 1183.43 |
| Congo | Household air pollution from solid fuels | 0.583075 | 409.95 (201.25 to 698.01) | 0.3 | 409.64 |
| Cook Islands | Household air pollution from solid fuels | 0.77911 | 0.22 (0 to 2.32) | 0 | 0.22 |
| Costa Rica | Household air pollution from solid fuels | 0.70034 | 3.52 (0.08 to 19.97) | 0.02 | 3.5 |
| Coted'Ivoire | Household air pollution from solid fuels | 0.425942 | 1076.47 (680.53 to 1599.61) | 136.86 | 939.61 |
| Croatia | Household air pollution from solid fuels | 0.798341 | 0.51 (0 to 4.85) | 0 | 0.51 |
| Cuba | Household air pollution from solid fuels | 0.66873 | 1.26 (0.11 to 5.1) | 0.03 | 1.23 |
| Cyprus | Household air pollution from solid fuels | 0.835631 | 0.01 (0 to 0.04) | 0 | 0 |
| Czechia | Household air pollution from solid fuels | 0.82845 | 0.04 (0 to 0.34) | 0 | 0.04 |
| Democratic People's Republic of Korea | Household air pollution from solid fuels | 0.569855 | 177.06 (105.31 to 261.27) | 0.5 | 176.56 |
| Democratic Republic of the Congo | Household air pollution from solid fuels | 0.38318 | 841.54 (515.65 to 1211.07) | 320.69 | 520.85 |
| Denmark | Household air pollution from solid fuels | 0.896424 | 0 (0 to 0.01) | 0 | 0 |
| Djibouti | Household air pollution from solid fuels | 0.487958 | 242.22 (95.31 to 457.39) | 16.41 | 225.81 |
| Dominica | Household air pollution from solid fuels | 0.746967 | 42.52 (2.86 to 154.3) | 0.01 | 42.51 |
| Dominican Republic | Household air pollution from solid fuels | 0.619388 | 24.79 (0.65 to 150.08) | 0.21 | 24.58 |
| Ecuador | Household air pollution from solid fuels | 0.661017 | 9.28 (0.63 to 38.78) | 0.03 | 9.25 |
| Egypt | Household air pollution from solid fuels | 0.606787 | 0.2 (0.01 to 0.86) | 0.2 | 0 |
| El Salvador | Household air pollution from solid fuels | 0.563775 | 25.91 (5.56 to 68.22) | 0.65 | 25.26 |
| Equatorial Guinea | Household air pollution from solid fuels | 0.657857 | 13.83 (0.01 to 136.09) | 0.04 | 13.79 |
| Eritrea | Household air pollution from solid fuels | 0.403864 | 709.1 (439.48 to 1022.3) | 206.16 | 502.93 |
| Estonia | Household air pollution from solid fuels | 0.844918 | 0.14 (0 to 1.2) | 0 | 0.14 |
| Eswatini | Household air pollution from solid fuels | 0.58546 | 267.83 (77.2 to 530.54) | 0.41 | 267.43 |
| Ethiopia | Household air pollution from solid fuels | 0.358823 | 1211.54 (915.61 to 1614.5) | 364.01 | 847.53 |
| Fiji | Household air pollution from solid fuels | 0.675052 | 80.38 (14.09 to 195.21) | 0.03 | 80.35 |
| Finland | Household air pollution from solid fuels | 0.859831 | 0 (0 to 0.02) | 0 | 0 |
| France | Household air pollution from solid fuels | 0.838365 | 0.01 (0 to 0.05) | 0 | 0.01 |
| Gabon | Household air pollution from solid fuels | 0.634691 | 22.74 (0.47 to 130.14) | 0.22 | 22.51 |
| Gambia | Household air pollution from solid fuels | 0.409714 | 1011.45 (665.48 to 1414.62) | 160.81 | 850.64 |
| Georgia | Household air pollution from solid fuels | 0.732474 | 26.42 (3.68 to 74.08) | 0.01 | 26.4 |
| Germany | Household air pollution from solid fuels | 0.902957 | 0 (0 to 0.02) | 0 | 0 |
| Ghana | Household air pollution from solid fuels | 0.56493 | 605.25 (319.83 to 987.87) | 0.68 | 604.57 |
| Greece | Household air pollution from solid fuels | 0.791854 | 0.05 (0 to 0.55) | 0 | 0.05 |
| Greenland | Household air pollution from solid fuels | 0.82621 | 0.01 (0 to 0.01) | 0 | 0 |
| Grenada | Household air pollution from solid fuels | 0.668993 | 4.06 (0.12 to 24.92) | 0.03 | 4.03 |
| Guam | Household air pollution from solid fuels | 0.803982 | 0.33 (0 to 2.23) | 0 | 0.33 |
| Guatemala | Household air pollution from solid fuels | 0.539972 | 172.44 (76.9 to 296.28) | 1.43 | 171.01 |
| Guinea | Household air pollution from solid fuels | 0.336401 | 1151.26 (737.68 to 1658.77) | 538.03 | 613.23 |
| Guinea-Bissau | Household air pollution from solid fuels | 0.35311 | 1265.43 (778.67 to 1799.53) | 399.59 | 865.84 |
| Guyana | Household air pollution from solid fuels | 0.650812 | 60.06 (9.07 to 170.13) | 0.22 | 59.84 |
| Haiti | Household air pollution from solid fuels | 0.448278 | 1015.55 (667.9 to 1396.26) | 66.64 | 948.91 |
| Honduras | Household air pollution from solid fuels | 0.513037 | 196.71 (111.76 to 303.46) | 2.85 | 193.87 |
| Hungary | Household air pollution from solid fuels | 0.790755 | 0.89 (0 to 10.27) | 0 | 0.88 |
| Iceland | Household air pollution from solid fuels | 0.876362 | 0 (0 to 0.01) | 0 | 0 |
| India | Household air pollution from solid fuels | 0.575402 | 683.15 (482.04 to 908.69) | 0.39 | 682.75 |
| Indonesia | Household air pollution from solid fuels | 0.656868 | 134.81 (66.52 to 235.11) | 0.07 | 134.74 |
| Iran (Islamic Republic of) | Household air pollution from solid fuels | 0.697207 | 0.35 (0.02 to 1.7) | 0.02 | 0.33 |
| Iraq | Household air pollution from solid fuels | 0.662626 | 2.04 (0.03 to 13.92) | 0.03 | 2.01 |
| Ireland | Household air pollution from solid fuels | 0.873754 | 0 (0 to 0) | 0 | 0 |
| Israel | Household air pollution from solid fuels | 0.809012 | 0 (0 to 0.03) | 0 | 0 |
| Italy | Household air pollution from solid fuels | 0.805774 | 0.02 (0 to 0.18) | 0 | 0.02 |
| Jamaica | Household air pollution from solid fuels | 0.683263 | 59.03 (8.71 to 153.25) | 0.03 | 59 |
| Japan | Household air pollution from solid fuels | 0.871242 | 0 (0 to 0.03) | 0 | 0 |
| Jordan | Household air pollution from solid fuels | 0.725307 | 0.13 (0.01 to 0.54) | 0.01 | 0.12 |
| Kazakhstan | Household air pollution from solid fuels | 0.725144 | 1.34 (0 to 12.8) | 0.02 | 1.33 |
| Kenya | Household air pollution from solid fuels | 0.523768 | 668.88 (529.25 to 837.79) | 2.25 | 666.64 |
| Kiribati | Household air pollution from solid fuels | 0.527187 | 460.32 (266.08 to 694.51) | 1.56 | 458.77 |
| Kuwait | Household air pollution from solid fuels | 0.846651 | 0.01 (0 to 0.01) | 0 | 0.01 |
| Kyrgyzstan | Household air pollution from solid fuels | 0.603979 | 165.22 (76.57 to 278.22) | 0.34 | 164.88 |
| Lao People's Democratic Republic | Household air pollution from solid fuels | 0.489136 | 594.35 (307.7 to 932.61) | 9.73 | 584.62 |
| Latvia | Household air pollution from solid fuels | 0.830664 | 0.79 (0 to 6.71) | 0 | 0.79 |
| Lebanon | Household air pollution from solid fuels | 0.744746 | 0.34 (0.01 to 2.28) | 0.01 | 0.33 |
| Lesotho | Household air pollution from solid fuels | 0.510393 | 1083.95 (666.1 to 1569.28) | 2.81 | 1081.13 |
| Liberia | Household air pollution from solid fuels | 0.352442 | 1159.42 (751.46 to 1652.38) | 398.48 | 760.94 |
| Libya | Household air pollution from solid fuels | 0.725771 | 0.93 (0.03 to 6.43) | 0.02 | 0.92 |
| Lithuania | Household air pollution from solid fuels | 0.856484 | 0.1 (0 to 0.88) | 0 | 0.1 |
| Luxembourg | Household air pollution from solid fuels | 0.884429 | 0 (0 to 0) | 0 | 0 |
| Madagascar | Household air pollution from solid fuels | 0.400247 | 954.33 (647.39 to 1380.64) | 310.26 | 644.07 |
| Malawi | Household air pollution from solid fuels | 0.384554 | 967.11 (609.66 to 1305.31) | 318.36 | 648.75 |
| Malaysia | Household air pollution from solid fuels | 0.742524 | 0.14 (0 to 1.15) | 0.01 | 0.14 |
| Maldives | Household air pollution from solid fuels | 0.650887 | 8.58 (0.11 to 44.3) | 0.22 | 8.37 |
| Mali | Household air pollution from solid fuels | 0.26858 | 1777.31 (1167.65 to 2390.45) | 867.94 | 909.37 |
| Malta | Household air pollution from solid fuels | 0.801585 | 0.02 (0 to 0.16) | 0 | 0.02 |
| Marshall Islands | Household air pollution from solid fuels | 0.574091 | 164.16 (79.63 to 269.66) | 0.58 | 163.58 |
| Mauritania | Household air pollution from solid fuels | 0.498945 | 504.11 (289.76 to 740.32) | 6.57 | 497.54 |
| Mauritius | Household air pollution from solid fuels | 0.71826 | 0.83 (0 to 7.01) | 0.02 | 0.82 |
| Mexico | Household air pollution from solid fuels | 0.664575 | 16.97 (3.02 to 47.66) | 0.03 | 16.94 |
| Micronesia (Federated States of) | Household air pollution from solid fuels | 0.587535 | 134.37 (56.57 to 223.73) | 0.34 | 134.03 |
| Monaco | Household air pollution from solid fuels | 0.908263 | 0 (0 to 0) | 0 | 0 |
| Mongolia | Household air pollution from solid fuels | 0.617622 | 61.3 (5.65 to 182.57) | 0.23 | 61.07 |
| Montenegro | Household air pollution from solid fuels | 0.795801 | 5.63 (0.1 to 26.22) | 0 | 5.63 |
| Morocco | Household air pollution from solid fuels | 0.562698 | 13.24 (2.09 to 42.43) | 0.61 | 12.63 |
| Mozambique | Household air pollution from solid fuels | 0.326463 | 1248.64 (800.45 to 1775.88) | 546.7 | 701.94 |
| Myanmar | Household air pollution from solid fuels | 0.533901 | 506.56 (283.08 to 771.23) | 1.48 | 505.07 |
| Namibia | Household air pollution from solid fuels | 0.617565 | 212.36 (38.71 to 491.08) | 0.22 | 212.13 |
| Nauru | Household air pollution from solid fuels | 0.625178 | 11.11 (0.15 to 48.99) | 0.27 | 10.84 |
| Nepal | Household air pollution from solid fuels | 0.433175 | 674.4 (361.51 to 1011.2) | 89.97 | 584.43 |
| Netherlands | Household air pollution from solid fuels | 0.888464 | 0 (0 to 0.01) | 0 | 0 |
| New Zealand | Household air pollution from solid fuels | 0.849442 | 0.02 (0 to 0.21) | 0 | 0.02 |
| Nicaragua | Household air pollution from solid fuels | 0.523958 | 185.56 (93.1 to 294.09) | 1.93 | 183.63 |
| Niger | Household air pollution from solid fuels | 0.168073 | 1052.73 (657.76 to 1483.91) | 1045.94 | 6.79 |
| Nigeria | Household air pollution from solid fuels | 0.503391 | 1092.84 (729.29 to 1502.94) | 8.64 | 1084.2 |
| Niue | Household air pollution from solid fuels | 0.726222 | 17.67 (0.77 to 80.85) | 0.01 | 17.66 |
| North Macedonia | Household air pollution from solid fuels | 0.75063 | 11.89 (0.39 to 50.86) | 0.01 | 11.89 |
| Northern Mariana Islands | Household air pollution from solid fuels | 0.771535 | 0.64 (0 to 5.82) | 0.01 | 0.63 |
| Norway | Household air pollution from solid fuels | 0.916133 | 0 (0 to 0) | 0 | 0 |
| Oman | Household air pollution from solid fuels | 0.773392 | 0.05 (0 to 0.28) | 0.01 | 0.04 |
| Pakistan | Household air pollution from solid fuels | 0.504029 | 1032.86 (592.1 to 1515.39) | 7.69 | 1025.17 |
| Palau | Household air pollution from solid fuels | 0.754047 | 0.11 (0 to 0.75) | 0.01 | 0.1 |
| Palestine | Household air pollution from solid fuels | 0.631012 | 9.28 (2.96 to 21.7) | 0.34 | 8.94 |
| Panama | Household air pollution from solid fuels | 0.708865 | 2.61 (0.01 to 23.49) | 0.02 | 2.59 |
| Papua New Guinea | Household air pollution from solid fuels | 0.417797 | 590.45 (375.92 to 844.42) | 131.73 | 458.72 |
| Paraguay | Household air pollution from solid fuels | 0.635718 | 62.29 (9.53 to 159.39) | 0.21 | 62.08 |
| Peru | Household air pollution from solid fuels | 0.662054 | 22.1 (1.39 to 92.93) | 0.03 | 22.06 |
| Philippines | Household air pollution from solid fuels | 0.651219 | 253.99 (172.23 to 359.18) | 0.05 | 253.94 |
| Poland | Household air pollution from solid fuels | 0.812043 | 0.98 (0 to 8.56) | 0 | 0.98 |
| Portugal | Household air pollution from solid fuels | 0.744152 | 0.03 (0 to 0.27) | 0.01 | 0.02 |
| Puerto Rico | Household air pollution from solid fuels | 0.825526 | 0.01 (0 to 0.06) | 0 | 0.01 |
| Qatar | Household air pollution from solid fuels | 0.846861 | 0 (0 to 0) | 0 | 0 |
| Republic of Korea | Household air pollution from solid fuels | 0.886675 | 0 (0 to 0.04) | 0 | 0 |
| Republic of Moldova | Household air pollution from solid fuels | 0.732215 | 28.01 (7.11 to 64.97) | 0.01 | 27.99 |
| Romania | Household air pollution from solid fuels | 0.768454 | 1.97 (0 to 14.99) | 0.01 | 1.96 |
| Russian Federation | Household air pollution from solid fuels | 0.808536 | 0.37 (0 to 2.78) | 0 | 0.37 |
| Rwanda | Household air pollution from solid fuels | 0.435589 | 720.04 (461.23 to 1039.46) | 88.01 | 632.03 |
| Saint Kitts and Nevis | Household air pollution from solid fuels | 0.754987 | 0.81 (0 to 7.75) | 0.01 | 0.8 |
| Saint Lucia | Household air pollution from solid fuels | 0.67251 | 12.55 (0.43 to 62.17) | 0.03 | 12.53 |
| Saint Vincent and the Grenadines | Household air pollution from solid fuels | 0.637196 | 9.3 (0.49 to 39.65) | 0.27 | 9.04 |
| Samoa | Household air pollution from solid fuels | 0.593393 | 169.08 (85.09 to 261.52) | 0.36 | 168.72 |
| San Marino | Household air pollution from solid fuels | 0.888005 | 0 (0 to 0) | 0 | 0 |
| Sao Tome and Principe | Household air pollution from solid fuels | 0.505414 | 222.2 (121.49 to 343.27) | 6.26 | 215.94 |
| Saudi Arabia | Household air pollution from solid fuels | 0.815143 | 0.01 (0 to 0.05) | 0 | 0.01 |
| Senegal | Household air pollution from solid fuels | 0.408054 | 863.83 (548.49 to 1238.09) | 289.1 | 574.72 |
| Serbia | Household air pollution from solid fuels | 0.792416 | 8.94 (0.33 to 41.3) | 0 | 8.94 |
| Seychelles | Household air pollution from solid fuels | 0.730151 | 0.21 (0 to 2.18) | 0.01 | 0.19 |
| Sierra Leone | Household air pollution from solid fuels | 0.358666 | 1441.17 (983.87 to 1964.62) | 376.92 | 1064.25 |
| Singapore | Household air pollution from solid fuels | 0.856098 | 0 (0 to 0) | 0 | 0 |
| Slovakia | Household air pollution from solid fuels | 0.810611 | 0.07 (0 to 0.53) | 0 | 0.07 |
| Slovenia | Household air pollution from solid fuels | 0.842431 | 0.14 (0 to 1.56) | 0 | 0.14 |
| Solomon Islands | Household air pollution from solid fuels | 0.42936 | 266.25 (156.16 to 391.2) | 117.25 | 149 |
| Somalia | Household air pollution from solid fuels | 0.077688 | 1340.57 (885.25 to 1859.72) | 1340.57 | 0 |
| South Africa | Household air pollution from solid fuels | 0.679627 | 107.51 (26.98 to 245.44) | 0.03 | 107.48 |
| South Sudan | Household air pollution from solid fuels | 0.278371 | 1756.94 (1149.14 to 2457.86) | 909.36 | 847.58 |
| Spain | Household air pollution from solid fuels | 0.769284 | 0.02 (0 to 0.26) | 0.01 | 0.02 |
| Sri Lanka | Household air pollution from solid fuels | 0.701535 | 44.08 (6.97 to 105.27) | 0.02 | 44.06 |
| Sudan | Household air pollution from solid fuels | 0.54195 | 372.14 (169.53 to 654.5) | 1.73 | 370.41 |
| Suriname | Household air pollution from solid fuels | 0.633666 | 22.83 (0.62 to 132.8) | 0.22 | 22.61 |
| Sweden | Household air pollution from solid fuels | 0.88688 | 0 (0 to 0.01) | 0 | 0 |
| Switzerland | Household air pollution from solid fuels | 0.933059 | 0 (0 to 0) | 0 | 0 |
| Syrian Arab Republic | Household air pollution from solid fuels | 0.623004 | 0.55 (0.16 to 1.46) | 0.27 | 0.29 |
| Taiwan (Province of China) | Household air pollution from solid fuels | 0.874747 | 0.17 (0 to 1.32) | 0 | 0.17 |
| Tajikistan | Household air pollution from solid fuels | 0.541511 | 309.7 (142.37 to 496.91) | 1.29 | 308.41 |
| Thailand | Household air pollution from solid fuels | 0.682548 | 4.29 (0.08 to 26.79) | 0.03 | 4.26 |
| Timor-Leste | Household air pollution from solid fuels | 0.444668 | 491.28 (260.31 to 751.56) | 43.01 | 448.28 |
| Togo | Household air pollution from solid fuels | 0.408534 | 969.14 (618.54 to 1399.15) | 251.96 | 717.18 |
| Tokelau | Household air pollution from solid fuels | 0.686426 | 0.85 (0.01 to 3.51) | 0.03 | 0.82 |
| Tonga | Household air pollution from solid fuels | 0.62635 | 87.76 (36.11 to 151.34) | 0.26 | 87.5 |
| Trinidad and Tobago | Household air pollution from solid fuels | 0.768763 | 0.04 (0 to 0.43) | 0.01 | 0.04 |
| Tunisia | Household air pollution from solid fuels | 0.682432 | 0.45 (0.03 to 2.21) | 0.03 | 0.43 |
| Turkey | Household air pollution from solid fuels | 0.712693 | 0.54 (0 to 4.67) | 0.02 | 0.52 |
| Turkmenistan | Household air pollution from solid fuels | 0.682161 | 0.18 (0 to 1.52) | 0.03 | 0.15 |
| Tuvalu | Household air pollution from solid fuels | 0.576621 | 58.64 (7.56 to 131.52) | 0.4 | 58.24 |
| Uganda | Household air pollution from solid fuels | 0.423261 | 952.79 (635.15 to 1362.3) | 144.12 | 808.67 |
| Ukraine | Household air pollution from solid fuels | 0.760774 | 4.05 (0.29 to 16.37) | 0.01 | 4.05 |
| United Arab Emirates | Household air pollution from solid fuels | 0.849318 | 0 (0 to 0) | 0 | 0 |
| United Kingdom | Household air pollution from solid fuels | 0.859 | 0 (0 to 0.03) | 0 | 0 |
| United Republic of Tanzania | Household air pollution from solid fuels | 0.446568 | 959.56 (597.44 to 1437.55) | 48.34 | 911.22 |
| United States Virgin Islands | Household air pollution from solid fuels | 0.821831 | 0.02 (0 to 0.08) | 0 | 0.02 |
| United States of America | Household air pollution from solid fuels | 0.862448 | 0.01 (0 to 0.05) | 0 | 0.01 |
| Uruguay | Household air pollution from solid fuels | 0.719283 | 0.56 (0 to 4.71) | 0.02 | 0.55 |
| Uzbekistan | Household air pollution from solid fuels | 0.662622 | 47.97 (8.7 to 137.07) | 0.04 | 47.94 |
| Vanuatu | Household air pollution from solid fuels | 0.473101 | 392.07 (253.05 to 566.65) | 23.53 | 368.54 |
| Venezuela (Bolivarian Republic of) | Household air pollution from solid fuels | 0.596513 | 2.92 (0.08 to 18.24) | 0.52 | 2.4 |
| Viet Nam | Household air pollution from solid fuels | 0.627934 | 113.6 (47.09 to 205.12) | 0.22 | 113.38 |
| Yemen | Household air pollution from solid fuels | 0.450376 | 444.91 (263.99 to 688.86) | 22.72 | 422.19 |
| Zambia | Household air pollution from solid fuels | 0.505949 | 737.58 (432.72 to 1117.71) | 4.98 | 732.6 |
| Zimbabwe | Household air pollution from solid fuels | 0.473819 | 928.05 (591.4 to 1338.01) | 13.95 | 914.09 |
| Afghanistan | Ambient particulate matter pollution | 0.3372 | 94.41 (50.19 to 155.35) | 18.03 | 76.38 |
| Albania | Ambient particulate matter pollution | 0.70685 | 95.17 (24.18 to 190.02) | 10 | 85.17 |
| Algeria | Ambient particulate matter pollution | 0.659501 | 190.89 (81.91 to 323.93) | 9.74 | 181.15 |
| American Samoa | Ambient particulate matter pollution | 0.723728 | 23.11 (2.43 to 55.92) | 9.57 | 13.54 |
| Andorra | Ambient particulate matter pollution | 0.869444 | 2.57 (0.02 to 6.88) | 2.57 | 0 |
| Angola | Ambient particulate matter pollution | 0.453722 | 294.23 (102.97 to 573.09) | 13.01 | 281.22 |
| Antigua and Barbuda | Ambient particulate matter pollution | 0.749887 | 82.5 (29.02 to 141.06) | 6.58 | 75.92 |
| Argentina | Ambient particulate matter pollution | 0.723123 | 62.59 (17.52 to 116.41) | 9.56 | 53.03 |
| Armenia | Ambient particulate matter pollution | 0.701833 | 124.91 (66.01 to 195.68) | 9.97 | 114.94 |
| Australia | Ambient particulate matter pollution | 0.844253 | 11.38 (0.49 to 29.83) | 5.15 | 6.23 |
| Austria | Ambient particulate matter pollution | 0.853837 | 14.92 (1.11 to 33.61) | 4.4 | 10.52 |
| Azerbaijan | Ambient particulate matter pollution | 0.694851 | 265.53 (100.79 to 477.76) | 9.97 | 255.56 |
| Bahamas | Ambient particulate matter pollution | 0.805021 | 80.05 (28.86 to 147.54) | 6.29 | 73.75 |
| Bahrain | Ambient particulate matter pollution | 0.753043 | 70.23 (44.11 to 103.31) | 7.79 | 62.44 |
| Bangladesh | Ambient particulate matter pollution | 0.492421 | 143.74 (66.61 to 276.26) | 13.03 | 130.7 |
| Barbados | Ambient particulate matter pollution | 0.746749 | 161.02 (59.8 to 301.32) | 6.69 | 154.33 |
| Belarus | Ambient particulate matter pollution | 0.784485 | 18.34 (3.72 to 35.77) | 6.08 | 12.26 |
| Belgium | Ambient particulate matter pollution | 0.853654 | 15.29 (1.72 to 34.86) | 4.37 | 10.92 |
| Belize | Ambient particulate matter pollution | 0.610229 | 164.44 (67.22 to 291.28) | 9.91 | 154.53 |
| Benin | Ambient particulate matter pollution | 0.373487 | 220.83 (109.63 to 379.51) | 13.08 | 207.74 |
| Bermuda | Ambient particulate matter pollution | 0.821365 | 8.35 (0.02 to 24.09) | 6.01 | 2.34 |
| Bhutan | Ambient particulate matter pollution | 0.473062 | 370.64 (153.83 to 596.44) | 13.14 | 357.5 |
| Bolivia (Plurinational State of) | Ambient particulate matter pollution | 0.599011 | 144.71 (61.61 to 261.08) | 9.97 | 134.74 |
| Bosnia and Herzegovina | Ambient particulate matter pollution | 0.723078 | 75.77 (20.68 to 134.49) | 9.73 | 66.04 |
| Botswana | Ambient particulate matter pollution | 0.642722 | 419.63 (117.78 to 793.97) | 9.97 | 409.66 |
| Brazil | Ambient particulate matter pollution | 0.653044 | 59.63 (34.07 to 84.97) | 9.97 | 49.66 |
| Brunei Darussalam | Ambient particulate matter pollution | 0.810234 | 27.27 (2.57 to 63.12) | 5.92 | 21.35 |
| Bulgaria | Ambient particulate matter pollution | 0.768151 | 39.22 (10.02 to 74.9) | 6.22 | 33 |
| Burkina Faso | Ambient particulate matter pollution | 0.285118 | 169.94 (91.3 to 288.65) | 28.13 | 141.81 |
| Burundi | Ambient particulate matter pollution | 0.289374 | 62.57 (30 to 112.91) | 28.42 | 34.16 |
| Cabo Verde | Ambient particulate matter pollution | 0.533535 | 215.05 (93.21 to 365.74) | 10.97 | 204.09 |
| Cambodia | Ambient particulate matter pollution | 0.473621 | 81.4 (31.6 to 162.5) | 13.04 | 68.37 |
| Cameroon | Ambient particulate matter pollution | 0.479691 | 194.04 (87.97 to 365.92) | 13.06 | 180.98 |
| Canada | Ambient particulate matter pollution | 0.873171 | 12.86 (0.01 to 35.89) | 3.58 | 9.28 |
| Central African Republic | Ambient particulate matter pollution | 0.309168 | 126.53 (66.52 to 206.28) | 28.33 | 98.2 |
| Chad | Ambient particulate matter pollution | 0.240436 | 219.64 (111.13 to 399.48) | 55.77 | 163.87 |
| Chile | Ambient particulate matter pollution | 0.771515 | 56.8 (19.87 to 97.21) | 6.06 | 50.73 |
| China | Ambient particulate matter pollution | 0.72163 | 58.55 (32.35 to 81.92) | 9.52 | 49.03 |
| Colombia | Ambient particulate matter pollution | 0.655443 | 59.58 (18.51 to 115.23) | 9.9 | 49.68 |
| Comoros | Ambient particulate matter pollution | 0.475979 | 59.57 (26.09 to 116.52) | 13.03 | 46.55 |
| Congo | Ambient particulate matter pollution | 0.583075 | 211.8 (68.45 to 429.23) | 9.91 | 201.89 |
| Cook Islands | Ambient particulate matter pollution | 0.77911 | 8.27 (0.67 to 20.37) | 6.35 | 1.92 |
| Costa Rica | Ambient particulate matter pollution | 0.70034 | 47.75 (6.14 to 100.13) | 10 | 37.75 |
| Coted'Ivoire | Ambient particulate matter pollution | 0.425942 | 328.17 (152.94 to 630.28) | 13.05 | 315.12 |
| Croatia | Ambient particulate matter pollution | 0.798341 | 26.49 (4.93 to 54.82) | 6.17 | 20.32 |
| Cuba | Ambient particulate matter pollution | 0.66873 | 24.29 (6.41 to 42.88) | 9.73 | 14.55 |
| Cyprus | Ambient particulate matter pollution | 0.835631 | 12.77 (3.89 to 23.96) | 5.53 | 7.24 |
| Czechia | Ambient particulate matter pollution | 0.82845 | 12.42 (2.2 to 25.55) | 5.85 | 6.56 |
| Democratic People's Republic of Korea | Ambient particulate matter pollution | 0.569855 | 19.71 (10.34 to 33.1) | 9.9 | 9.8 |
| Democratic Republic of the Congo | Ambient particulate matter pollution | 0.38318 | 60.67 (31.41 to 101.96) | 13.02 | 47.64 |
| Denmark | Ambient particulate matter pollution | 0.896424 | 15.65 (0.28 to 38.8) | 3.62 | 12.02 |
| Djibouti | Ambient particulate matter pollution | 0.487958 | 302.85 (136.88 to 550.05) | 13.01 | 289.84 |
| Dominica | Ambient particulate matter pollution | 0.746967 | 278.64 (101.38 to 527.85) | 7.01 | 271.63 |
| Dominican Republic | Ambient particulate matter pollution | 0.619388 | 310.73 (116.93 to 550.19) | 9.91 | 300.81 |
| Ecuador | Ambient particulate matter pollution | 0.661017 | 80.63 (25.25 to 150.03) | 9.92 | 70.72 |
| Egypt | Ambient particulate matter pollution | 0.606787 | 107.75 (63.82 to 164.21) | 9.91 | 97.83 |
| El Salvador | Ambient particulate matter pollution | 0.563775 | 52.18 (15.28 to 106.85) | 9.73 | 42.45 |
| Equatorial Guinea | Ambient particulate matter pollution | 0.657857 | 469.7 (206.5 to 799.98) | 9.73 | 459.97 |
| Eritrea | Ambient particulate matter pollution | 0.403864 | 110.89 (51.28 to 209.66) | 13.05 | 97.84 |
| Estonia | Ambient particulate matter pollution | 0.844918 | 4.03 (0.19 to 10.27) | 3.97 | 0.05 |
| Eswatini | Ambient particulate matter pollution | 0.58546 | 225.78 (57.67 to 482.22) | 10 | 215.78 |
| Ethiopia | Ambient particulate matter pollution | 0.358823 | 93.97 (55.2 to 146.92) | 15.99 | 77.97 |
| Fiji | Ambient particulate matter pollution | 0.675052 | 86.68 (18.16 to 193.05) | 9.91 | 76.76 |
| Finland | Ambient particulate matter pollution | 0.859831 | 5.11 (0 to 15.62) | 4.23 | 0.88 |
| France | Ambient particulate matter pollution | 0.838365 | 14.97 (1.11 to 38.03) | 5.49 | 9.48 |
| Gabon | Ambient particulate matter pollution | 0.634691 | 373.1 (166.22 to 642.23) | 9.97 | 363.13 |
| Gambia | Ambient particulate matter pollution | 0.409714 | 191.74 (100.73 to 342.65) | 13.01 | 178.72 |
| Georgia | Ambient particulate matter pollution | 0.732474 | 65.45 (19.14 to 131.72) | 8.82 | 56.63 |
| Germany | Ambient particulate matter pollution | 0.902957 | 17.26 (2.27 to 36.78) | 3.31 | 13.94 |
| Ghana | Ambient particulate matter pollution | 0.56493 | 302.75 (115.68 to 584.2) | 9.9 | 292.85 |
| Greece | Ambient particulate matter pollution | 0.791854 | 25.17 (6.46 to 46.05) | 5.98 | 19.19 |
| Greenland | Ambient particulate matter pollution | 0.82621 | 24.45 (0.02 to 66.16) | 6.35 | 18.1 |
| Grenada | Ambient particulate matter pollution | 0.668993 | 177.42 (59.14 to 324.2) | 9.97 | 167.45 |
| Guam | Ambient particulate matter pollution | 0.803982 | 48.47 (5.04 to 105.4) | 6.09 | 42.38 |
| Guatemala | Ambient particulate matter pollution | 0.539972 | 98.87 (27.85 to 215.26) | 9.74 | 89.13 |
| Guinea | Ambient particulate matter pollution | 0.336401 | 177.58 (84.98 to 305.02) | 17.64 | 159.94 |
| Guinea-Bissau | Ambient particulate matter pollution | 0.35311 | 202.74 (103.93 to 357.86) | 17.25 | 185.49 |
| Guyana | Ambient particulate matter pollution | 0.650812 | 336.62 (130.64 to 635.49) | 9.74 | 326.88 |
| Haiti | Ambient particulate matter pollution | 0.448278 | 78.13 (31.89 to 159.47) | 13.03 | 65.1 |
| Honduras | Ambient particulate matter pollution | 0.513037 | 52.71 (18.34 to 112.09) | 11.47 | 41.24 |
| Hungary | Ambient particulate matter pollution | 0.790755 | 20.16 (4.11 to 40.8) | 5.8 | 14.35 |
| Iceland | Ambient particulate matter pollution | 0.876362 | 3.64 (0 to 10.24) | 3.61 | 0.02 |
| India | Ambient particulate matter pollution | 0.575402 | 363.37 (209.84 to 554.91) | 9.74 | 353.63 |
| Indonesia | Ambient particulate matter pollution | 0.656868 | 166.39 (74.06 to 261.44) | 9.91 | 156.48 |
| Iran (Islamic Republic of) | Ambient particulate matter pollution | 0.697207 | 80.01 (62.18 to 101.02) | 9.73 | 70.27 |
| Iraq | Ambient particulate matter pollution | 0.662626 | 319.54 (171.04 to 503.41) | 9.9 | 309.64 |
| Ireland | Ambient particulate matter pollution | 0.873754 | 10.16 (0.01 to 28.97) | 3.82 | 6.35 |
| Israel | Ambient particulate matter pollution | 0.809012 | 12.46 (4.3 to 23.2) | 5.9 | 6.56 |
| Italy | Ambient particulate matter pollution | 0.805774 | 16.22 (11.04 to 21.82) | 5.94 | 10.29 |
| Jamaica | Ambient particulate matter pollution | 0.683263 | 168.32 (56.74 to 329.39) | 9.91 | 158.4 |
| Japan | Ambient particulate matter pollution | 0.871242 | 4.76 (3.67 to 5.94) | 3.07 | 1.69 |
| Jordan | Ambient particulate matter pollution | 0.725307 | 151.97 (75.15 to 251.48) | 9.68 | 142.29 |
| Kazakhstan | Ambient particulate matter pollution | 0.725144 | 66.83 (26.33 to 113.88) | 9.74 | 57.09 |
| Kenya | Ambient particulate matter pollution | 0.523768 | 68.54 (37.09 to 118.67) | 10.98 | 57.56 |
| Kiribati | Ambient particulate matter pollution | 0.527187 | 30.86 (11.12 to 69.03) | 10.62 | 20.24 |
| Kuwait | Ambient particulate matter pollution | 0.846651 | 112.5 (59.44 to 171.26) | 3.78 | 108.72 |
| Kyrgyzstan | Ambient particulate matter pollution | 0.603979 | 94.78 (35.57 to 192.6) | 9.72 | 85.06 |
| Lao People's Democratic Republic | Ambient particulate matter pollution | 0.489136 | 175.04 (50.39 to 369.36) | 13.03 | 162.02 |
| Latvia | Ambient particulate matter pollution | 0.830664 | 16.48 (1.44 to 35.35) | 6.05 | 10.43 |
| Lebanon | Ambient particulate matter pollution | 0.744746 | 64.6 (25.51 to 116.65) | 7.57 | 57.04 |
| Lesotho | Ambient particulate matter pollution | 0.510393 | 163.6 (74.81 to 304.53) | 12.88 | 150.72 |
| Liberia | Ambient particulate matter pollution | 0.352442 | 127.75 (70.43 to 205.9) | 16.88 | 110.87 |
| Libya | Ambient particulate matter pollution | 0.725771 | 232.25 (113.22 to 381.37) | 9.72 | 222.53 |
| Lithuania | Ambient particulate matter pollution | 0.856484 | 9.95 (0.75 to 23.14) | 4.17 | 5.79 |
| Luxembourg | Ambient particulate matter pollution | 0.884429 | 9.3 (0.75 to 22.12) | 3.49 | 5.81 |
| Madagascar | Ambient particulate matter pollution | 0.400247 | 36.6 (20.16 to 63.52) | 13.03 | 23.57 |
| Malawi | Ambient particulate matter pollution | 0.384554 | 56.96 (31.44 to 97.26) | 13.02 | 43.93 |
| Malaysia | Ambient particulate matter pollution | 0.742524 | 43.78 (15.18 to 79.5) | 8.03 | 35.75 |
| Maldives | Ambient particulate matter pollution | 0.650887 | 77.75 (17.01 to 160.82) | 9.91 | 67.84 |
| Mali | Ambient particulate matter pollution | 0.26858 | 355.32 (184.34 to 636.11) | 58.2 | 297.11 |
| Malta | Ambient particulate matter pollution | 0.801585 | 27.84 (2.74 to 61.01) | 5.87 | 21.97 |
| Marshall Islands | Ambient particulate matter pollution | 0.574091 | 32.66 (10.77 to 73.09) | 9.71 | 22.95 |
| Mauritania | Ambient particulate matter pollution | 0.498945 | 378.81 (196.82 to 622.22) | 12.91 | 365.9 |
| Mauritius | Ambient particulate matter pollution | 0.71826 | 58.42 (11.91 to 119.23) | 9.91 | 48.5 |
| Mexico | Ambient particulate matter pollution | 0.664575 | 78.26 (45.31 to 107.06) | 9.91 | 68.34 |
| Micronesia (Federated States of) | Ambient particulate matter pollution | 0.587535 | 27.7 (8.58 to 65.73) | 9.74 | 17.96 |
| Monaco | Ambient particulate matter pollution | 0.908263 | 7.99 (0.79 to 18.06) | 3.62 | 4.38 |
| Mongolia | Ambient particulate matter pollution | 0.617622 | 149.48 (39.77 to 278.18) | 10 | 139.48 |
| Montenegro | Ambient particulate matter pollution | 0.795801 | 29.25 (5.72 to 57.12) | 6.41 | 22.84 |
| Morocco | Ambient particulate matter pollution | 0.562698 | 156.94 (63.71 to 257.94) | 9.97 | 146.98 |
| Mozambique | Ambient particulate matter pollution | 0.326463 | 69.96 (39.45 to 119.95) | 18.03 | 51.94 |
| Myanmar | Ambient particulate matter pollution | 0.533901 | 192.48 (62.06 to 393.34) | 10.61 | 181.87 |
| Namibia | Ambient particulate matter pollution | 0.617565 | 276.06 (68.98 to 562.73) | 9.74 | 266.32 |
| Nauru | Ambient particulate matter pollution | 0.625178 | 43.81 (0.84 to 112.75) | 9.74 | 34.06 |
| Nepal | Ambient particulate matter pollution | 0.433175 | 200.87 (101.29 to 365.25) | 13.15 | 187.73 |
| Netherlands | Ambient particulate matter pollution | 0.888464 | 18.56 (1.34 to 42.42) | 3.35 | 15.21 |
| New Zealand | Ambient particulate matter pollution | 0.849442 | 14.26 (1.59 to 32.45) | 4.59 | 9.67 |
| Nicaragua | Ambient particulate matter pollution | 0.523958 | 50.46 (17.18 to 110.45) | 12.06 | 38.4 |
| Niger | Ambient particulate matter pollution | 0.168073 | 173.46 (82.49 to 301.6) | 57.82 | 115.64 |
| Nigeria | Ambient particulate matter pollution | 0.503391 | 531.66 (246.55 to 935.25) | 12.34 | 519.32 |
| Niue | Ambient particulate matter pollution | 0.726222 | 85.68 (6.56 to 189.36) | 9.73 | 75.95 |
| North Macedonia | Ambient particulate matter pollution | 0.75063 | 79.04 (27.08 to 142.35) | 6.82 | 72.22 |
| Northern Mariana Islands | Ambient particulate matter pollution | 0.771535 | 26.17 (2.89 to 62.11) | 6.39 | 19.78 |
| Norway | Ambient particulate matter pollution | 0.916133 | 5.75 (3.17 to 9.38) | 3.35 | 2.4 |
| Oman | Ambient particulate matter pollution | 0.773392 | 127.42 (75.73 to 192.33) | 6.29 | 121.13 |
| Pakistan | Ambient particulate matter pollution | 0.504029 | 576.92 (275.37 to 1014.95) | 12.23 | 564.69 |
| Palau | Ambient particulate matter pollution | 0.754047 | 51.68 (2.12 to 127.56) | 6.84 | 44.84 |
| Palestine | Ambient particulate matter pollution | 0.631012 | 120.99 (61.28 to 207.36) | 9.74 | 111.25 |
| Panama | Ambient particulate matter pollution | 0.708865 | 54.54 (13.53 to 112.32) | 9.97 | 44.57 |
| Papua New Guinea | Ambient particulate matter pollution | 0.417797 | 58.38 (15.36 to 142.7) | 13.06 | 45.33 |
| Paraguay | Ambient particulate matter pollution | 0.635718 | 64.65 (10.53 to 147.4) | 9.92 | 54.74 |
| Peru | Ambient particulate matter pollution | 0.662054 | 130.64 (51.41 to 230.46) | 9.97 | 120.67 |
| Philippines | Ambient particulate matter pollution | 0.651219 | 129.4 (60.9 to 209.18) | 10 | 119.4 |
| Poland | Ambient particulate matter pollution | 0.812043 | 30.1 (19.95 to 41.35) | 6.04 | 24.06 |
| Portugal | Ambient particulate matter pollution | 0.744152 | 8.85 (0.67 to 21.43) | 7.65 | 1.21 |
| Puerto Rico | Ambient particulate matter pollution | 0.825526 | 19.81 (0.01 to 49.93) | 6.33 | 13.48 |
| Qatar | Ambient particulate matter pollution | 0.846861 | 62.92 (37.83 to 92.53) | 4.68 | 58.24 |
| Republic of Korea | Ambient particulate matter pollution | 0.886675 | 18.74 (6.88 to 33.16) | 3.41 | 15.33 |
| Republic of Moldova | Ambient particulate matter pollution | 0.732215 | 36.4 (9.77 to 77.22) | 9.74 | 26.66 |
| Romania | Ambient particulate matter pollution | 0.768454 | 38.98 (12.41 to 69.31) | 6.33 | 32.64 |
| Russian Federation | Ambient particulate matter pollution | 0.808536 | 21.79 (16.95 to 27) | 6.1 | 15.69 |
| Rwanda | Ambient particulate matter pollution | 0.435589 | 47.9 (19.64 to 93.5) | 13.03 | 34.87 |
| Saint Kitts and Nevis | Ambient particulate matter pollution | 0.754987 | 61.98 (3.6 to 147.97) | 7.43 | 54.55 |
| Saint Lucia | Ambient particulate matter pollution | 0.67251 | 251.82 (92.24 to 465.76) | 9.97 | 241.85 |
| Saint Vincent and the Grenadines | Ambient particulate matter pollution | 0.637196 | 171.59 (61.97 to 310.88) | 10 | 161.59 |
| Samoa | Ambient particulate matter pollution | 0.593393 | 33.66 (7.53 to 83.03) | 9.97 | 23.69 |
| San Marino | Ambient particulate matter pollution | 0.888005 | 5.59 (0.23 to 14.79) | 3.62 | 1.97 |
| Sao Tome and Principe | Ambient particulate matter pollution | 0.505414 | 60.19 (23.94 to 115.57) | 12.72 | 47.47 |
| Saudi Arabia | Ambient particulate matter pollution | 0.815143 | 62.27 (35.75 to 98.15) | 5.86 | 56.42 |
| Senegal | Ambient particulate matter pollution | 0.408054 | 147.71 (63.44 to 281.27) | 13.01 | 134.69 |
| Serbia | Ambient particulate matter pollution | 0.792416 | 58.68 (19.62 to 104.15) | 6.48 | 52.2 |
| Seychelles | Ambient particulate matter pollution | 0.730151 | 57.92 (6.17 to 121.41) | 8.98 | 48.94 |
| Sierra Leone | Ambient particulate matter pollution | 0.358666 | 171.13 (90.04 to 294.28) | 15.63 | 155.5 |
| Singapore | Ambient particulate matter pollution | 0.856098 | 8.68 (2.62 to 15.93) | 4.31 | 4.37 |
| Slovakia | Ambient particulate matter pollution | 0.810611 | 29.62 (8.04 to 55.75) | 6.41 | 23.2 |
| Slovenia | Ambient particulate matter pollution | 0.842431 | 9.01 (1.74 to 19.51) | 5.11 | 3.9 |
| Solomon Islands | Ambient particulate matter pollution | 0.42936 | 13.91 (4.12 to 32.46) | 13.03 | 0.88 |
| Somalia | Ambient particulate matter pollution | 0.077688 | 73.03 (42.94 to 118.27) | 58.14 | 14.89 |
| South Africa | Ambient particulate matter pollution | 0.679627 | 376.86 (231.43 to 538.49) | 9.97 | 366.89 |
| South Sudan | Ambient particulate matter pollution | 0.278371 | 208.06 (98.25 to 397.54) | 55.4 | 152.66 |
| Spain | Ambient particulate matter pollution | 0.769284 | 10.5 (1.34 to 23.11) | 6.27 | 4.23 |
| Sri Lanka | Ambient particulate matter pollution | 0.701535 | 60.99 (11.47 to 127.08) | 9.97 | 51.02 |
| Sudan | Ambient particulate matter pollution | 0.54195 | 299.14 (138.26 to 565.52) | 10.79 | 288.35 |
| Suriname | Ambient particulate matter pollution | 0.633666 | 382.27 (181.05 to 632.62) | 9.9 | 372.37 |
| Sweden | Ambient particulate matter pollution | 0.88688 | 5.1 (0.57 to 11.62) | 3.36 | 1.74 |
| Switzerland | Ambient particulate matter pollution | 0.933059 | 13.28 (0.32 to 32.17) | 3.51 | 9.77 |
| Syrian Arab Republic | Ambient particulate matter pollution | 0.623004 | 94.64 (46.6 to 151.55) | 9.74 | 84.9 |
| Taiwan (Province of China) | Ambient particulate matter pollution | 0.874747 | 25.99 (6.75 to 49.98) | 2.93 | 23.07 |
| Tajikistan | Ambient particulate matter pollution | 0.541511 | 157.92 (67.26 to 289.67) | 10.52 | 147.4 |
| Thailand | Ambient particulate matter pollution | 0.682548 | 57.36 (26.74 to 93.01) | 9.9 | 47.46 |
| Timor-Leste | Ambient particulate matter pollution | 0.444668 | 131.85 (31.47 to 312.8) | 13.03 | 118.82 |
| Togo | Ambient particulate matter pollution | 0.408534 | 163.01 (83.74 to 286.21) | 13.05 | 149.96 |
| Tokelau | Ambient particulate matter pollution | 0.686426 | 109.07 (0.03 to 271.1) | 9.97 | 99.09 |
| Tonga | Ambient particulate matter pollution | 0.62635 | 28.4 (6.87 to 65.02) | 9.91 | 18.49 |
| Trinidad and Tobago | Ambient particulate matter pollution | 0.768763 | 149.78 (50.85 to 296.95) | 6.41 | 143.37 |
| Tunisia | Ambient particulate matter pollution | 0.682432 | 111.55 (49.44 to 177.01) | 9.97 | 101.58 |
| Turkey | Ambient particulate matter pollution | 0.712693 | 93.15 (42.83 to 153.52) | 9.91 | 83.24 |
| Turkmenistan | Ambient particulate matter pollution | 0.682161 | 170.77 (56.56 to 296.52) | 9.92 | 160.85 |
| Tuvalu | Ambient particulate matter pollution | 0.576621 | 11.28 (1.26 to 29.38) | 9.91 | 1.37 |
| Uganda | Ambient particulate matter pollution | 0.423261 | 106.05 (55.91 to 180.57) | 13.05 | 93 |
| Ukraine | Ambient particulate matter pollution | 0.760774 | 38.08 (5.55 to 76.39) | 6.82 | 31.27 |
| United Arab Emirates | Ambient particulate matter pollution | 0.849318 | 63.55 (38.96 to 98.16) | 4.56 | 59 |
| United Kingdom | Ambient particulate matter pollution | 0.859 | 20.23 (15.89 to 24.93) | 4 | 16.23 |
| United Republic of Tanzania | Ambient particulate matter pollution | 0.446568 | 88.37 (40.05 to 172.11) | 13.01 | 75.36 |
| United States Virgin Islands | Ambient particulate matter pollution | 0.821831 | 17.33 (1.28 to 44.77) | 5.86 | 11.47 |
| United States of America | Ambient particulate matter pollution | 0.862448 | 17.64 (12.53 to 23.63) | 4.12 | 13.52 |
| Uruguay | Ambient particulate matter pollution | 0.719283 | 24.12 (4.2 to 53.65) | 9.74 | 14.39 |
| Uzbekistan | Ambient particulate matter pollution | 0.662622 | 205.75 (94.9 to 350.99) | 9.97 | 195.78 |
| Vanuatu | Ambient particulate matter pollution | 0.473101 | 26.09 (8.35 to 64.79) | 13.09 | 13 |
| Venezuela (Bolivarian Republic of) | Ambient particulate matter pollution | 0.596513 | 146.6 (49.5 to 272.82) | 9.73 | 136.87 |
| Viet Nam | Ambient particulate matter pollution | 0.627934 | 68.71 (22.29 to 141.34) | 9.74 | 58.97 |
| Yemen | Ambient particulate matter pollution | 0.450376 | 340.91 (173.81 to 555.1) | 13.06 | 327.85 |
| Zambia | Ambient particulate matter pollution | 0.505949 | 119.68 (49.58 to 247.49) | 12.55 | 107.13 |
| Zimbabwe | Ambient particulate matter pollution | 0.473819 | 86.61 (43.91 to 156.06) | 13.06 | 73.55 |

Frontier DALYs refer to the lowest achievable DALYs at a specific SDI level. Effective Difference refers to the gap between the actual DALYs of a country or region and Frontier DALYs.
